# Supplementary material for: CaMKII suppresses proteotoxicity by phosphorylating BAG3 in response to proteasomal dysfunction
Source: EMBO Rep. 2024 Sep 11;25(10):4488–514. doi: 10.1038/s44319-024-00248-w (PMC11466968; doi:10.1038/s44319-024-00248-w)
Supplement: Supplementary file 1 — Appendix [file 44319_2024_248_MOESM1_ESM.pdf]

Table of Contents

Appendix Fig. S1 2

Appendix Fig. S2 3

Appendix Fig. S3 4

Appendix Fig. S4 5

Appendix Table S1. Sequences of the primers used for ORF amplification. 6-7

Appendix Table S2. Sequences of shRNA and siRNA. 8

Appendix Table S3. Sequences of qRT-PCR Primer. 9

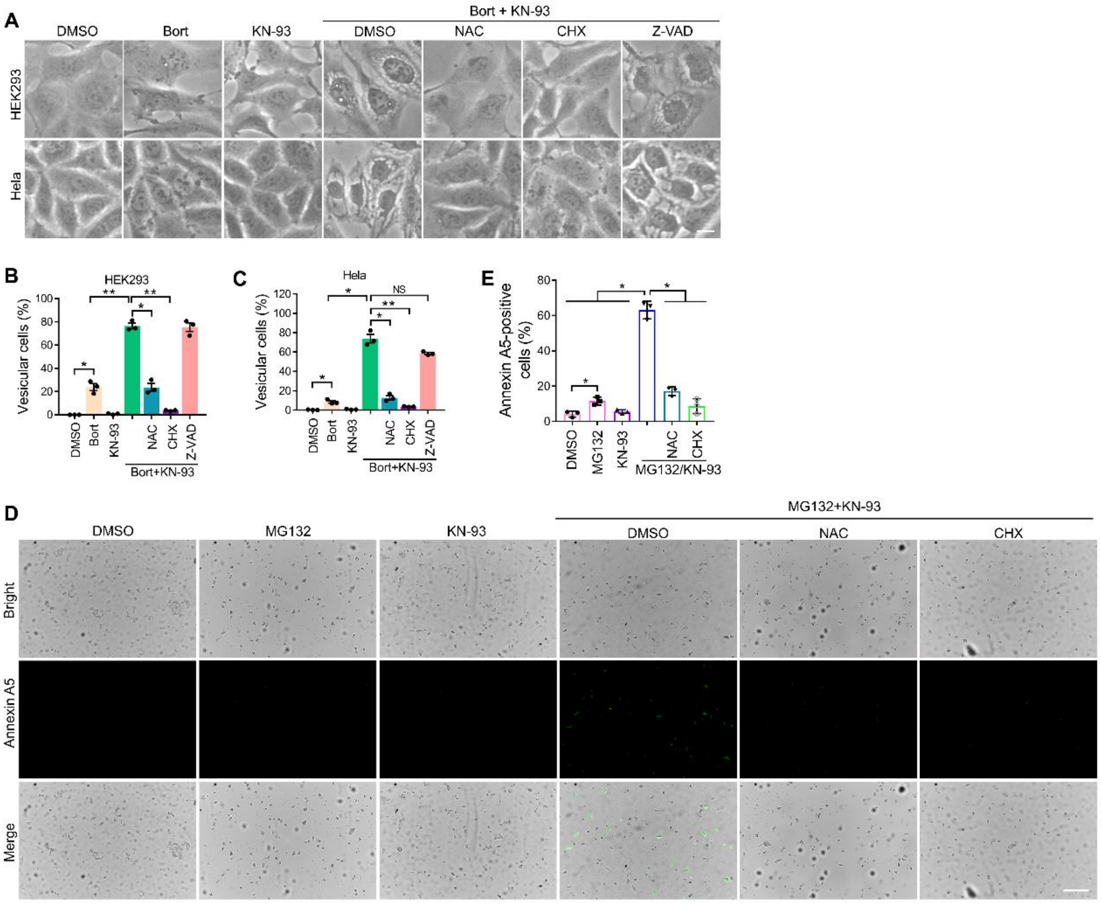

Appendix Fig. S1. Inhibition of CaMKII aggravates proteasome inhibitor-induced paraptosis, related to Figure 2.

(A) Cell morphology imaging of HEK293 and Hela cells treated with indicated drugs (Bortezomib (1 μM), KN-93 (10 μM), CHX (25μg/ml), N-Acetylcysteine (NAC, 2 mM), and Z-VAD-FMK (Z-VAD, 10 μM)) for 24 h. Scale bar: 10 μm. **(B-C)** Quantitative analysis of results in (**A**). Data are mean ± SEM of biological replicates (n = 3). At least 50 cells were randomly selected from each group to score. **(D)** Annexin 5-staining of Hela cells treated with indicated drugs (MG132 (1 μM), KN-93 (10 μM), CHX (50 μg/ml) and N-Acetylcysteine (NAC, 2 mM)) for 24 h. Scale bar: 200 μm. **(E)** Quantitative analysis of results in (**D**). Data are mean ± SEM of biological replicates (n = 3). At least 200 cells were randomly selected from each group to score. For **B**, **C**, and **E**, the P value was determined by a one-way ANOVA analysis. NS, not significant; *$P < 0.05$; **$P < 0.01$.

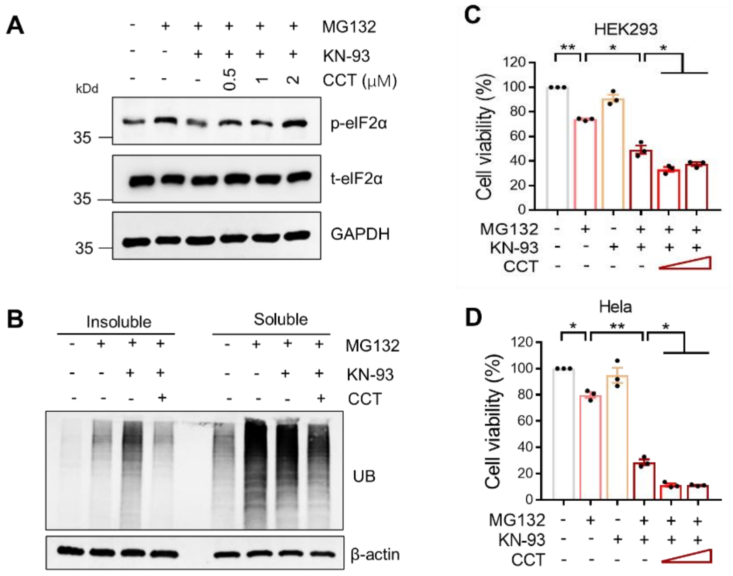

Appendix Fig. S2. Activation of PERK rescues CaMKII inhibition-induced aggregation of ubiquitinated proteins, but does not reverse cell damage.

(A) Immunoblotting of p-eIF2α (S51) in Hela cells treated with indicated drugs (MG132 (1 μM), KN-93 (10 μM), CCT (0.5, 1, 2 μM)) for 14 h.

(B) Immunoblotting of UB in NP-40-soluble and -insoluble fractions of Hela cells treated with indicated drugs MG132 (1 μM), KN-93 (10 μM), CCT (2 μM) for 14 h.

(C-D) Cell viability of HEK293 and Hela cells treated with indicated drugs (MG132 (1 μM), KN-93 (10 μM), CCT020312 (CCT, 1 μM, 2 μM)) for 48 h. The *P* value was determined by a one-way ANOVA analysis. **P* < 0.05; ***P* < 0.01.

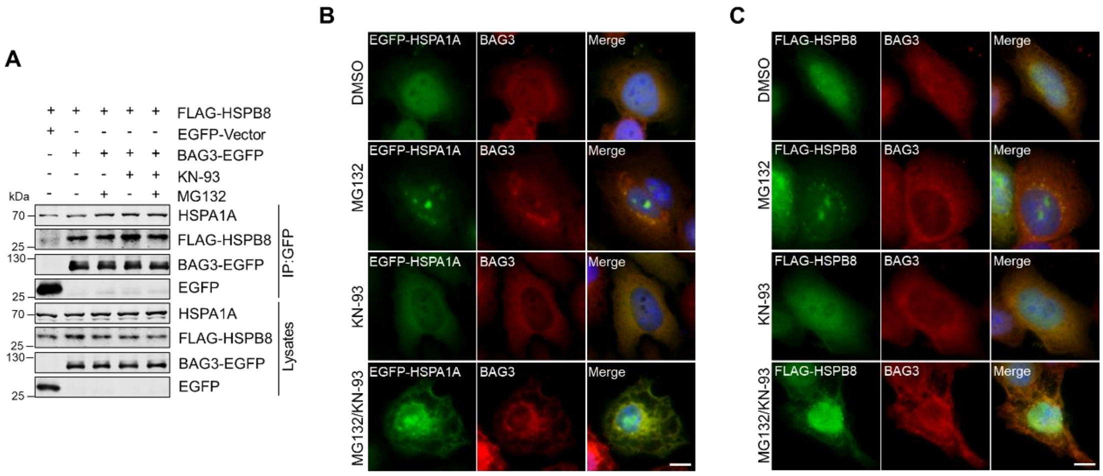

Appendix Fig. S3. Inhibition of CaMKIIs do not affect the interaction of BAG3 with HSPs, related to Figure 5

(A) Interaction between BAG3 and HSPA1A, HSPB8 in HEK293 cells transfected with indicated plasmids and treated with indicated drugs (MG132 (2 μM), KN-93 (10 μM)). Immunoprecipitation were performed using anti-GFP antibody.

(B-C) Representative BAG3 and exogenous HSPA1A or HSPB8 staining images of Hela cells. Scale bar: 10 μm.

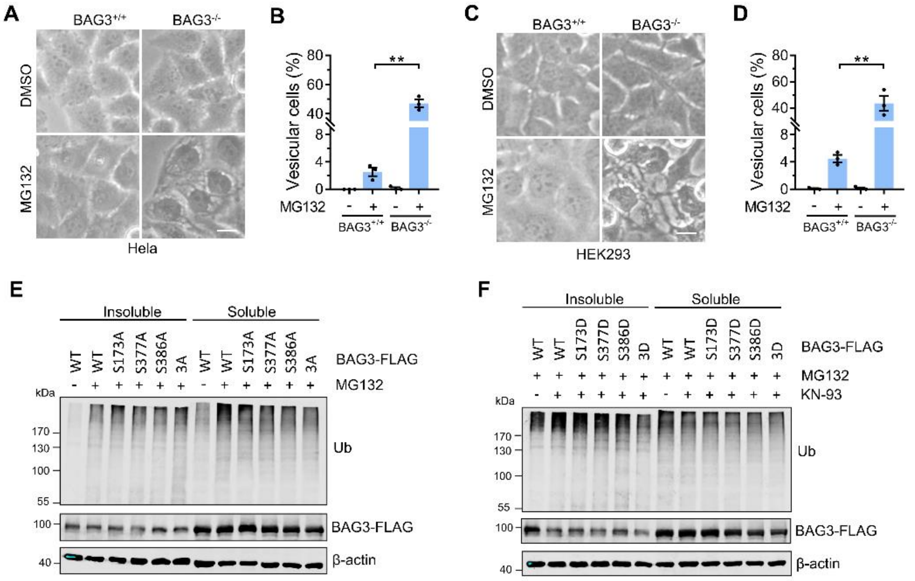

Appendix Fig. S4. Phosphorylated BAG3 regulates proteasome inhibition-induced proteotoxicity, related Figure 6.

(A) Cell morphology imaging of wildtype (BAG3$^{+/+}$) or BAG3 knockout (BAG3$^{-/-}$) Hela cells treated with indicated drugs for 24 h. Scale bar: 10 µm.

(B) Quantitative analysis of results in (**A**). Data are mean ± SEM of biological replicates (*n* = 3). At least 50 cells were randomly selected from each group to score.

(C) Cell morphology imaging of wildtype (BAG3$^{+/+}$) or BAG3 knockout (BAG3$^{-/-}$) HEK293 cells treated with indicated drugs for 24 h. Scale bar: 10 µm.

(D) Quantitative analysis of results in (**C**). Data are mean ± SEM of biological replicates (*n* = 3). At least 50 cells were randomly selected from each group to score.

(E-F) Immunoblotting of UB in NP-40-soluble and -insoluble fractions of BAG3$^{-/-}$ HEK293 cells that were transiently re-expressed WT or mutated BAG3 and then treated with indicated drugs.

For **B** and **D**, the *P* value was determined by Student's *t*-test (two-sided). $^{**}P < 0.01$.

Appendix Table S1. Sequences of the primers used for ORF amplification.

PCRed DNA Fragment		Primer Sequence (5'-3')
CaMKIIβ	Primer-f:	ctagccttaagcttggtaccggatccagccaccatggccaccacggtgacctgcac
	Primer-r:	ccttgtaatctctagactcgagctgcagcggggccacaggcgcg
CaMKIIδ	Primer-f:	ctagccttaagcttggtaccggatccagccaccatggcttcgaccacaacctgcac
	Primer-r:	ccttgtaatctctagactcgaggatgttttgccacaaagaggtgcctcc
HSPB8	Primer-f:	gatgacgataaaggatccgaattcatggctgacggtcagatgcccttc
	Primer-r:	taaacgggccctctagactcgagtcaggtacaggtgacttcctggctg
HRI	Primer-f:	ggccagaacggccgctagccttaagcttgccaccatgcagggggcaactccggggt
	Primer-r:	tccttgtaatctctagactcgagtcccacgcccccatcctttccgtcatcc
BAG3 S173A-N	Primer-f:	agccttaagcttggtaccggatccgccaccatgagcgctgcaactcattcacctatg
	Primer-r:	cagaggcagctggggcctgggaccgctc
BAG3 S173A-C	Primer-f:	gagcggtcccaggccccagctgcctctg
	Primer-r:	gtcatcatccttgtaatctctagacggtgctgctgggttaccaggggtgtctg
BAG3 S377A -N	Primer-f:	agccttaagcttggtaccggatccgccaccatgagcgctgcaactcattcacctatg
	Primer-r:	cagaagggccaggggcgggaggagg
BAG3 S377A-C	Primer-f:	cctcctcccgcccctggcccttctg
	Primer-r:	gtcatcatccttgtaatctctagacggtgctgctgggttaccaggggtgtctg
BAG3 S386A -N	Primer-f:	agccttaagcttggtaccggatccgccaccatgagcgctgcaactcattcacctatg
	Primer-r:	gtagccacactcttgggtgcagaggggacagcag
BAG3 S386A-C	Primer-f:	ctgctgtcccctctgcacccaagagtgtggctac
	Primer-r:	gtcatcatccttgtaatctctagacggtgctgctgggttaccaggggtgtctg
BAG3 S173D-N	Primer-f:	agccttaagcttggtaccggatccgccaccatgagcgctgcaactcattcacctatg
	Primer-r:	cagaggcagctgggtcctgggaccgctc
BAG3 S173D-C	Primer-f:	gagcggtcccaggacccagctgcctctg
	Primer-r:	gtcatcatccttgtaatctctagacggtgctgctgggttaccaggggtgtctg
BAG3 S377D-N	Primer-f:	agccttaagcttggtaccggatccgccaccatgagcgctgcaactcattcacctatg
	Primer-r:	cagaagggccagggtcgggaggagg
BAG3 S377D-C	Primer-f:	cctcctcccgaccctggcccttctg
	Primer-r:	gtcatcatccttgtaatctctagacggtgctgctgggttaccaggggtgtctg
BAG3 S386D-N	Primer-f:	agccttaagcttggtaccggatccgccaccatgagcgctgcaactcattcacctatg
	Primer-r:	gtagccacactcttgggatcagaggggacagcag
BAG3 S386D-C	Primer-f:	ctgctgtcccctctgatcccaagagtgtggctac
	Primer-r:	gtcatcatccttgtaatctctagacggtgctgctgggttaccaggggtgtctg
CaMKIIβ T287A-N	Primer-f:	ctagccttaagcttggtaccggatccagccaccatggccaccacggtgacctgcac
	Primer-r:	cagacactccacggcctcctgtctgtg
CaMKIIβ T287A-C	Primer-f:	cacagacaggaggccgtggagtgtctg
	Primer-r:	ccttgtaatctctagactcgagctgcagcggggccacaggcgcg
CaMKIIδ T287A-N	Primer-f:	ctagccttaagcttggtaccggatccagccaccatggcttcgaccacaacctgcac

	Primer-r:	atgcacagacaggaggctgtagactgcttgaag
CaMKIIδ T287A-C	Primer-f:	cttcaagcagtctacagcctcctgtctgtgcat
	Primer-r:	ccttgtaatctctagactcgaggatgttttgccacaaagaggtgcctcc
CaMKIIβ K43M-N	Primer-f:	ctagccttaagcttggtaccggatccagccaccatggccaccacggtgacctgcac
	Primer-r:	ggtgttgatgatcatggctgcatactc
CaMKIIβ K43M-C	Primer-f:	gagtatgcagccatgatcatcaacacc
	Primer-r:	ccttgtaatctctagactcgagctgcagcggggccacaggcgcg
CaMKIIβ D-S	Primer-f:	ctagccttaagcttggtaccggatccagccaccatggccaccacggtgacctgcac
	Primer-r:	ccttgtaatctctagactcgagctttttcagacactccacgtcctcctgtctg
CaMKIIδ D-S	Primer-f:	ctagccttaagcttggtaccggatccagccaccatggcttcgaccacaacctgcac
	Primer-r:	ccttgtaatctctagactcgagtttcttcaagcagtctacgtcctcctgtctgtgc

Appendix Table S2. Sequences of shRNA and siRNA.

Name		sequence（5‘——3’）
shCAMK2A	forward:	CCGGCACCACTACCTTATCTTCGATCTCGAGATCGAAGATAAGGTAGTGGTGTTTTTG
	reverse:	AATTCAAAAACACCACTACCTTATCTTCGATCTCGAGATCGAAGATAAGGTAGTGGTG
shCAMK2B	forward:	CCGGATGAGTATGCAGCTAAGATCACTCGAGTGATCTTAGCTGCATACTCATTTTTTG
	reverse:	AATTCAAAAAATGAGTATGCAGCTAAGATCACTCGAGTGATCTTAGCTGCATACTCAT
shCAMK2G-1#	forward:	CCGGGGATATGTCGACTTCTGAAACCTCGAGGTTTCAGAAGTCGACATATCCTTTTTG
	reverse:	AATTCAAAAAGGATATGTCGACTTCTGAAACCTCGAGGTTTCAGAAGTCGACATATCC
shCAMK2G-2#	forward:	CCGGGGAGCCTATGATTTCCCATCACTCGAGTGATGGGAAATCATAGGCTCCTTTTTG
	reverse:	AATTCAAAAAGGAGCCTATGATTTCCCATCACTCGAGTGATGGGAAATCATAGGCTCC
shCAMK2D-1#	forward:	CCGGAACTATGCTGGCTACGAGAAACTCGAGTTTCTCGTAGCCAGCATAGTTTTTTTG
	reverse:	AATTCAAAAAAACTATGCTGGCTACGAGAAACTCGAGTTTCTCGTAGCCAGCATAGTT
shCAMK2D-2#	forward:	CCGGAAGACATAGTGGCAAGAGAATCTCGAGATTCTCTTGCCACTATGTCTTTTTTTG
	reverse:	AATTCAAAAAAAGACATAGTGGCAAGAGAATCTCGAGATTCTCTTGCCACTATGTCTT
siPEKR		GUGGAAAGGUGAGGUAUAUdTdT
siPKR		CCACAUGAUAGGAGGUUUAdTdT
siGCN2		GUGGAUUUGAGGGUUAAAUdTdT
siHRI		GCAGCGAUCUGAUGAAUUGdTdT

Appendix Table S3. Sequences of qRT-PCR Primer

Target mRNA	primer direction	primer sequence (5'-3')
CAMK2A	forward	TGGAATCCTCAGAGAGCACC
	reverse	CGCACATCTTCGTGTAGGACT
CAMK2B	forward	GCACACCAGGCTACCTGTC
	reverse	GGACGGGAAGTCATAGGCA
CAMK2G	forward	GTGGAGTGTTTGCGCAAGTT
	reverse	TGACACCGCCATCCGACT
CAMK2D	forward	GGAATTTCTCAGCAGCCAAG
	reverse	GCTTTCGTGCTTTCACATCT
GAPDH	forward	TCGTGGAAGGACTCATGACCA
	reverse	AGGCAGGGATGATGTTCTGGA
