# Supplementary figures and images for: CaMKII suppresses proteotoxicity by phosphorylating BAG3 in response to proteasomal dysfunction (part 1 of 2)
Source: EMBO Rep. 2024 Sep 11;25(10):4488–514. doi: 10.1038/s44319-024-00248-w (PMC11466968; doi:10.1038/s44319-024-00248-w)

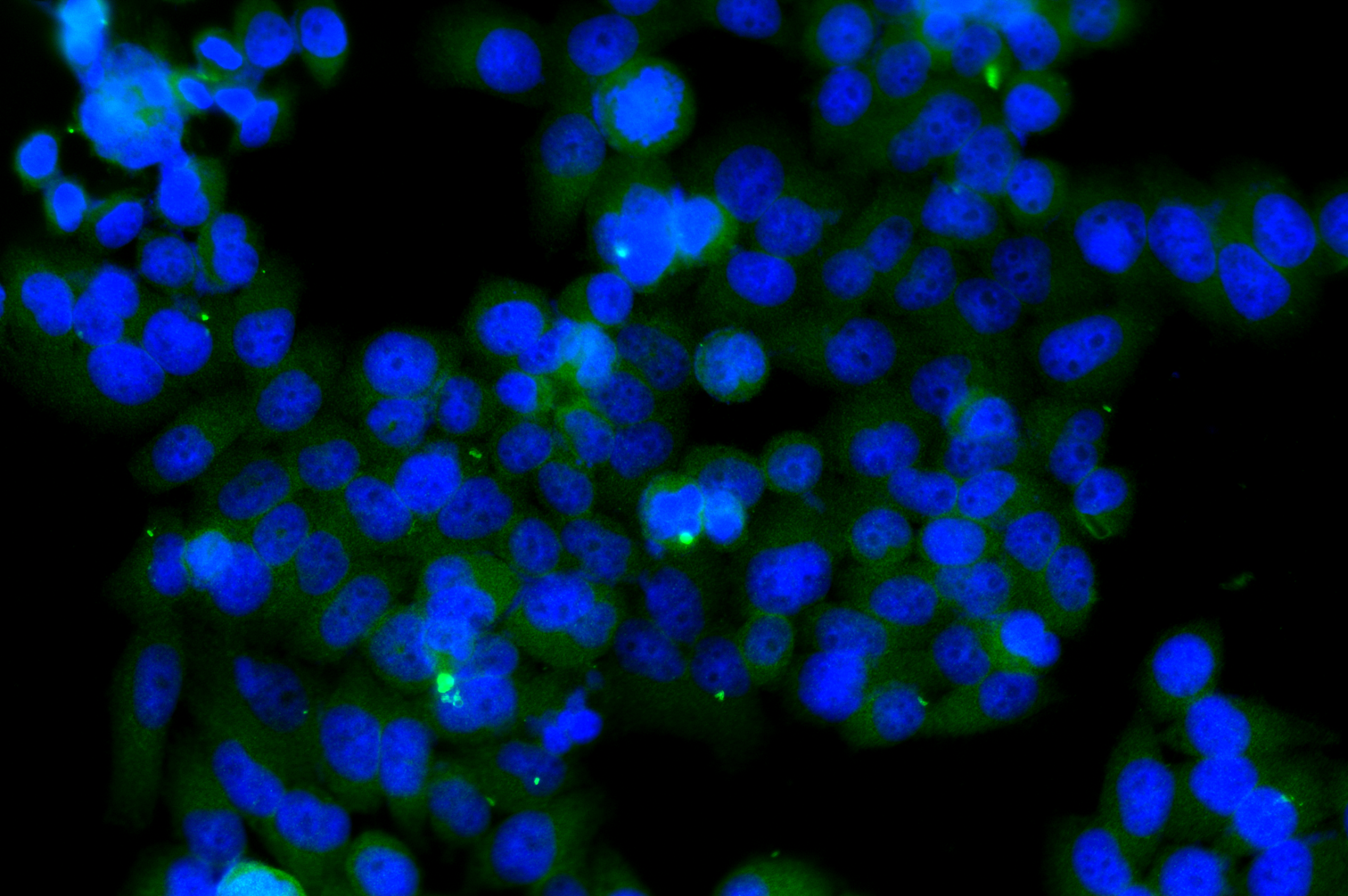

Supplement: Supplementary file 3 — Source data Fig. 1 [file 44319_2024_248_MOESM3_ESM.zip › Figure 1/Fig. 1C/CaMKIIs-DMSO (with DAPI).tif]

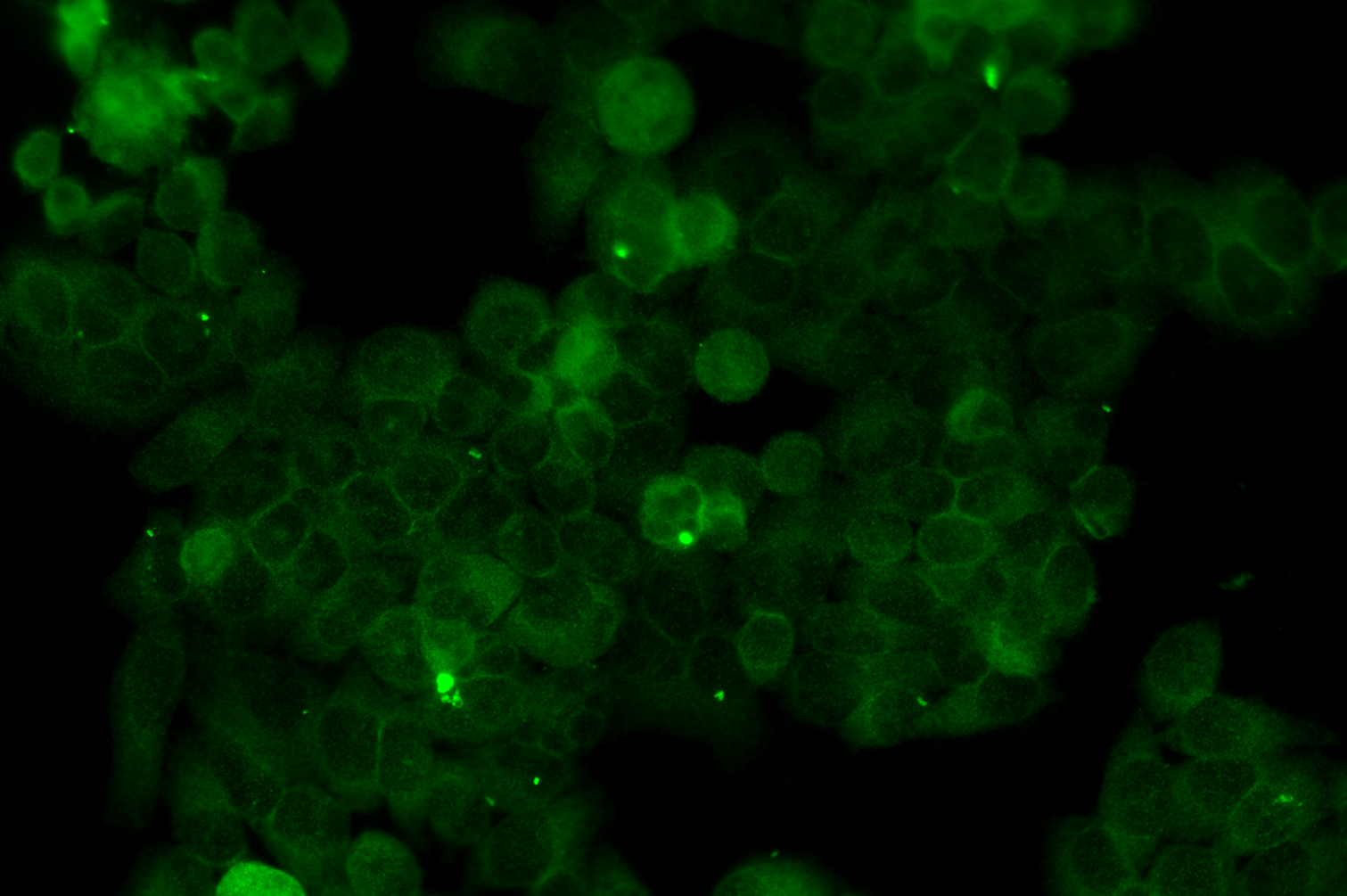

Supplement: Supplementary file 3 — Source data Fig. 1 [file 44319_2024_248_MOESM3_ESM.zip › Figure 1/Fig. 1C/CaMKIIs-DMSO (without DAPI).tif]

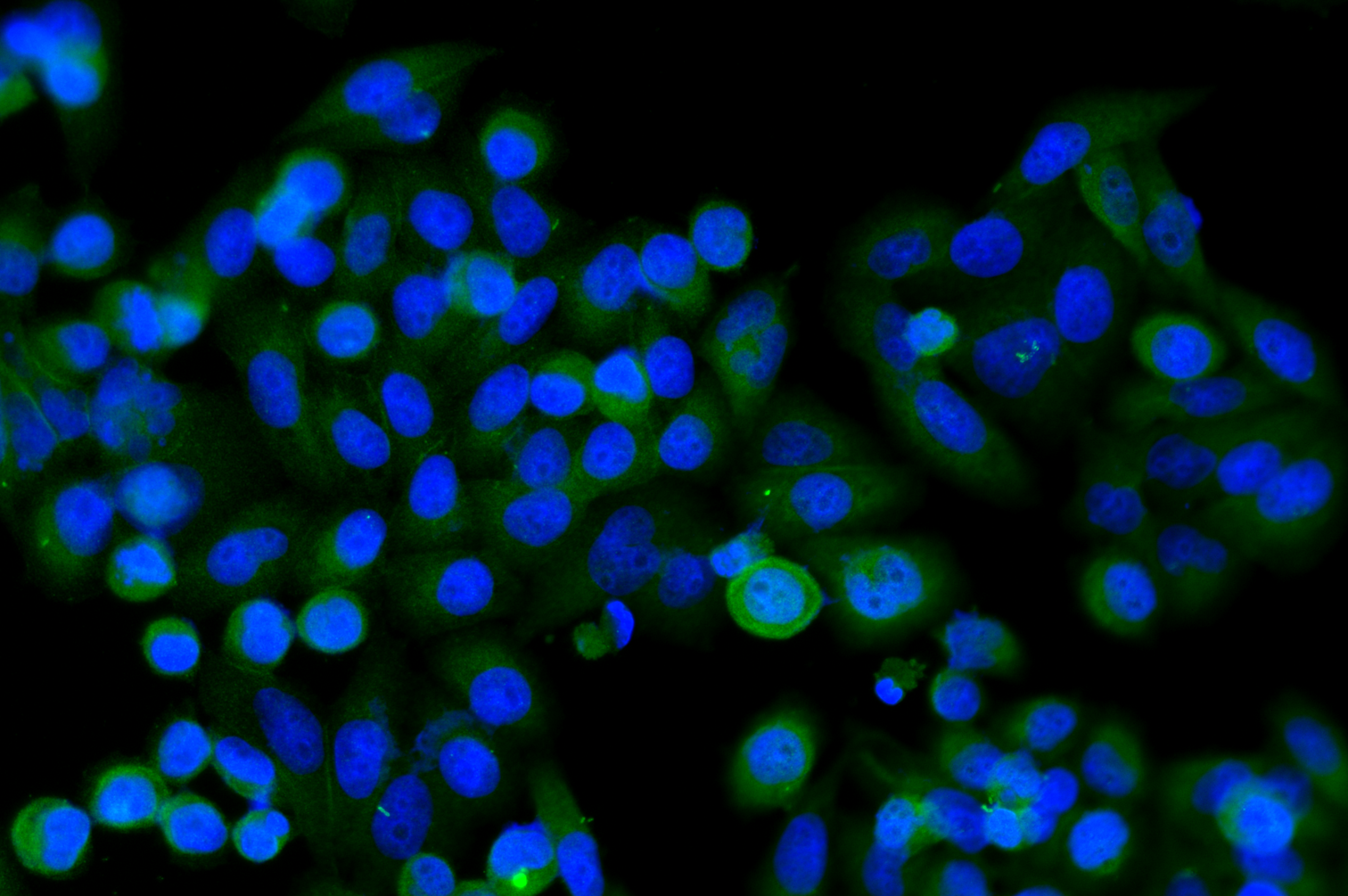

Supplement: Supplementary file 3 — Source data Fig. 1 [file 44319_2024_248_MOESM3_ESM.zip › Figure 1/Fig. 1C/CaMKIIs-MG132 (with DAPI).tif]

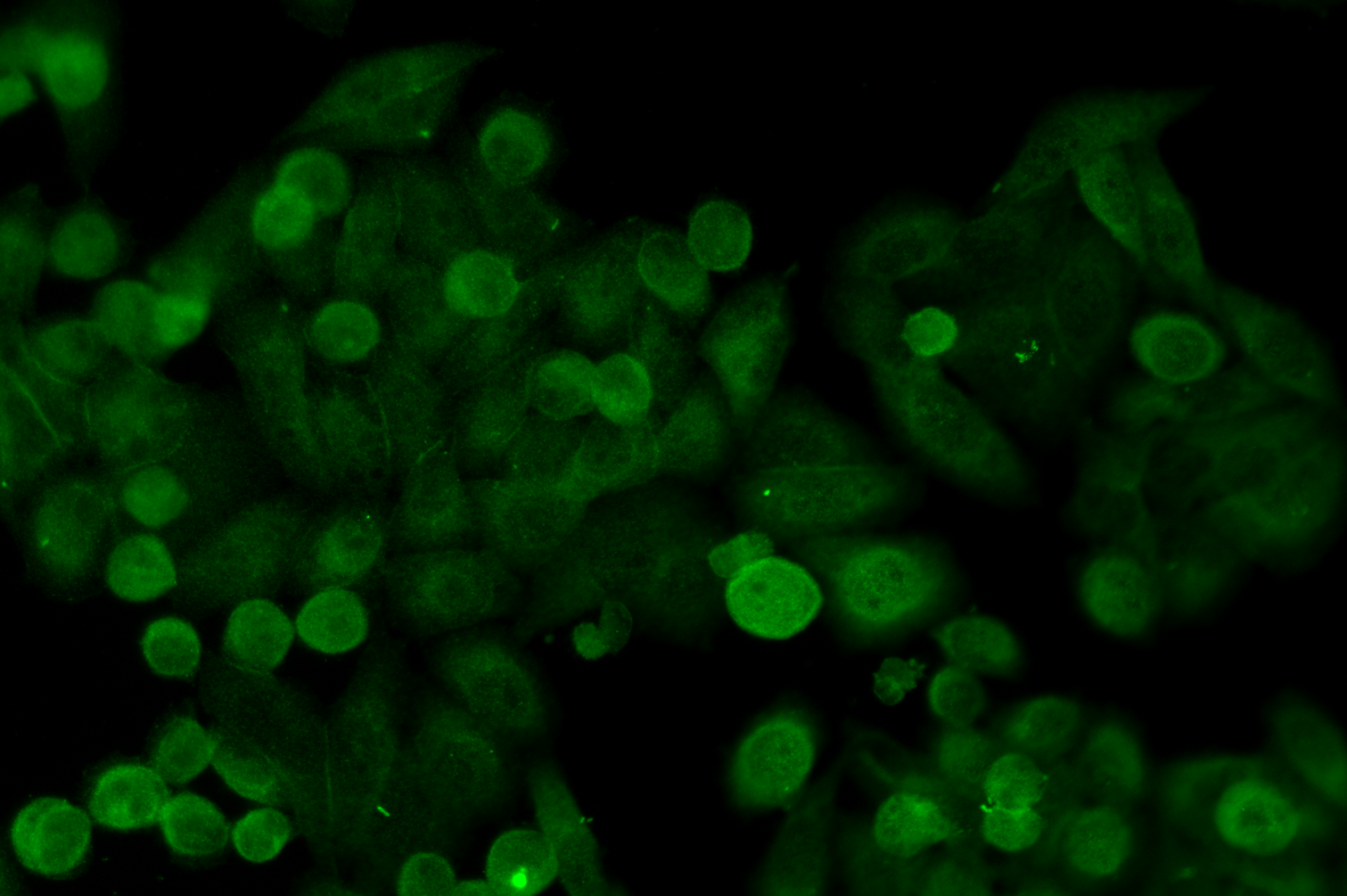

Supplement: Supplementary file 3 — Source data Fig. 1 [file 44319_2024_248_MOESM3_ESM.zip › Figure 1/Fig. 1C/CaMKIIs-MG132 (without DAPI).tif]

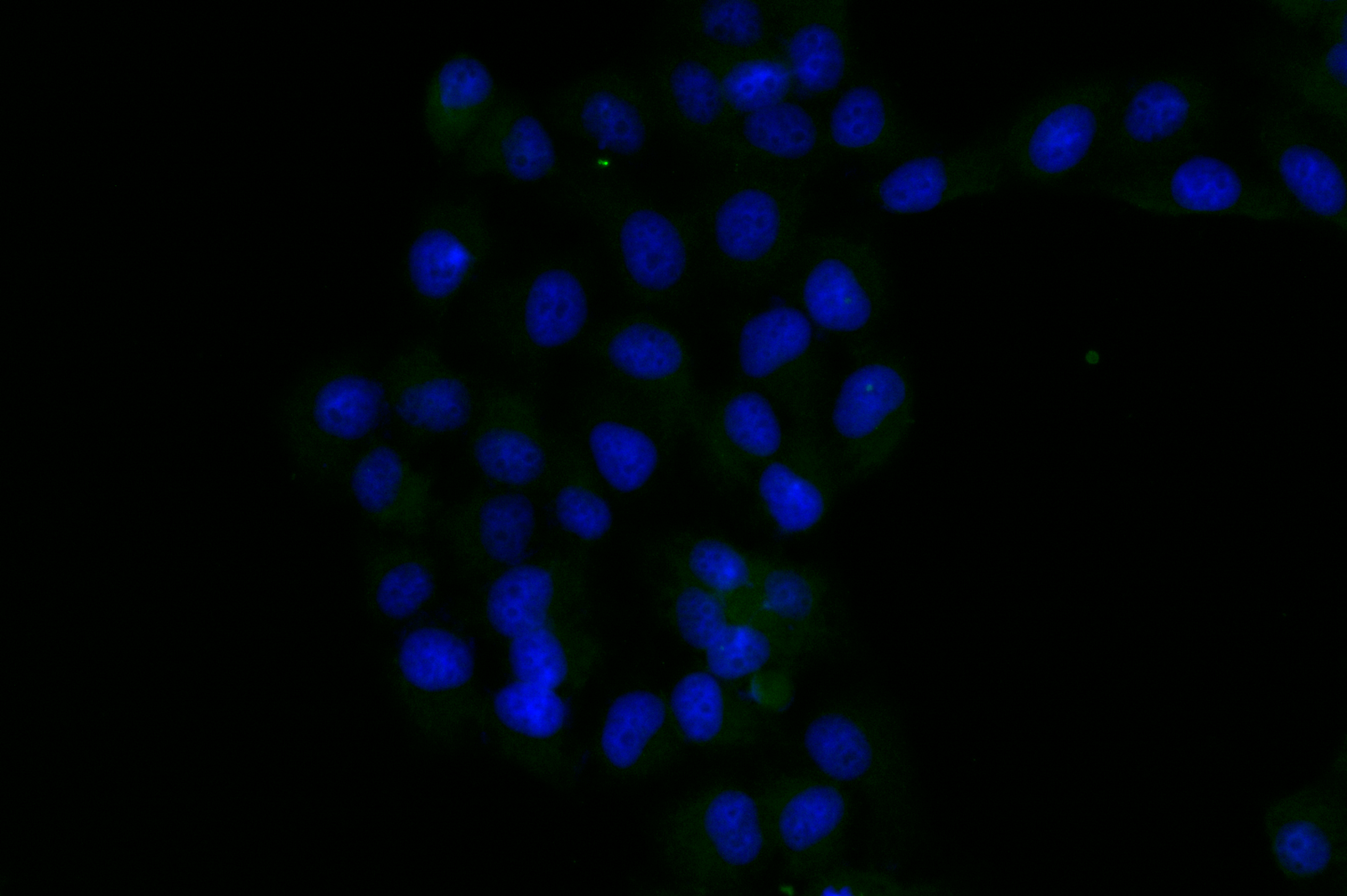

Supplement: Supplementary file 3 — Source data Fig. 1 [file 44319_2024_248_MOESM3_ESM.zip › Figure 1/Fig. 1C/p-T286 DMSO (with DAPI).tif]

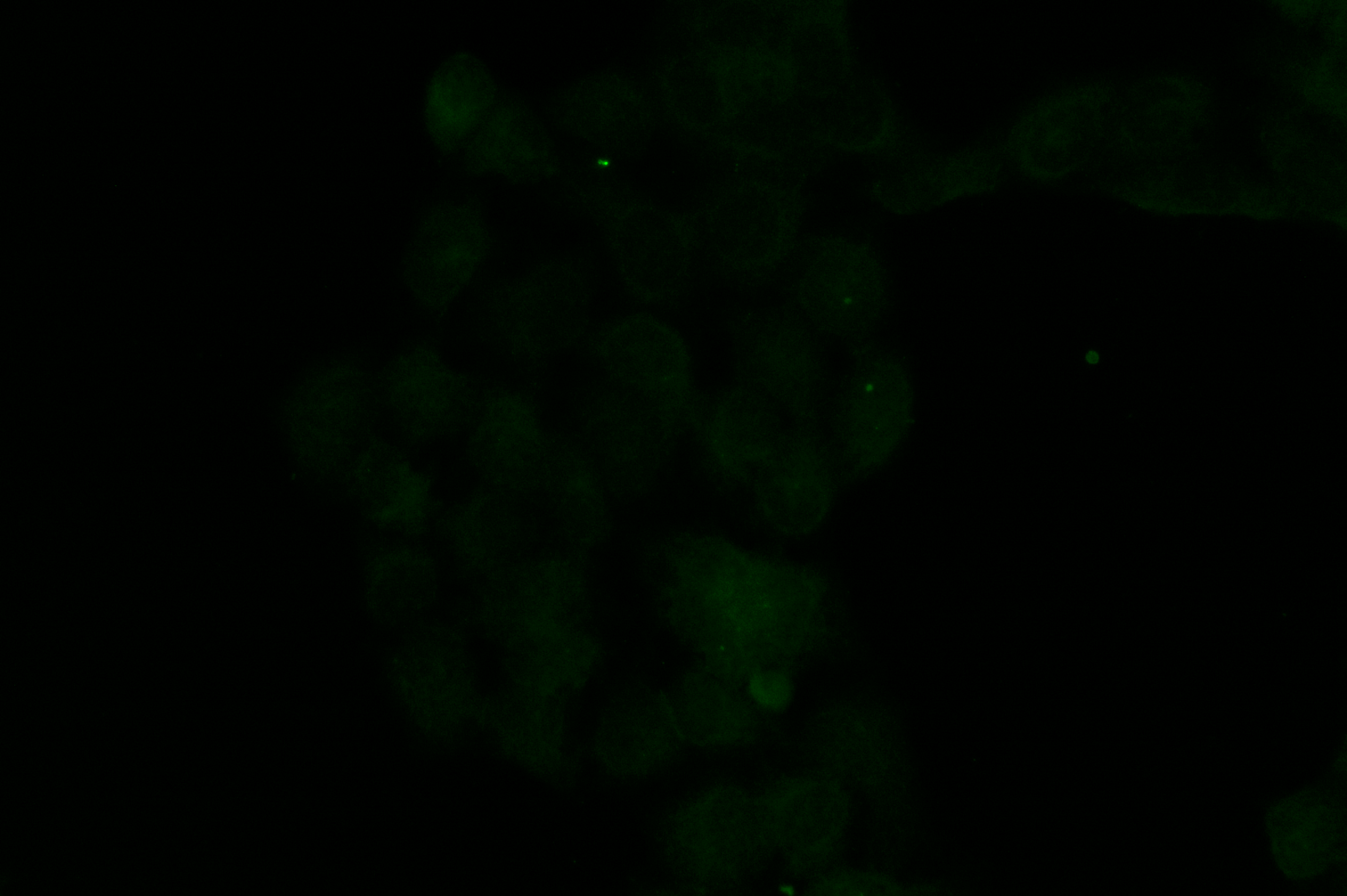

Supplement: Supplementary file 3 — Source data Fig. 1 [file 44319_2024_248_MOESM3_ESM.zip › Figure 1/Fig. 1C/p-T286 DMSO (without DAPI).tif]

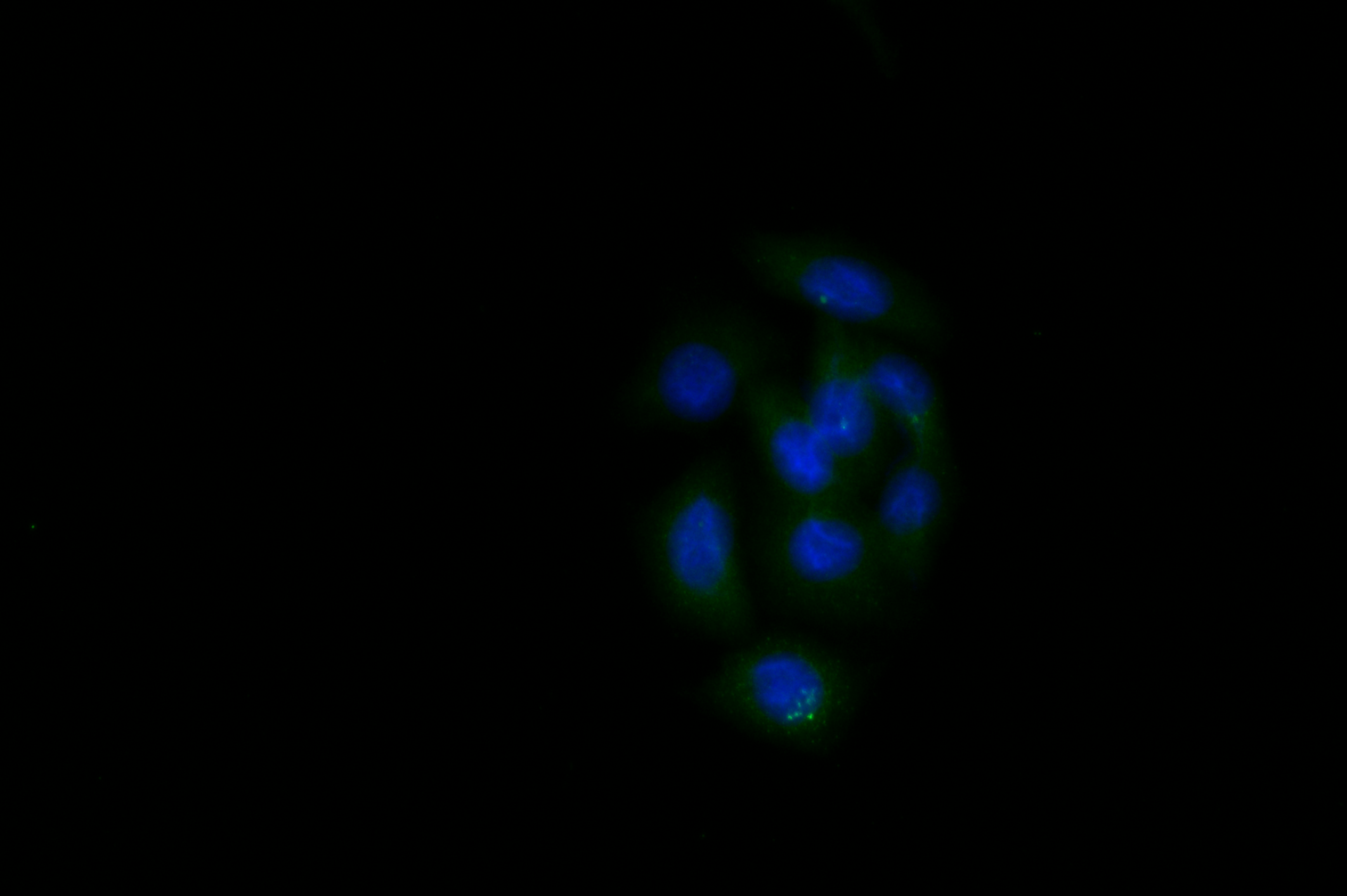

Supplement: Supplementary file 3 — Source data Fig. 1 [file 44319_2024_248_MOESM3_ESM.zip › Figure 1/Fig. 1C/p-T286 MG132 (with DAPI).tif]

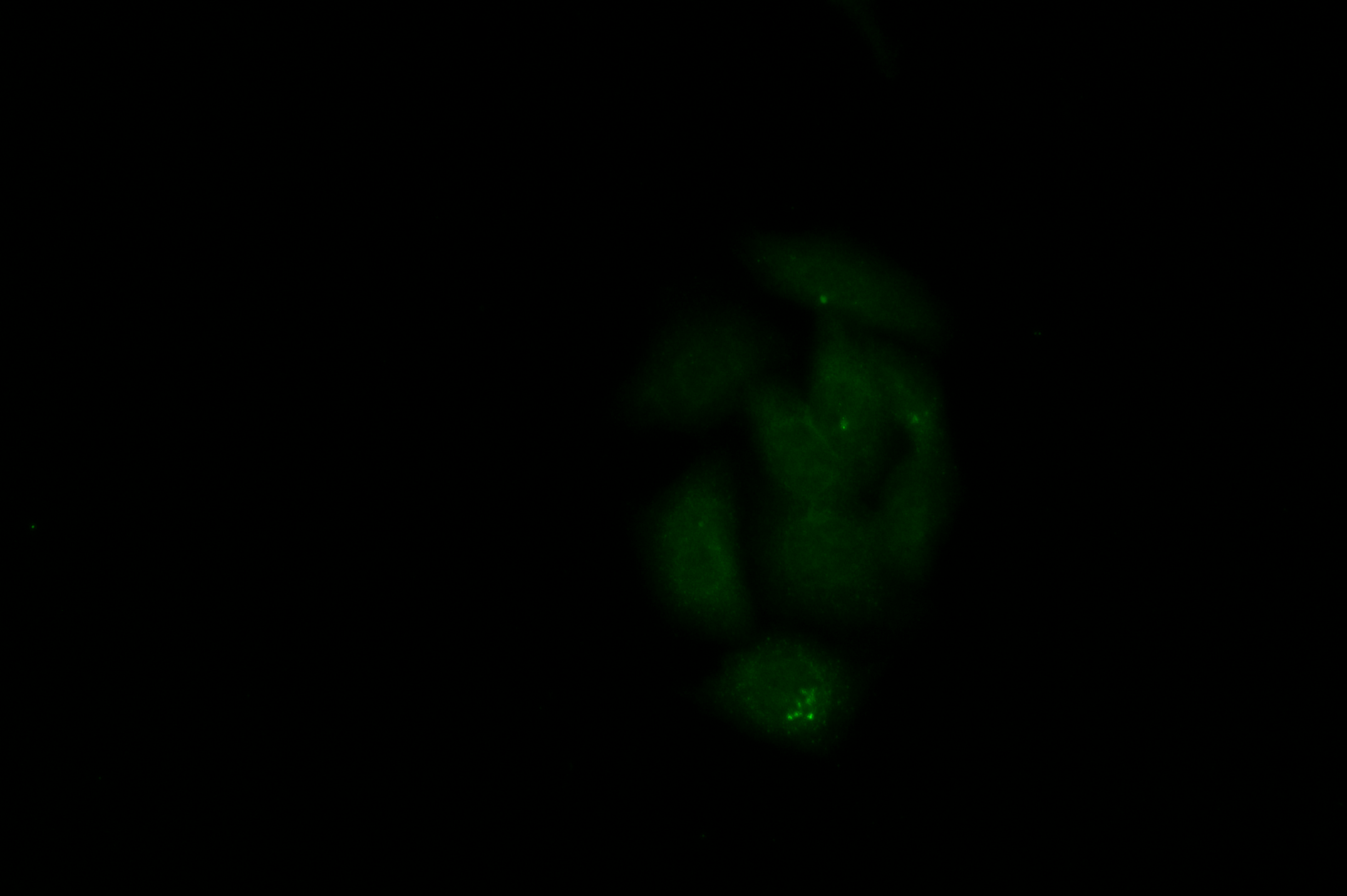

Supplement: Supplementary file 3 — Source data Fig. 1 [file 44319_2024_248_MOESM3_ESM.zip › Figure 1/Fig. 1C/p-T286 MG132 (without DAPI).tif]

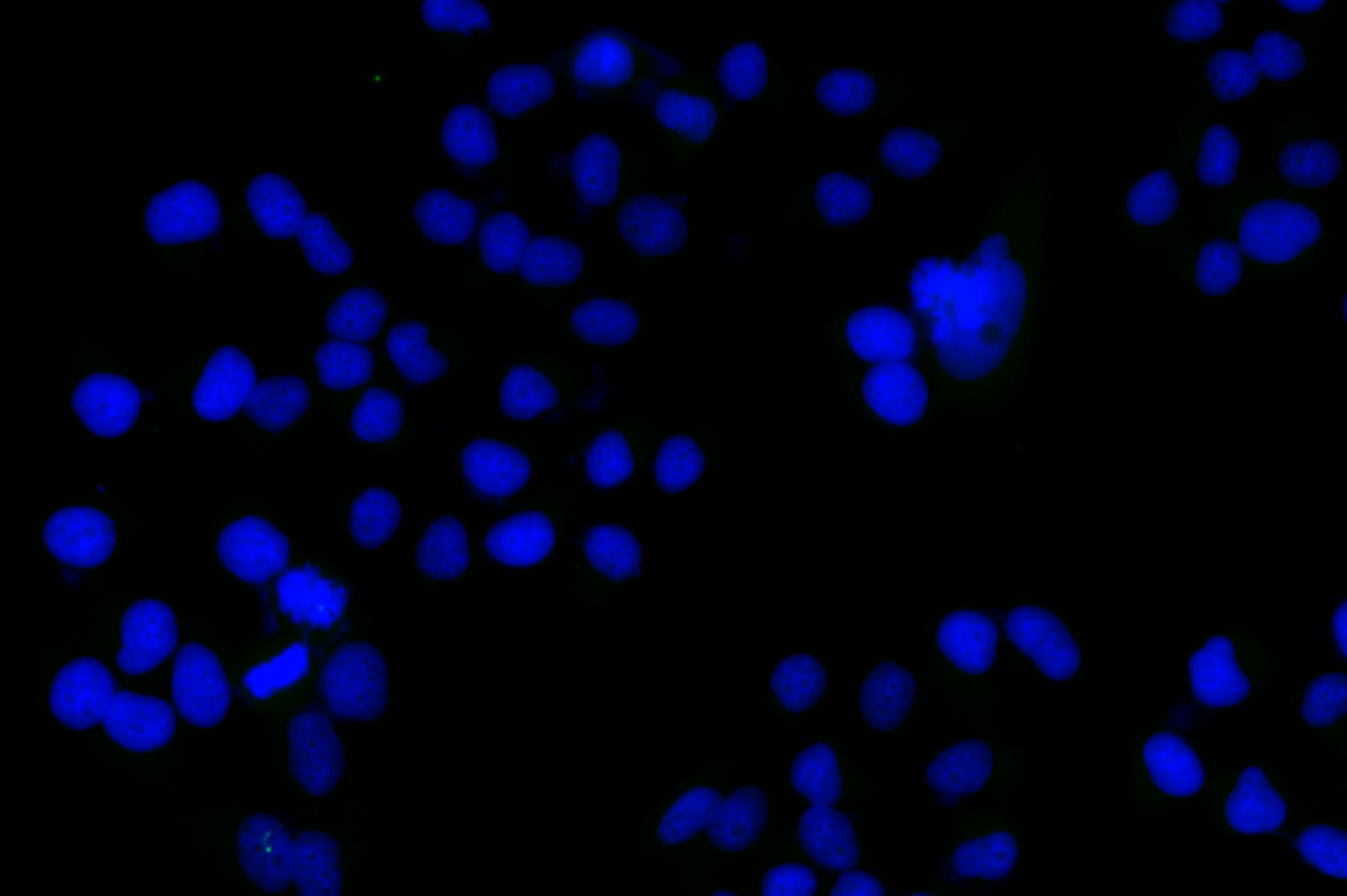

Supplement: Supplementary file 3 — Source data Fig. 1 [file 44319_2024_248_MOESM3_ESM.zip › Figure 1/Fig. 1C/p-T287 DMSO (with DAPI).tif]

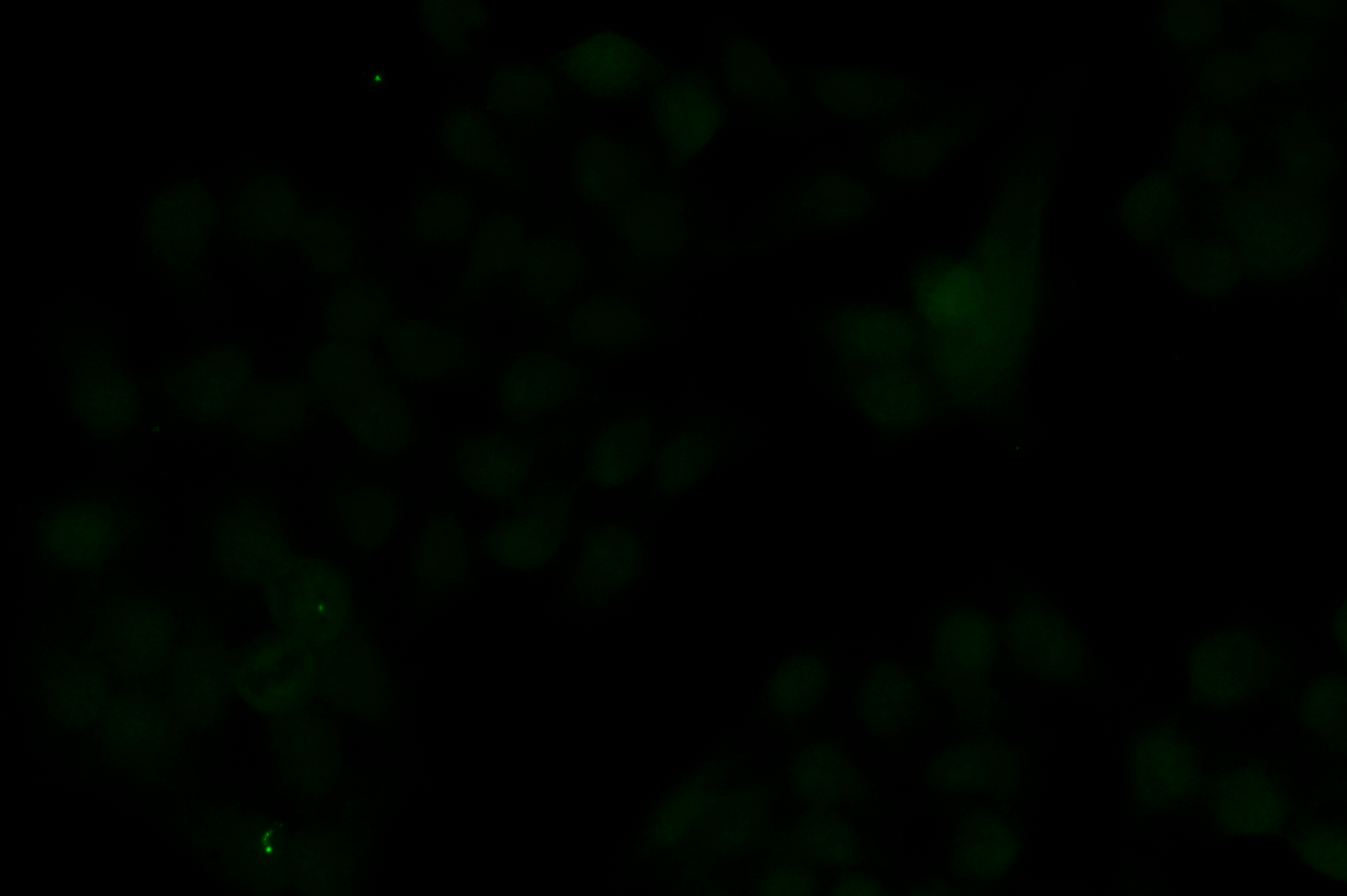

Supplement: Supplementary file 3 — Source data Fig. 1 [file 44319_2024_248_MOESM3_ESM.zip › Figure 1/Fig. 1C/p-T287 DMSO (without DAPI).tif]

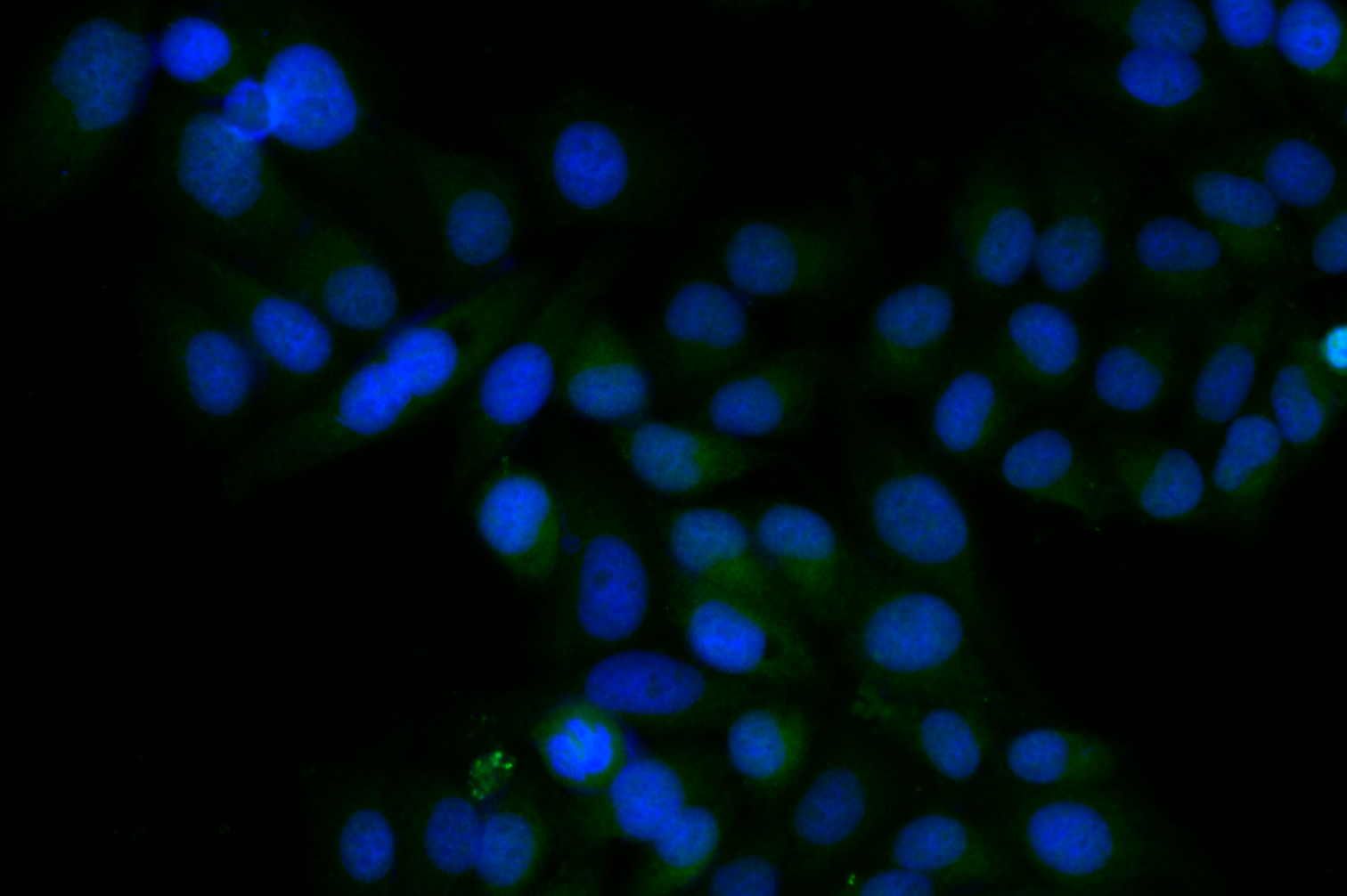

Supplement: Supplementary file 3 — Source data Fig. 1 [file 44319_2024_248_MOESM3_ESM.zip › Figure 1/Fig. 1C/p-T287 MG132 (with DAPI).tif]

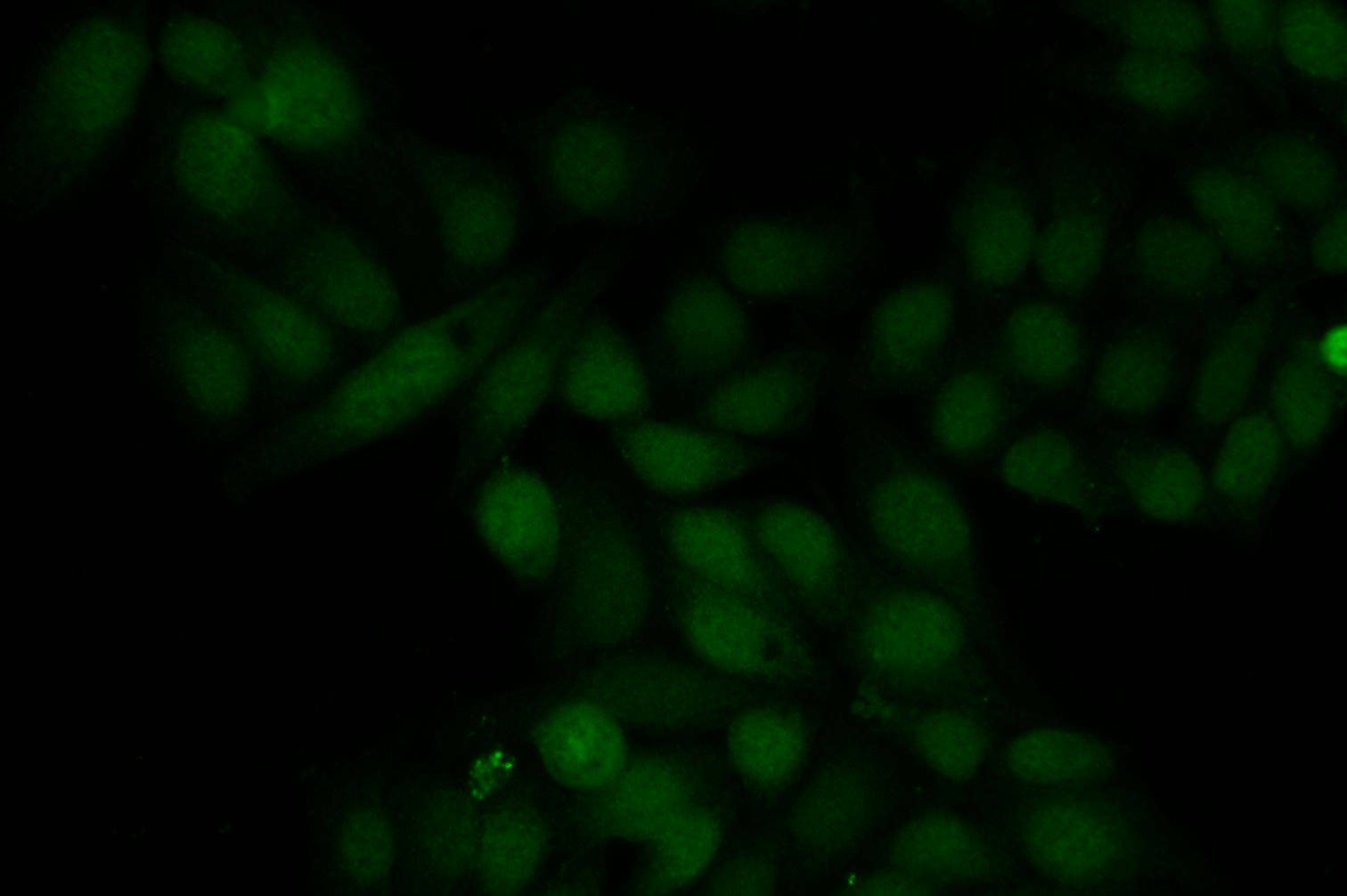

Supplement: Supplementary file 3 — Source data Fig. 1 [file 44319_2024_248_MOESM3_ESM.zip › Figure 1/Fig. 1C/p-T287 MG132 (without DAPI).tif]

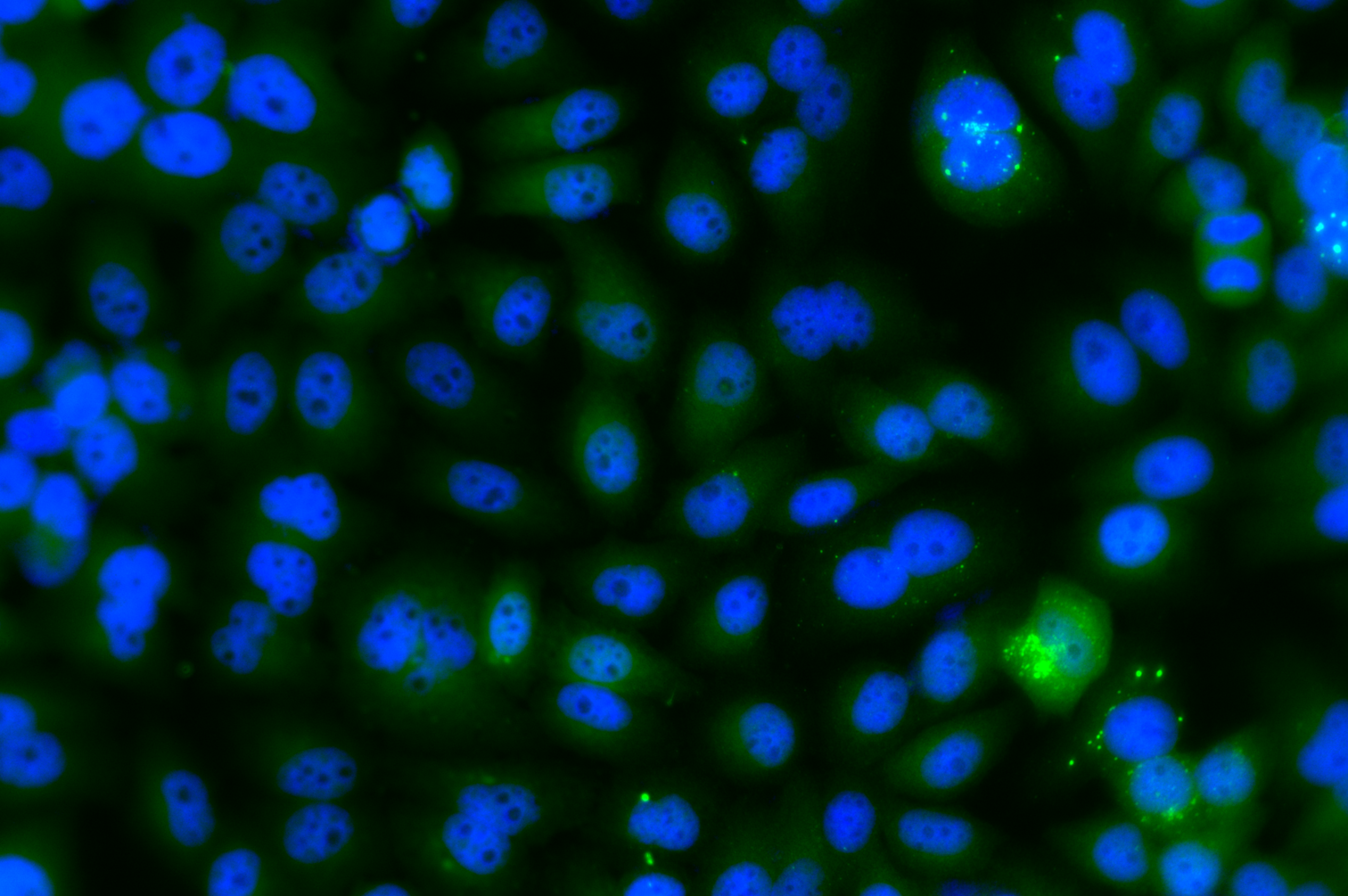

Supplement: Supplementary file 3 — Source data Fig. 1 [file 44319_2024_248_MOESM3_ESM.zip › Figure 1/Fig. 1F/shCaMKIIs-DMSO.tif]

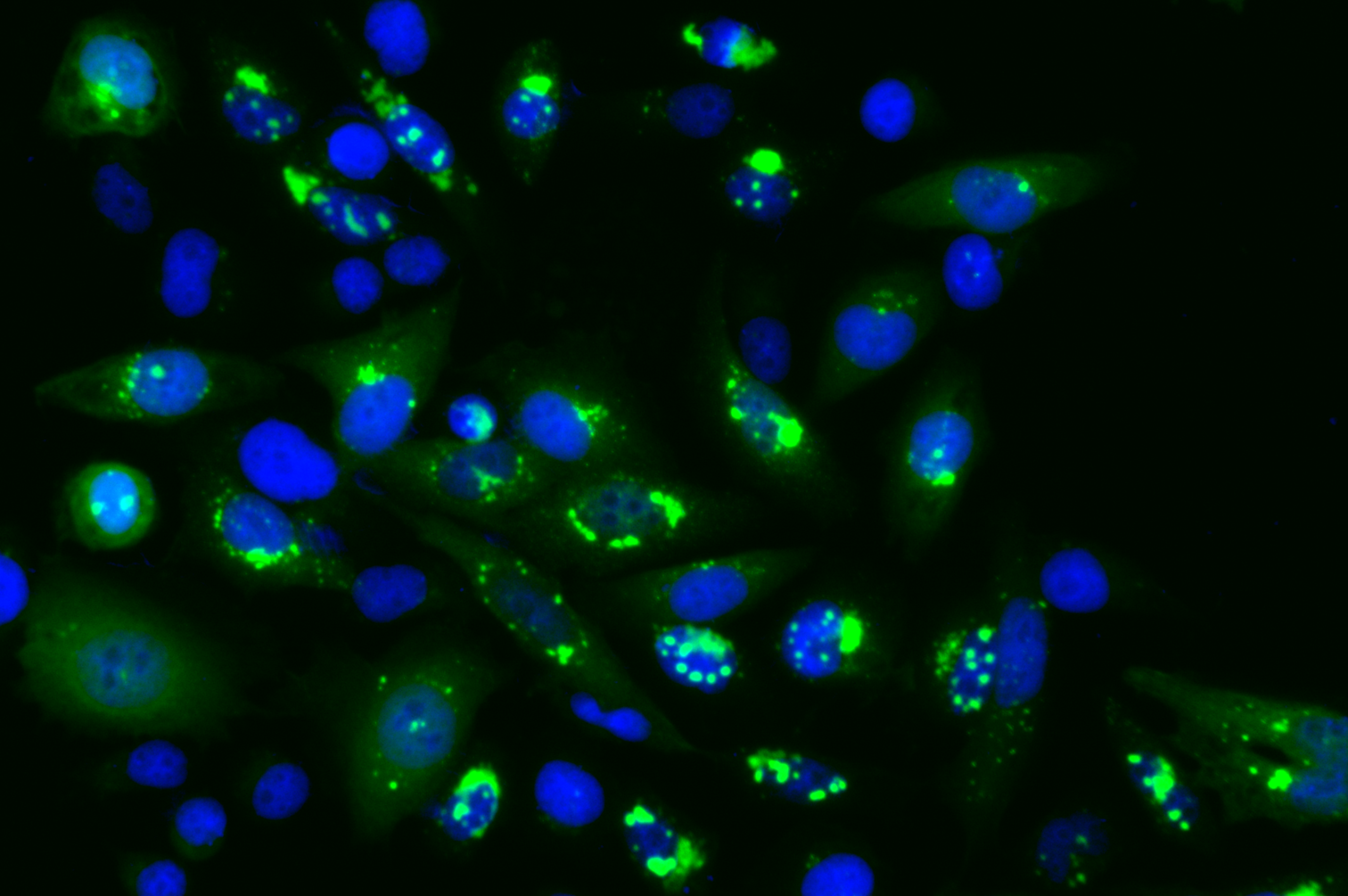

Supplement: Supplementary file 3 — Source data Fig. 1 [file 44319_2024_248_MOESM3_ESM.zip › Figure 1/Fig. 1F/shCaMKIIs-MG132.tif]

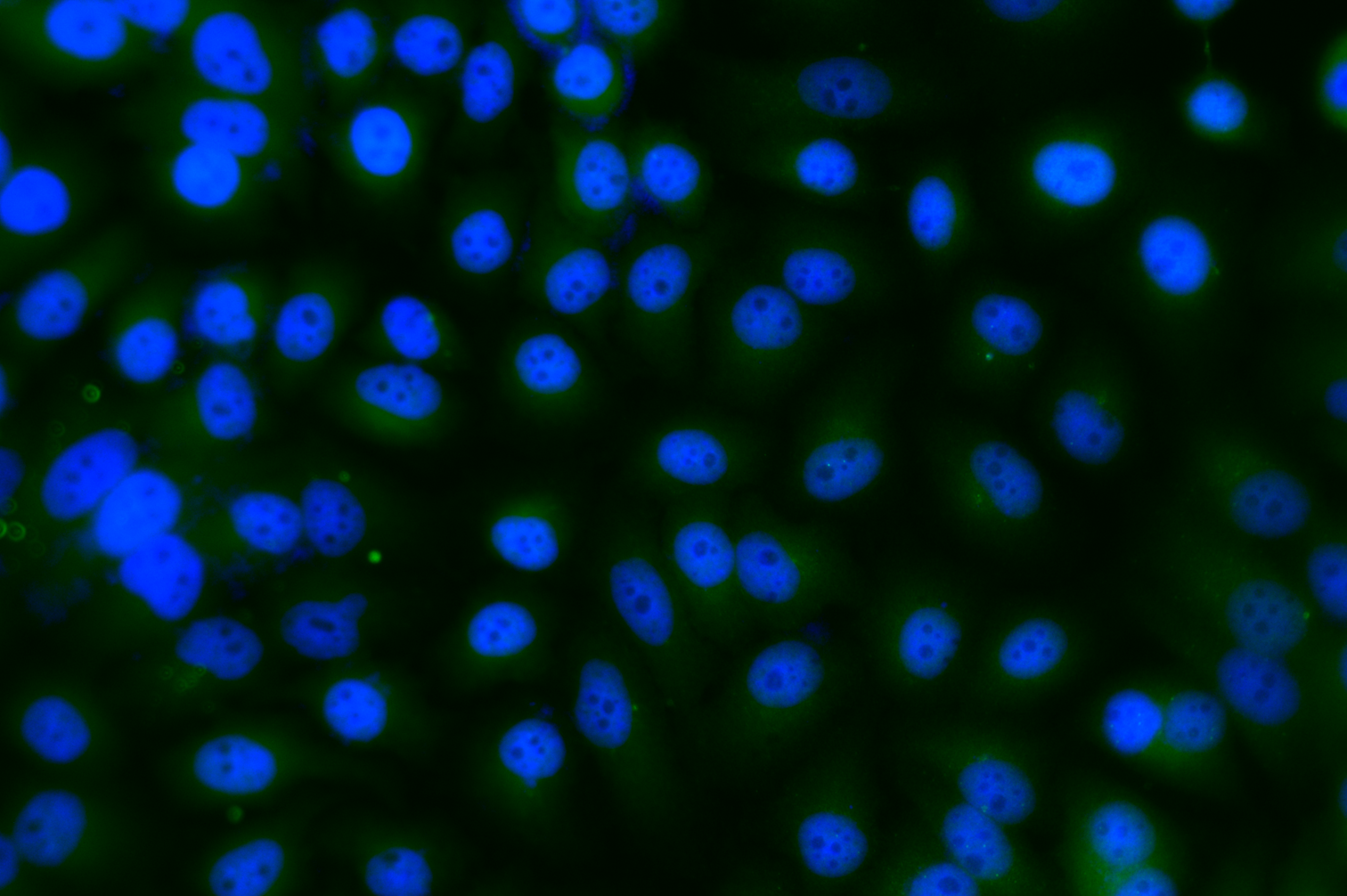

Supplement: Supplementary file 3 — Source data Fig. 1 [file 44319_2024_248_MOESM3_ESM.zip › Figure 1/Fig. 1F/shScr-DMSO.tif]

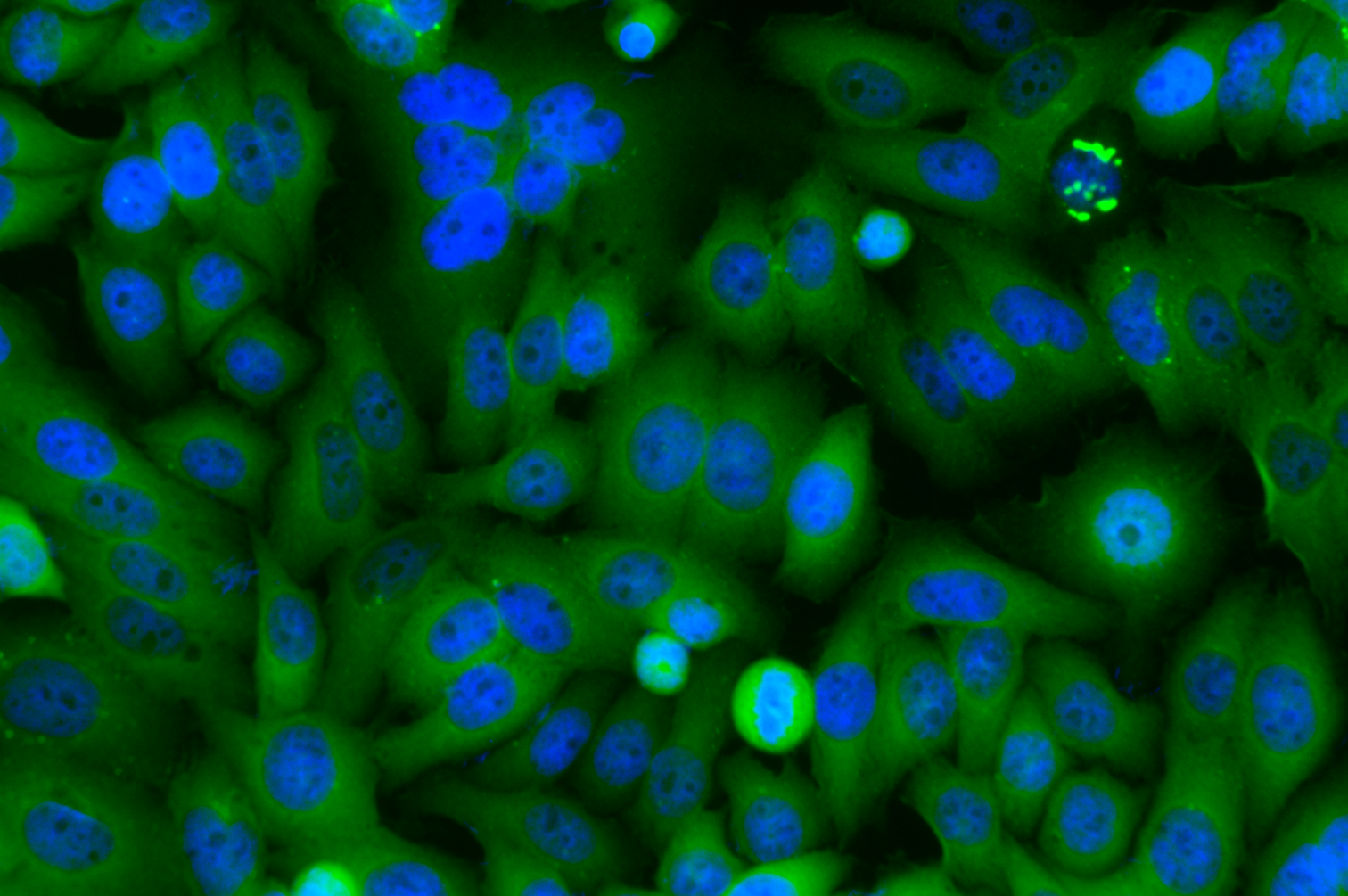

Supplement: Supplementary file 3 — Source data Fig. 1 [file 44319_2024_248_MOESM3_ESM.zip › Figure 1/Fig. 1F/shScr-MG132.tif]

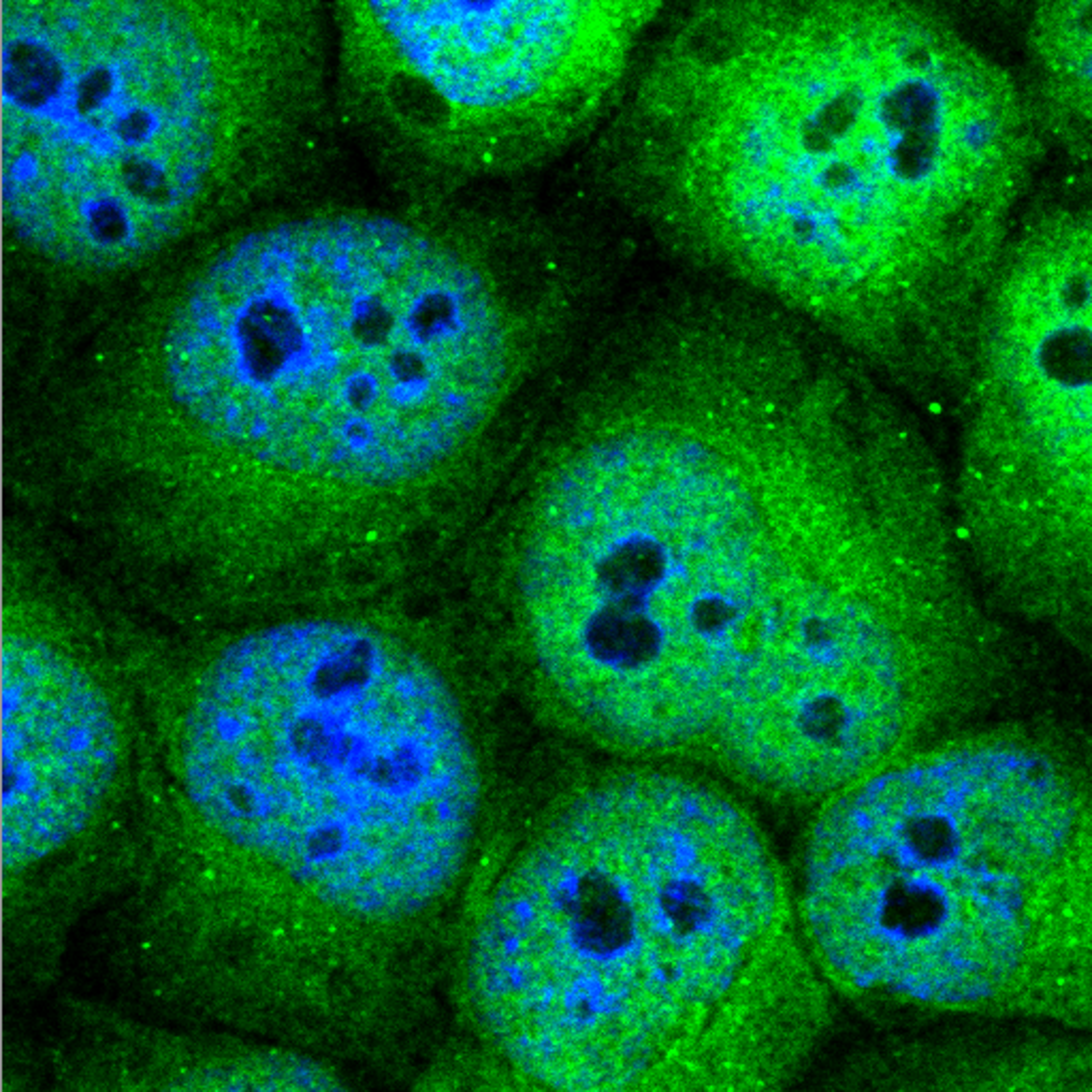

Supplement: Supplementary file 3 — Source data Fig. 1 [file 44319_2024_248_MOESM3_ESM.zip › Figure 1/Fig. 1I/DMSO.tif]

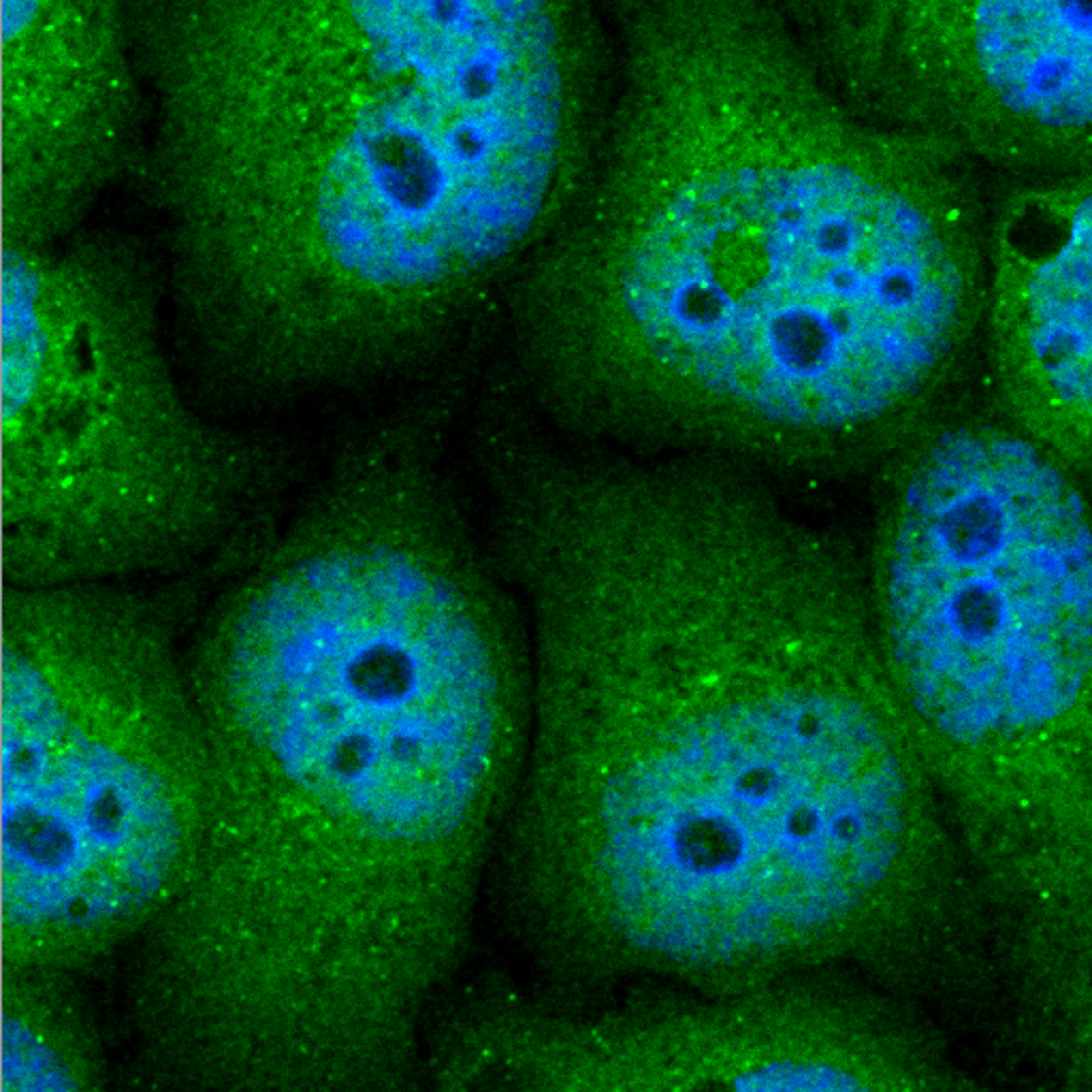

Supplement: Supplementary file 3 — Source data Fig. 1 [file 44319_2024_248_MOESM3_ESM.zip › Figure 1/Fig. 1I/KN-93.tif]

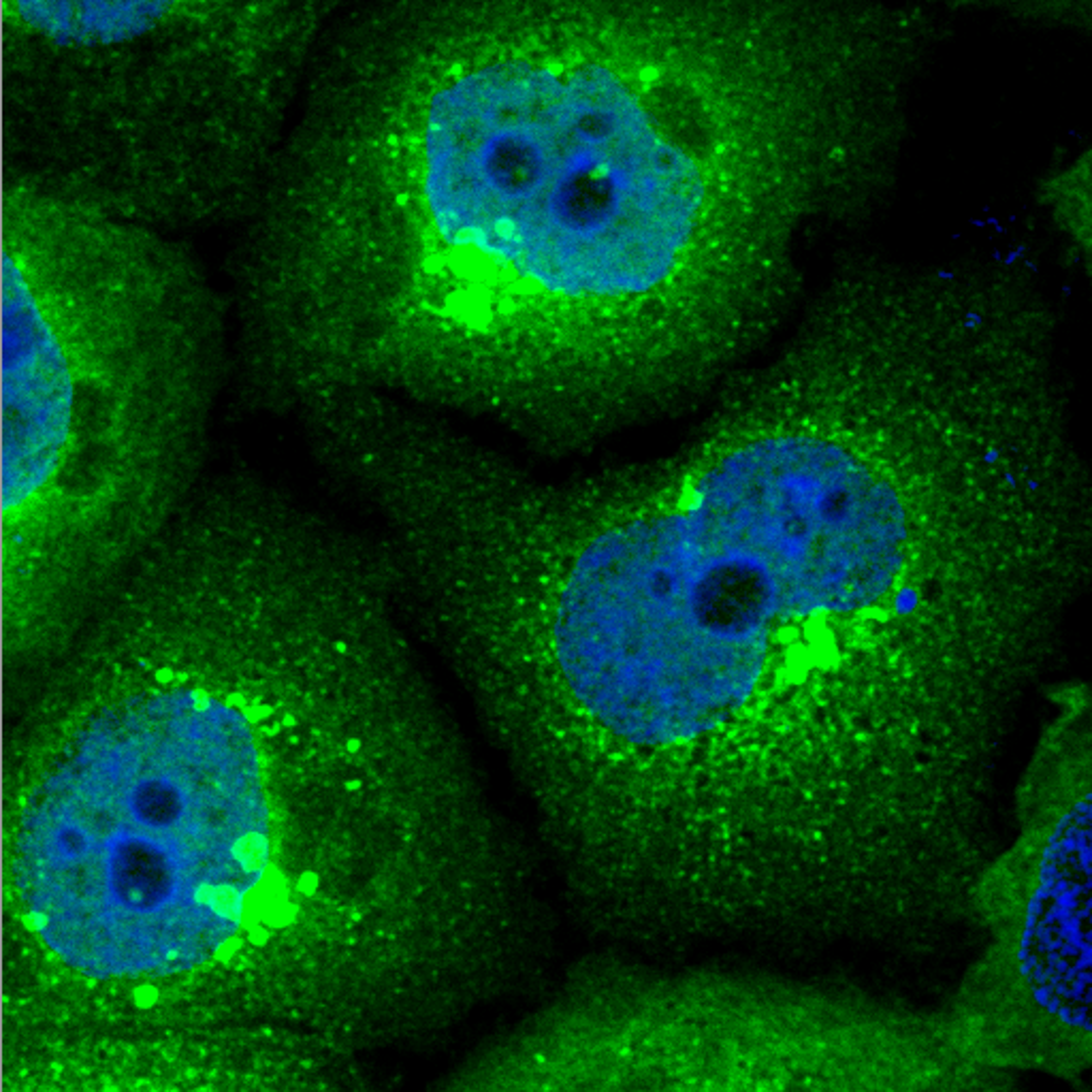

Supplement: Supplementary file 3 — Source data Fig. 1 [file 44319_2024_248_MOESM3_ESM.zip › Figure 1/Fig. 1I/MG132 0.5uM + KN-93.tif]

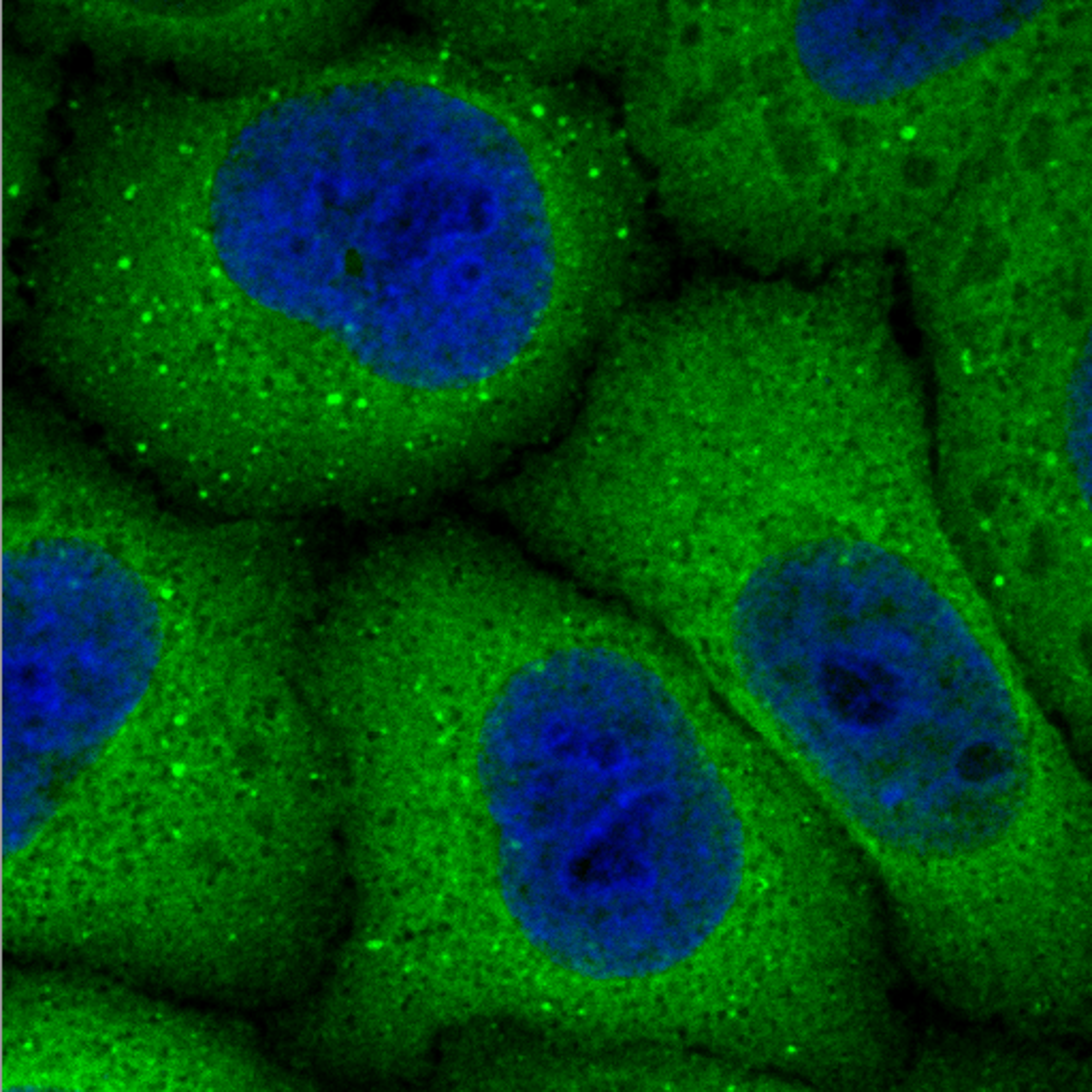

Supplement: Supplementary file 3 — Source data Fig. 1 [file 44319_2024_248_MOESM3_ESM.zip › Figure 1/Fig. 1I/MG132 0.5uM.tif]

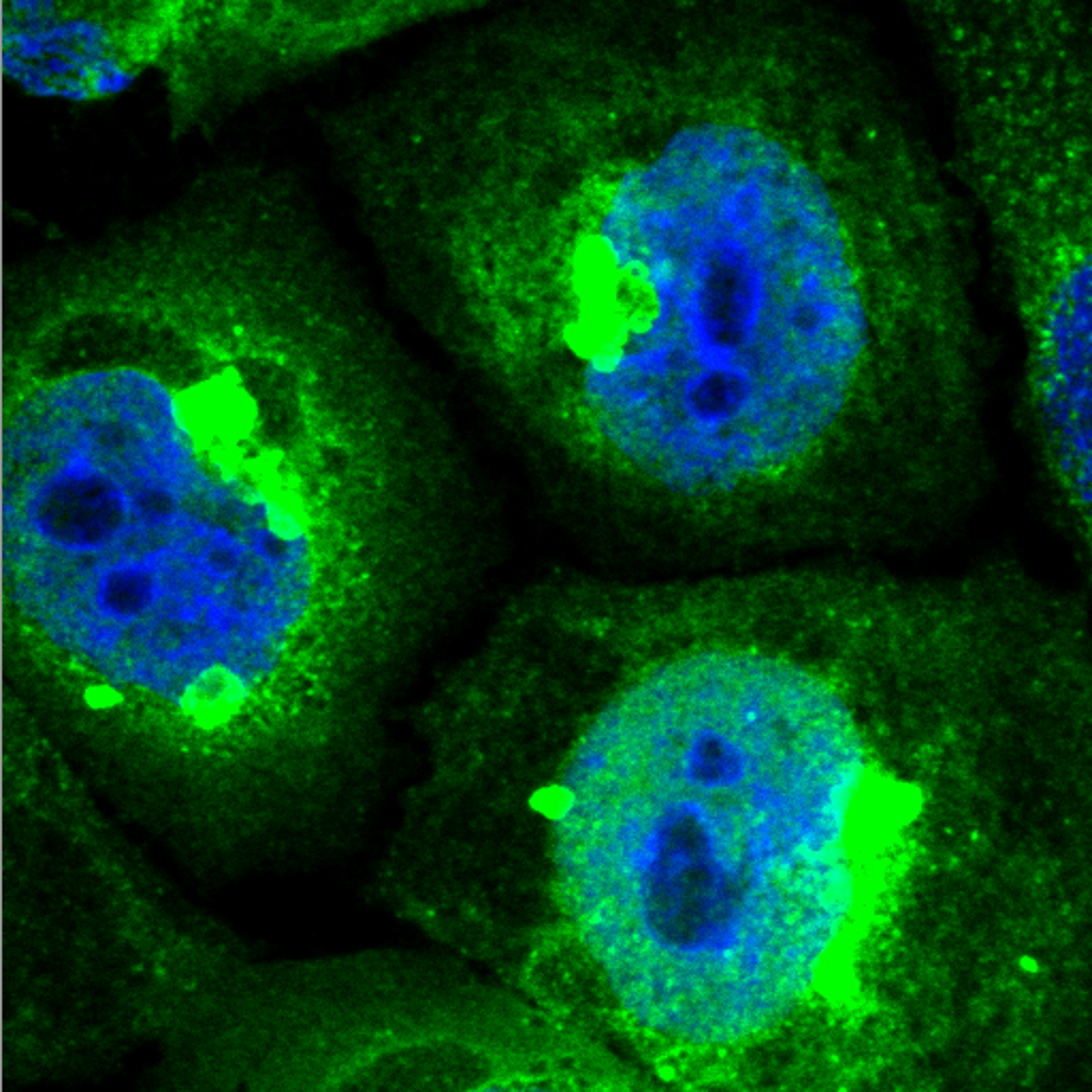

Supplement: Supplementary file 3 — Source data Fig. 1 [file 44319_2024_248_MOESM3_ESM.zip › Figure 1/Fig. 1I/MG132 1uM + KN-93.tif]

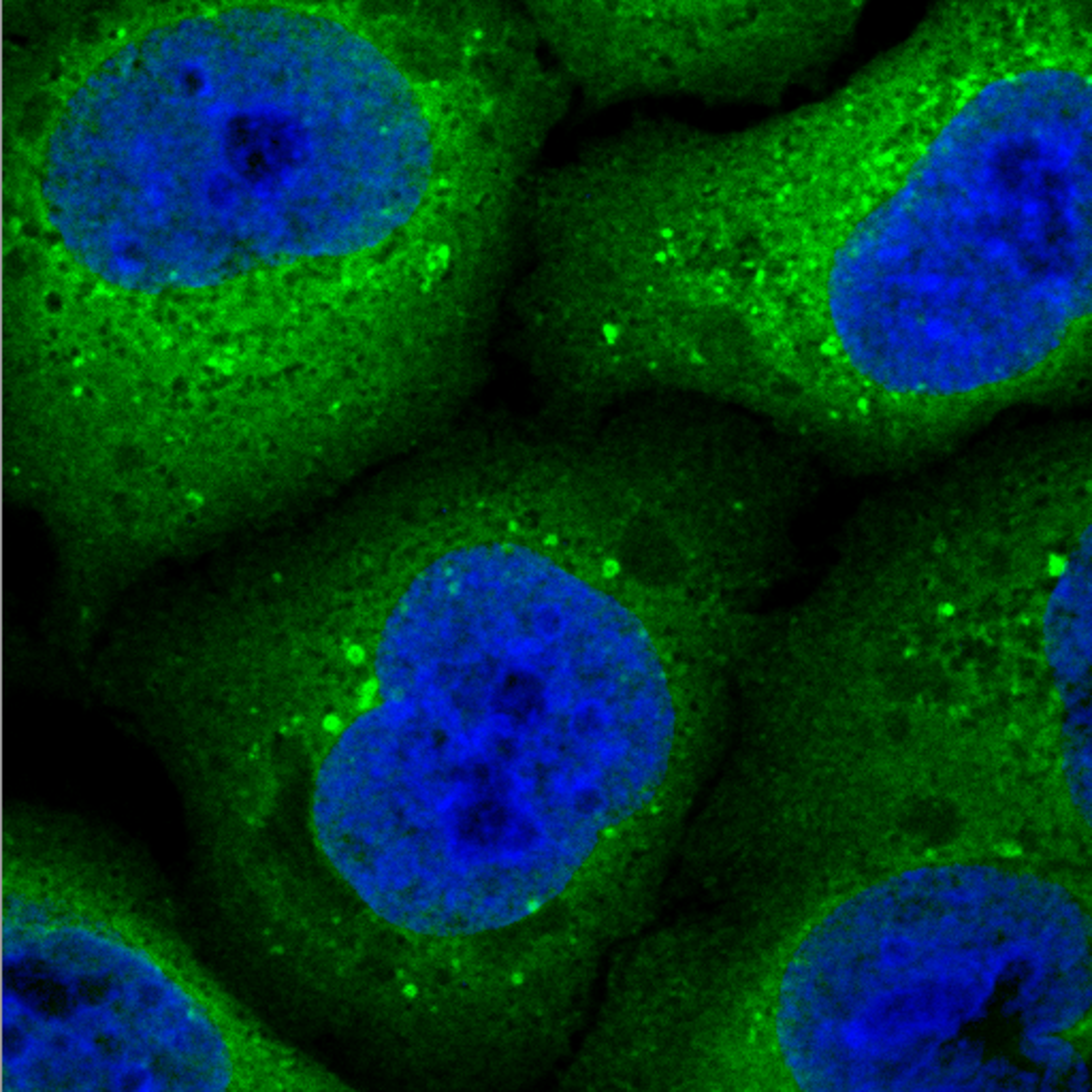

Supplement: Supplementary file 3 — Source data Fig. 1 [file 44319_2024_248_MOESM3_ESM.zip › Figure 1/Fig. 1I/MG132 1uM.tif]

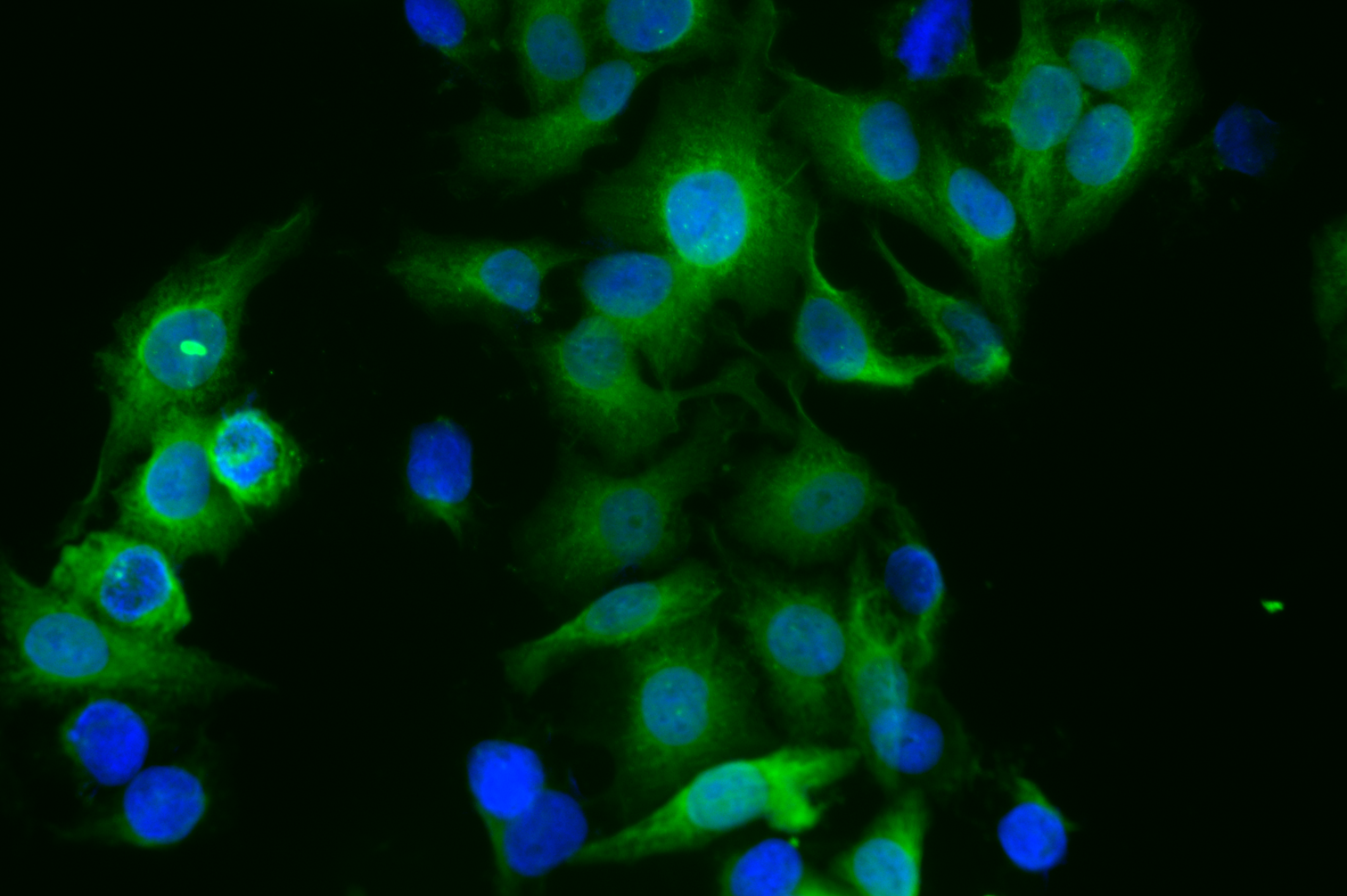

Supplement: Supplementary file 3 — Source data Fig. 1 [file 44319_2024_248_MOESM3_ESM.zip › Figure 1/Fig. 1N/MG132.tif]

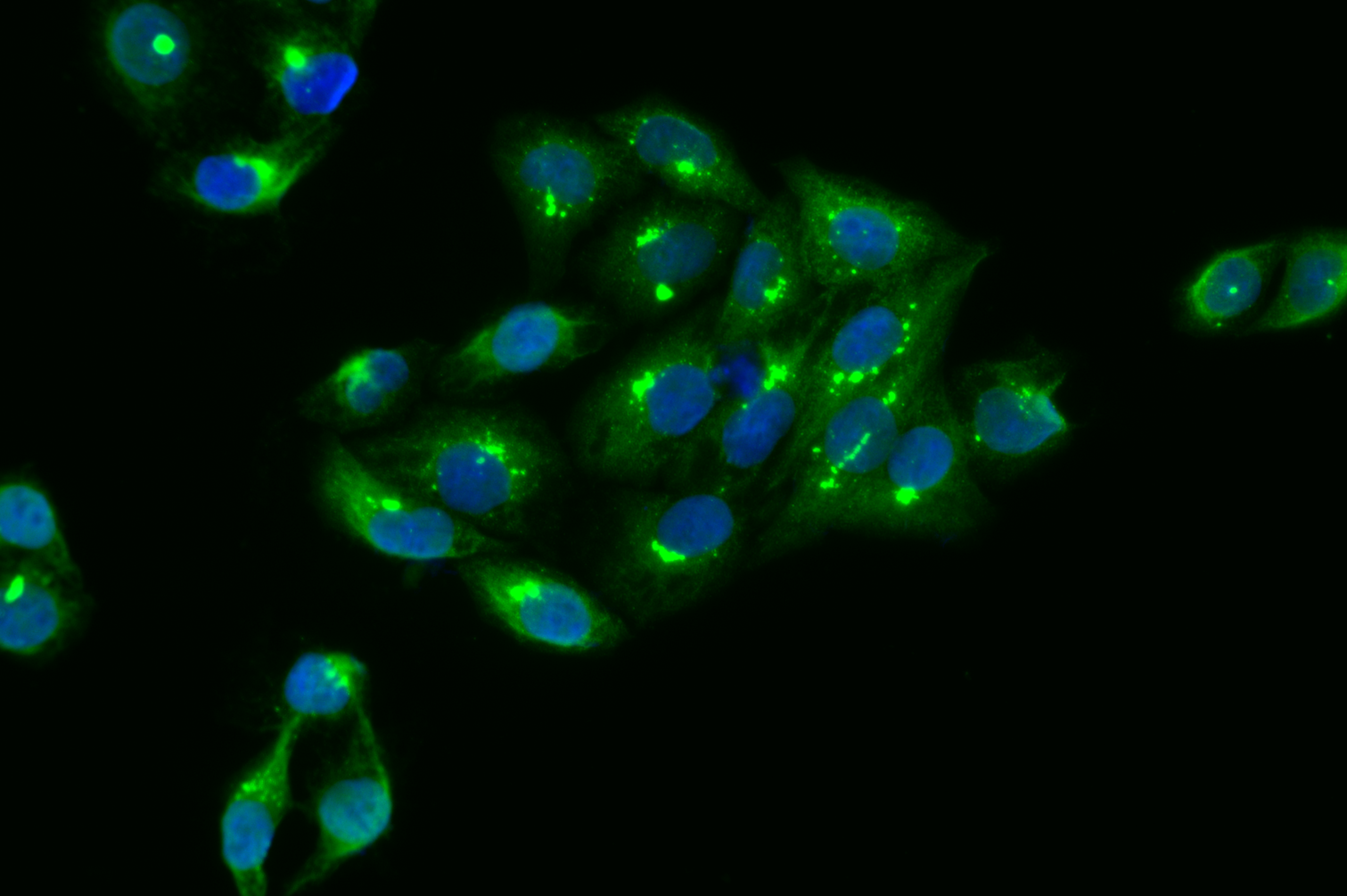

Supplement: Supplementary file 3 — Source data Fig. 1 [file 44319_2024_248_MOESM3_ESM.zip › Figure 1/Fig. 1N/MG132+KN-93.tif]

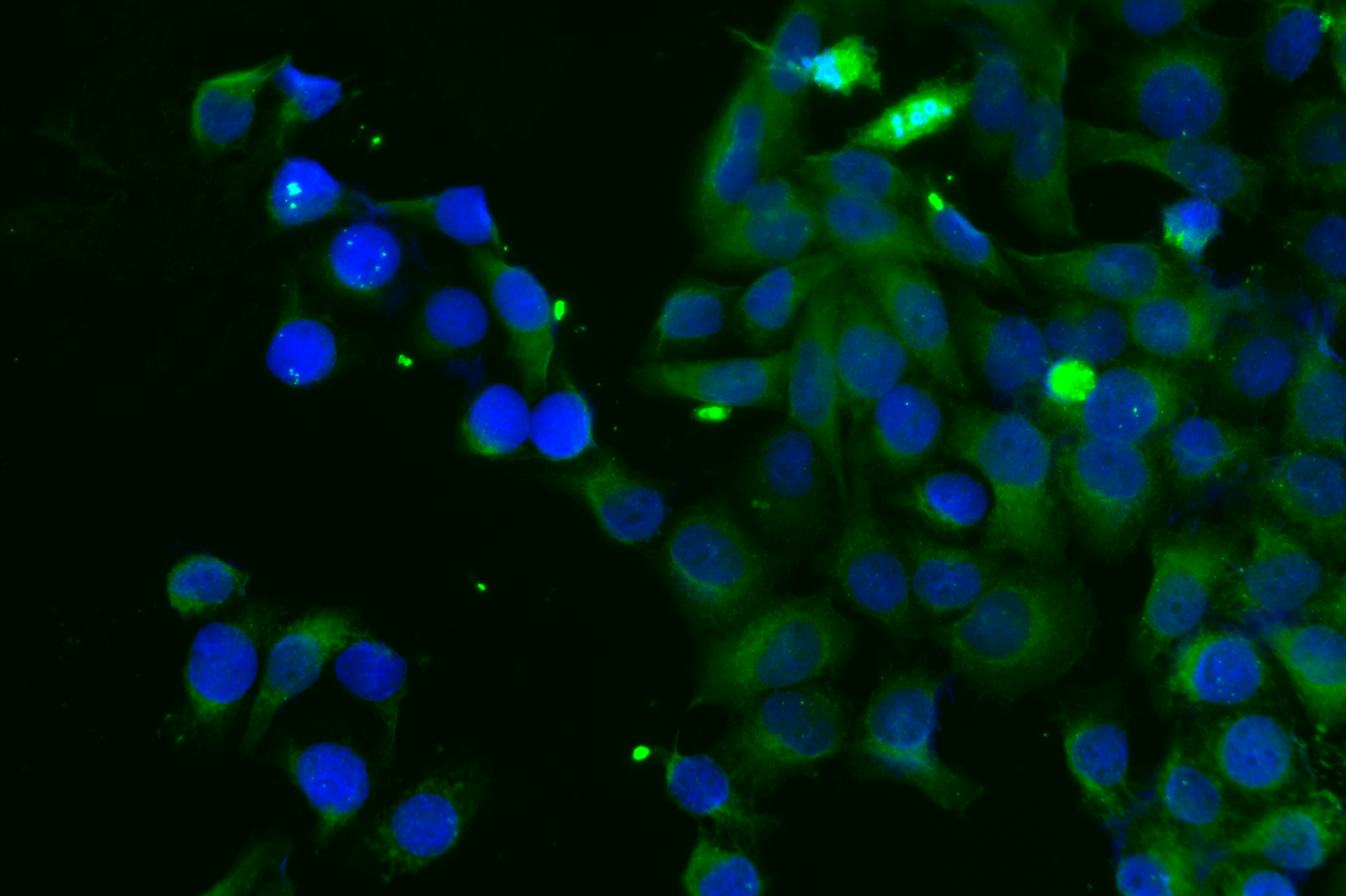

Supplement: Supplementary file 3 — Source data Fig. 1 [file 44319_2024_248_MOESM3_ESM.zip › Figure 1/Fig. 1N/MG132+KN-93+CHX.tif]

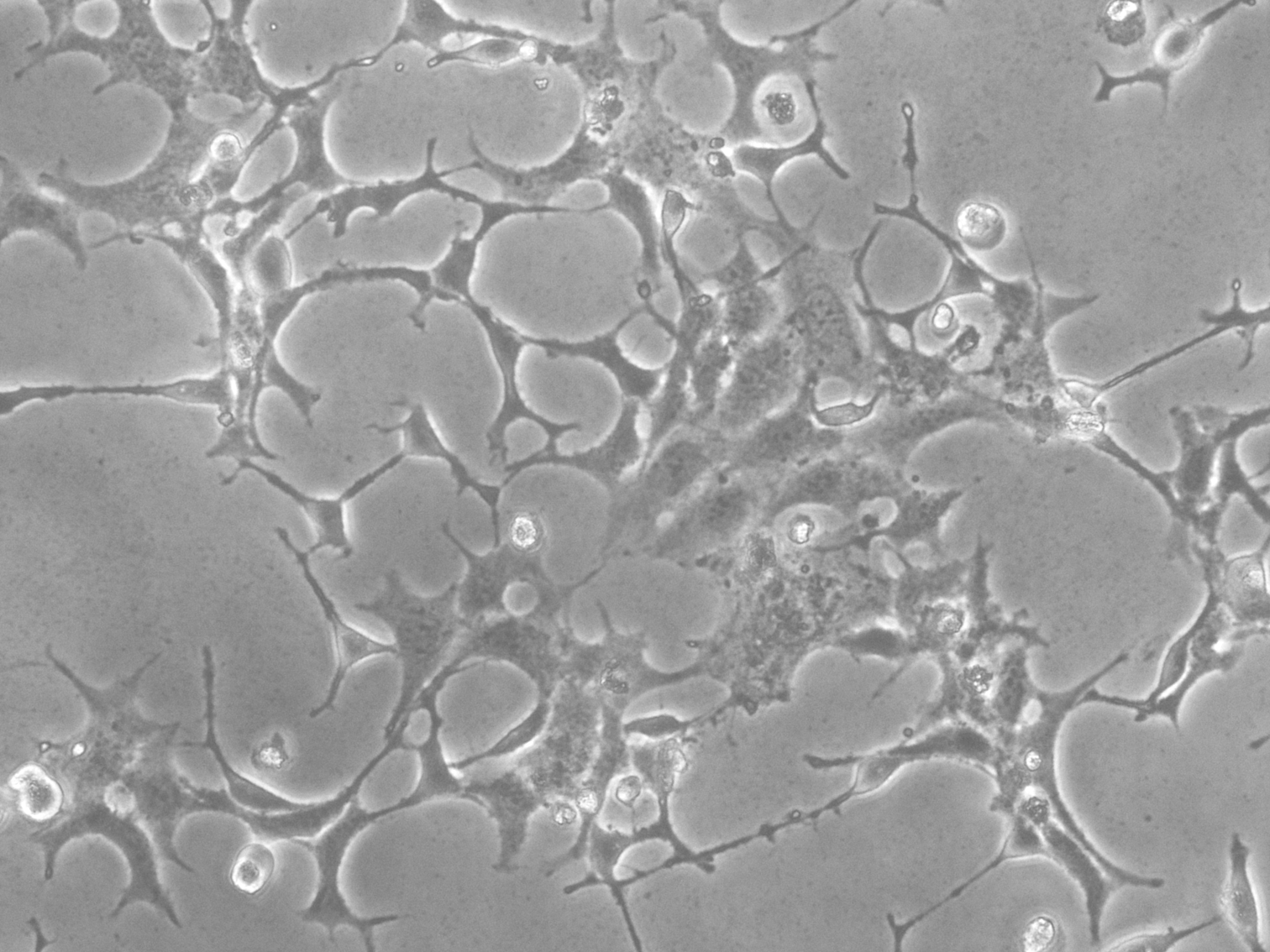

Supplement: Supplementary file 4 — Source data Fig. 2 [file 44319_2024_248_MOESM4_ESM.zip › Figure 2/Fig. 2A/293-shCaMKIIs-DMSO.tif]

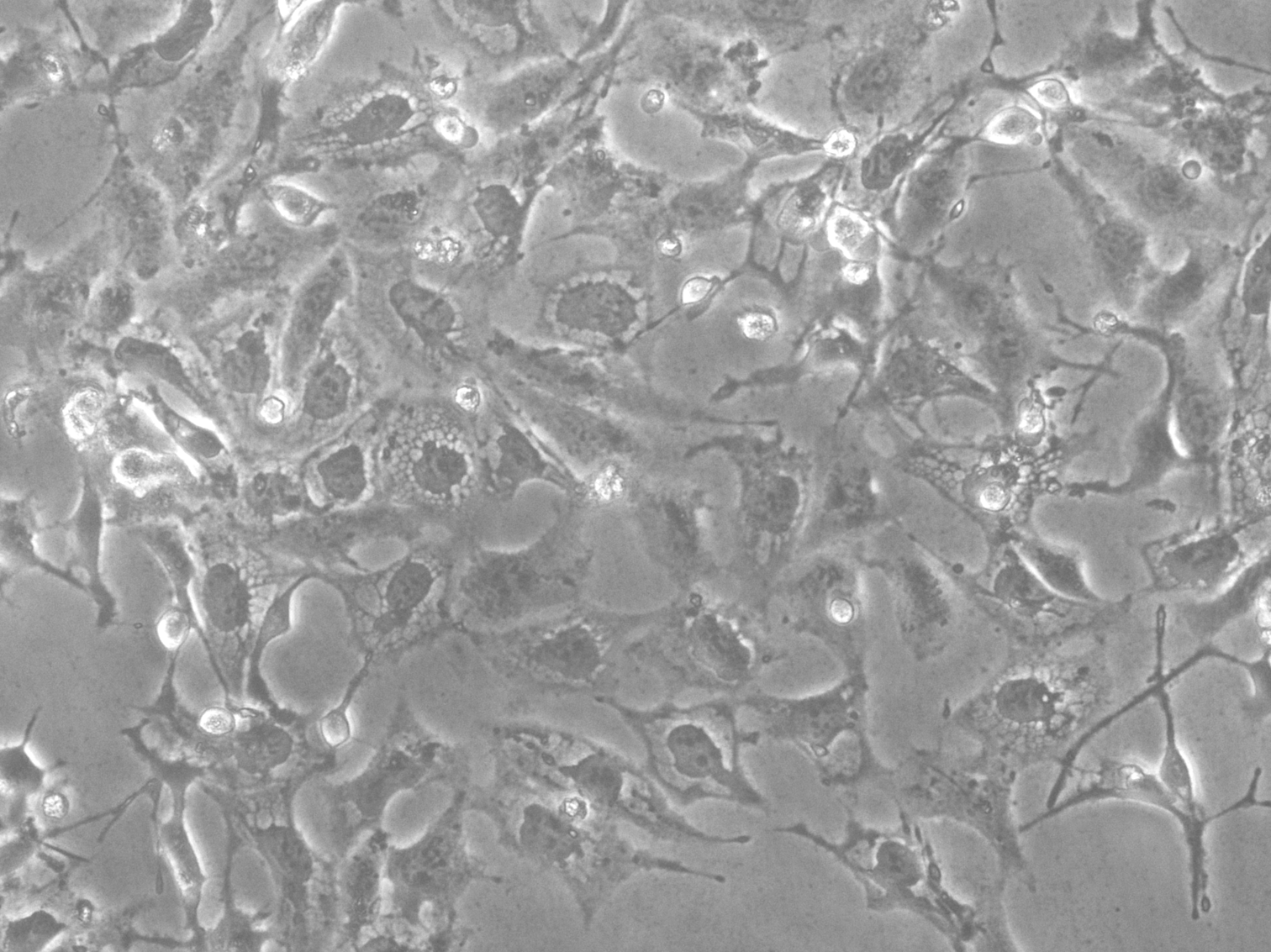

Supplement: Supplementary file 4 — Source data Fig. 2 [file 44319_2024_248_MOESM4_ESM.zip › Figure 2/Fig. 2A/293-shCaMKIIs-MG132.tif]

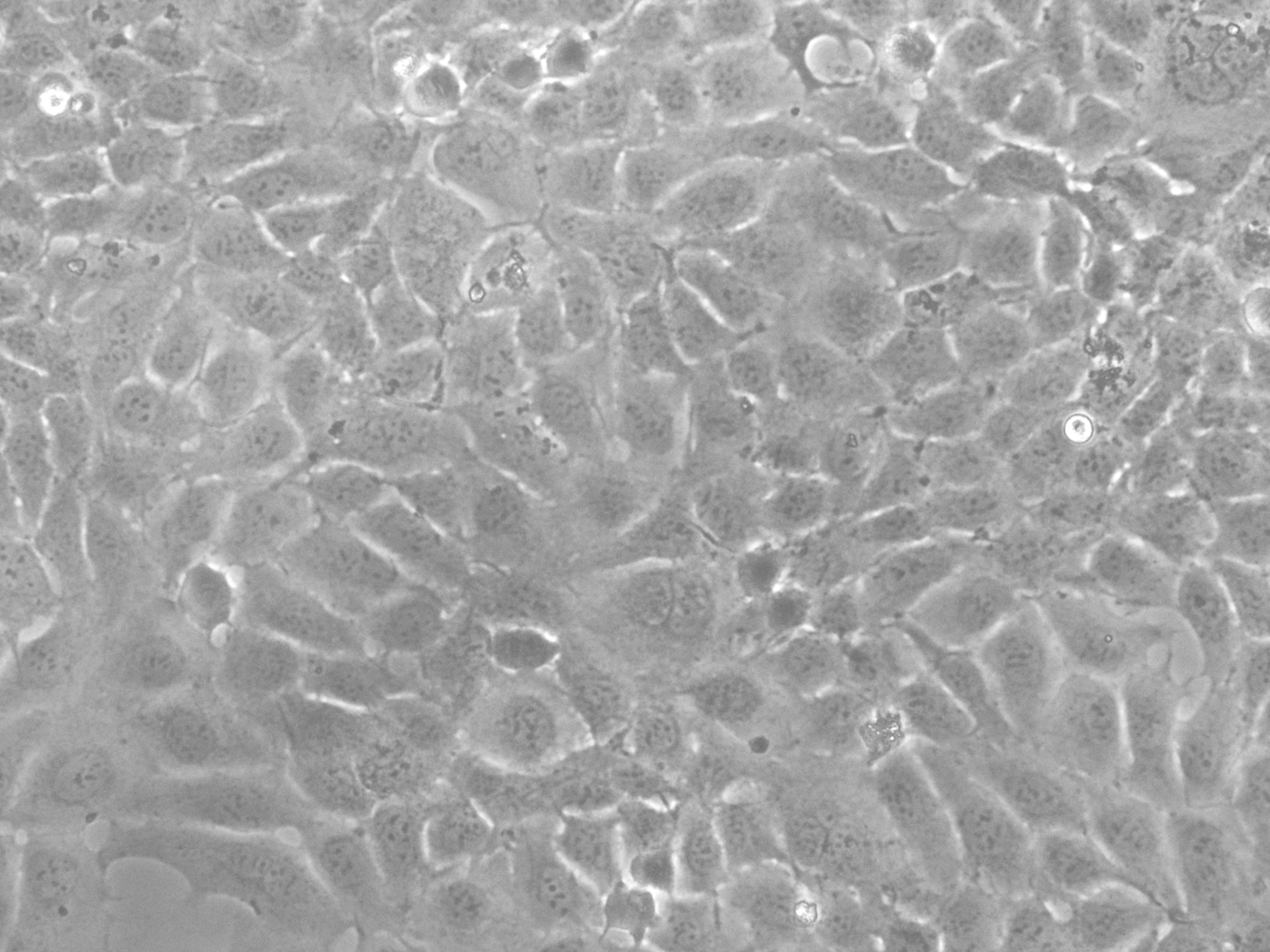

Supplement: Supplementary file 4 — Source data Fig. 2 [file 44319_2024_248_MOESM4_ESM.zip › Figure 2/Fig. 2A/293-shScr-DMSO.tif]

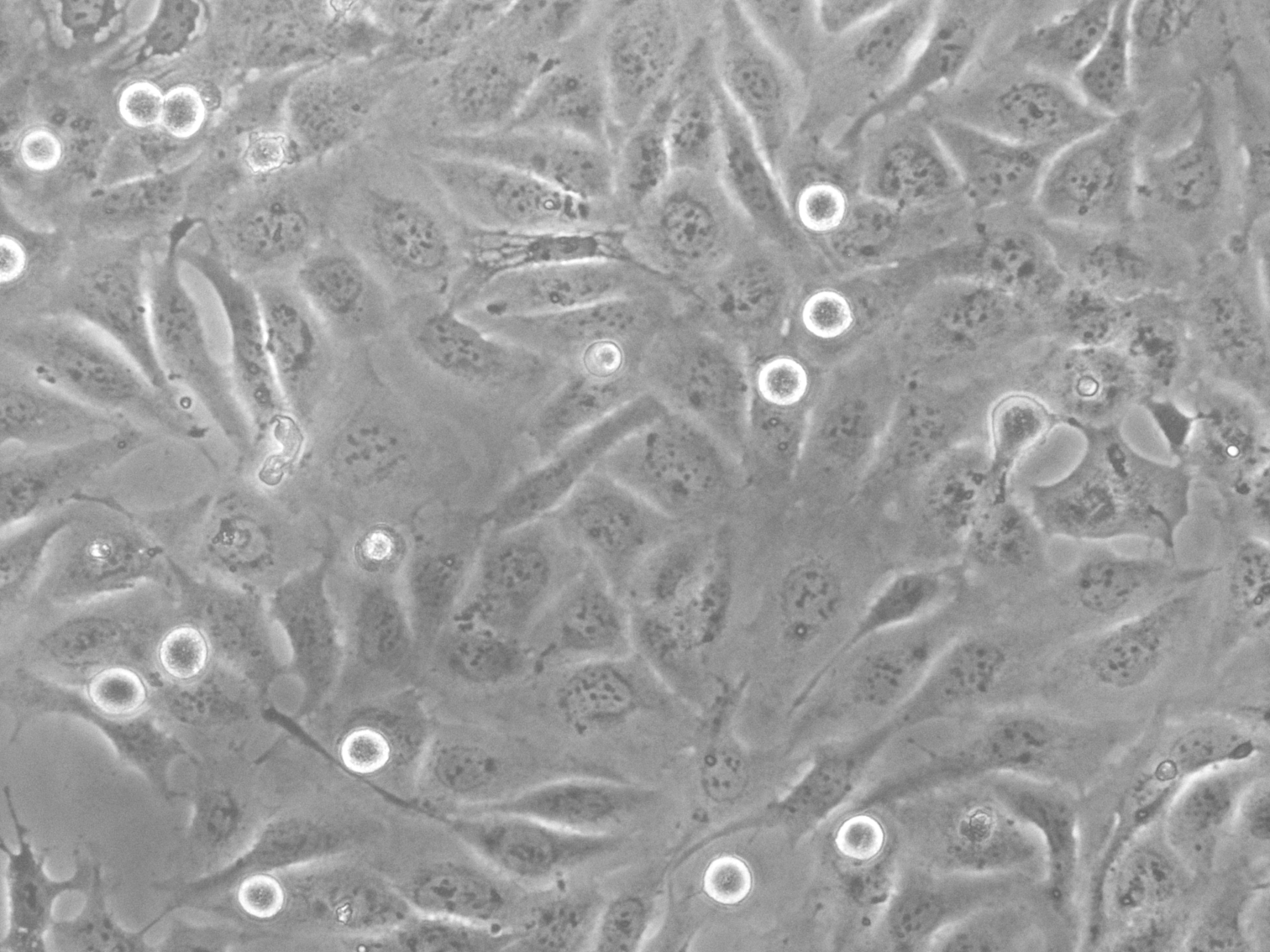

Supplement: Supplementary file 4 — Source data Fig. 2 [file 44319_2024_248_MOESM4_ESM.zip › Figure 2/Fig. 2A/293-shScr-MG132.tif]

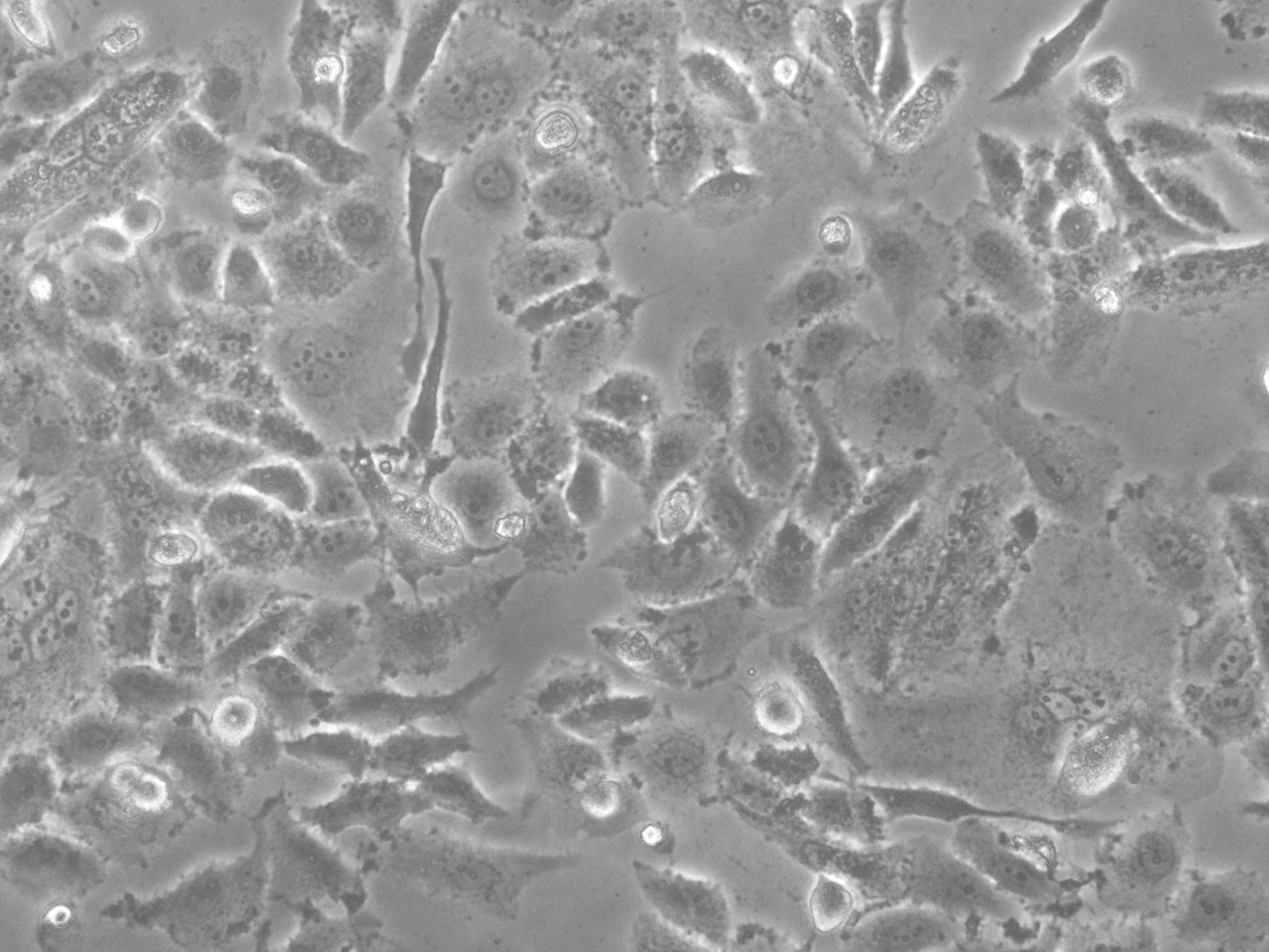

Supplement: Supplementary file 4 — Source data Fig. 2 [file 44319_2024_248_MOESM4_ESM.zip › Figure 2/Fig. 2A/Hela-shCaMKIIs-DMSO.tif]

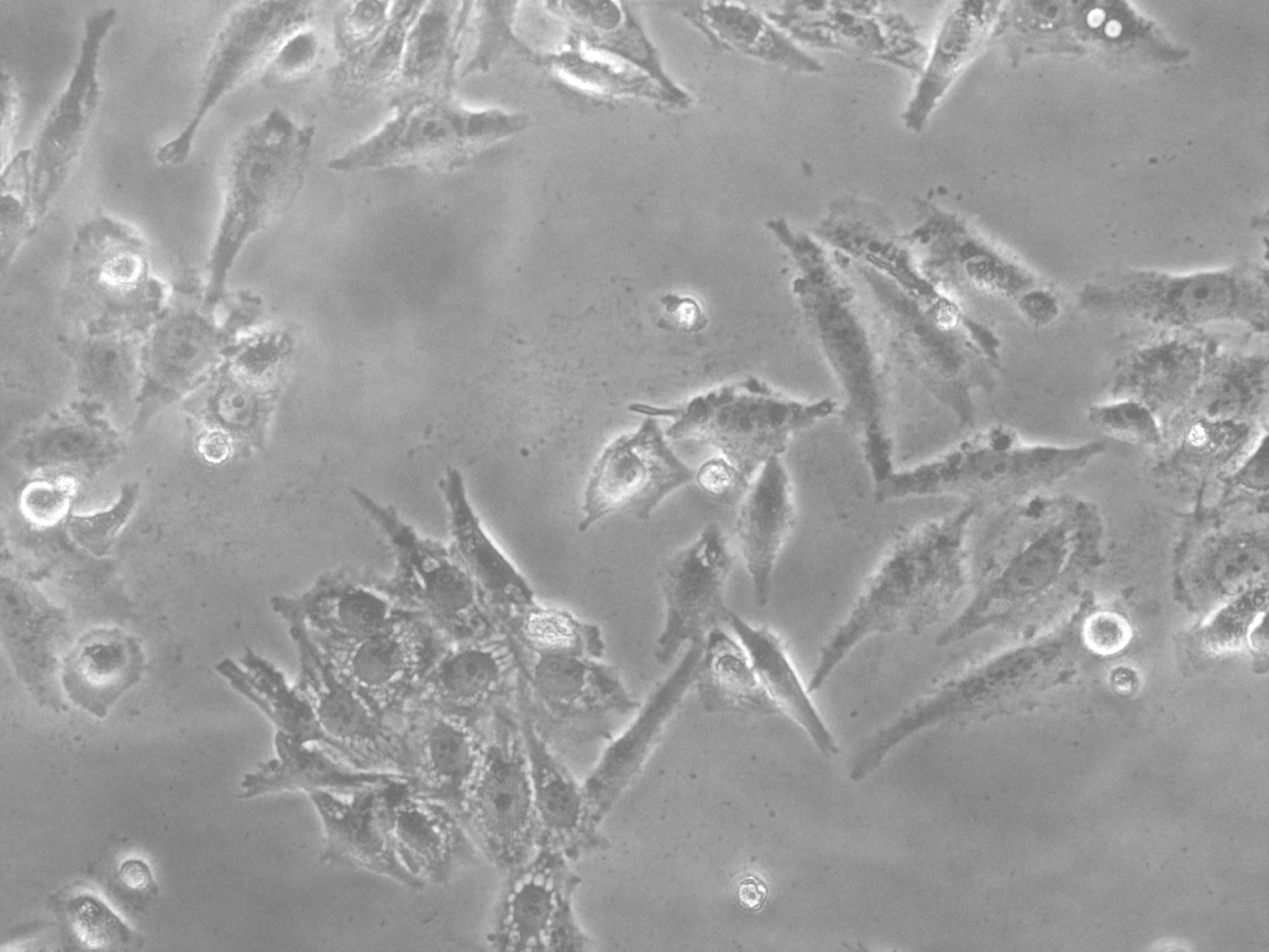

Supplement: Supplementary file 4 — Source data Fig. 2 [file 44319_2024_248_MOESM4_ESM.zip › Figure 2/Fig. 2A/Hela-shCaMKIIs-MG132.tif]

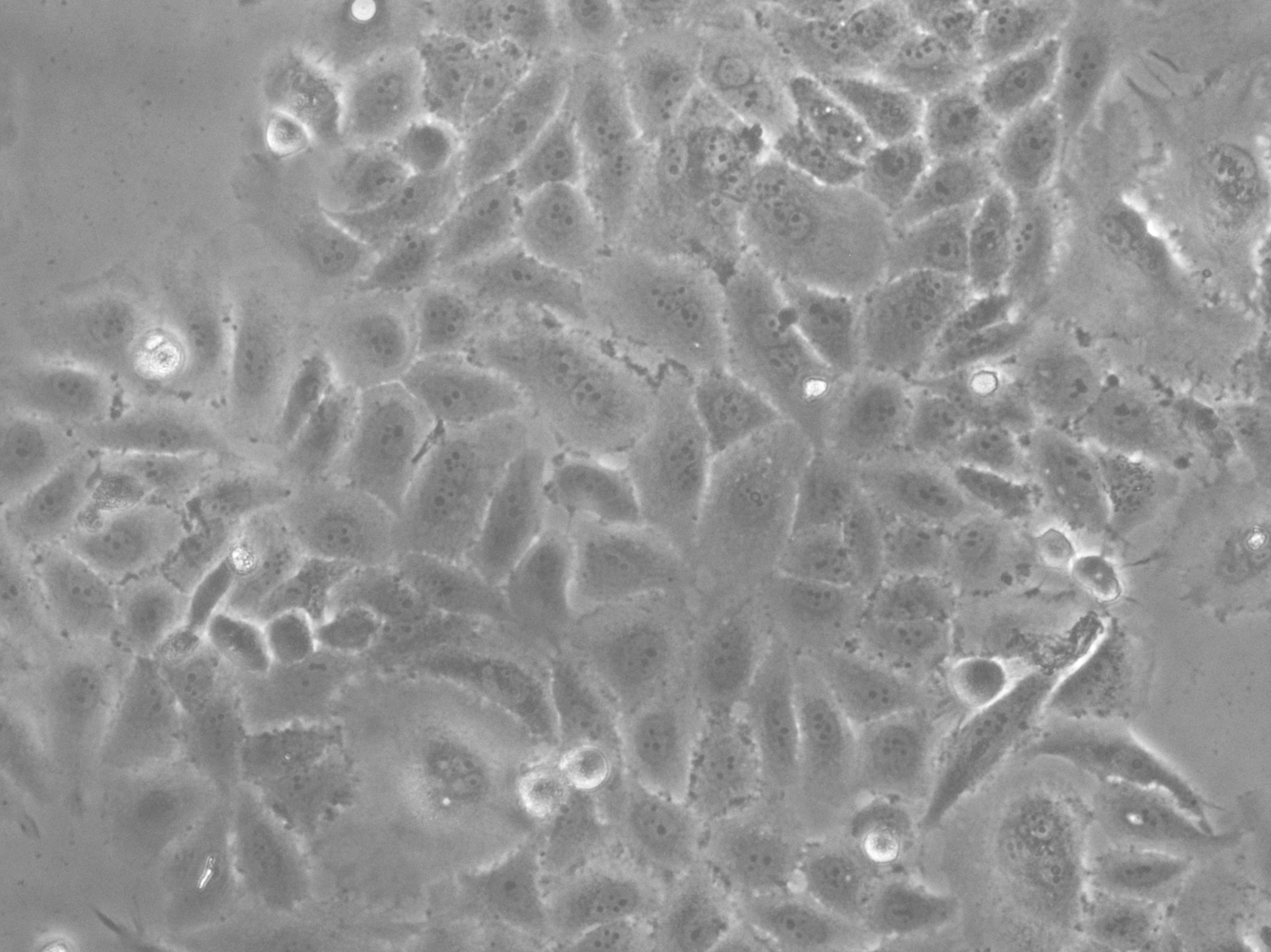

Supplement: Supplementary file 4 — Source data Fig. 2 [file 44319_2024_248_MOESM4_ESM.zip › Figure 2/Fig. 2A/Hela-shScr-DMSO.tif]

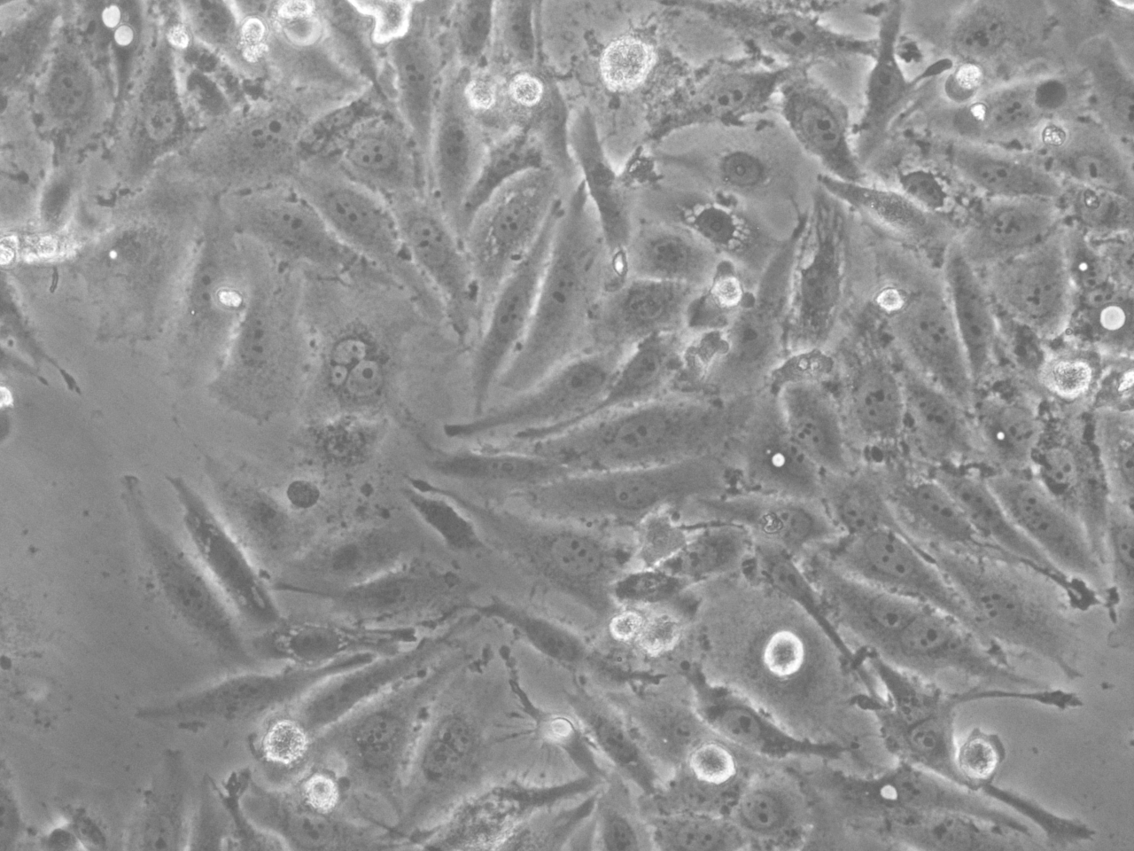

Supplement: Supplementary file 4 — Source data Fig. 2 [file 44319_2024_248_MOESM4_ESM.zip › Figure 2/Fig. 2A/Hela-shScr-MG132.tif]

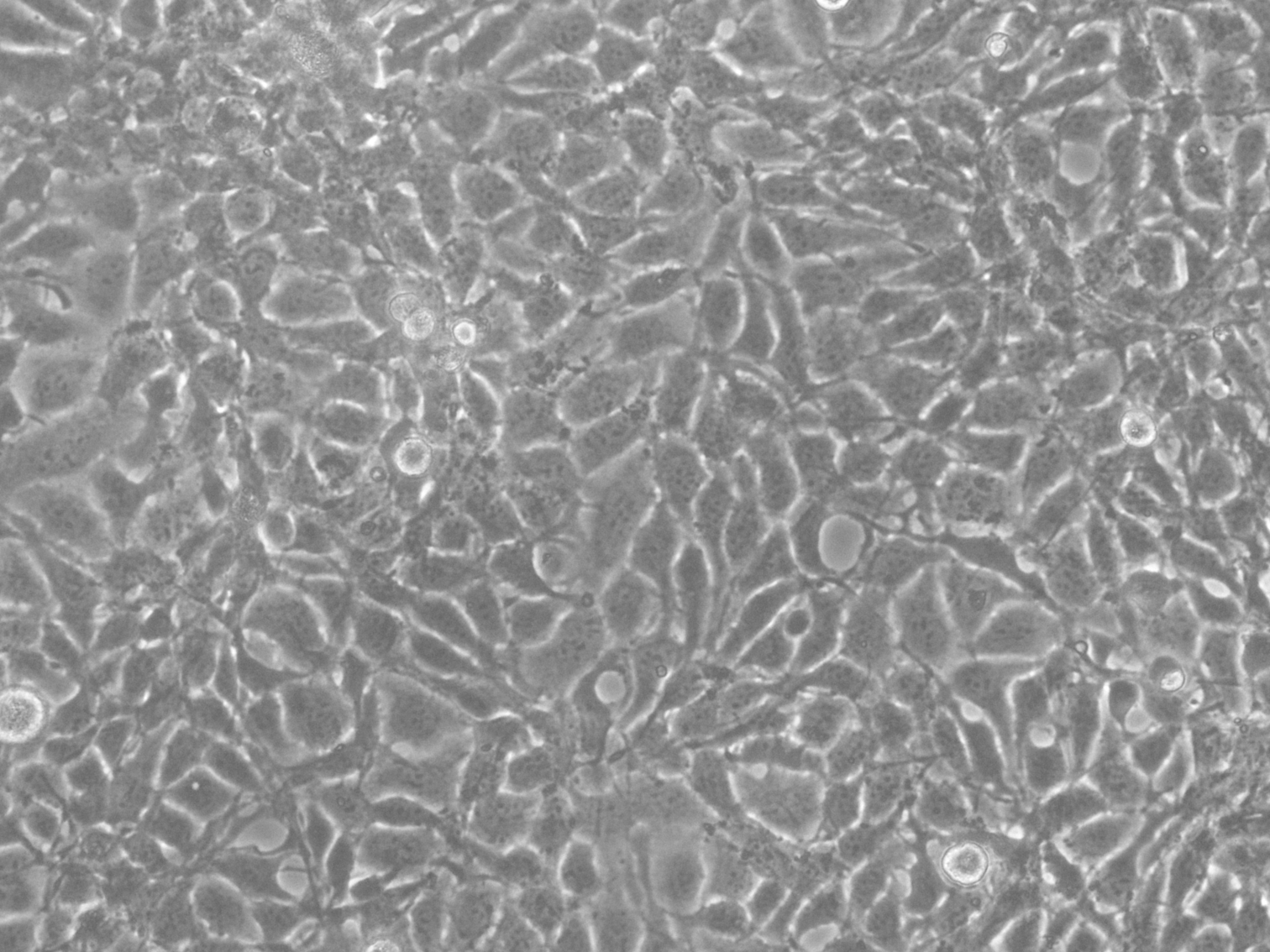

Supplement: Supplementary file 4 — Source data Fig. 2 [file 44319_2024_248_MOESM4_ESM.zip › Figure 2/Fig. 2C/293-DMSO.tif]

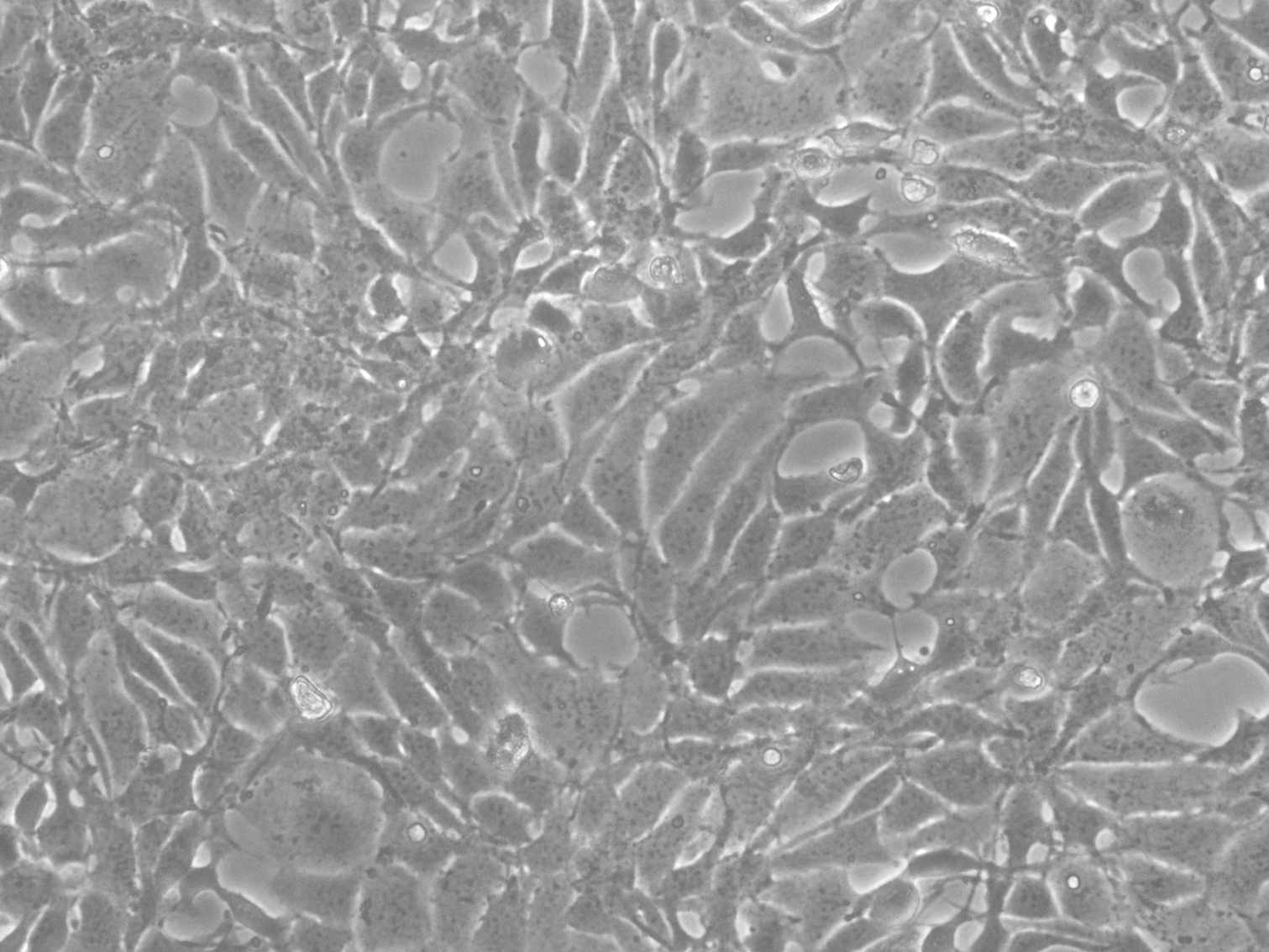

Supplement: Supplementary file 4 — Source data Fig. 2 [file 44319_2024_248_MOESM4_ESM.zip › Figure 2/Fig. 2C/293-KN-93.tif]

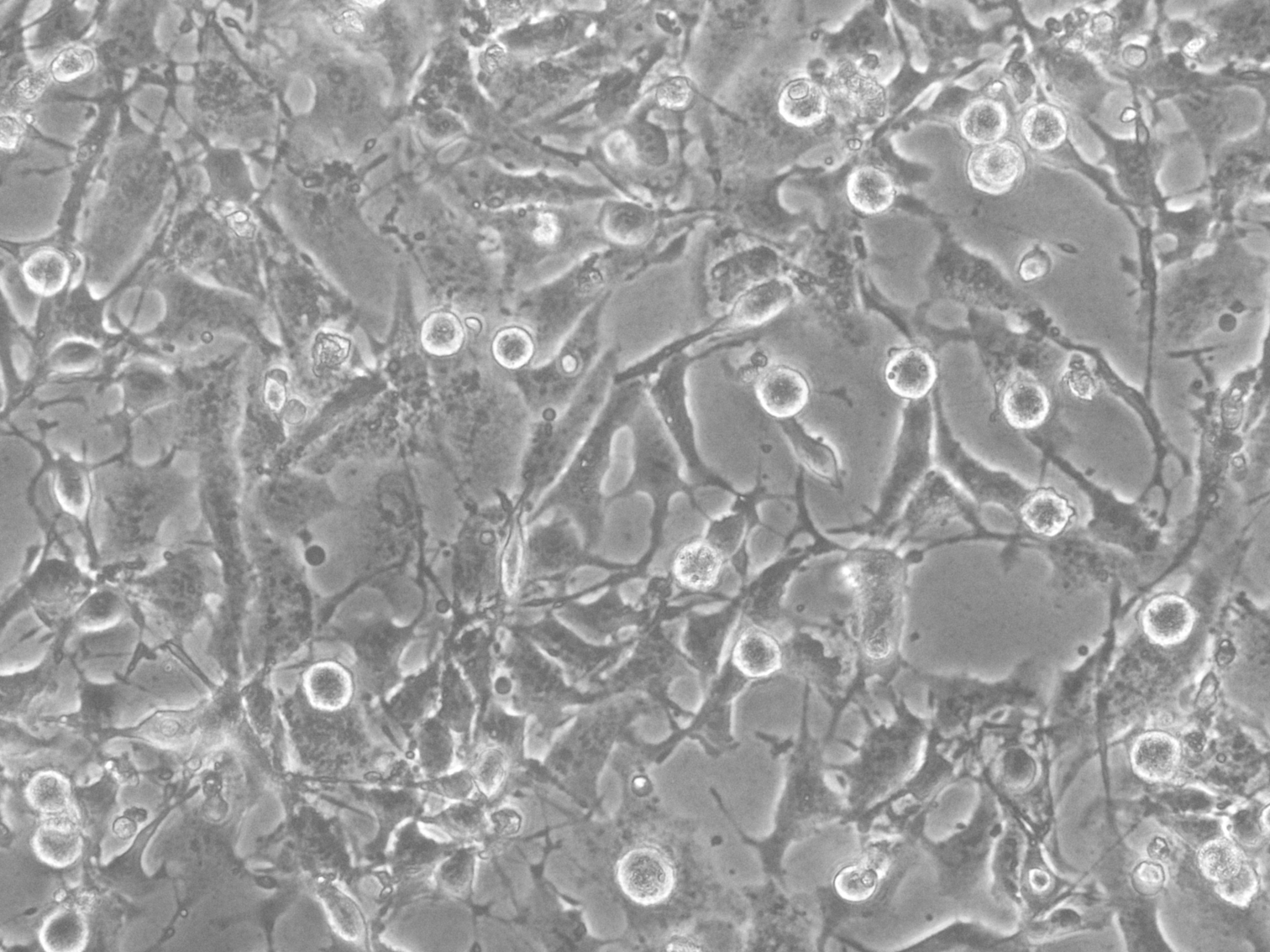

Supplement: Supplementary file 4 — Source data Fig. 2 [file 44319_2024_248_MOESM4_ESM.zip › Figure 2/Fig. 2C/293-MG132.tif]

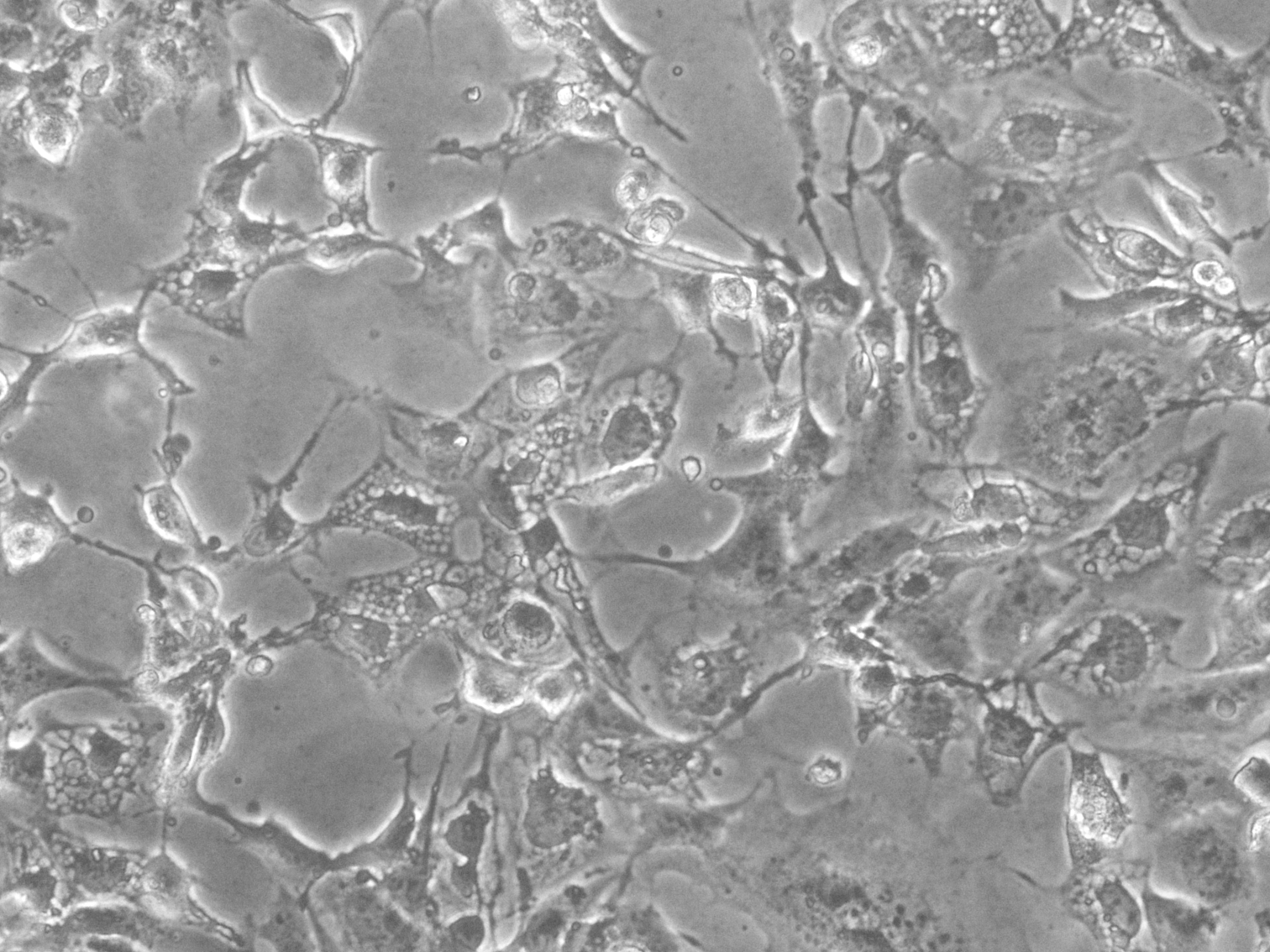

Supplement: Supplementary file 4 — Source data Fig. 2 [file 44319_2024_248_MOESM4_ESM.zip › Figure 2/Fig. 2C/293-MG132+KN-93.tif]

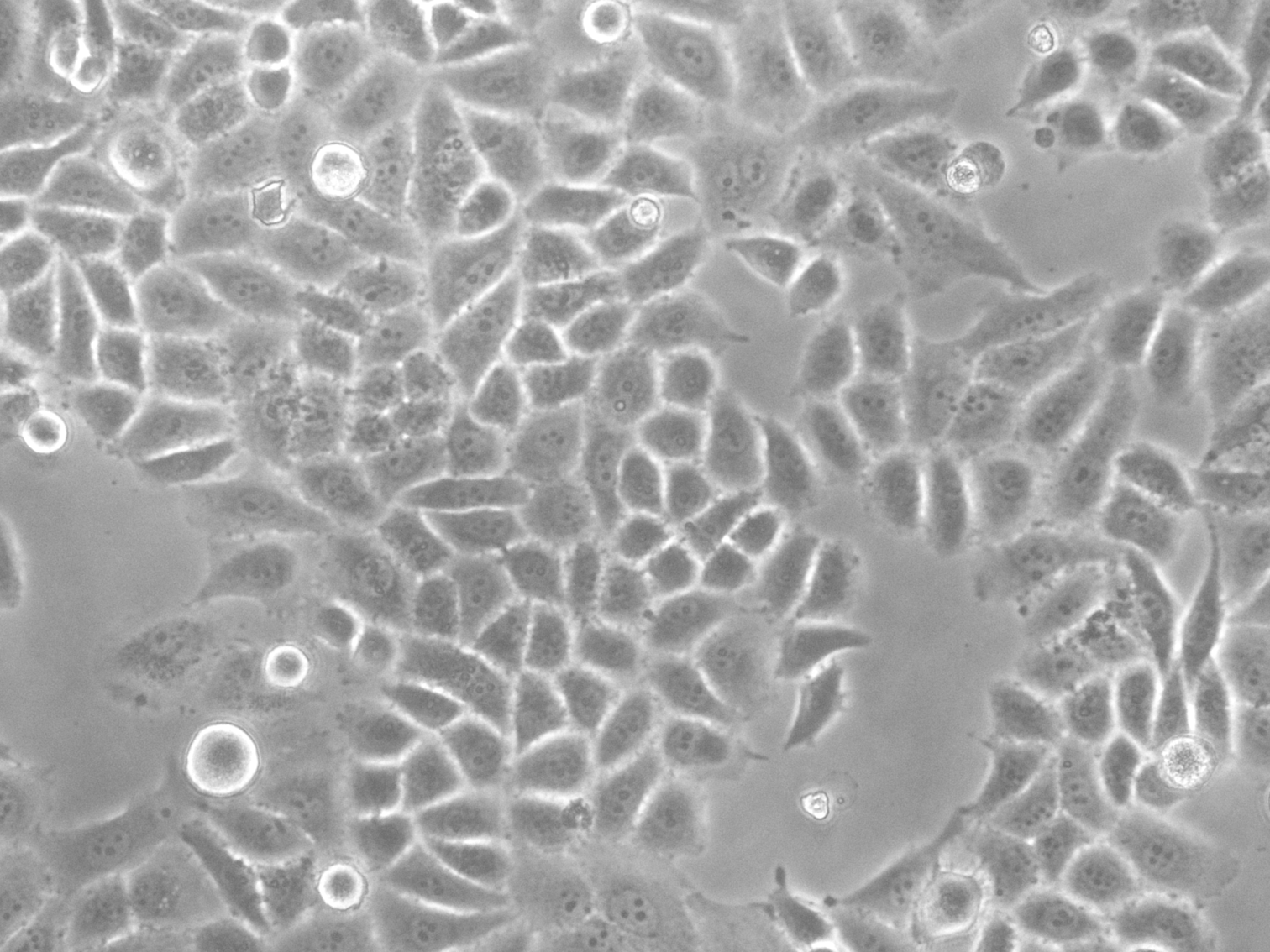

Supplement: Supplementary file 4 — Source data Fig. 2 [file 44319_2024_248_MOESM4_ESM.zip › Figure 2/Fig. 2C/Hela-DMSO.tif]

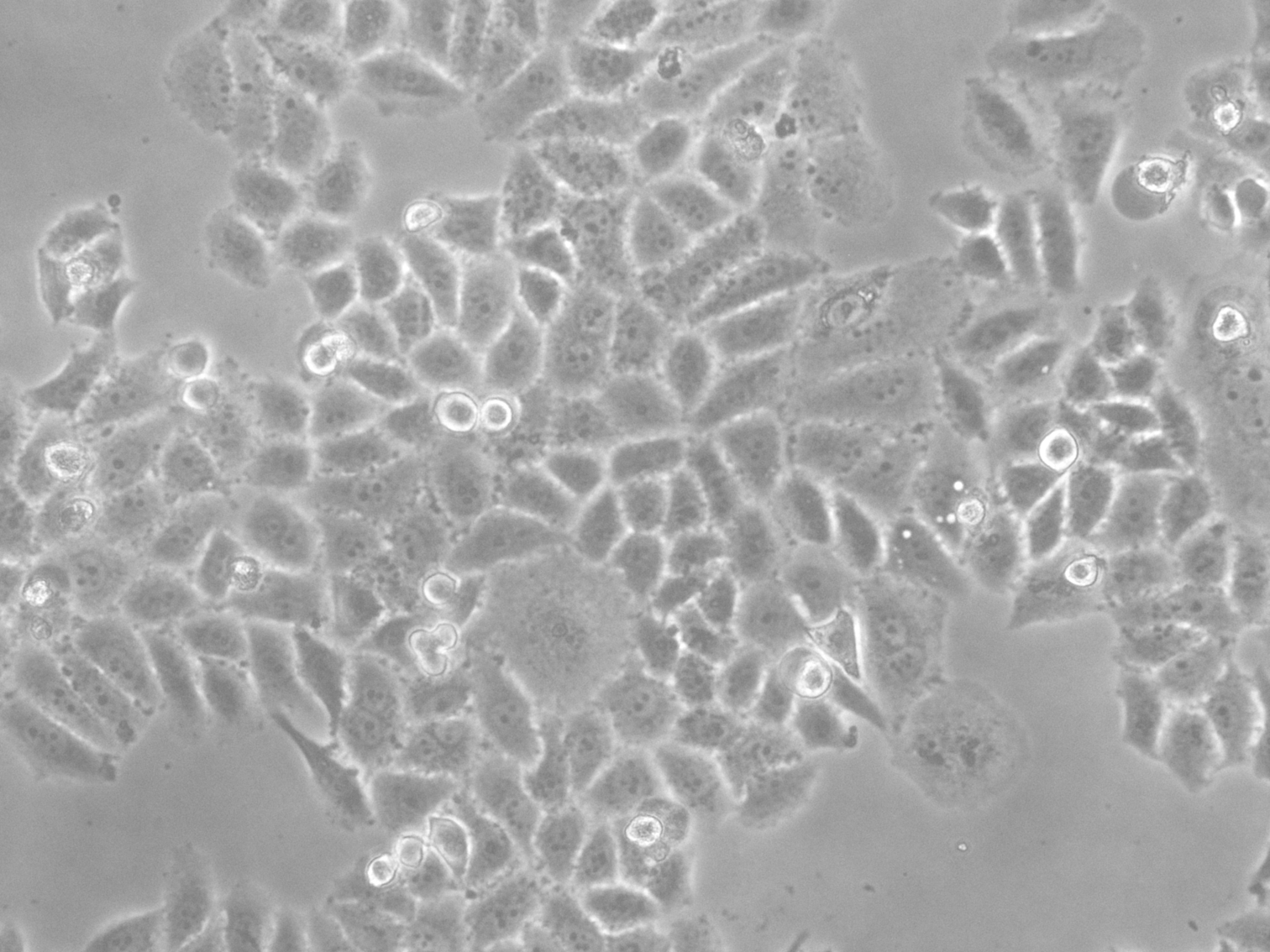

Supplement: Supplementary file 4 — Source data Fig. 2 [file 44319_2024_248_MOESM4_ESM.zip › Figure 2/Fig. 2C/Hela-KN-93.tif]

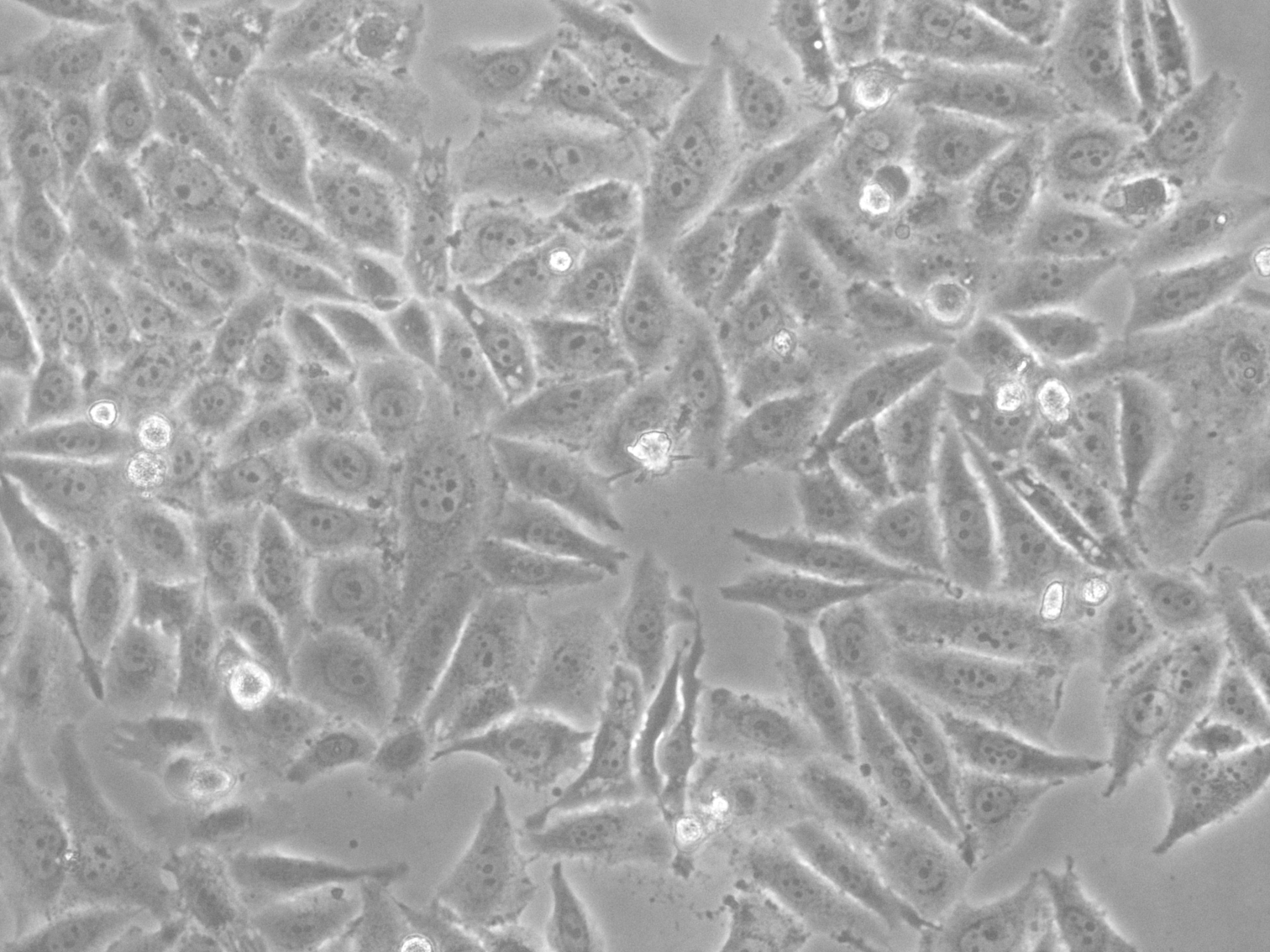

Supplement: Supplementary file 4 — Source data Fig. 2 [file 44319_2024_248_MOESM4_ESM.zip › Figure 2/Fig. 2C/Hela-MG132.tif]

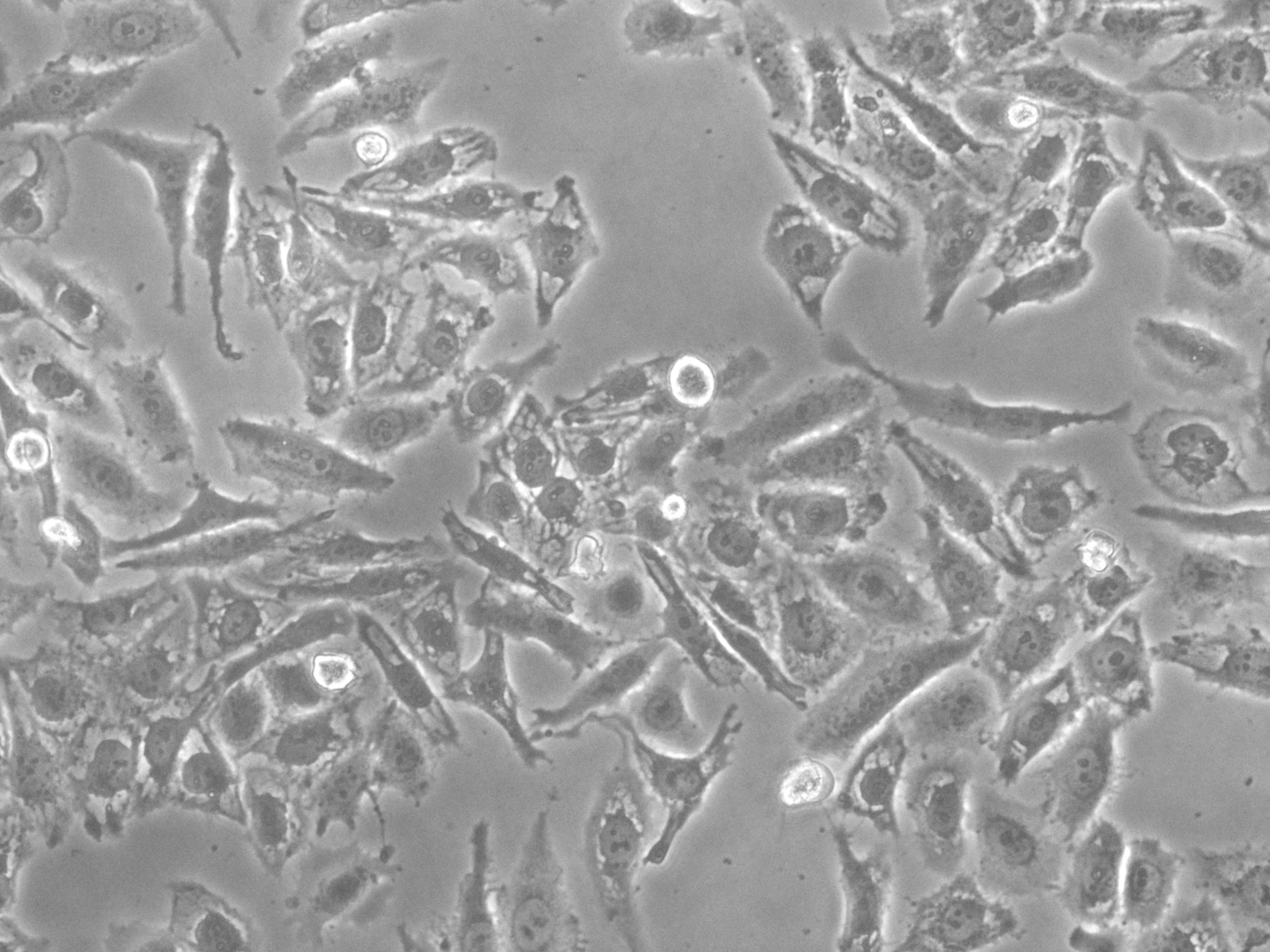

Supplement: Supplementary file 4 — Source data Fig. 2 [file 44319_2024_248_MOESM4_ESM.zip › Figure 2/Fig. 2C/Hela-MG132+KN-93.tif]

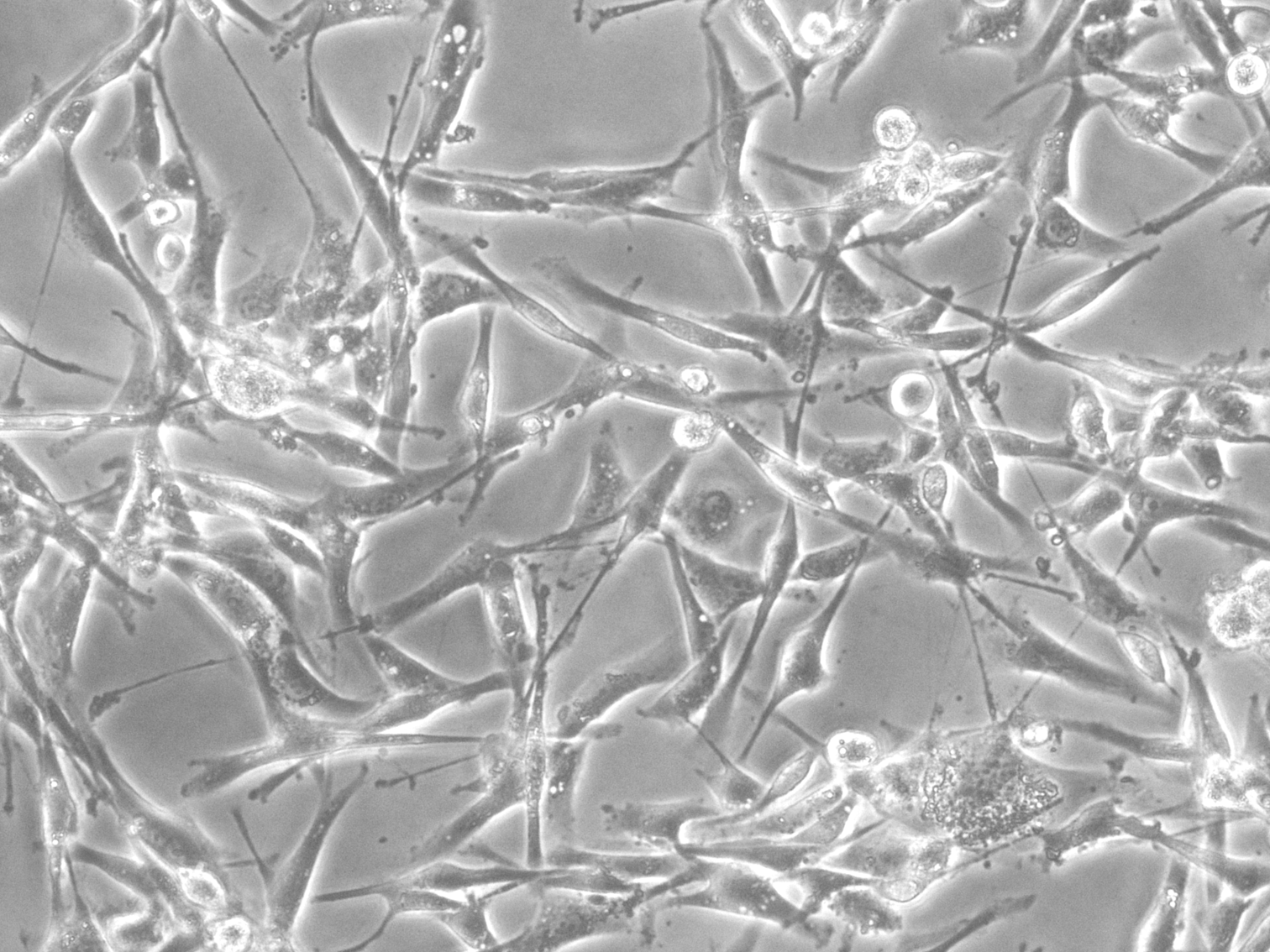

Supplement: Supplementary file 4 — Source data Fig. 2 [file 44319_2024_248_MOESM4_ESM.zip › Figure 2/Fig. 2C/U87MG-DMSO.tif]

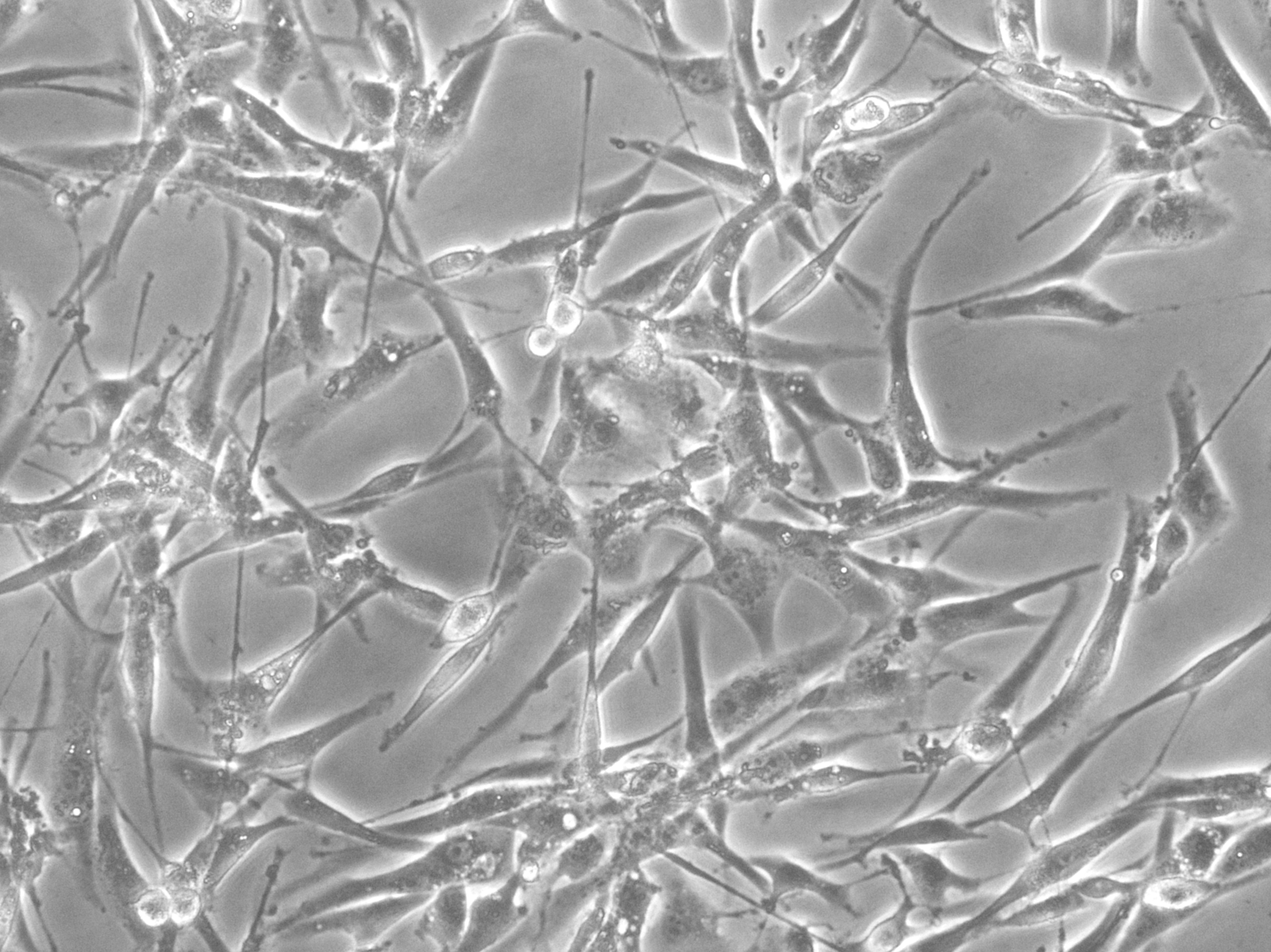

Supplement: Supplementary file 4 — Source data Fig. 2 [file 44319_2024_248_MOESM4_ESM.zip › Figure 2/Fig. 2C/U87MG-KN-93.tif]

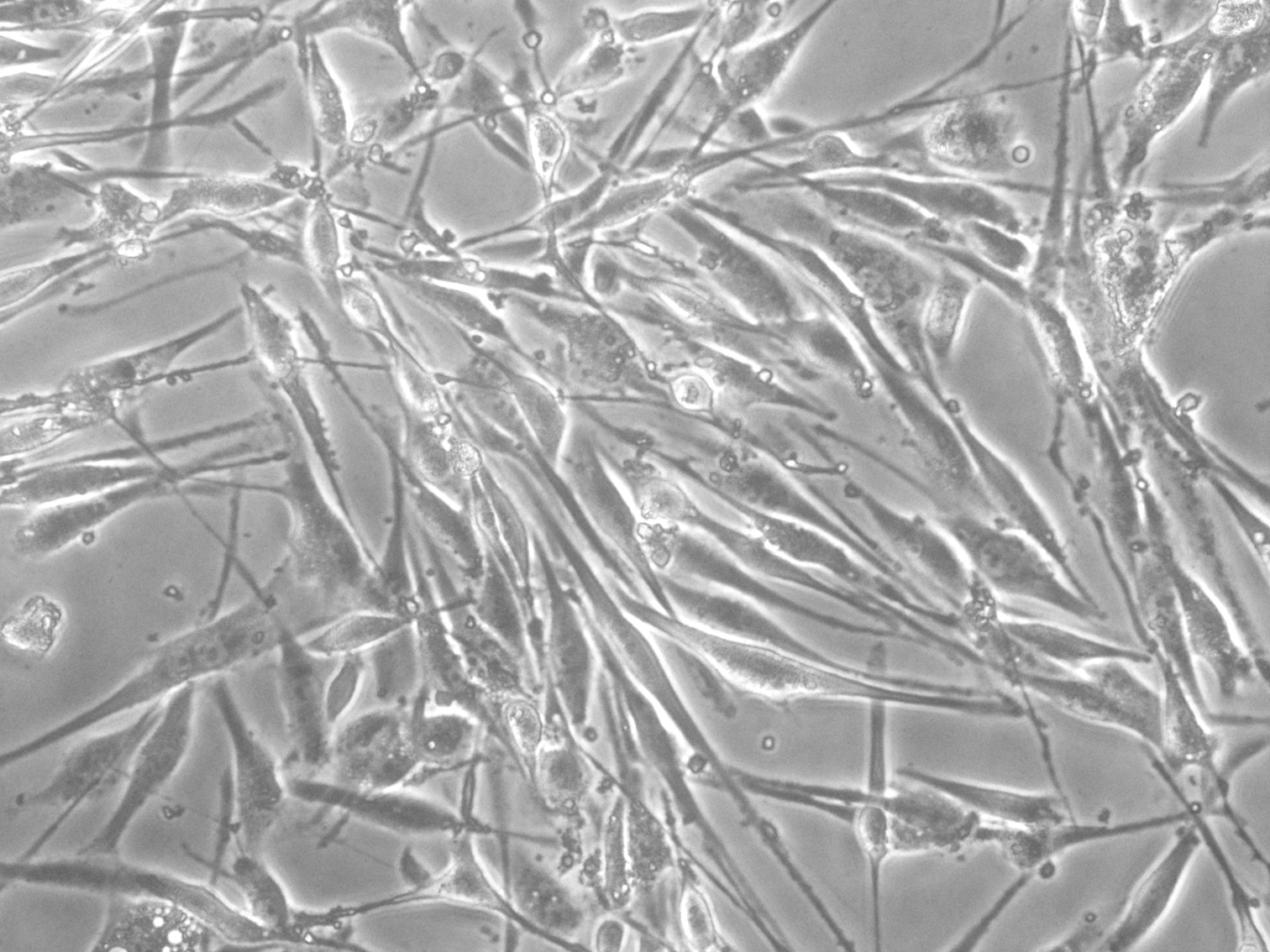

Supplement: Supplementary file 4 — Source data Fig. 2 [file 44319_2024_248_MOESM4_ESM.zip › Figure 2/Fig. 2C/U87MG-MG132.tif]

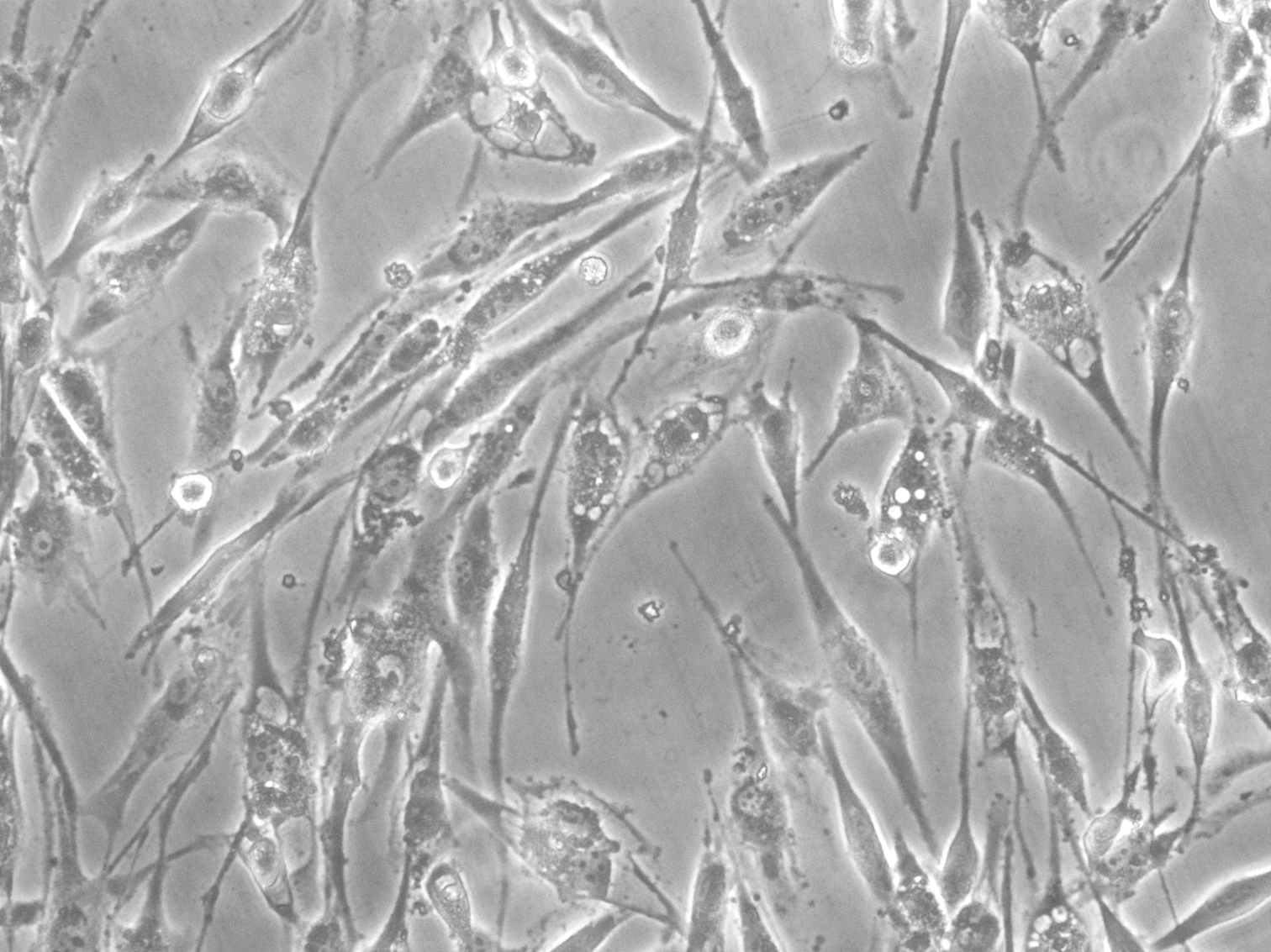

Supplement: Supplementary file 4 — Source data Fig. 2 [file 44319_2024_248_MOESM4_ESM.zip › Figure 2/Fig. 2C/U87MG-MG132+MG132.tif]

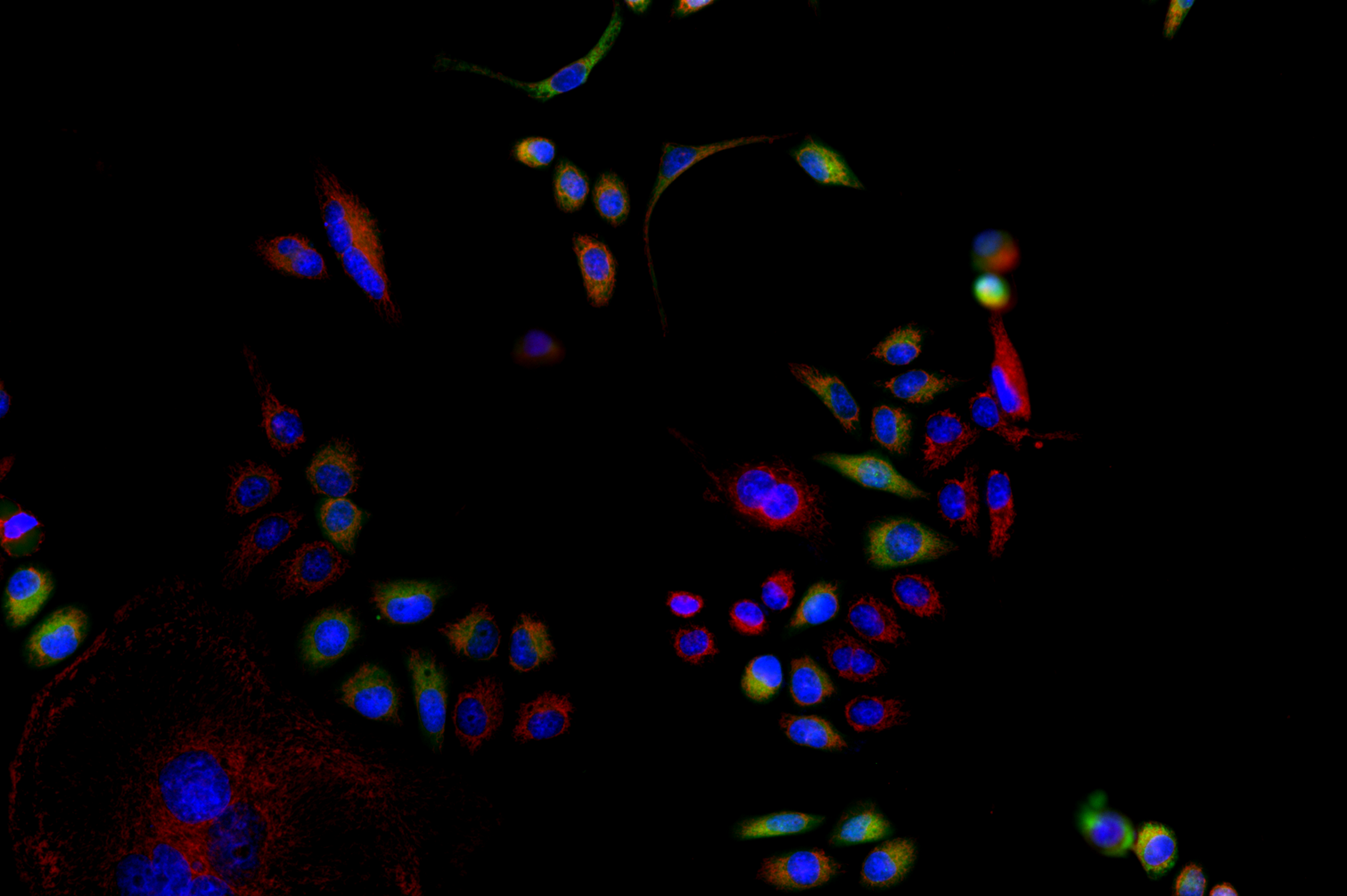

Supplement: Supplementary file 4 — Source data Fig. 2 [file 44319_2024_248_MOESM4_ESM.zip › Figure 2/Fig. 2E/DMSO 1.tif]

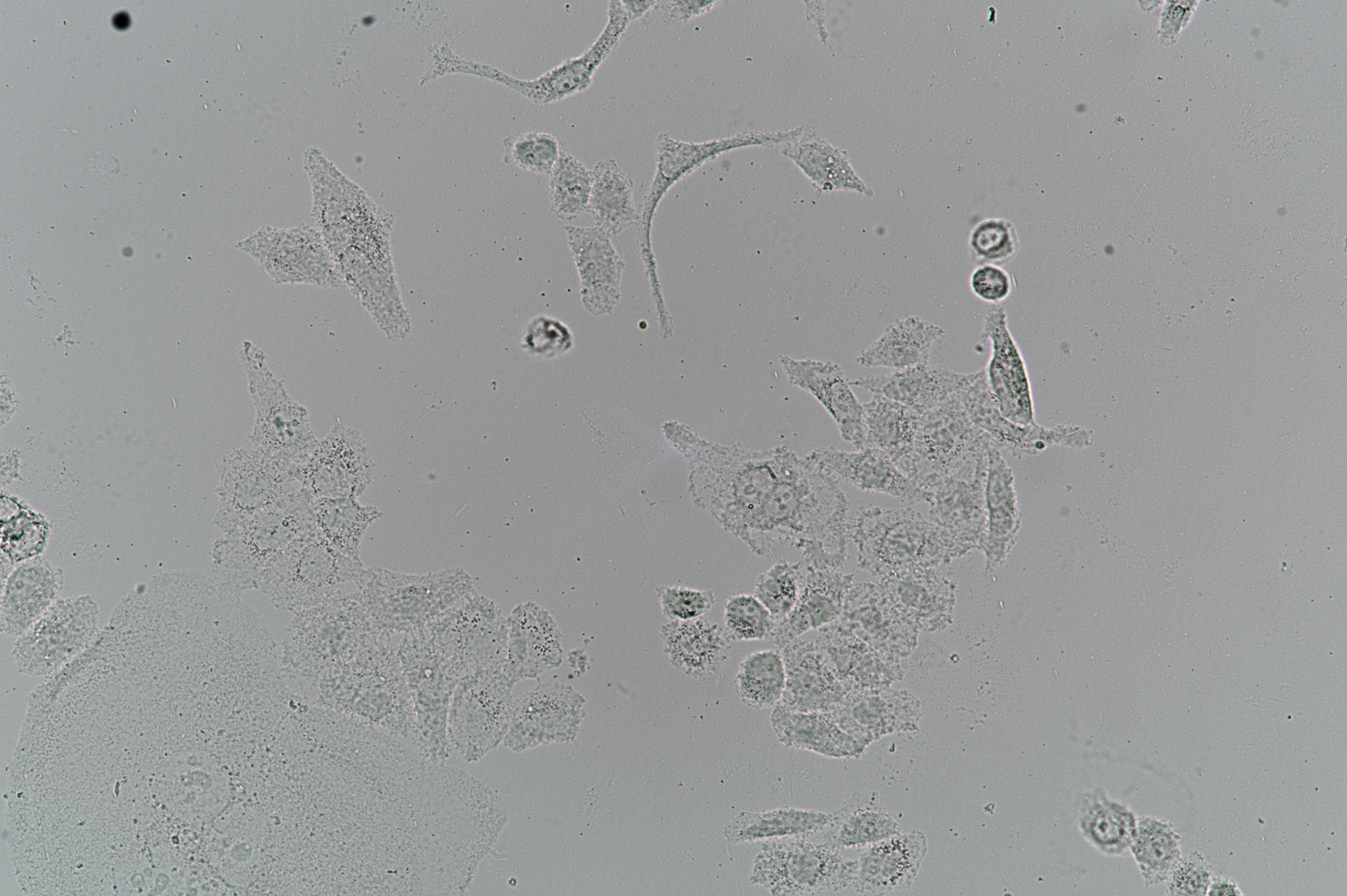

Supplement: Supplementary file 4 — Source data Fig. 2 [file 44319_2024_248_MOESM4_ESM.zip › Figure 2/Fig. 2E/DMSO 2.tif]

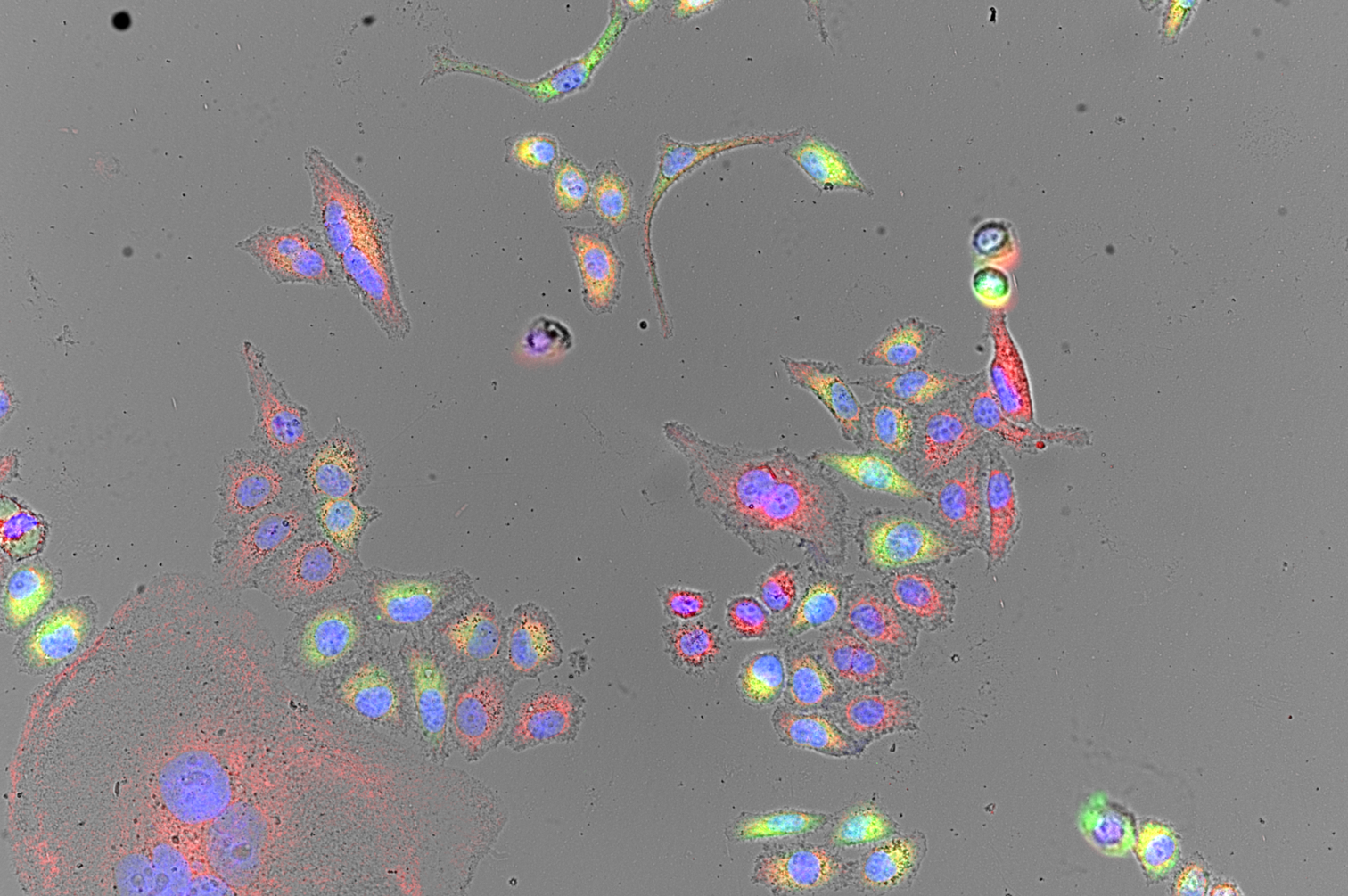

Supplement: Supplementary file 4 — Source data Fig. 2 [file 44319_2024_248_MOESM4_ESM.zip › Figure 2/Fig. 2E/DMSO 3.tif]

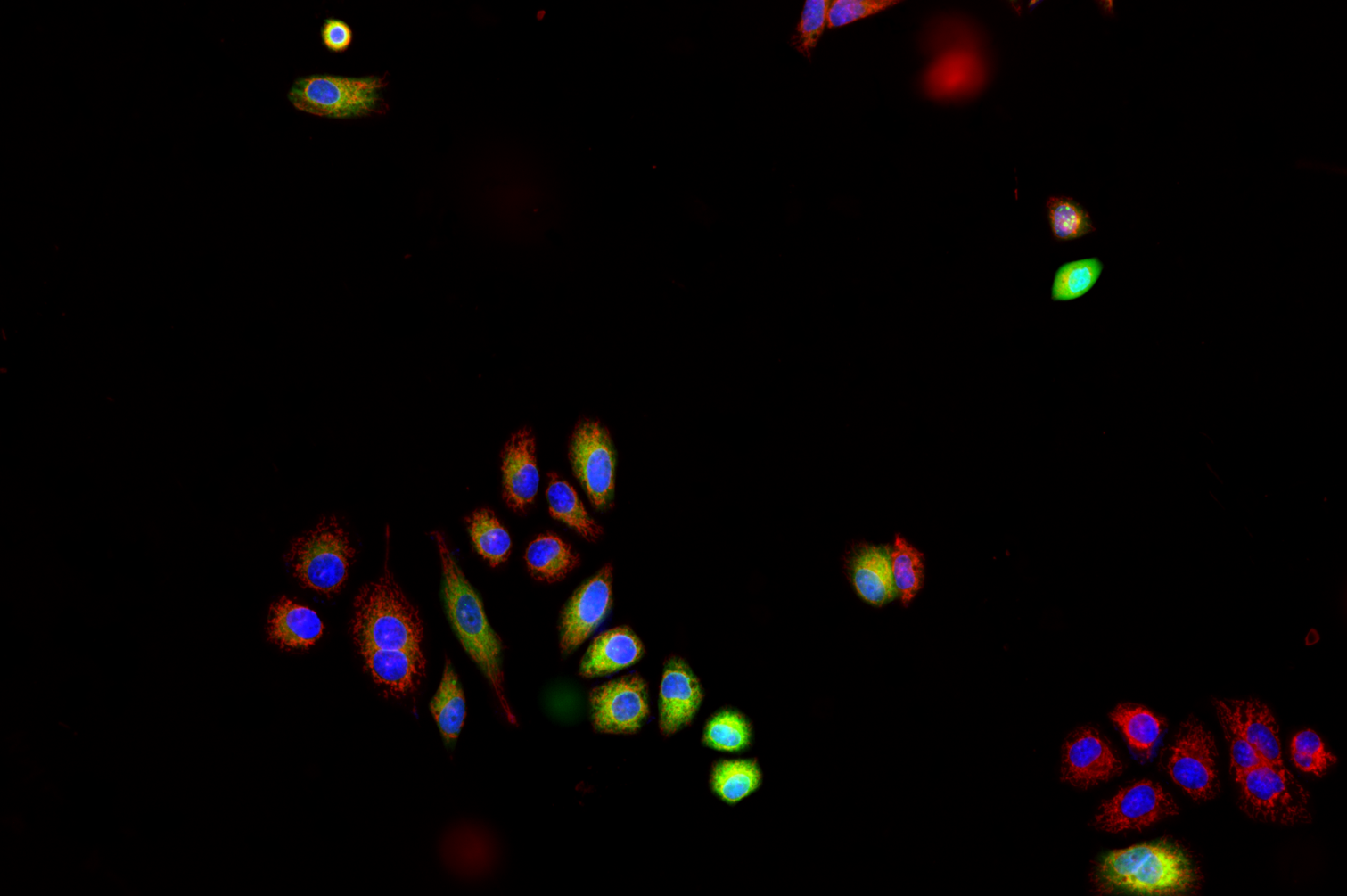

Supplement: Supplementary file 4 — Source data Fig. 2 [file 44319_2024_248_MOESM4_ESM.zip › Figure 2/Fig. 2E/KN-93 1.tif]

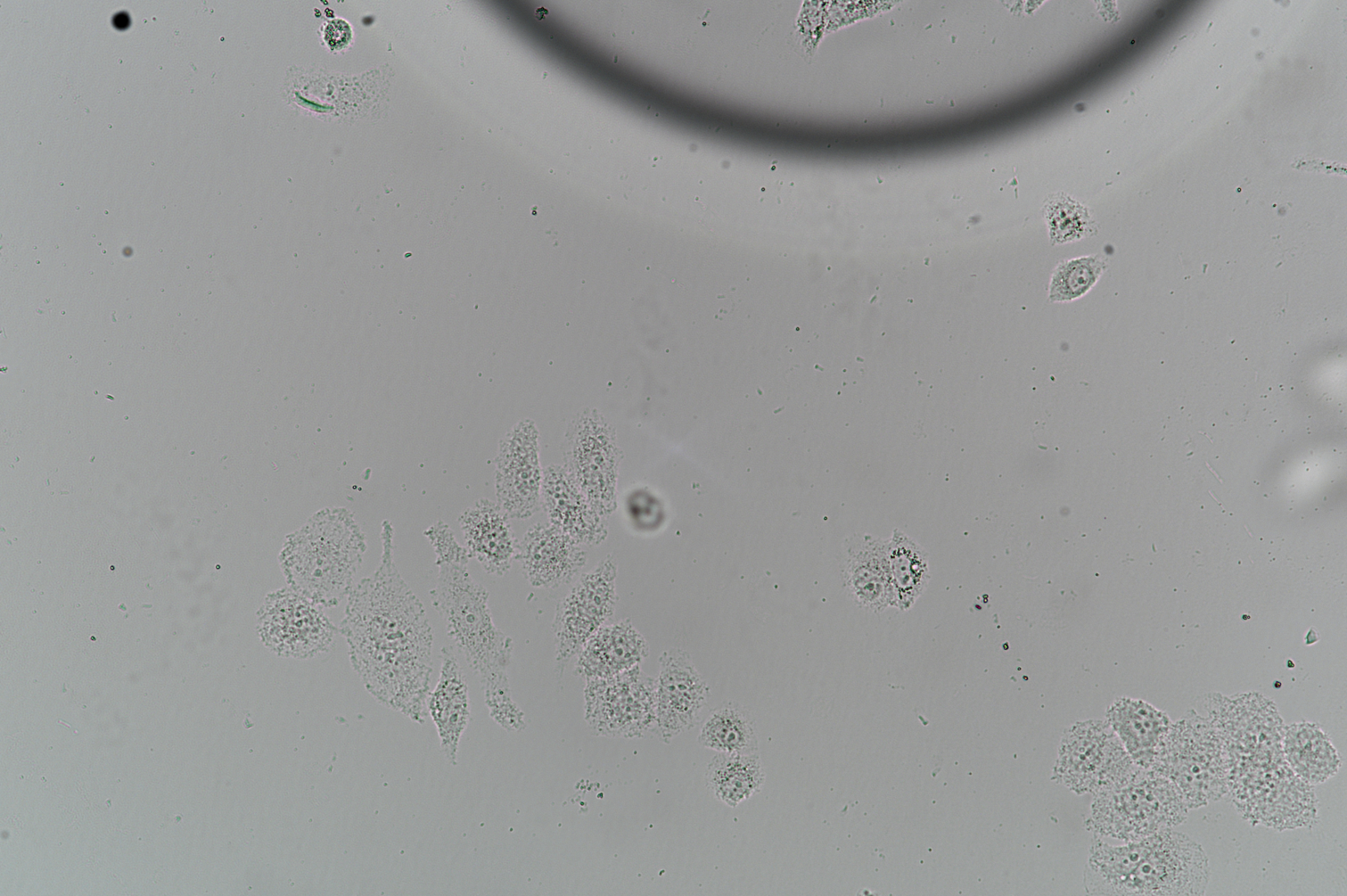

Supplement: Supplementary file 4 — Source data Fig. 2 [file 44319_2024_248_MOESM4_ESM.zip › Figure 2/Fig. 2E/KN-93 2.tif]

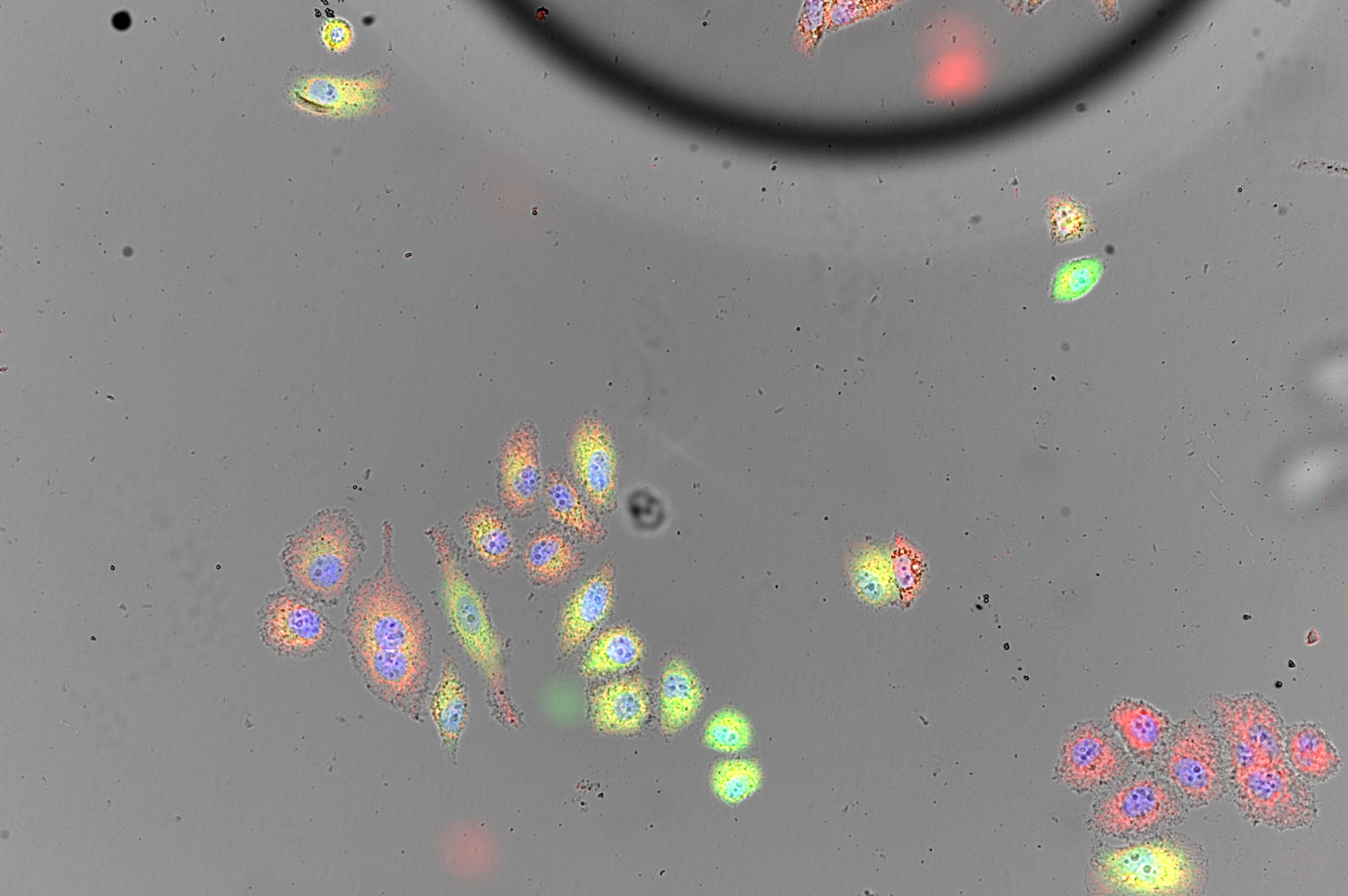

Supplement: Supplementary file 4 — Source data Fig. 2 [file 44319_2024_248_MOESM4_ESM.zip › Figure 2/Fig. 2E/KN-93 3.tif]

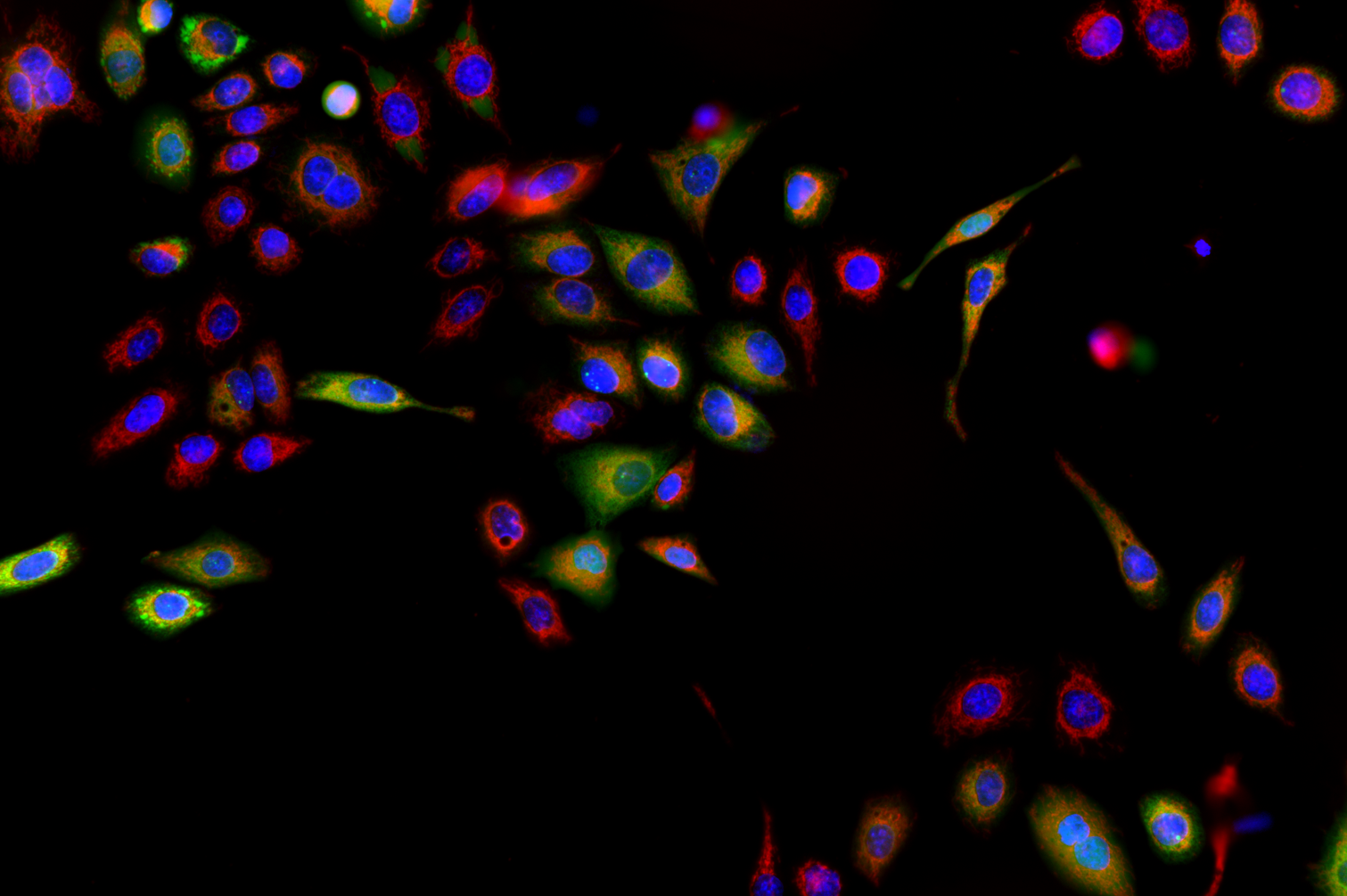

Supplement: Supplementary file 4 — Source data Fig. 2 [file 44319_2024_248_MOESM4_ESM.zip › Figure 2/Fig. 2E/MG132 1.tif]

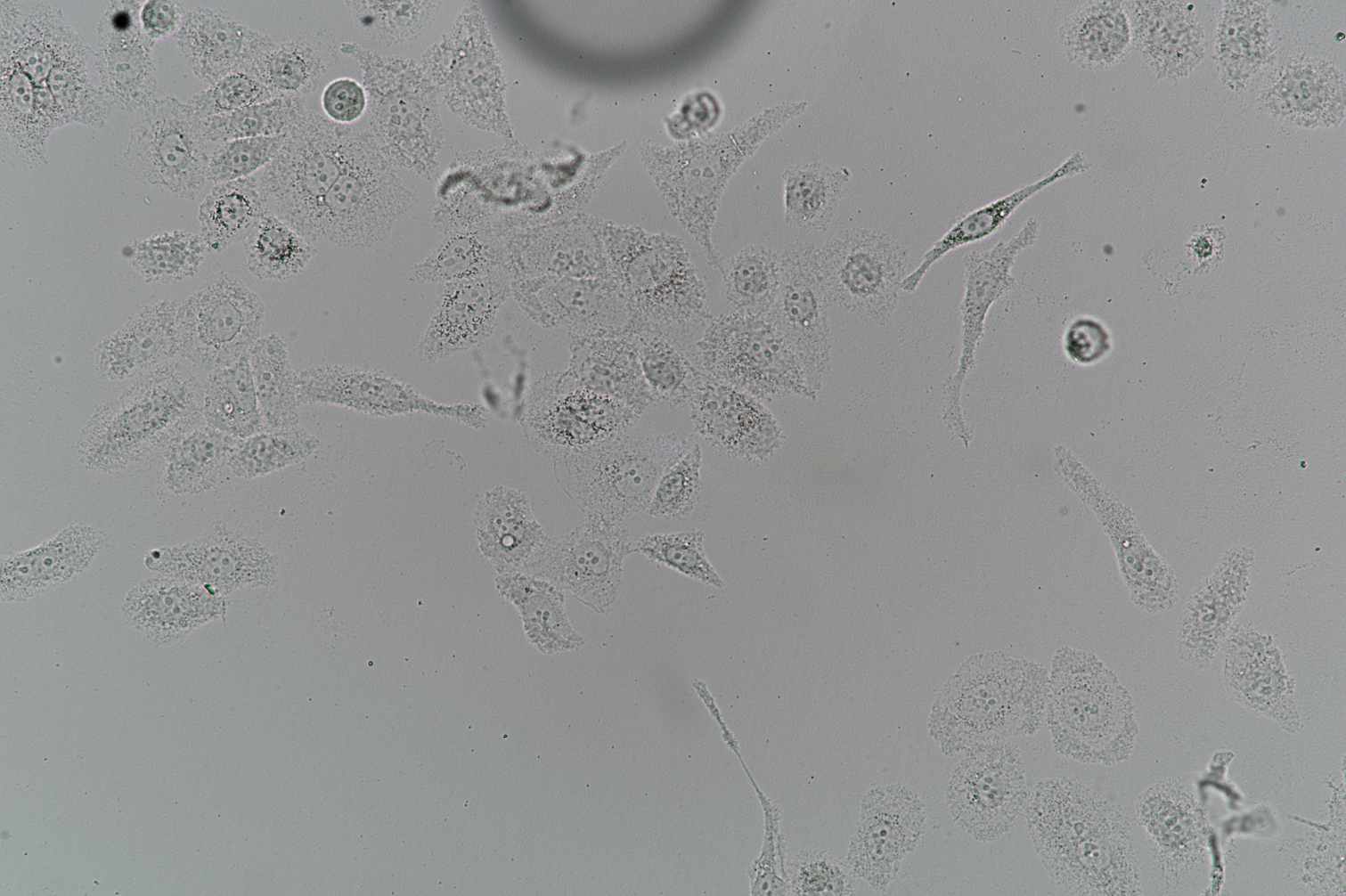

Supplement: Supplementary file 4 — Source data Fig. 2 [file 44319_2024_248_MOESM4_ESM.zip › Figure 2/Fig. 2E/MG132 2.tif]

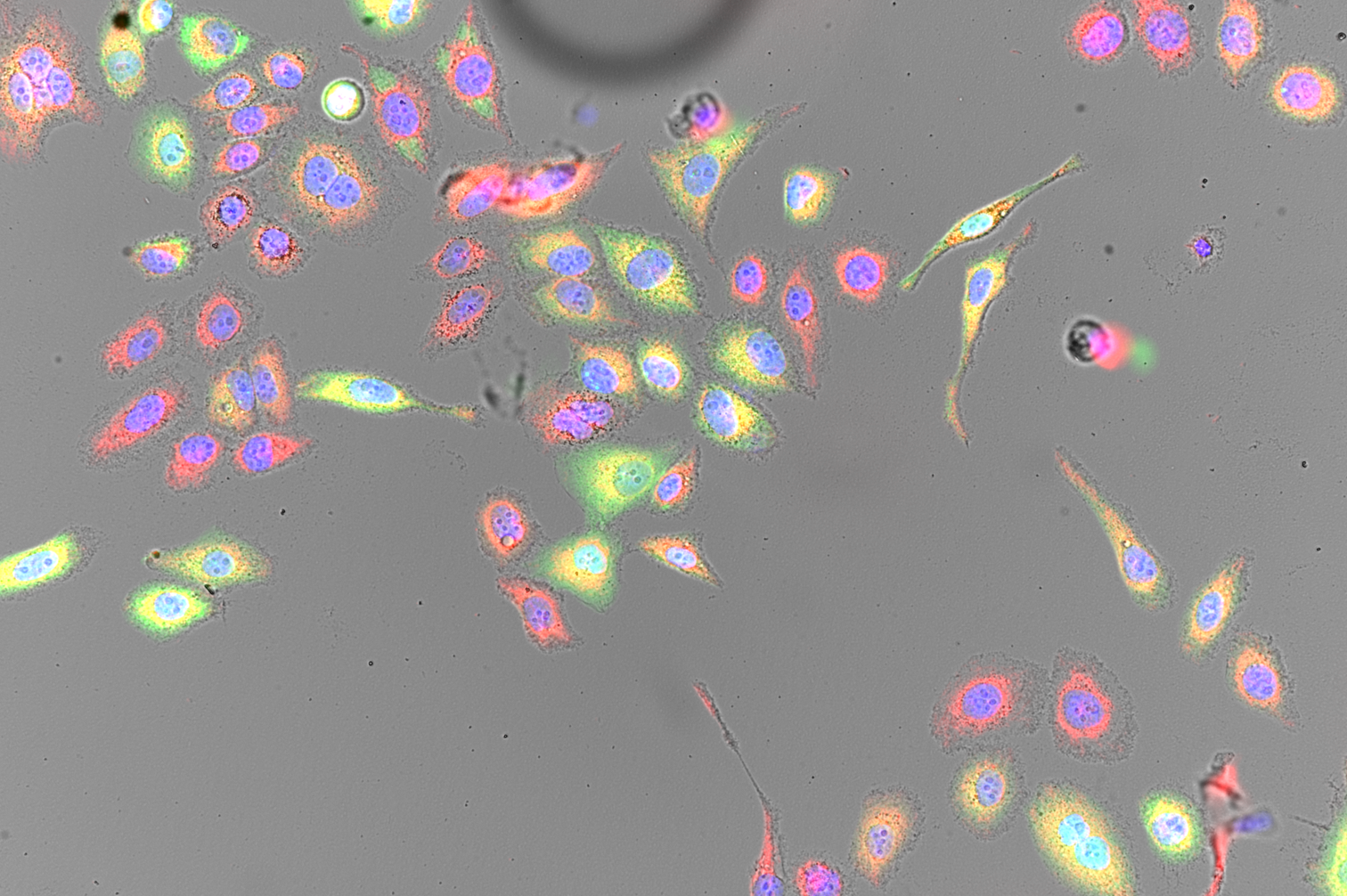

Supplement: Supplementary file 4 — Source data Fig. 2 [file 44319_2024_248_MOESM4_ESM.zip › Figure 2/Fig. 2E/MG132 3.tif]

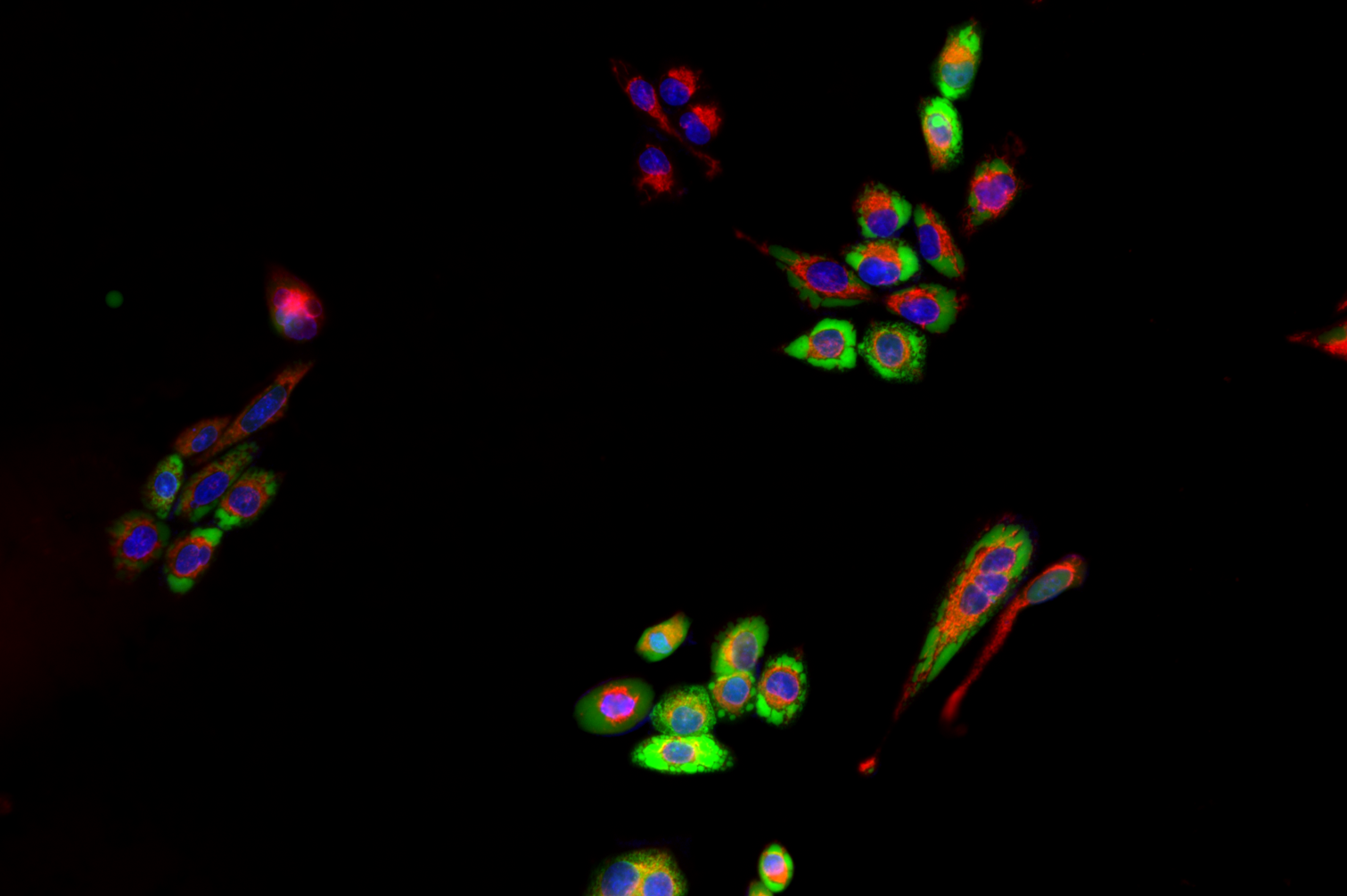

Supplement: Supplementary file 4 — Source data Fig. 2 [file 44319_2024_248_MOESM4_ESM.zip › Figure 2/Fig. 2E/MG132+KN-93 1.tif]

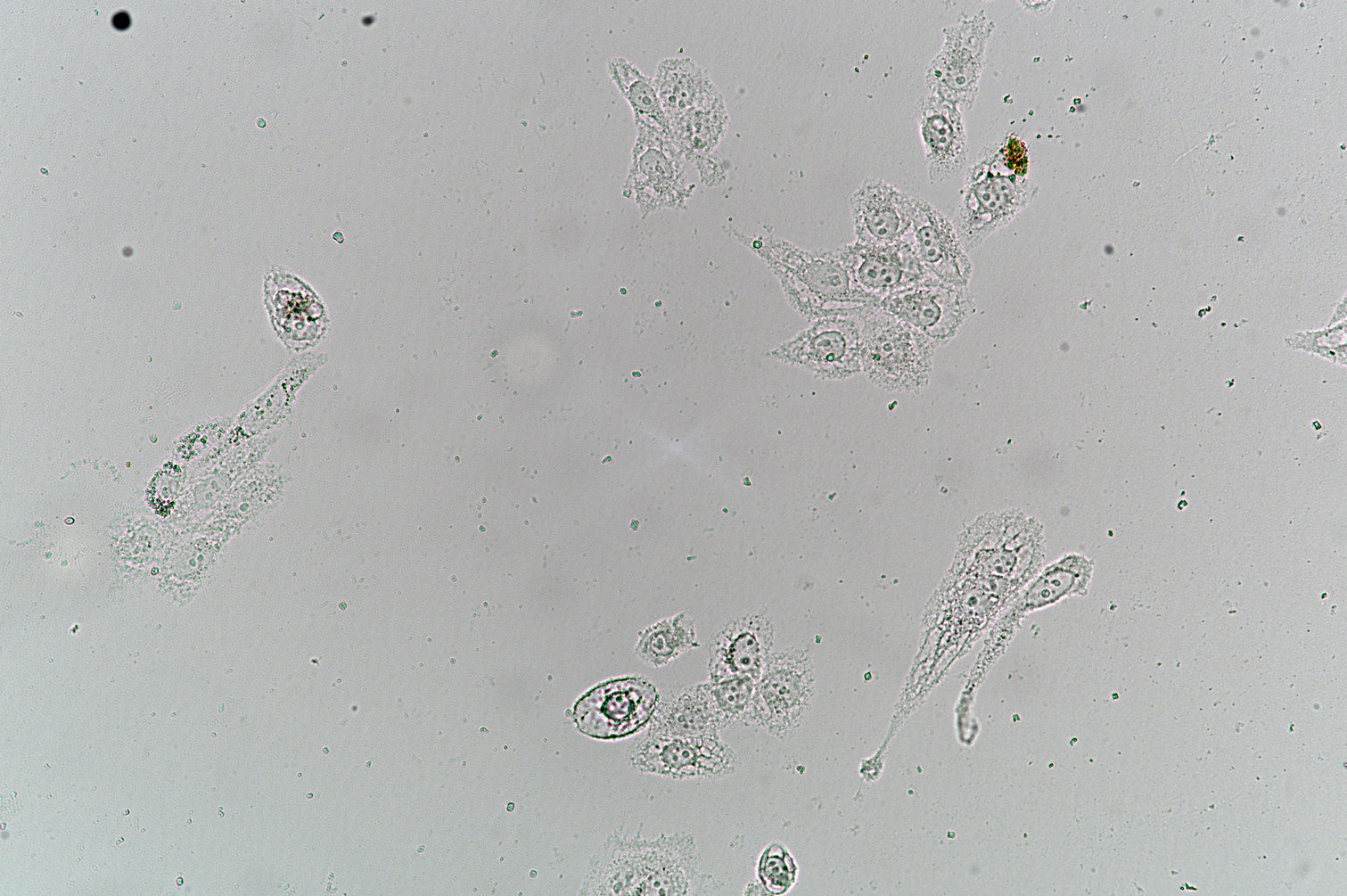

Supplement: Supplementary file 4 — Source data Fig. 2 [file 44319_2024_248_MOESM4_ESM.zip › Figure 2/Fig. 2E/MG132+KN-93 2.tif]

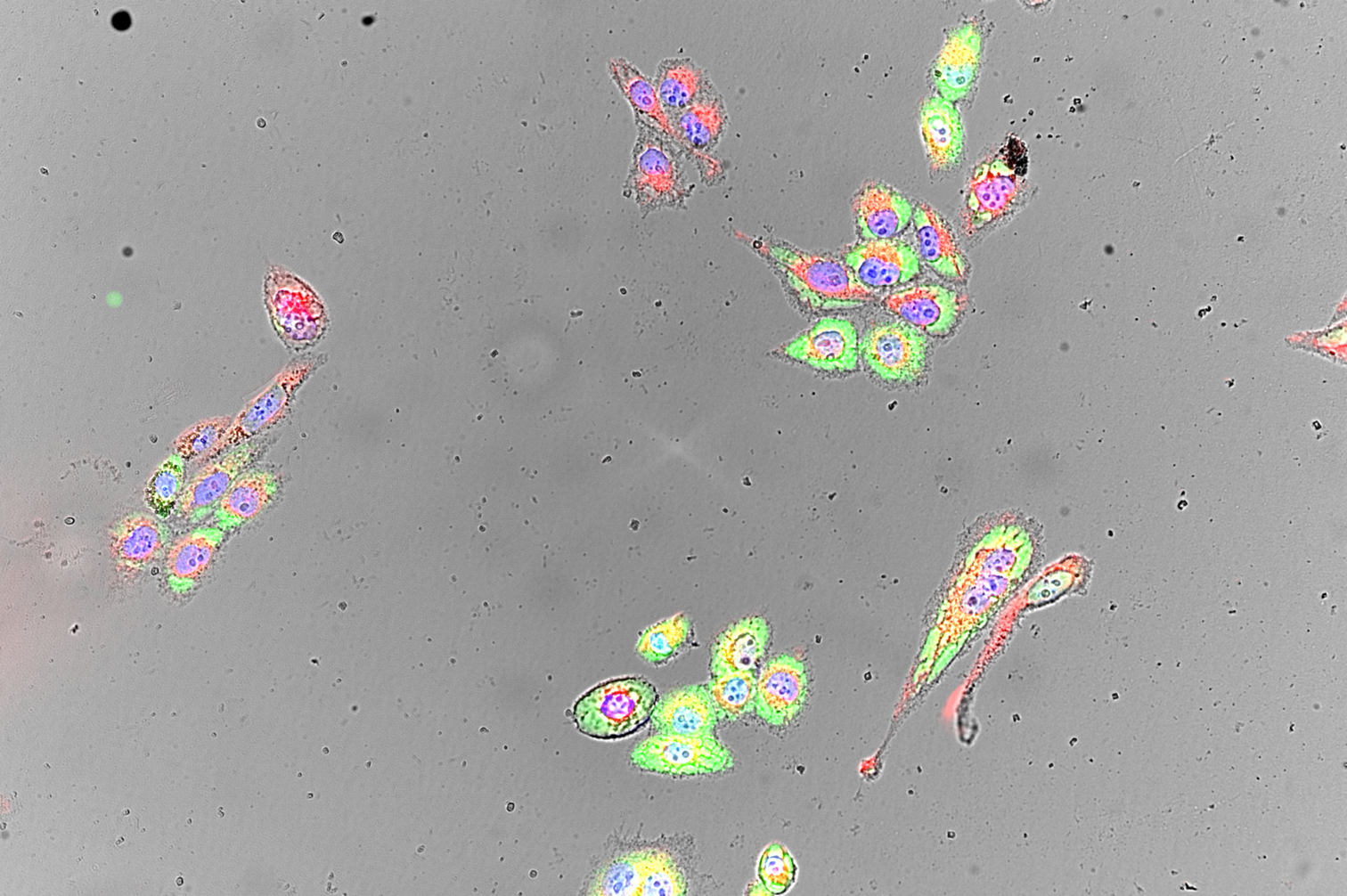

Supplement: Supplementary file 4 — Source data Fig. 2 [file 44319_2024_248_MOESM4_ESM.zip › Figure 2/Fig. 2E/MG132+KN-93 3.tif]

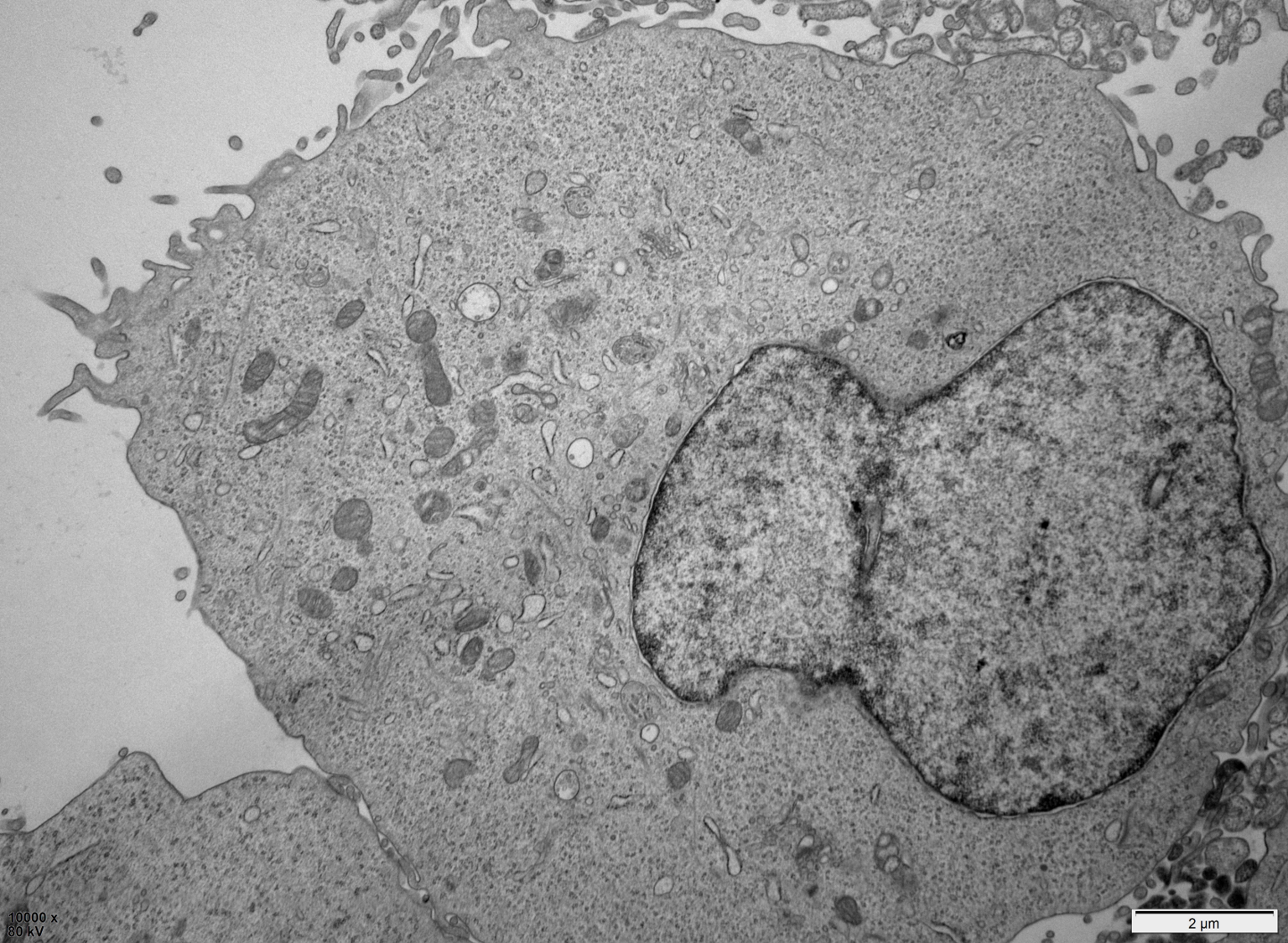

Supplement: Supplementary file 4 — Source data Fig. 2 [file 44319_2024_248_MOESM4_ESM.zip › Figure 2/Fig. 2F/DMSO-1.tif]

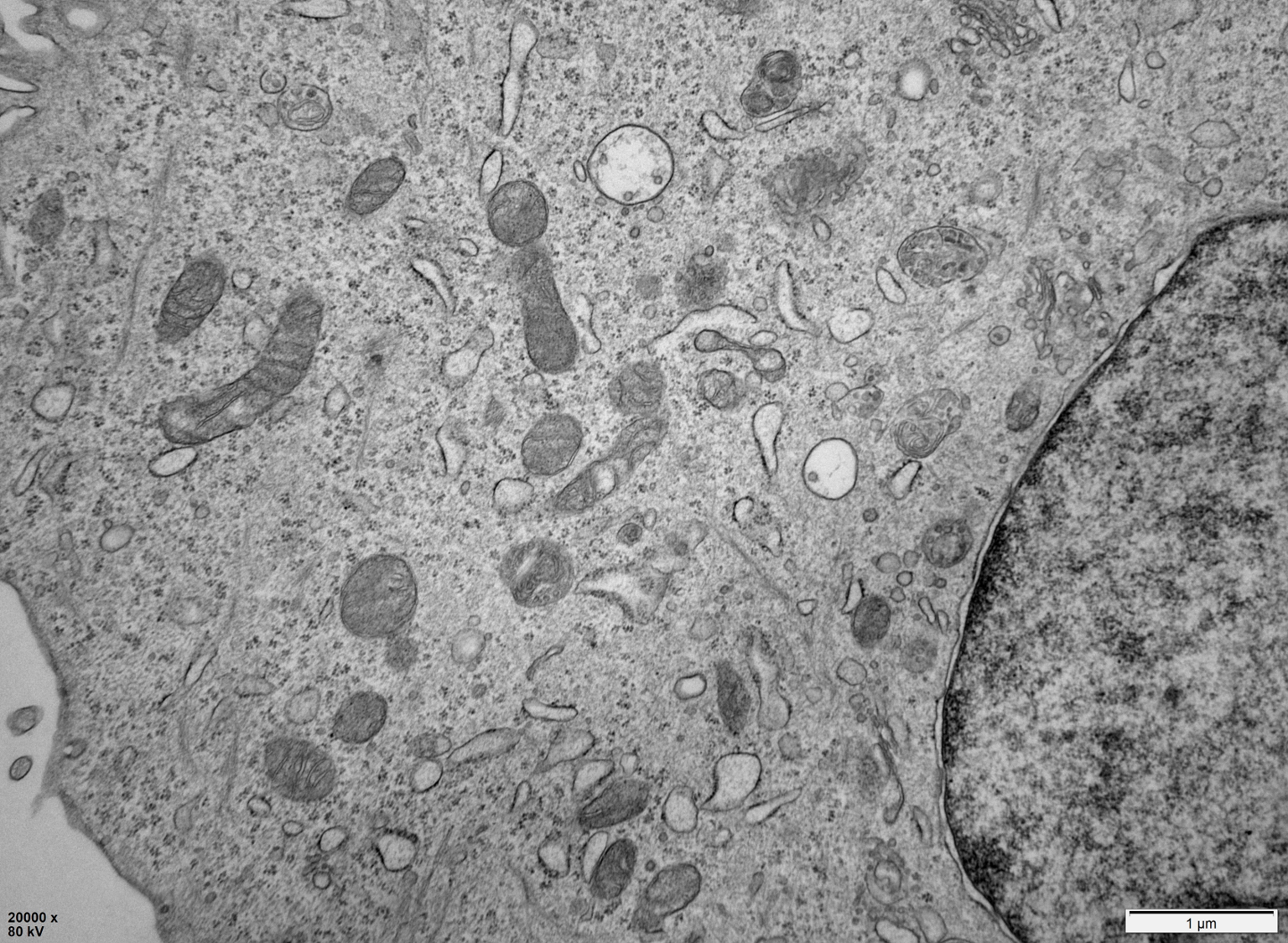

Supplement: Supplementary file 4 — Source data Fig. 2 [file 44319_2024_248_MOESM4_ESM.zip › Figure 2/Fig. 2F/DMSO-2.tif]

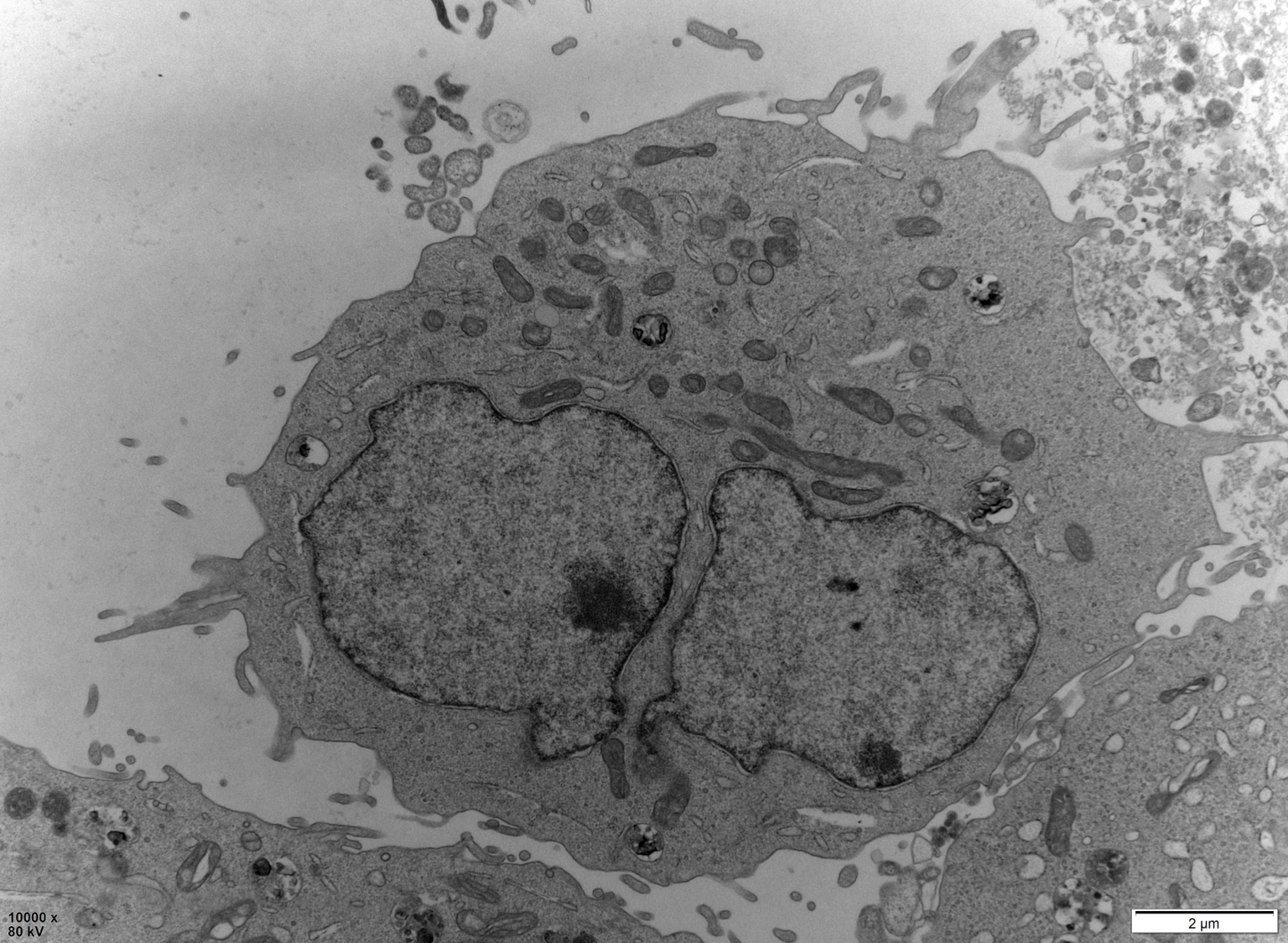

Supplement: Supplementary file 4 — Source data Fig. 2 [file 44319_2024_248_MOESM4_ESM.zip › Figure 2/Fig. 2F/KN-93-1.tif]

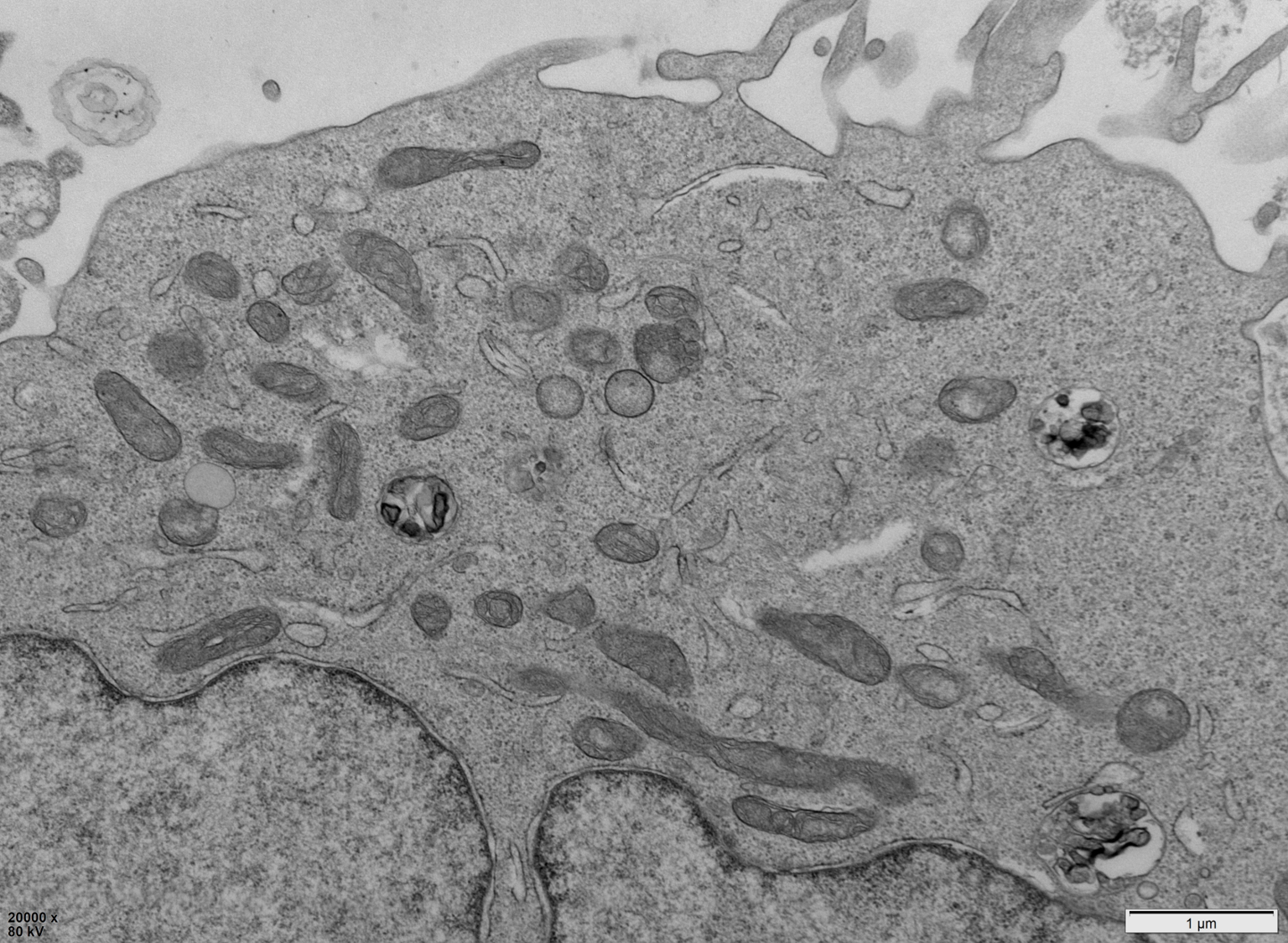

Supplement: Supplementary file 4 — Source data Fig. 2 [file 44319_2024_248_MOESM4_ESM.zip › Figure 2/Fig. 2F/KN-93-2.tif]

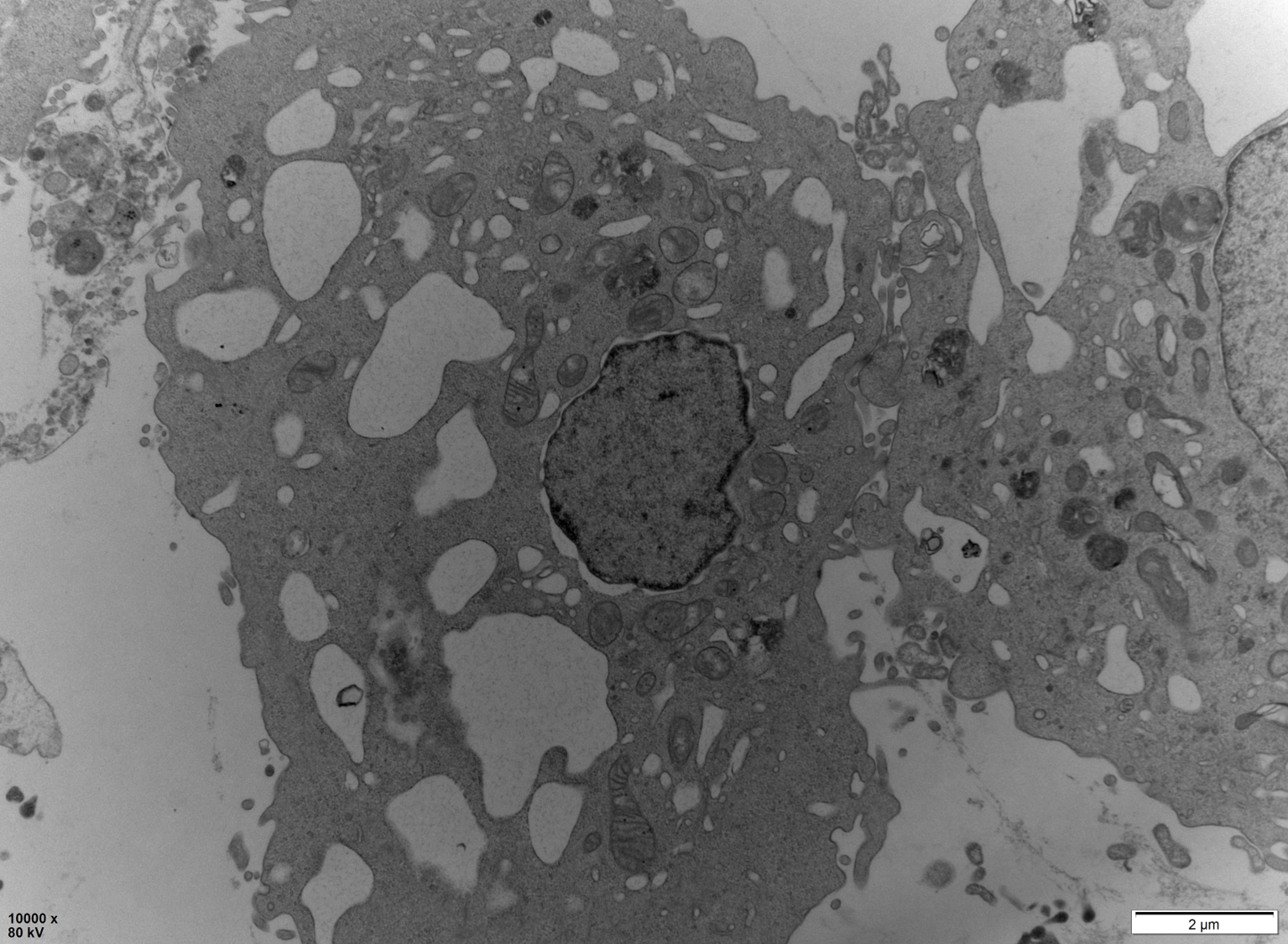

Supplement: Supplementary file 4 — Source data Fig. 2 [file 44319_2024_248_MOESM4_ESM.zip › Figure 2/Fig. 2F/MG132+KN-93 -1.tif]

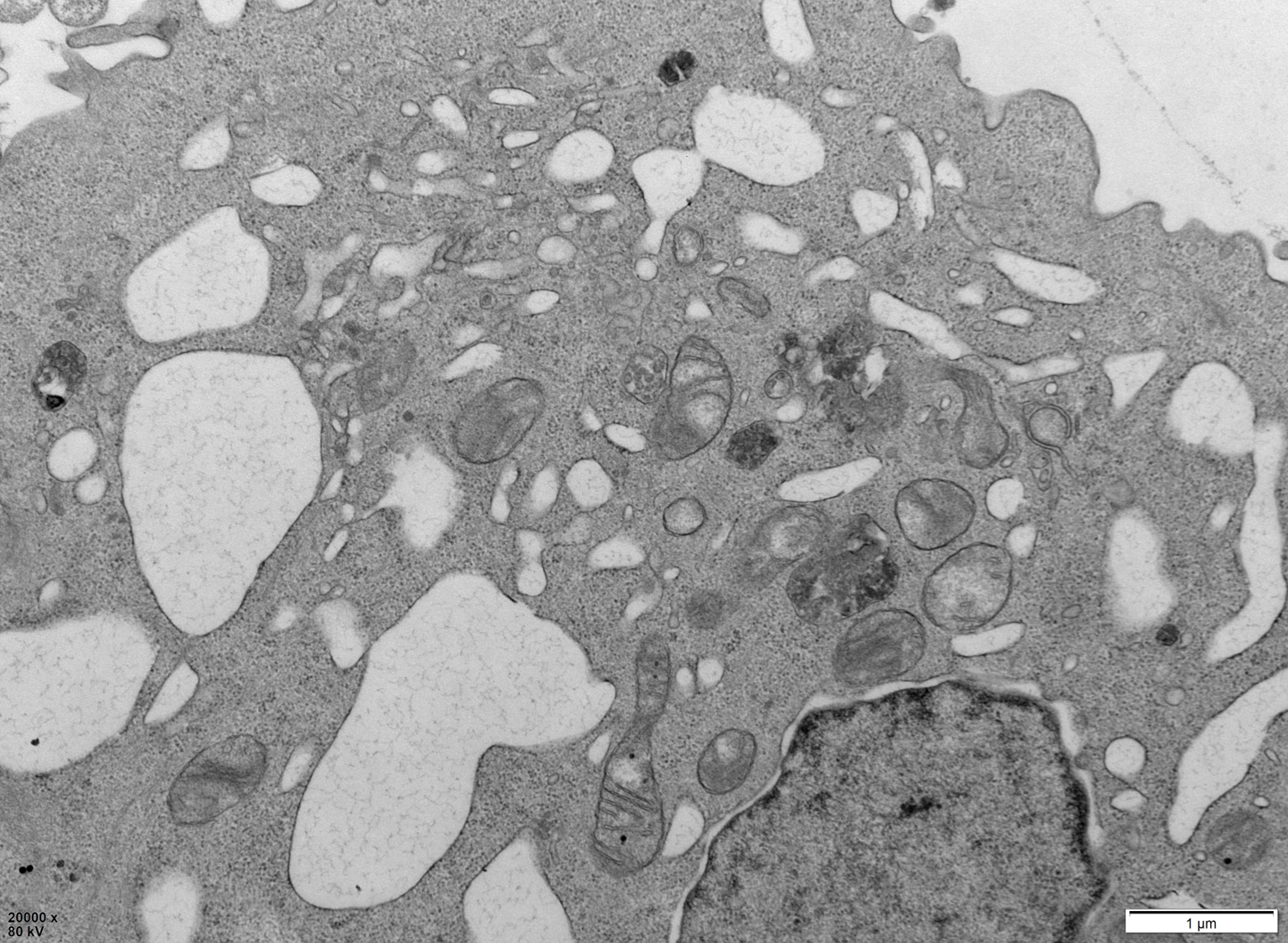

Supplement: Supplementary file 4 — Source data Fig. 2 [file 44319_2024_248_MOESM4_ESM.zip › Figure 2/Fig. 2F/MG132+KN-93 -2.tif]

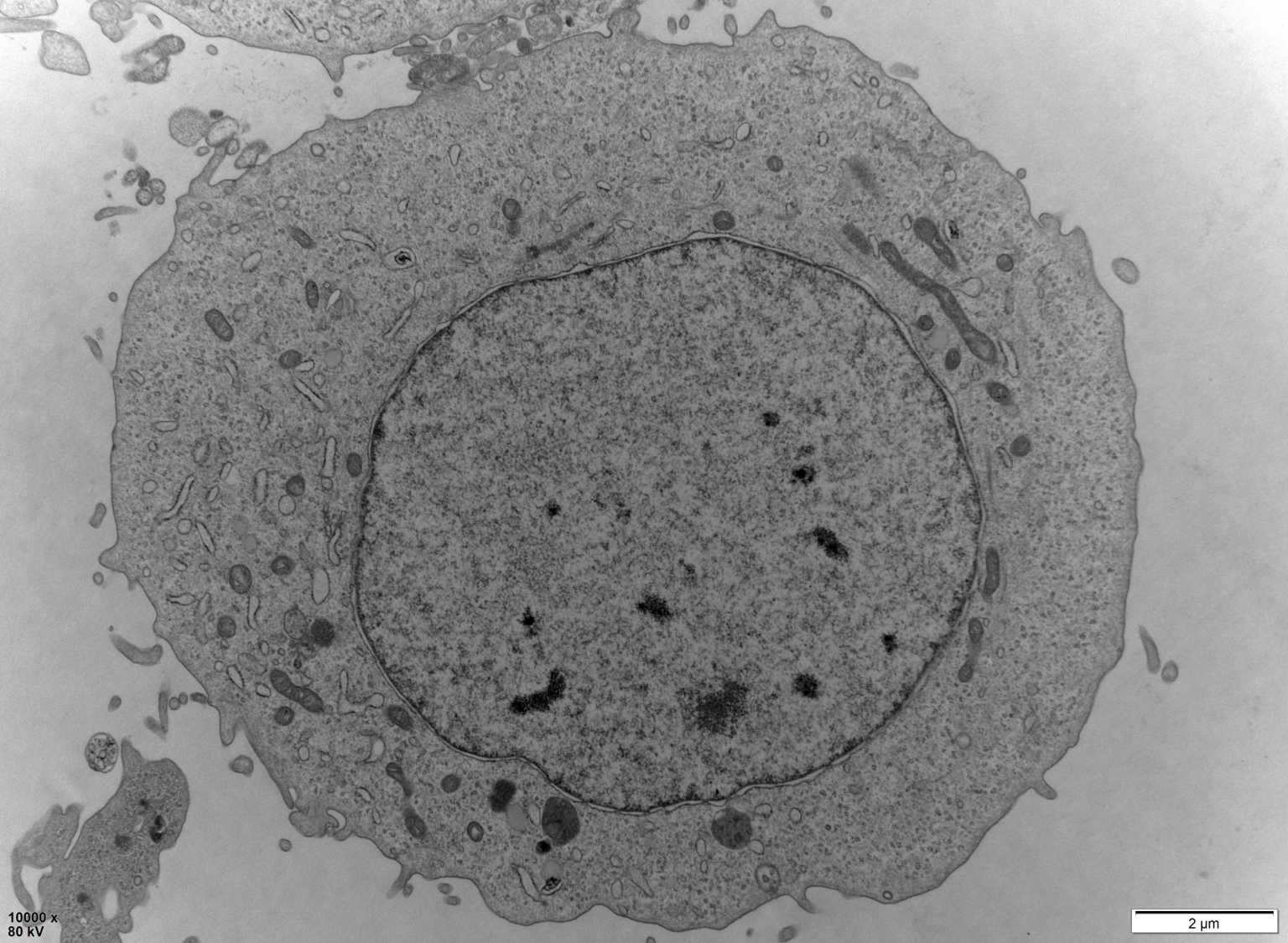

Supplement: Supplementary file 4 — Source data Fig. 2 [file 44319_2024_248_MOESM4_ESM.zip › Figure 2/Fig. 2F/MG132-1.tif]

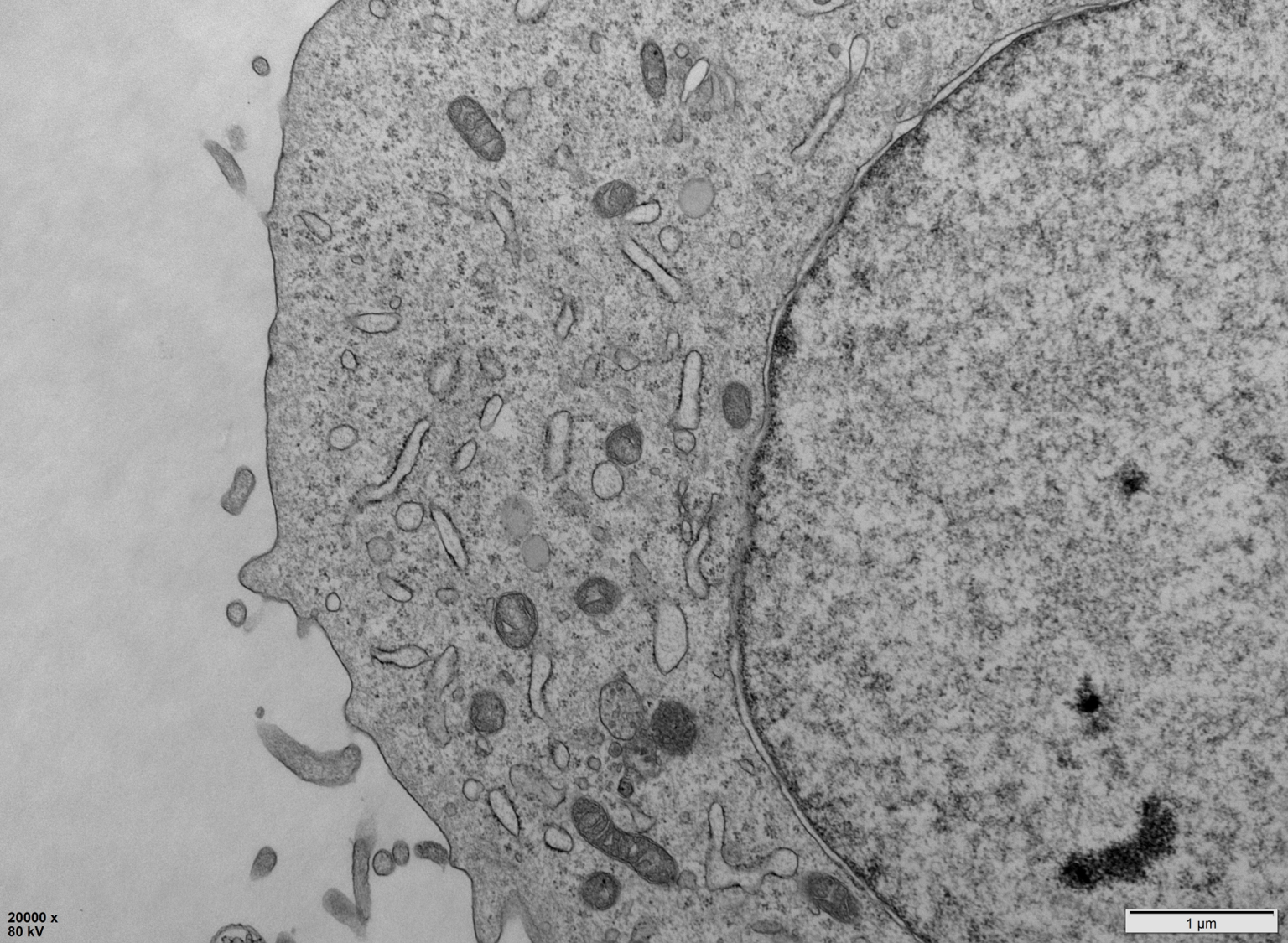

Supplement: Supplementary file 4 — Source data Fig. 2 [file 44319_2024_248_MOESM4_ESM.zip › Figure 2/Fig. 2F/MG132-2.tif]

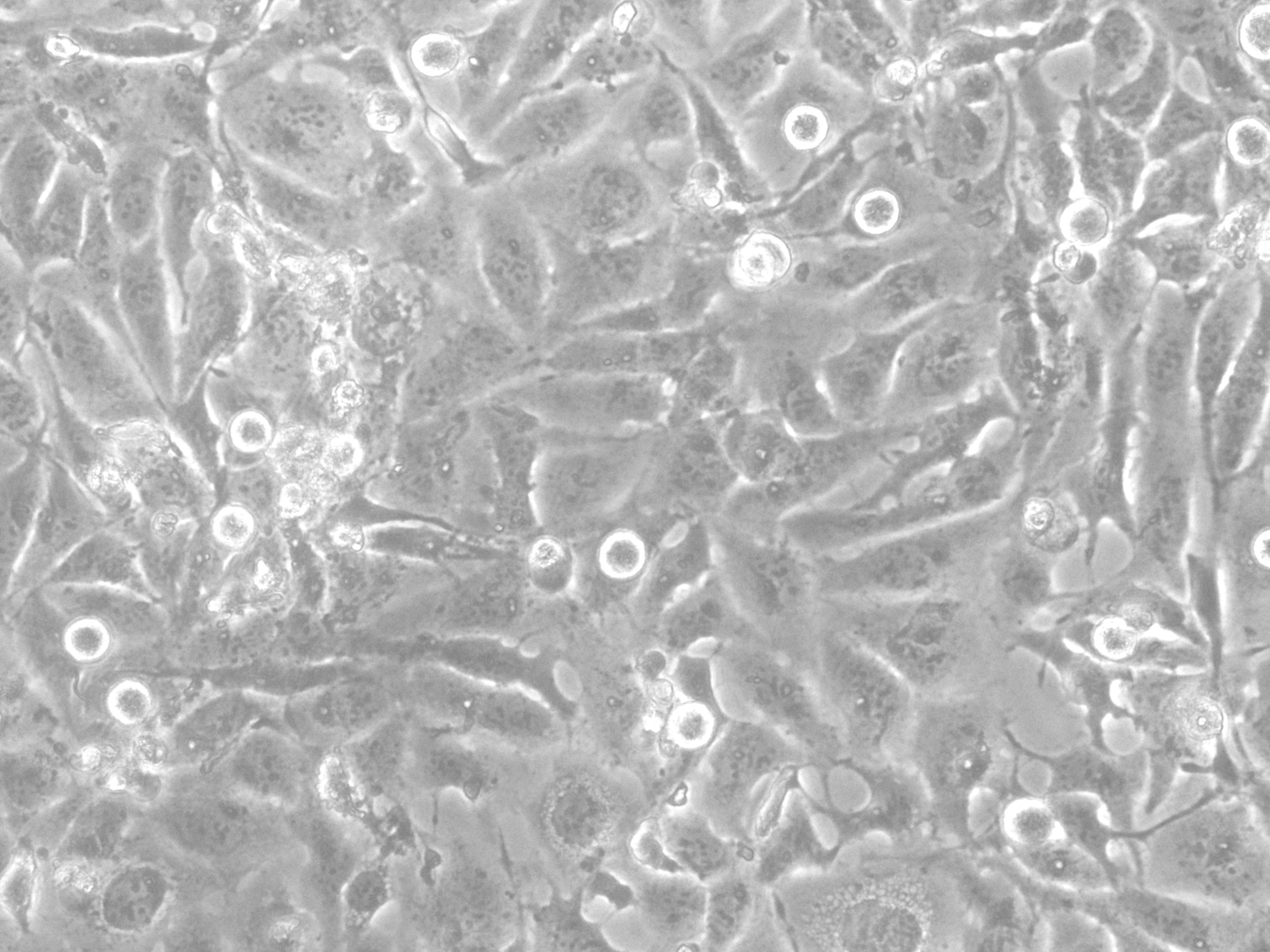

Supplement: Supplementary file 4 — Source data Fig. 2 [file 44319_2024_248_MOESM4_ESM.zip › Figure 2/Fig. 2G/293-MG132.tif]

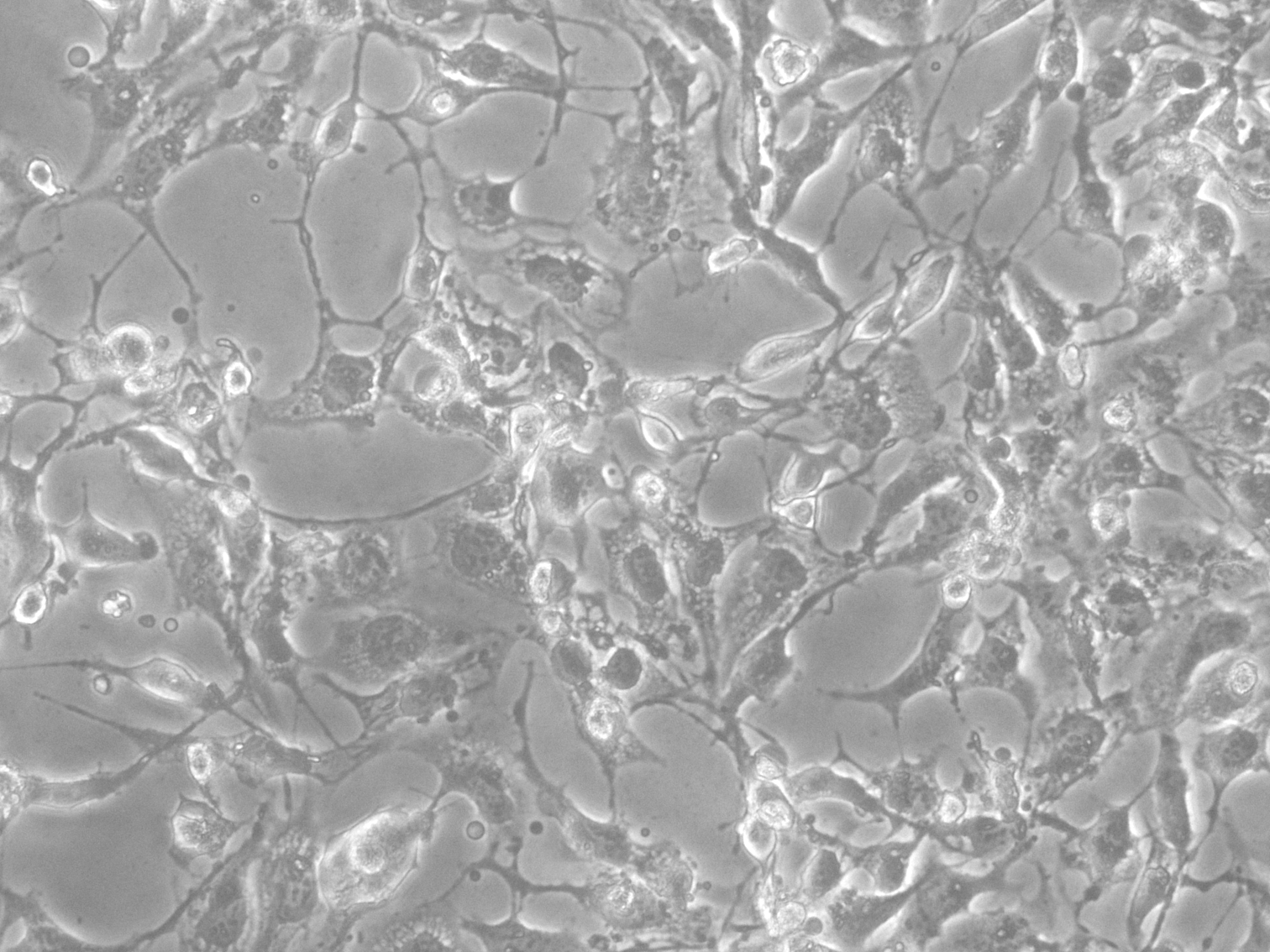

Supplement: Supplementary file 4 — Source data Fig. 2 [file 44319_2024_248_MOESM4_ESM.zip › Figure 2/Fig. 2G/293-MG132+KN-93.tif]

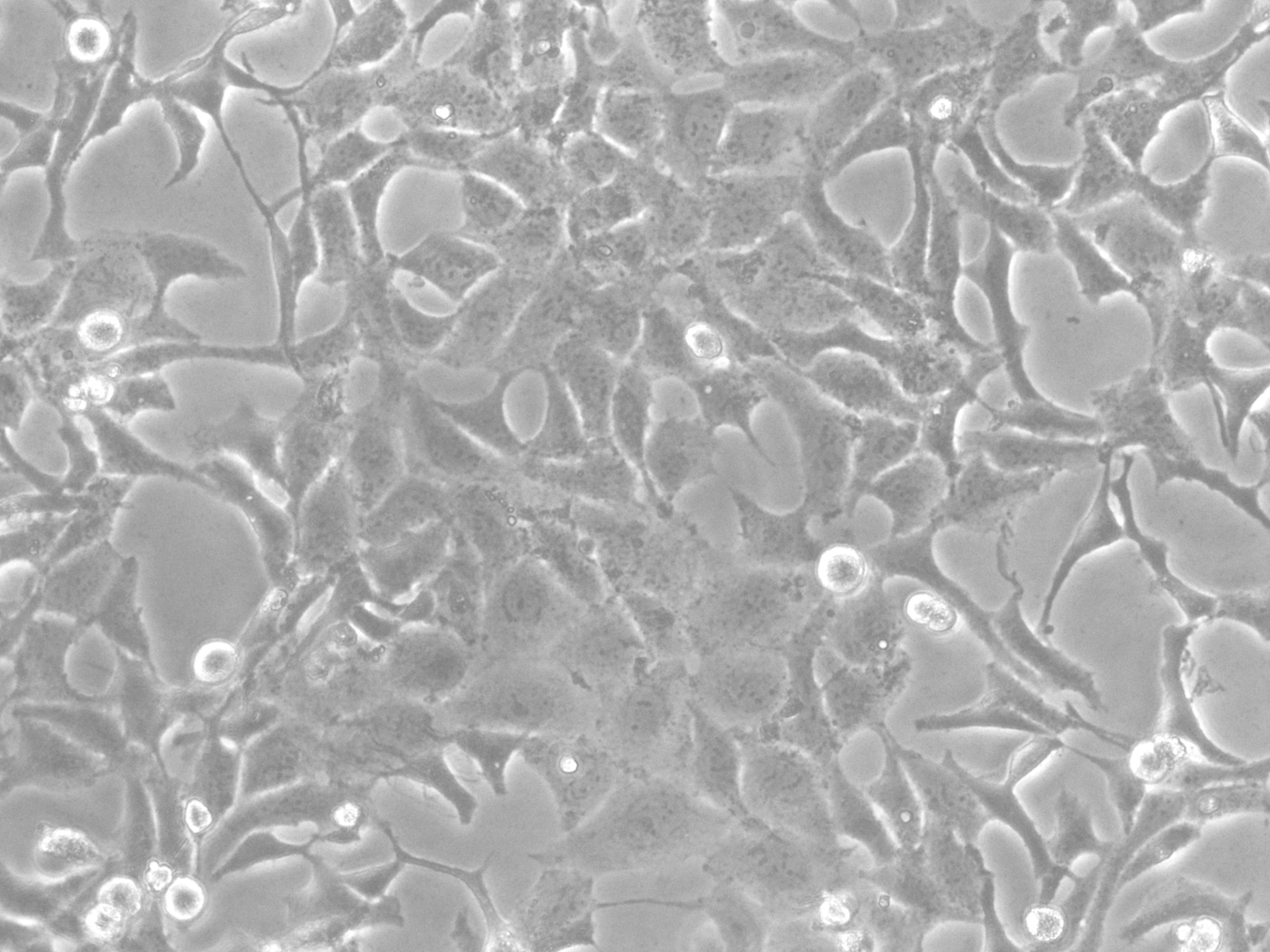

Supplement: Supplementary file 4 — Source data Fig. 2 [file 44319_2024_248_MOESM4_ESM.zip › Figure 2/Fig. 2G/293-MG132+KN-93+CHX.tif]

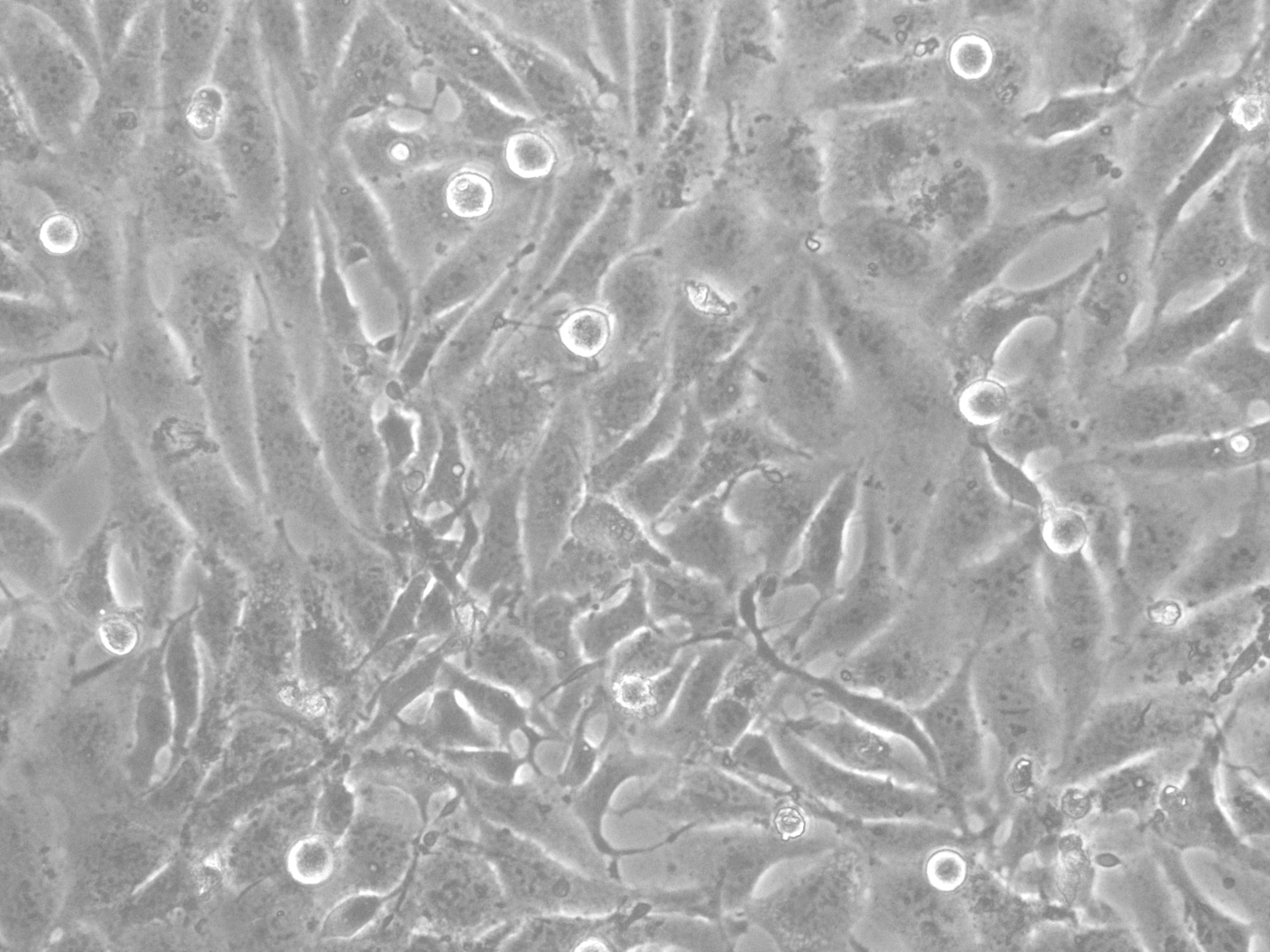

Supplement: Supplementary file 4 — Source data Fig. 2 [file 44319_2024_248_MOESM4_ESM.zip › Figure 2/Fig. 2G/293-MG132+KN-93+NAC.tif]

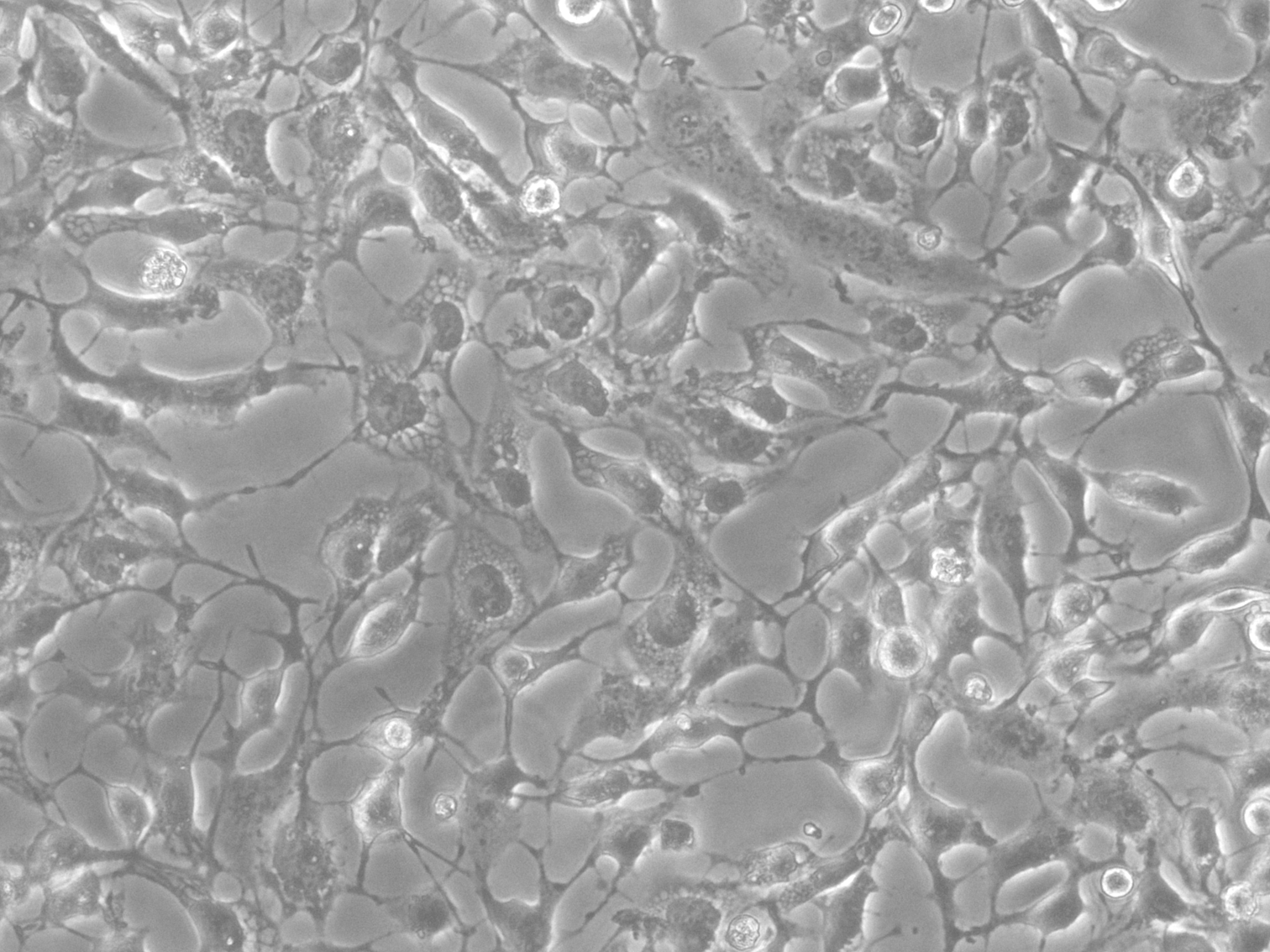

Supplement: Supplementary file 4 — Source data Fig. 2 [file 44319_2024_248_MOESM4_ESM.zip › Figure 2/Fig. 2G/293-MG132+KN-93+Z-VAD.tif]

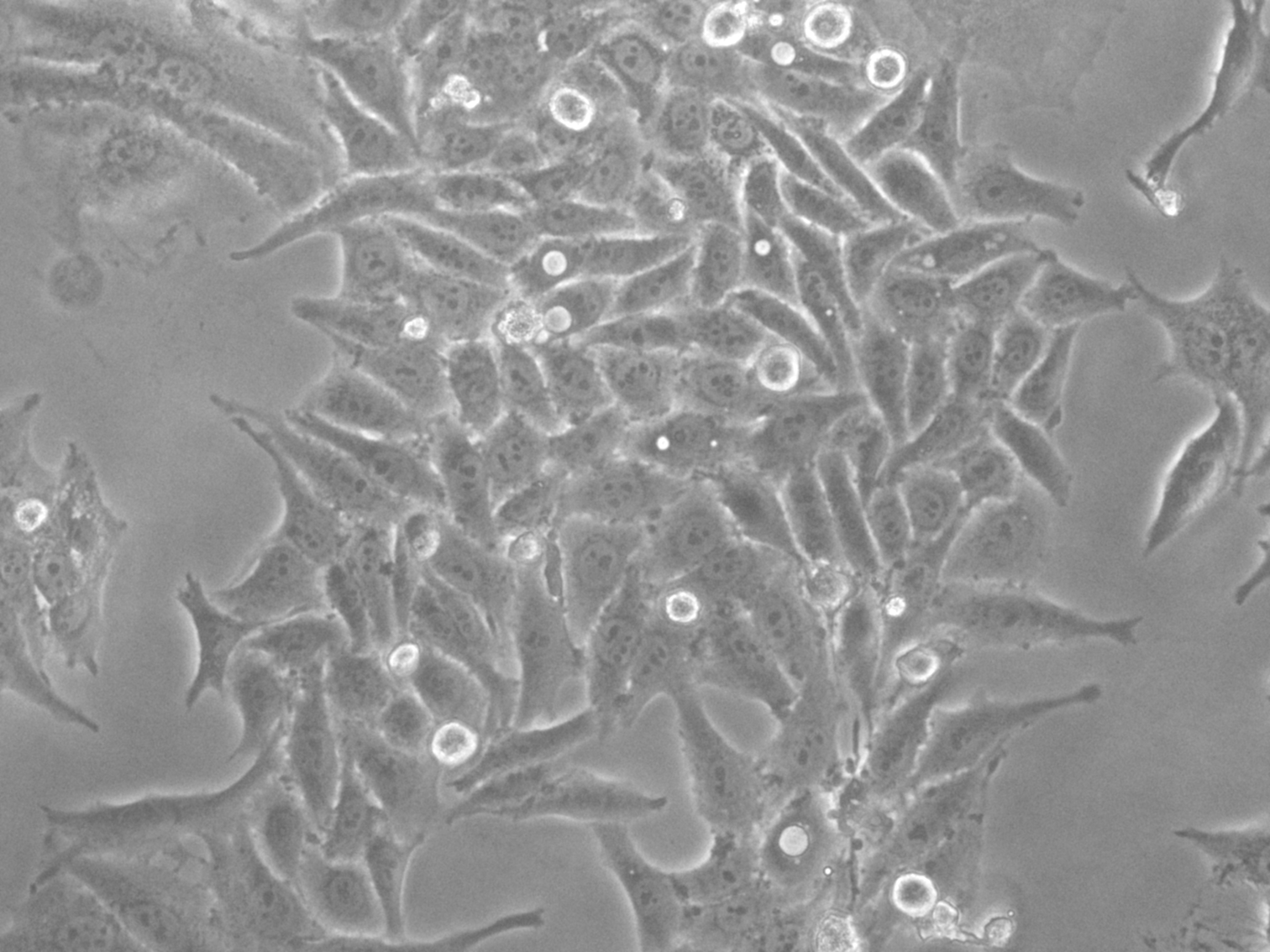

Supplement: Supplementary file 4 — Source data Fig. 2 [file 44319_2024_248_MOESM4_ESM.zip › Figure 2/Fig. 2G/Hela-MG132.tif]

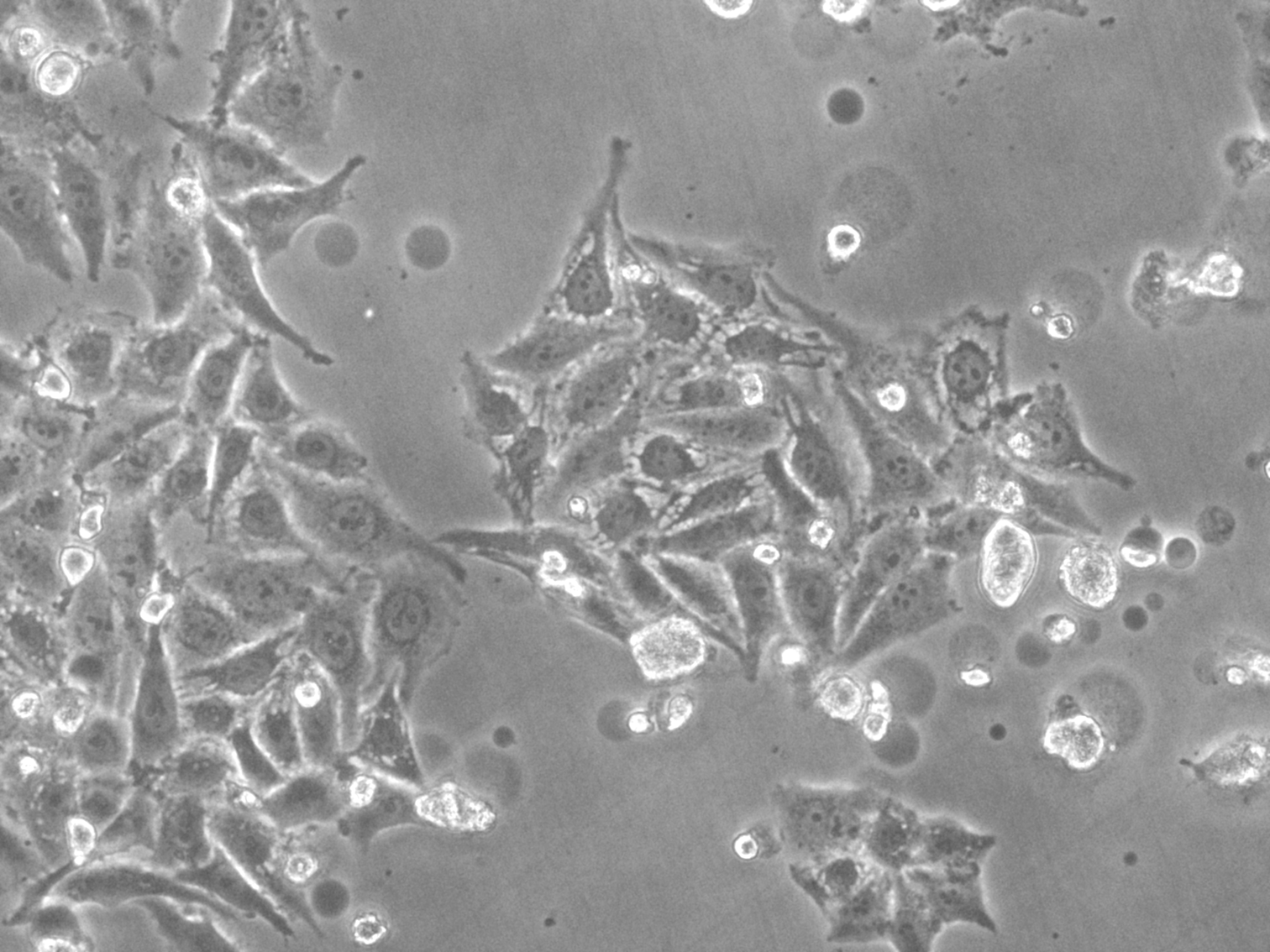

Supplement: Supplementary file 4 — Source data Fig. 2 [file 44319_2024_248_MOESM4_ESM.zip › Figure 2/Fig. 2G/Hela-MG132+KN-93.tif]

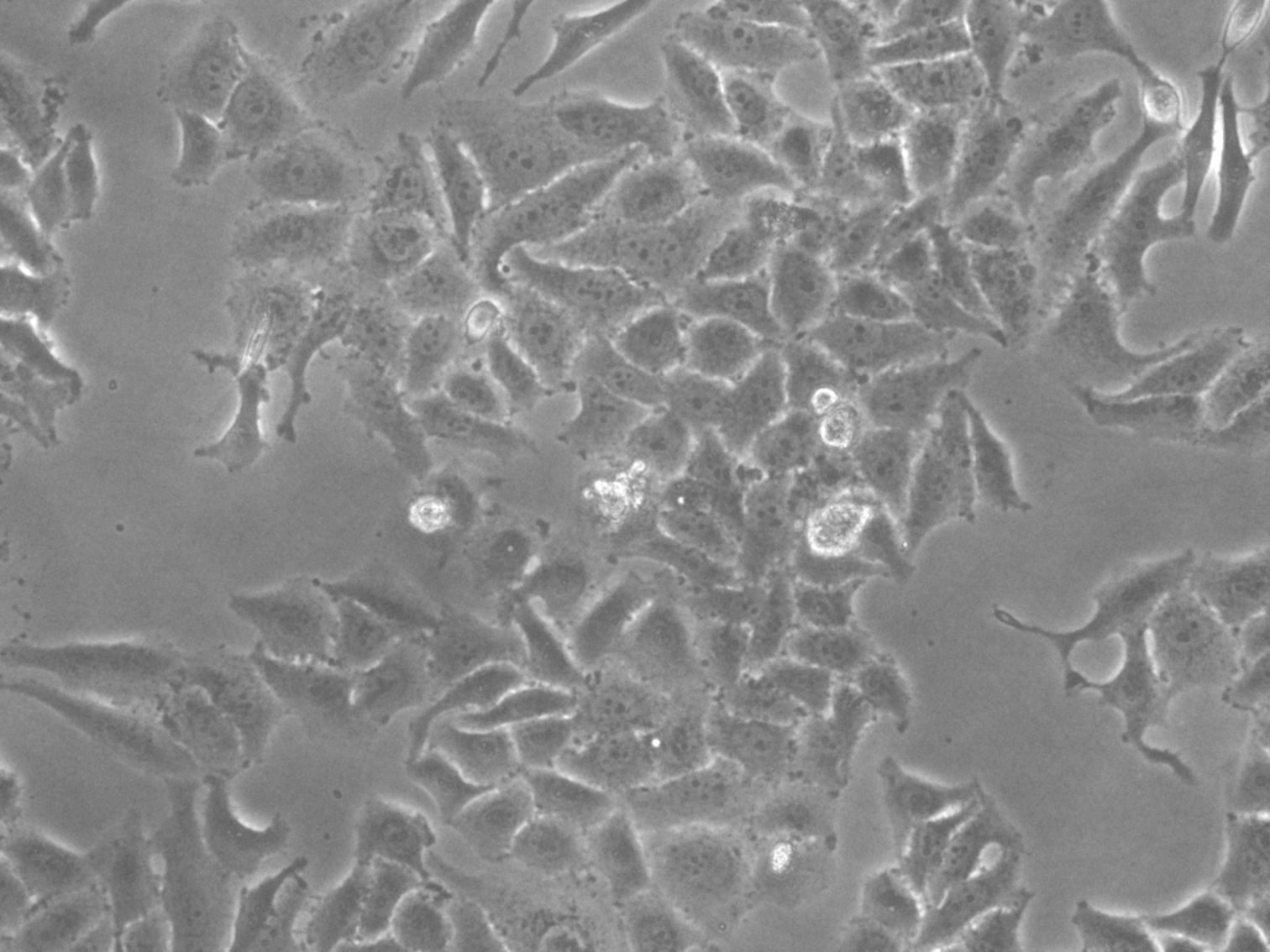

Supplement: Supplementary file 4 — Source data Fig. 2 [file 44319_2024_248_MOESM4_ESM.zip › Figure 2/Fig. 2G/Hela-MG132+KN-93+CHX.tif]

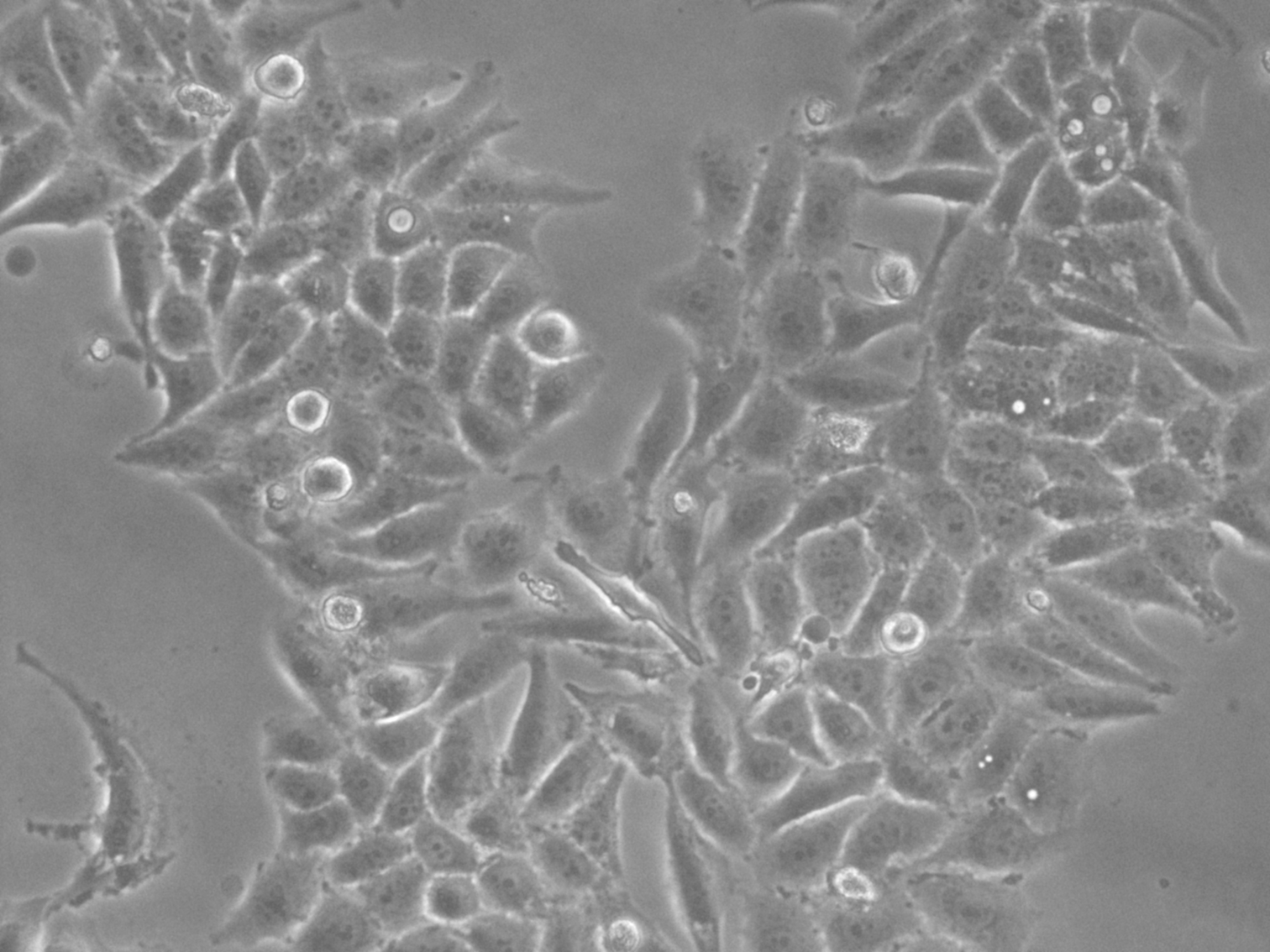

Supplement: Supplementary file 4 — Source data Fig. 2 [file 44319_2024_248_MOESM4_ESM.zip › Figure 2/Fig. 2G/Hela-MG132+KN-93+NAC.tif]

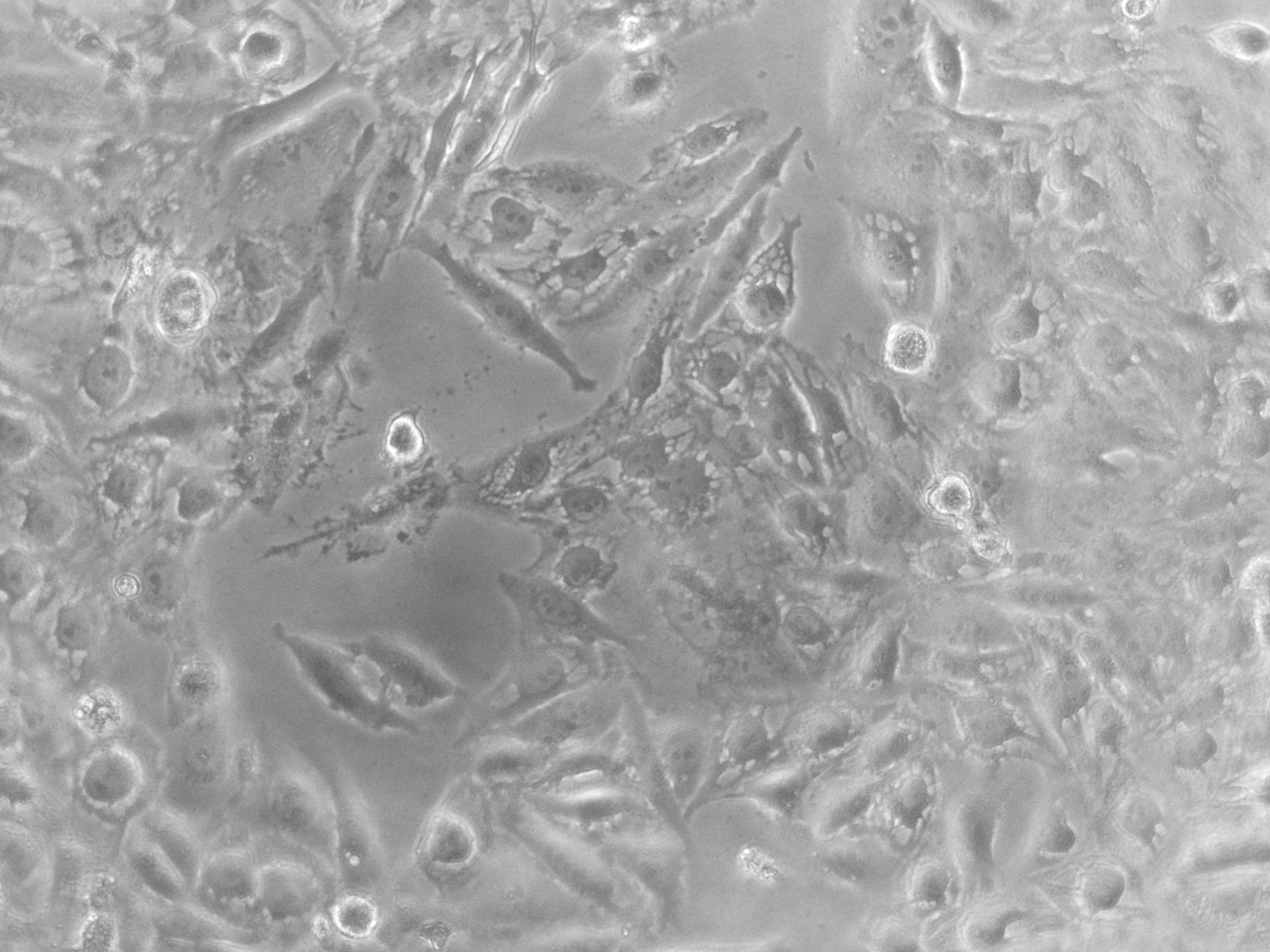

Supplement: Supplementary file 4 — Source data Fig. 2 [file 44319_2024_248_MOESM4_ESM.zip › Figure 2/Fig. 2G/Hela-MG132+KN-93+Z-VAD.tif]

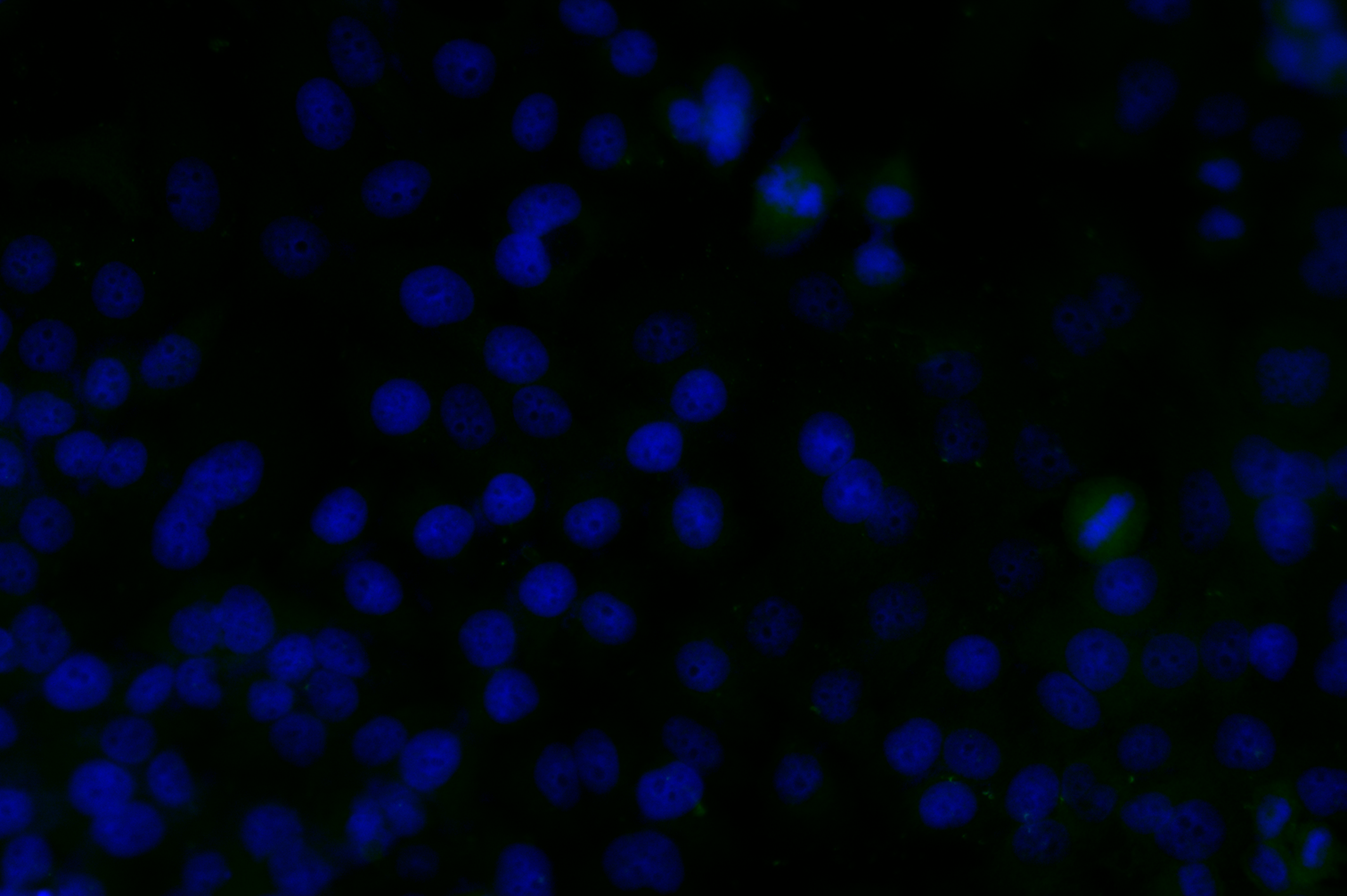

Supplement: Supplementary file 5 — Source data Fig. 3 [file 44319_2024_248_MOESM5_ESM.zip › Figure 3/Fig. 3B/DMSO.tif]

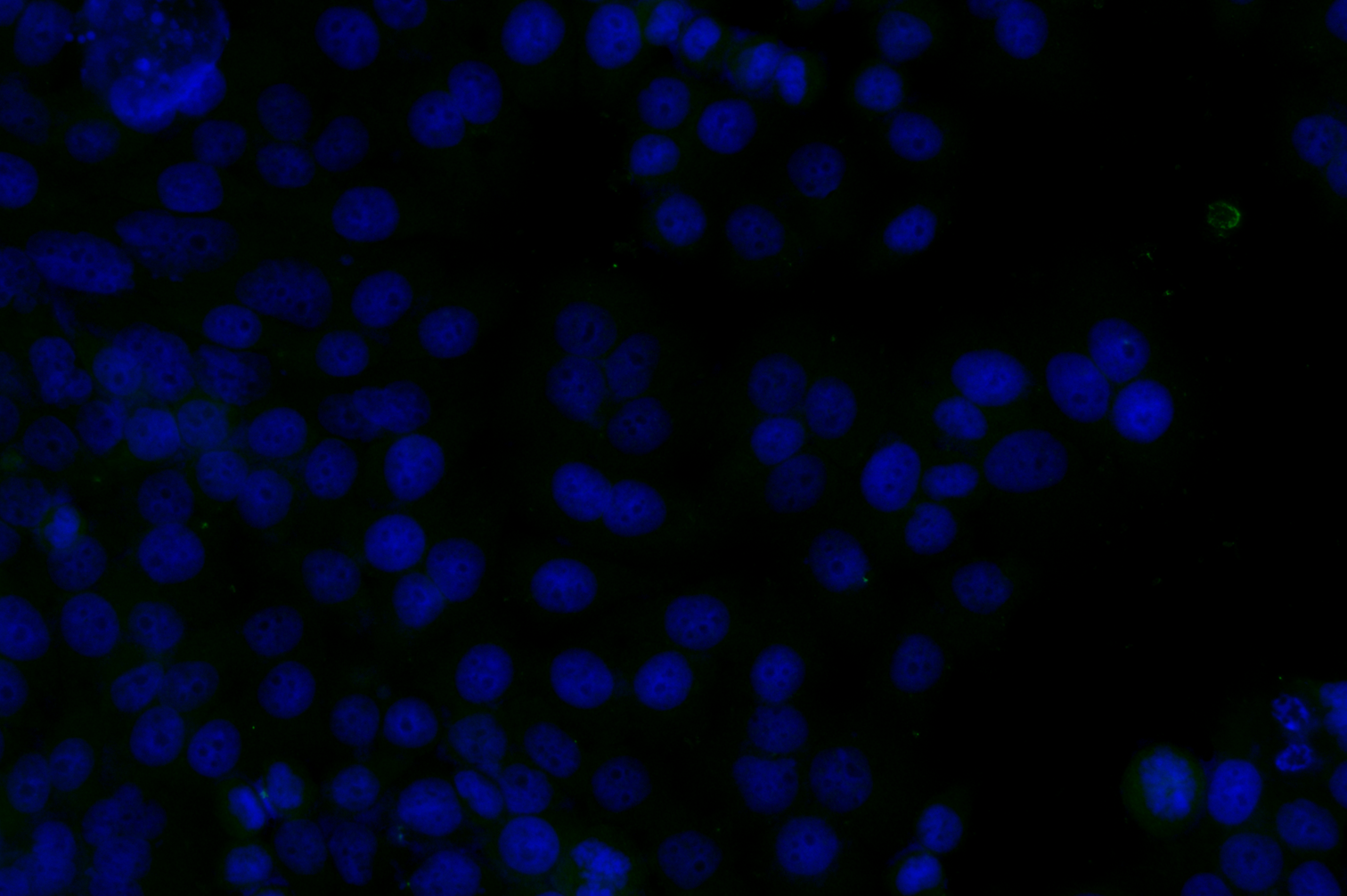

Supplement: Supplementary file 5 — Source data Fig. 3 [file 44319_2024_248_MOESM5_ESM.zip › Figure 3/Fig. 3B/KN-93.tif]

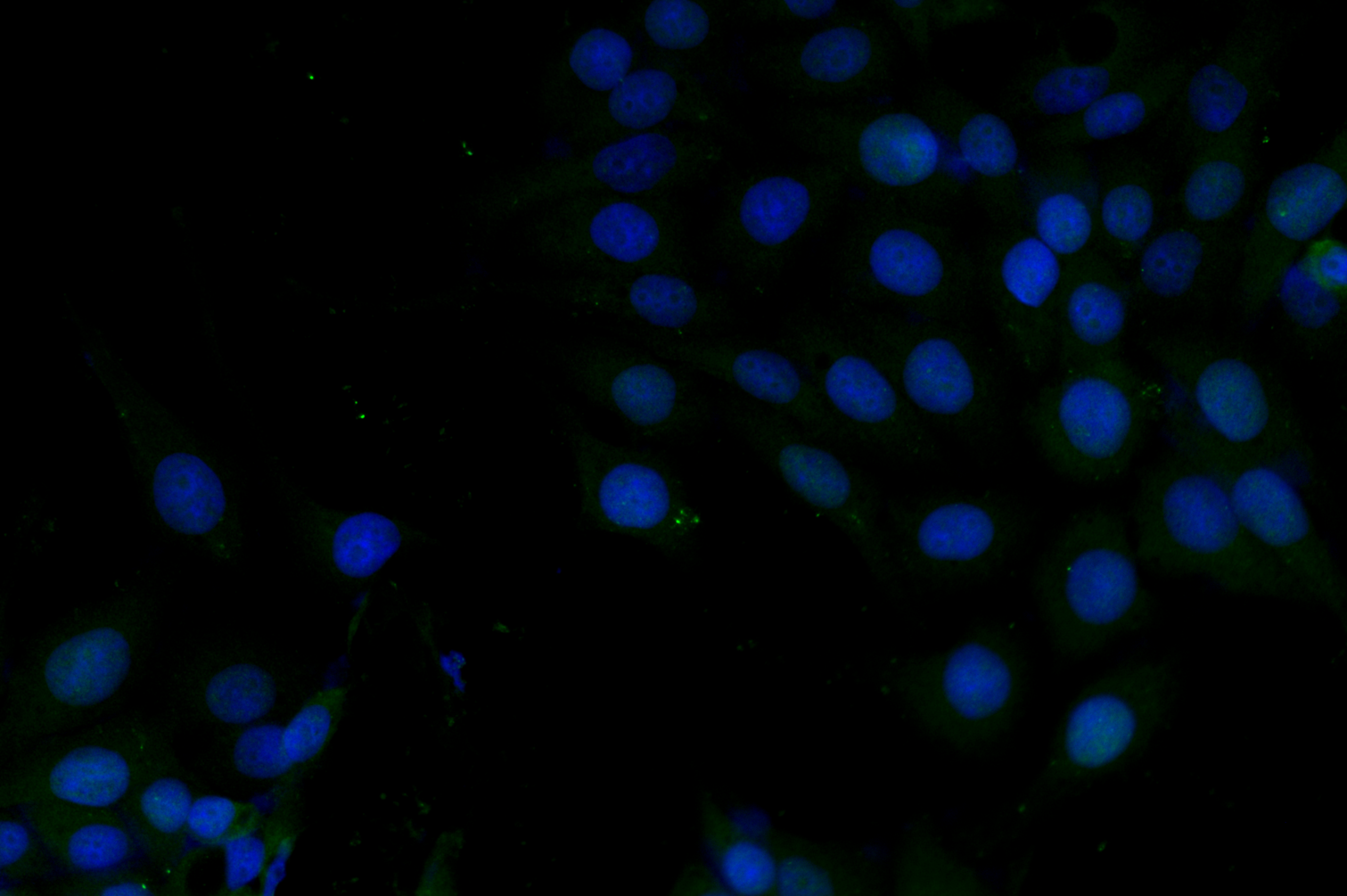

Supplement: Supplementary file 5 — Source data Fig. 3 [file 44319_2024_248_MOESM5_ESM.zip › Figure 3/Fig. 3B/MG132.tif]

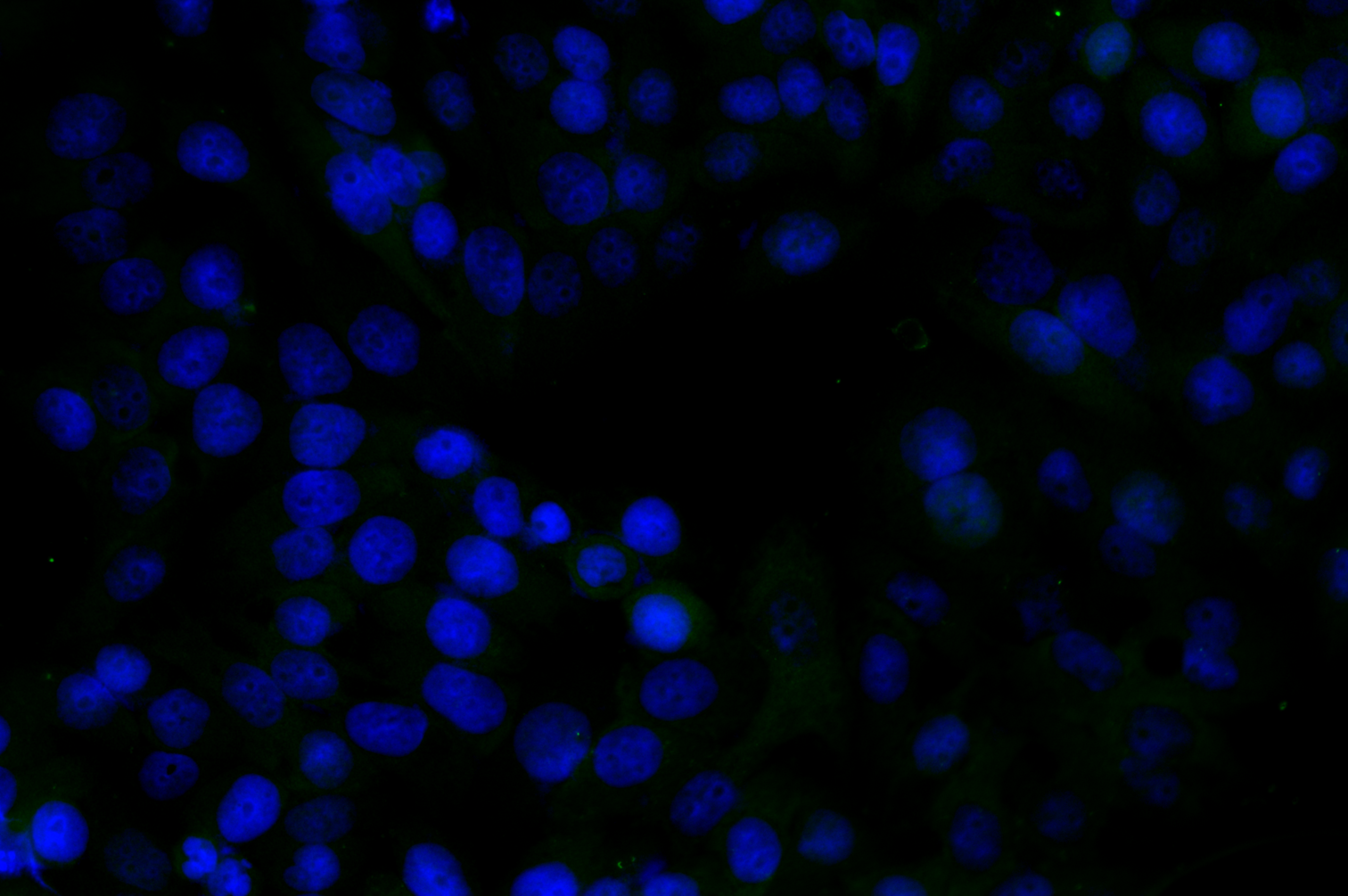

Supplement: Supplementary file 5 — Source data Fig. 3 [file 44319_2024_248_MOESM5_ESM.zip › Figure 3/Fig. 3B/MG132+KN-93.tif]

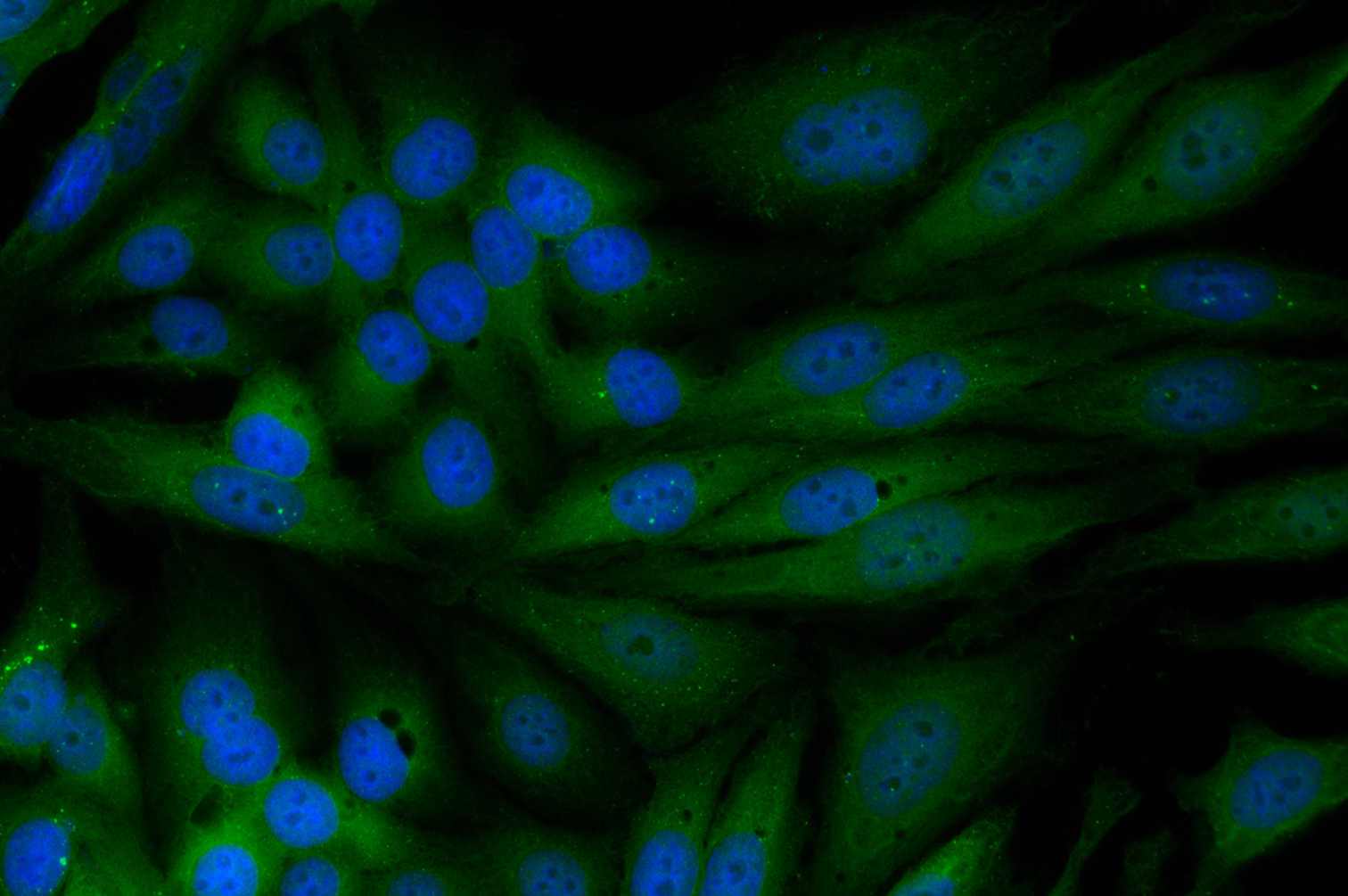

Supplement: Supplementary file 5 — Source data Fig. 3 [file 44319_2024_248_MOESM5_ESM.zip › Figure 3/Fig. 3F/MG132.tif]

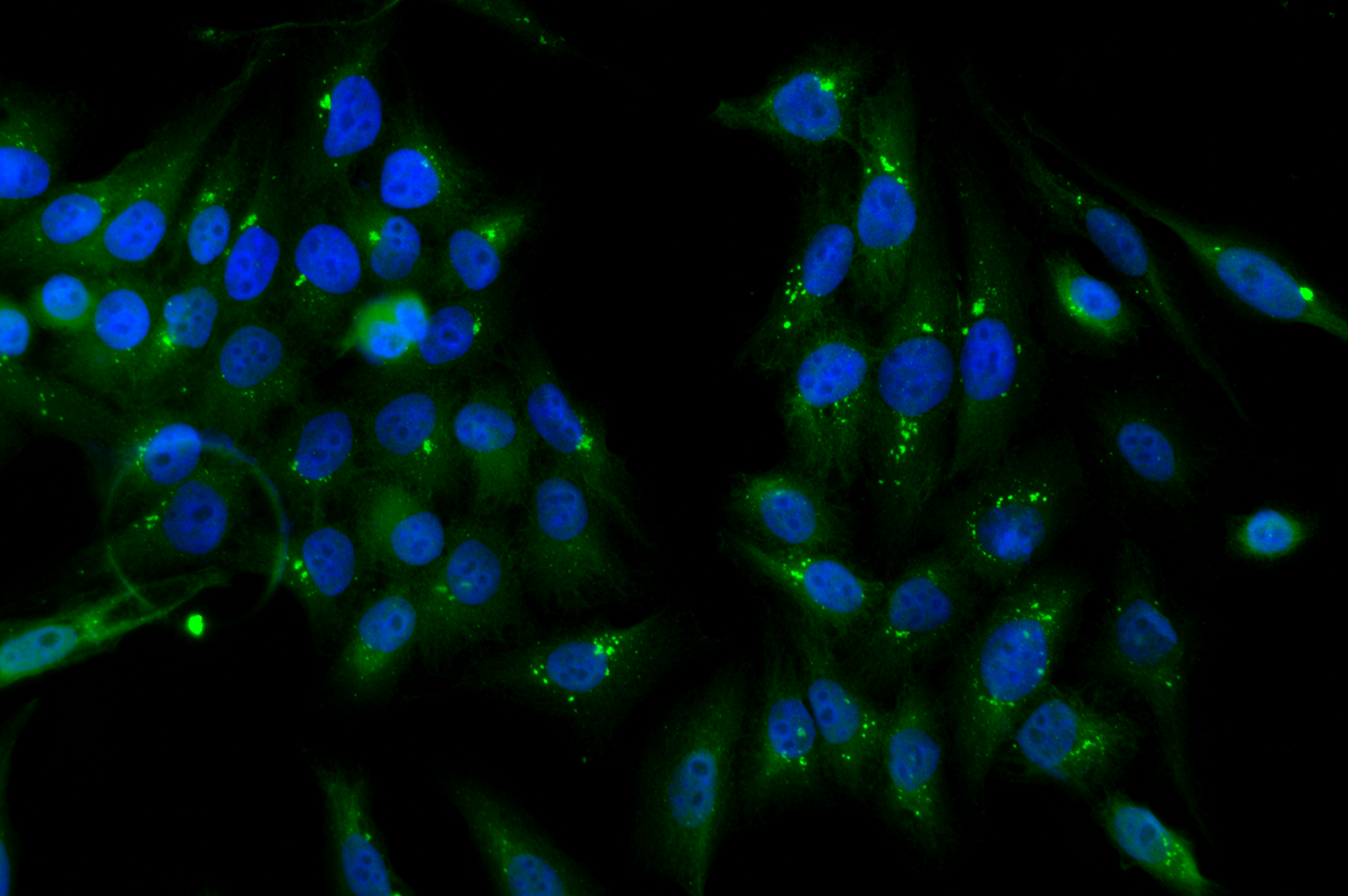

Supplement: Supplementary file 5 — Source data Fig. 3 [file 44319_2024_248_MOESM5_ESM.zip › Figure 3/Fig. 3F/MG132+KN-93.tif]

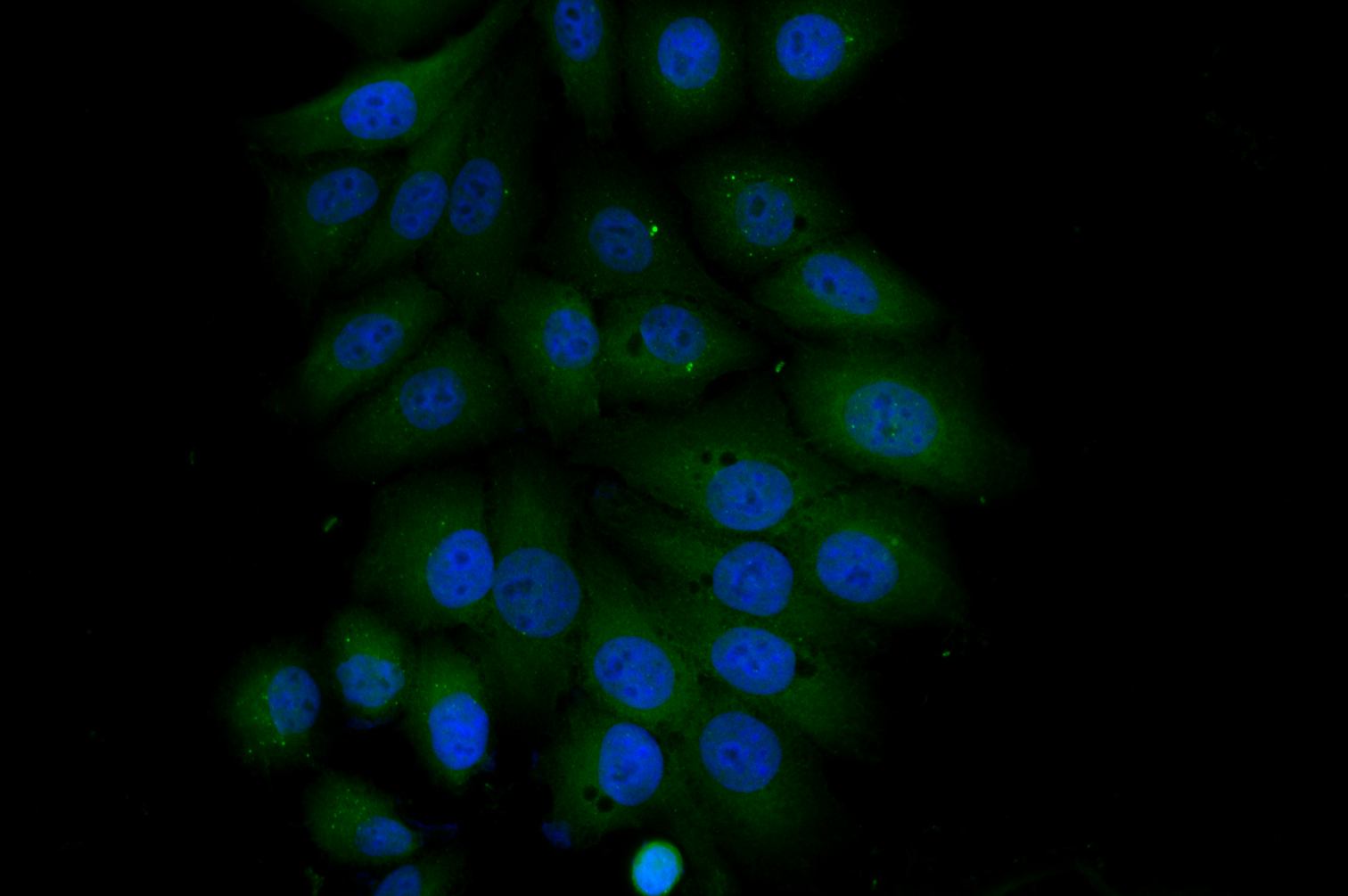

Supplement: Supplementary file 5 — Source data Fig. 3 [file 44319_2024_248_MOESM5_ESM.zip › Figure 3/Fig. 3F/MG132+KN-93+BTdCPU.tif]

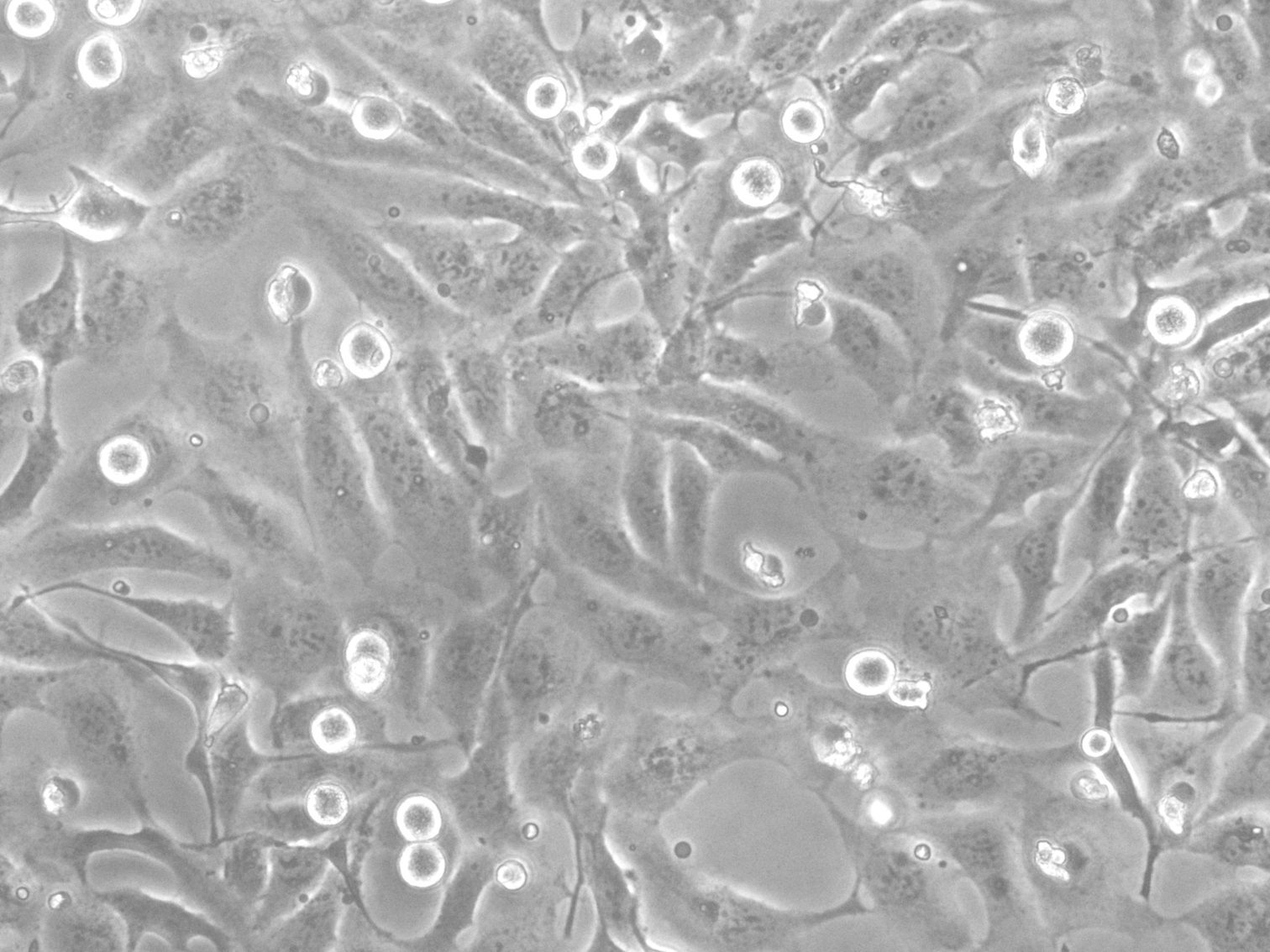

Supplement: Supplementary file 5 — Source data Fig. 3 [file 44319_2024_248_MOESM5_ESM.zip › Figure 3/Fig. 3H/HEK293-MG132.tif]

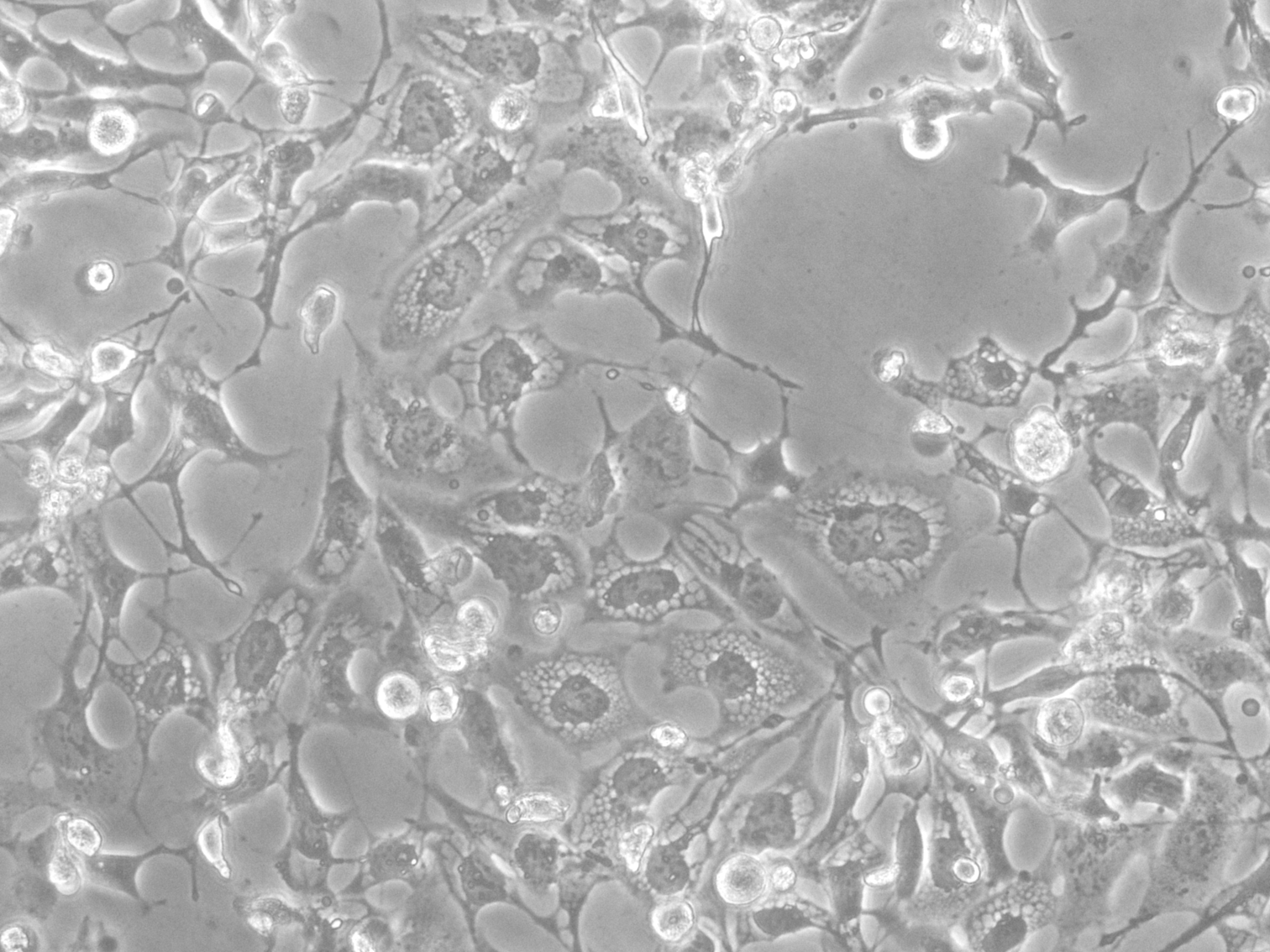

Supplement: Supplementary file 5 — Source data Fig. 3 [file 44319_2024_248_MOESM5_ESM.zip › Figure 3/Fig. 3H/HEK293-MG132+KN-93.tif]

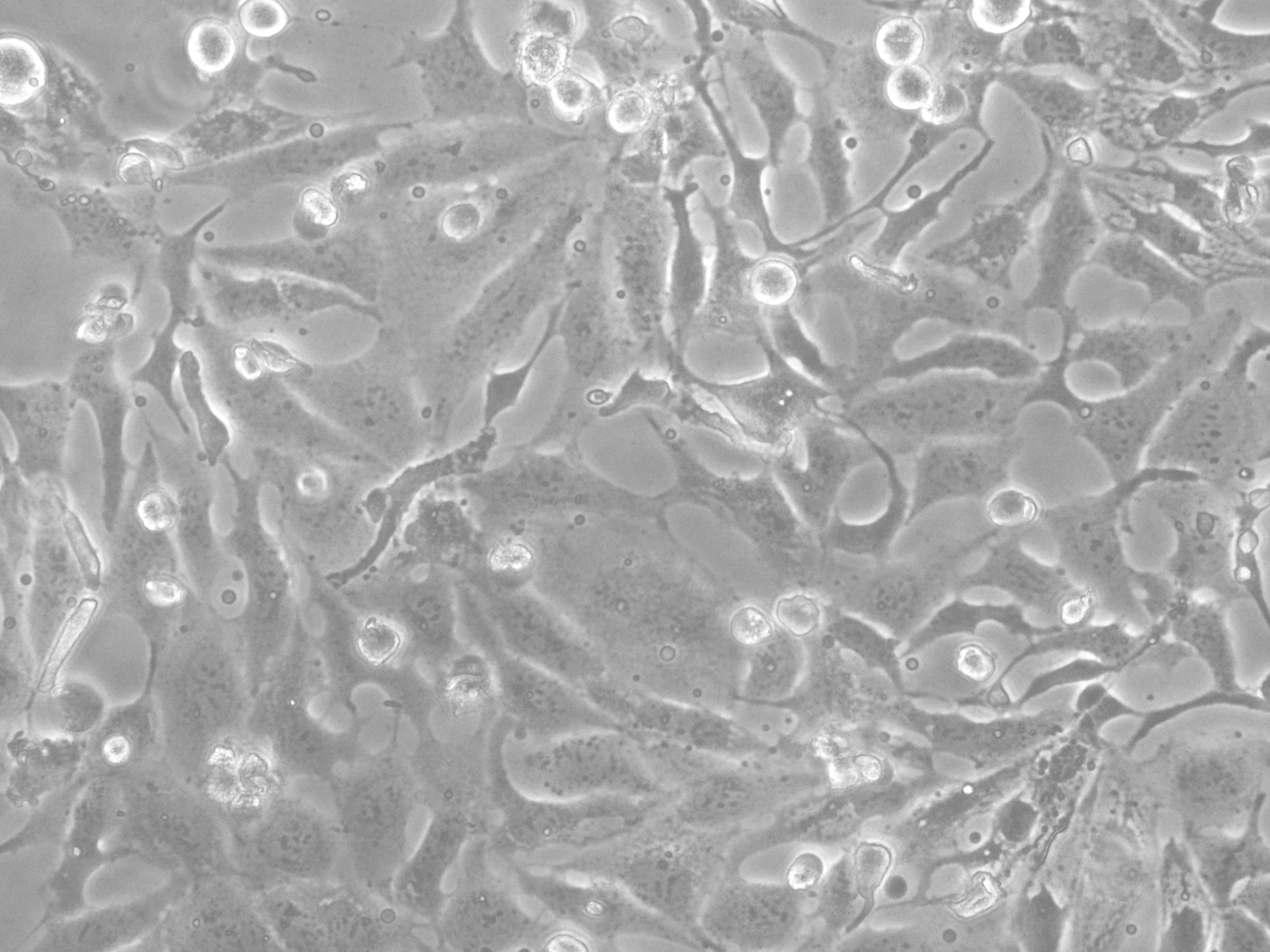

Supplement: Supplementary file 5 — Source data Fig. 3 [file 44319_2024_248_MOESM5_ESM.zip › Figure 3/Fig. 3H/HEK293-MG132+KN-93+BTdCPU.tif]

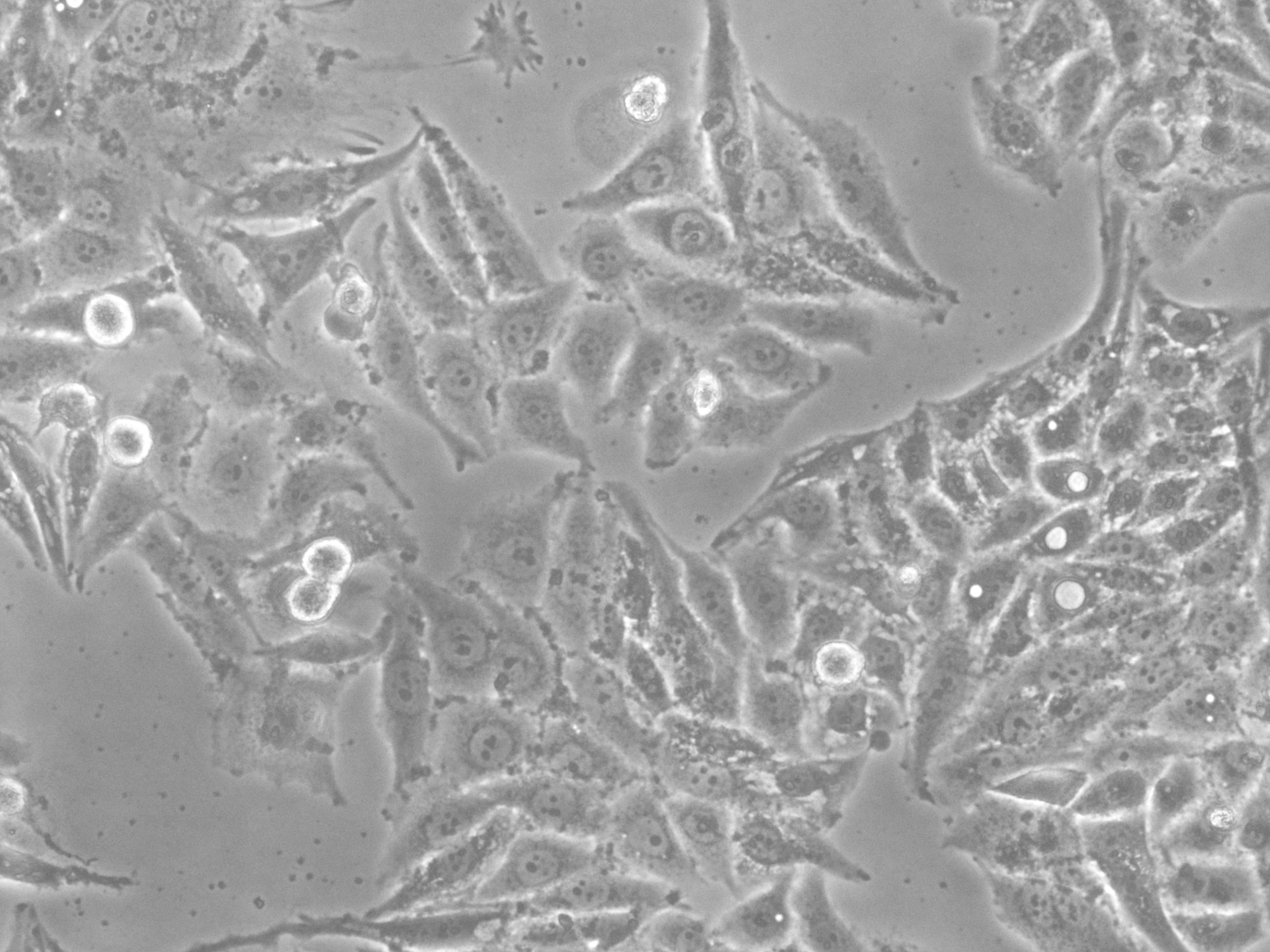

Supplement: Supplementary file 5 — Source data Fig. 3 [file 44319_2024_248_MOESM5_ESM.zip › Figure 3/Fig. 3H/Hela-MG132.tif]

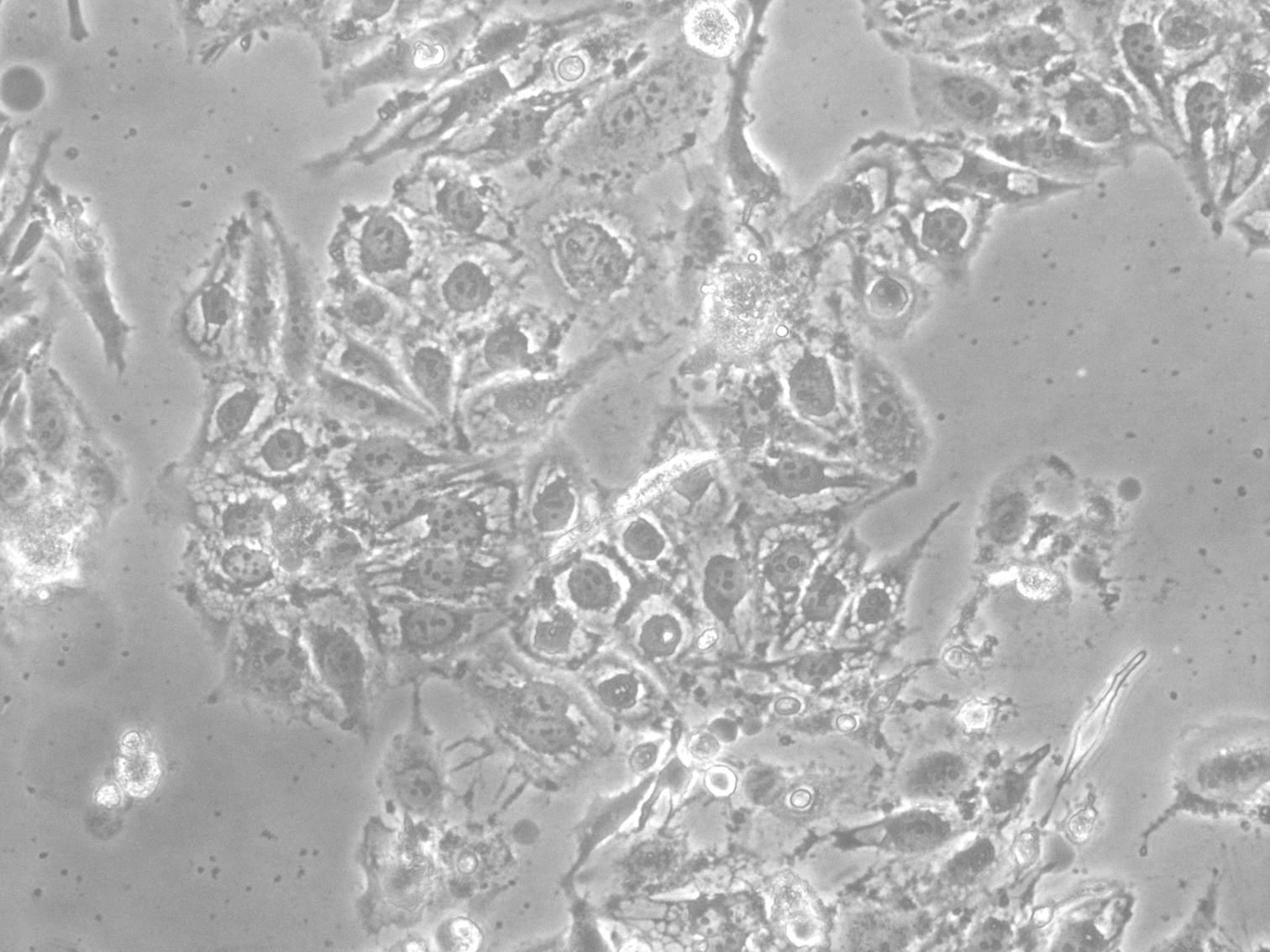

Supplement: Supplementary file 5 — Source data Fig. 3 [file 44319_2024_248_MOESM5_ESM.zip › Figure 3/Fig. 3H/Hela-MG132+KN-93.tif]

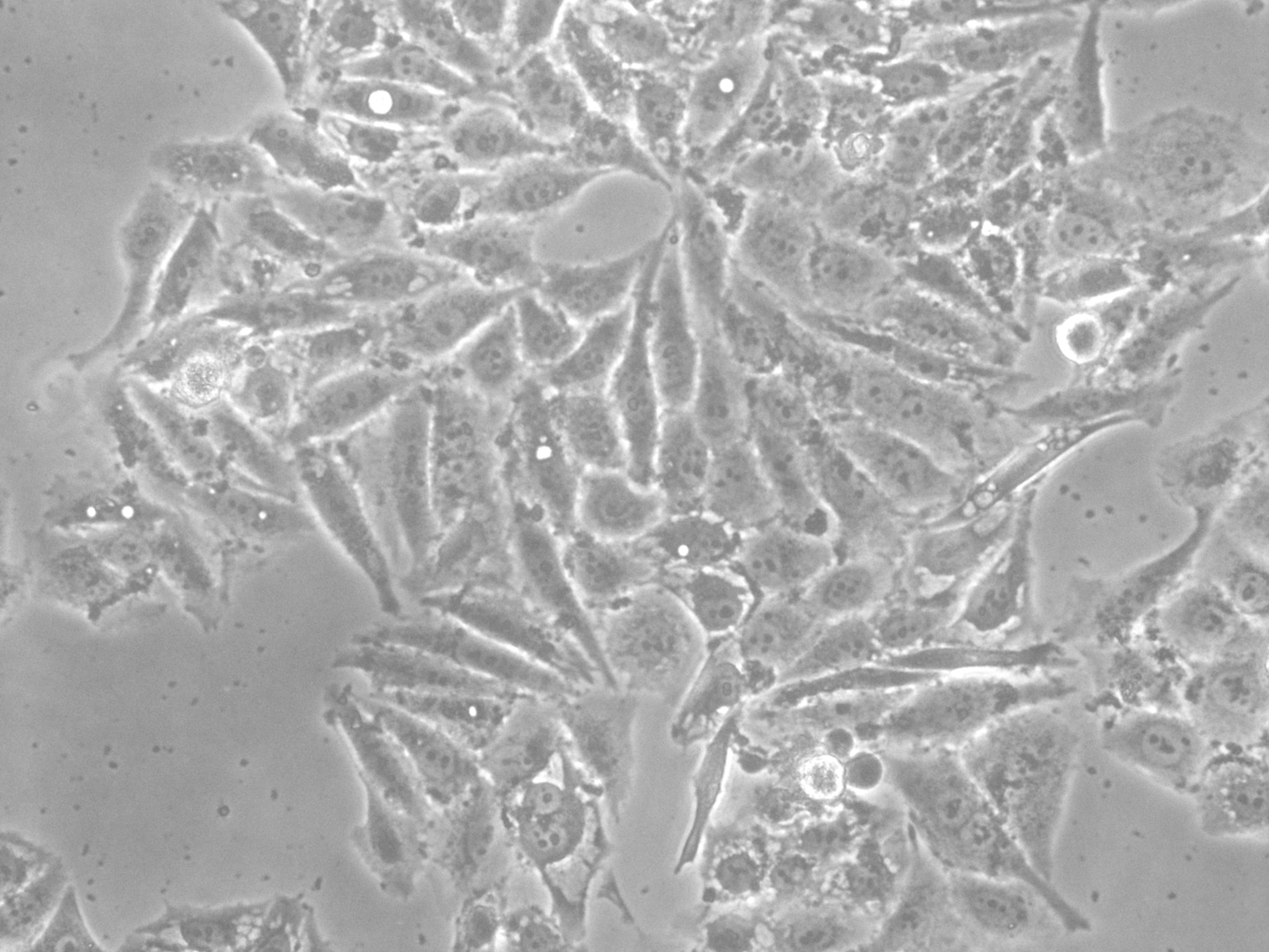

Supplement: Supplementary file 5 — Source data Fig. 3 [file 44319_2024_248_MOESM5_ESM.zip › Figure 3/Fig. 3H/Hela-MG132+KN-93+BTdCPU.tif]

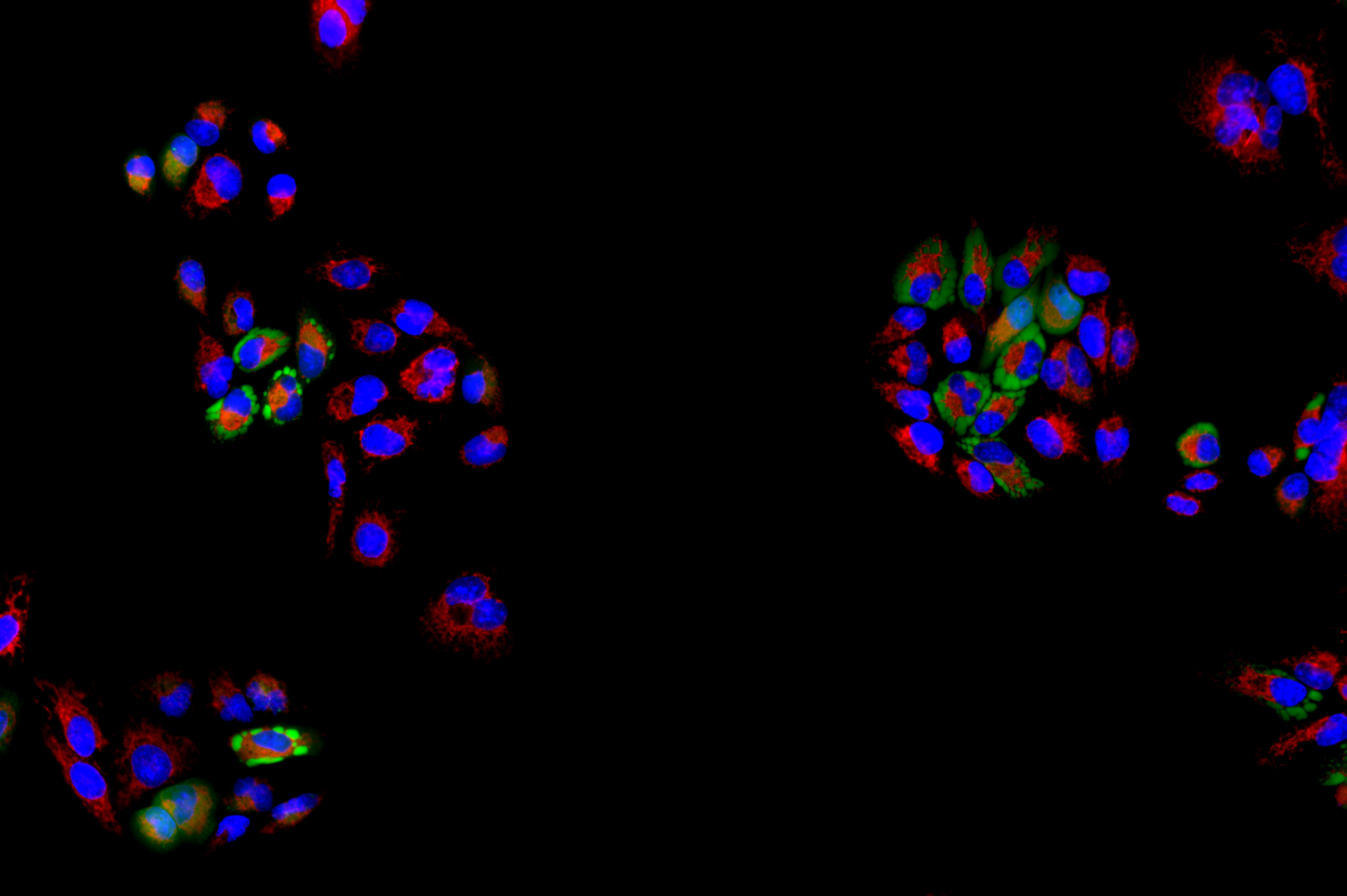

Supplement: Supplementary file 5 — Source data Fig. 3 [file 44319_2024_248_MOESM5_ESM.zip › Figure 3/Fig. 3J/MG132+KN-93 1.tif]

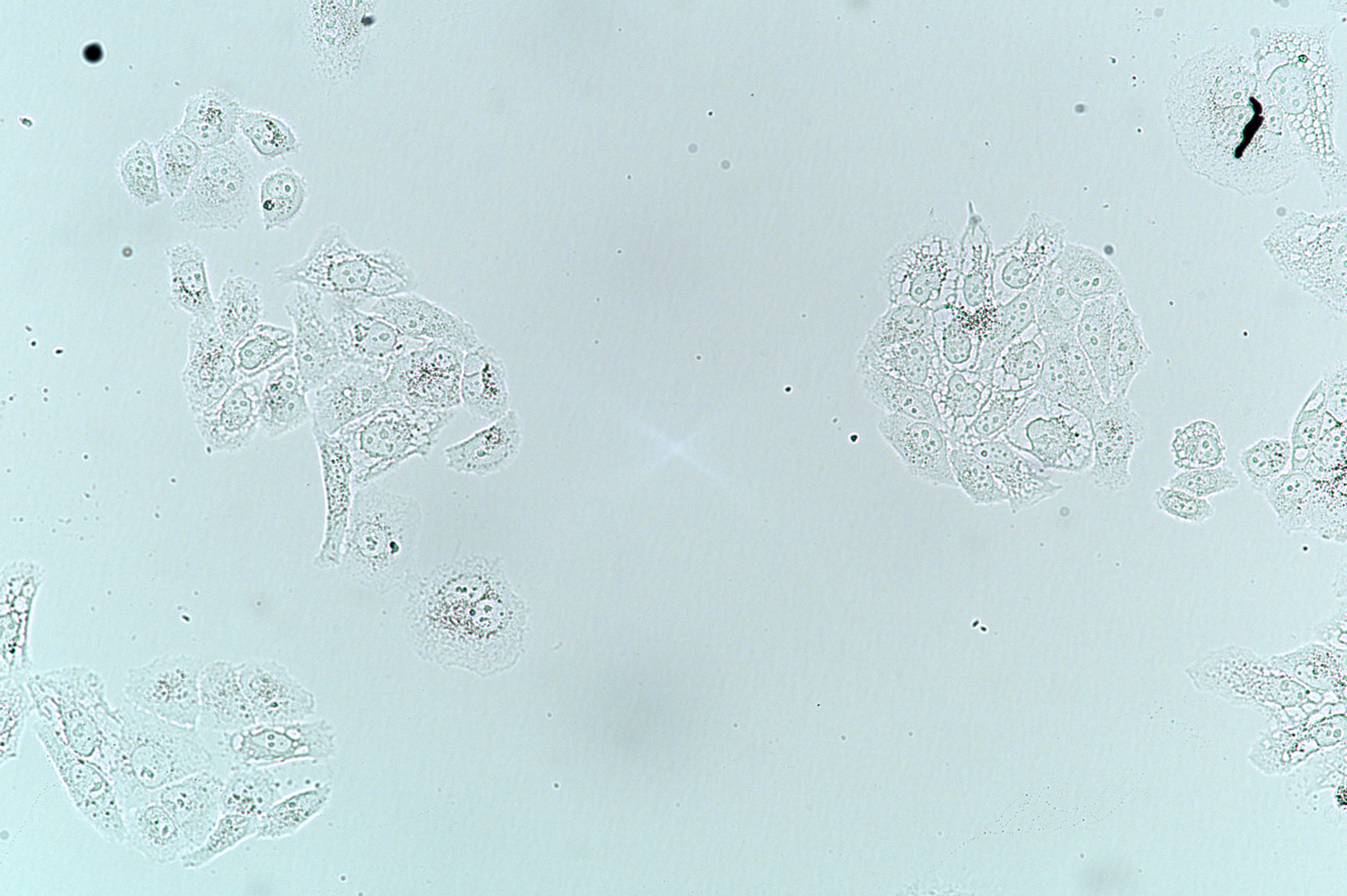

Supplement: Supplementary file 5 — Source data Fig. 3 [file 44319_2024_248_MOESM5_ESM.zip › Figure 3/Fig. 3J/MG132+KN-93 2.tif]

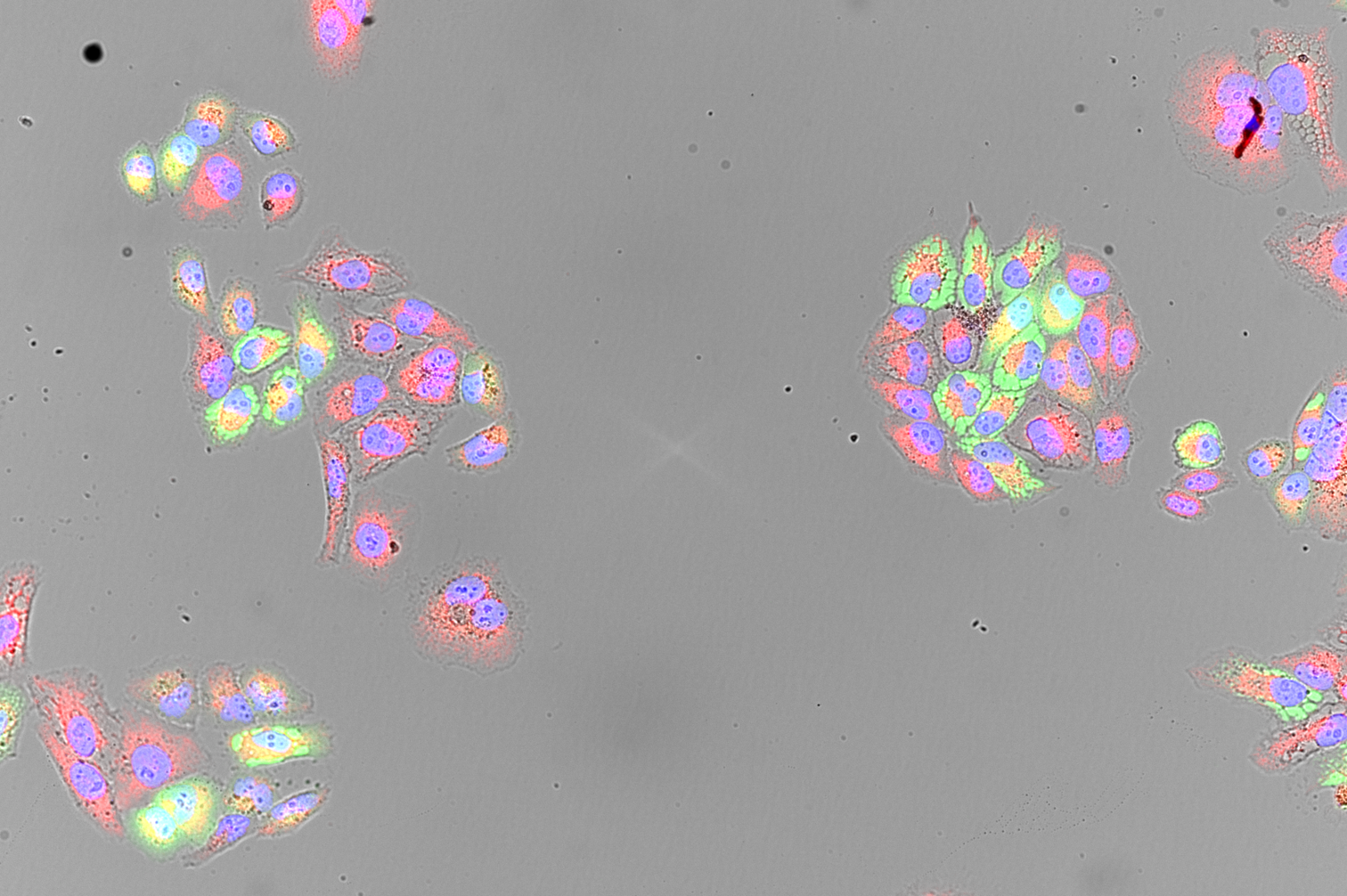

Supplement: Supplementary file 5 — Source data Fig. 3 [file 44319_2024_248_MOESM5_ESM.zip › Figure 3/Fig. 3J/MG132+KN-93 3.tif]

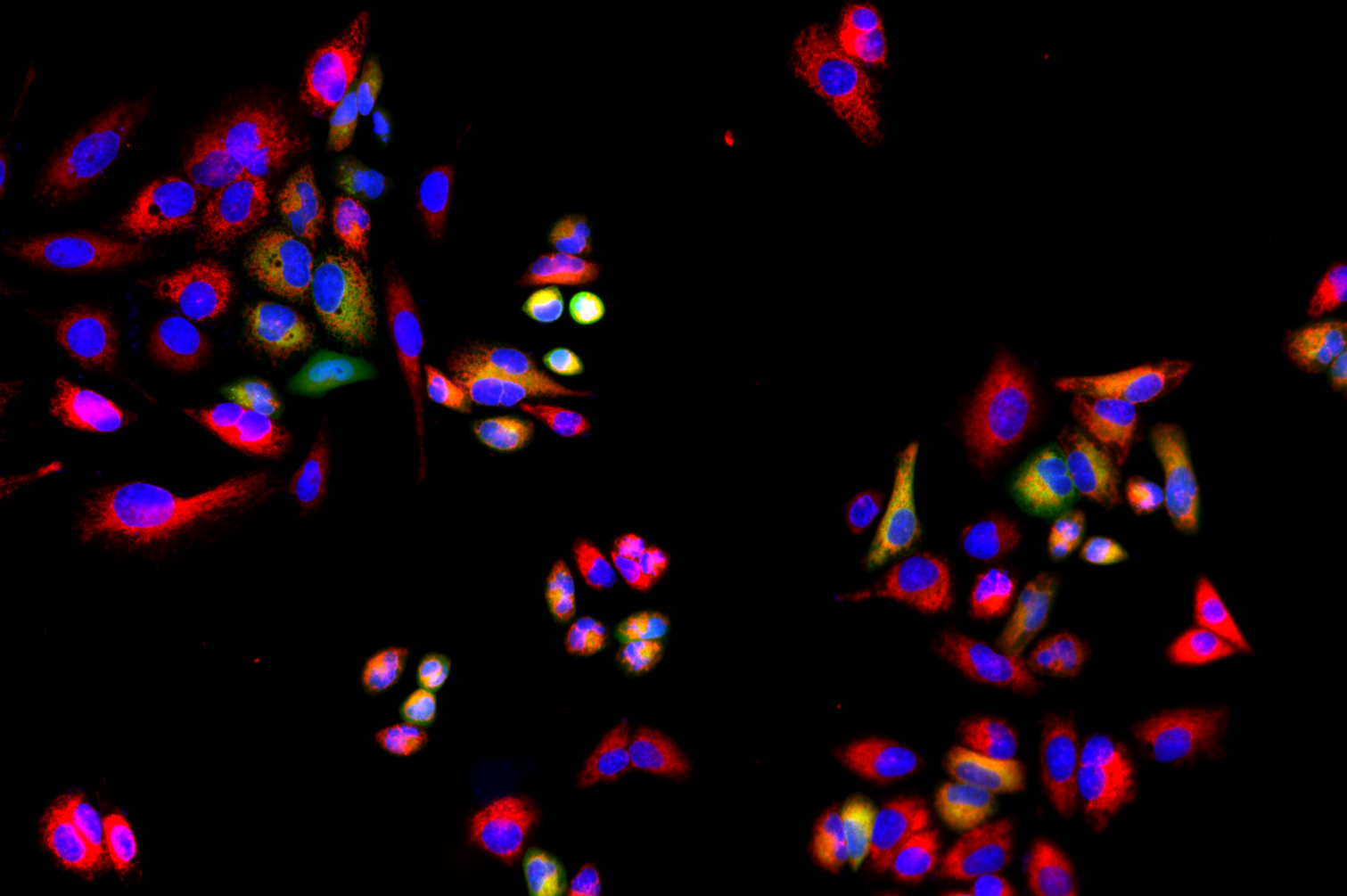

Supplement: Supplementary file 5 — Source data Fig. 3 [file 44319_2024_248_MOESM5_ESM.zip › Figure 3/Fig. 3J/MG132+KN-93+BTdCPU 1.tif]

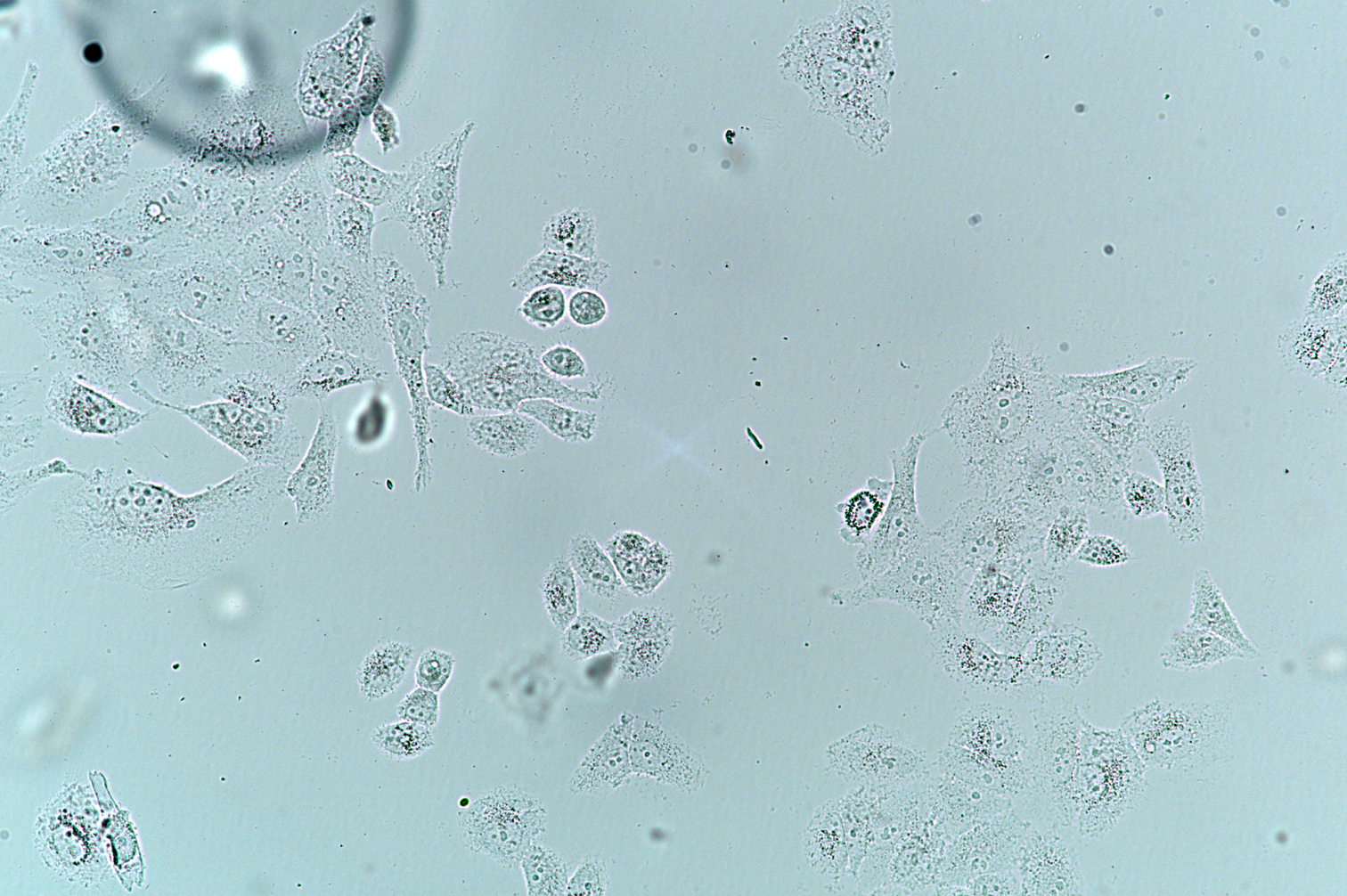

Supplement: Supplementary file 5 — Source data Fig. 3 [file 44319_2024_248_MOESM5_ESM.zip › Figure 3/Fig. 3J/MG132+KN-93+BTdCPU 2.tif]

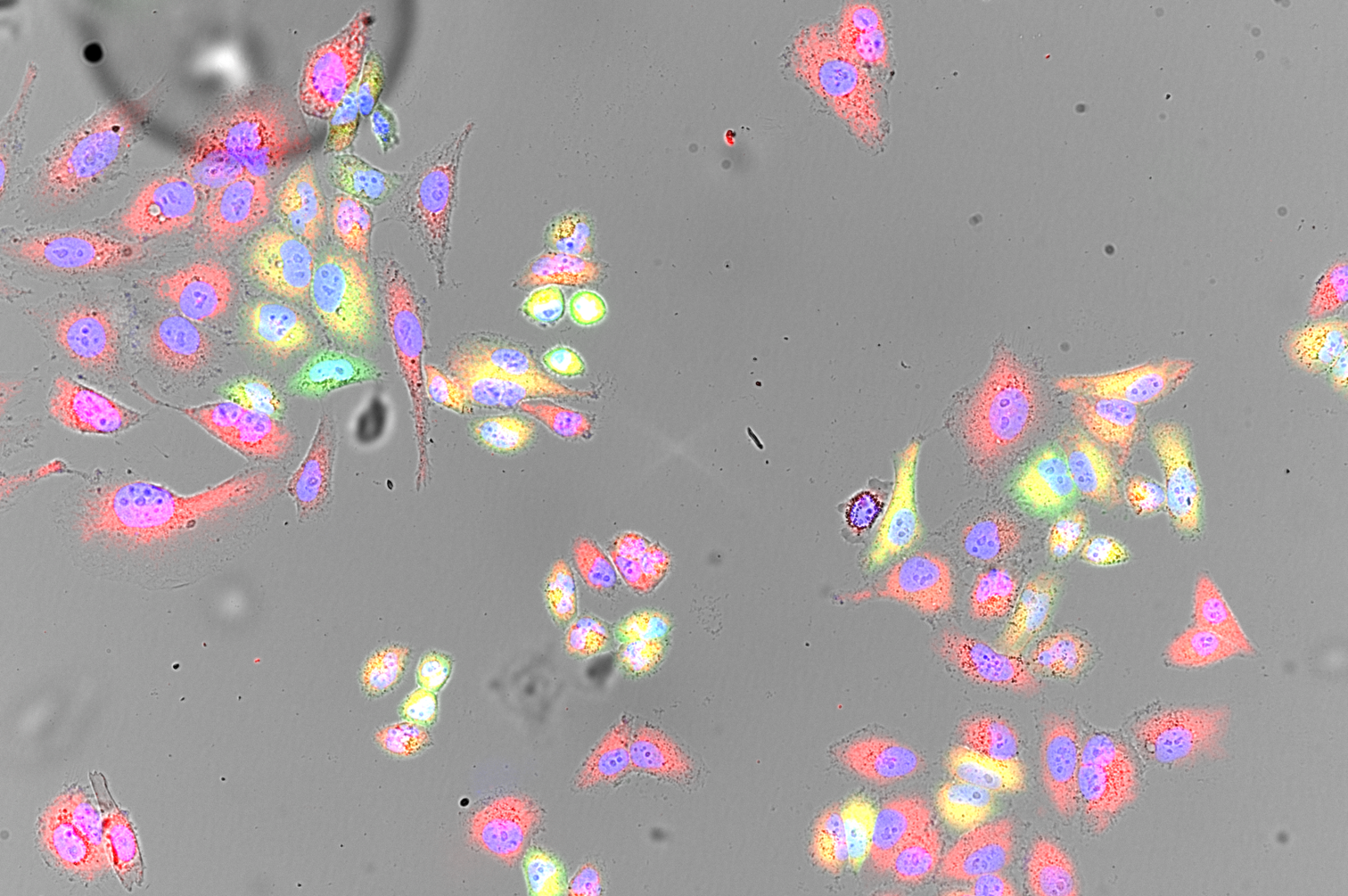

Supplement: Supplementary file 5 — Source data Fig. 3 [file 44319_2024_248_MOESM5_ESM.zip › Figure 3/Fig. 3J/MG132+KN-93+BTdCPU 3.tif]

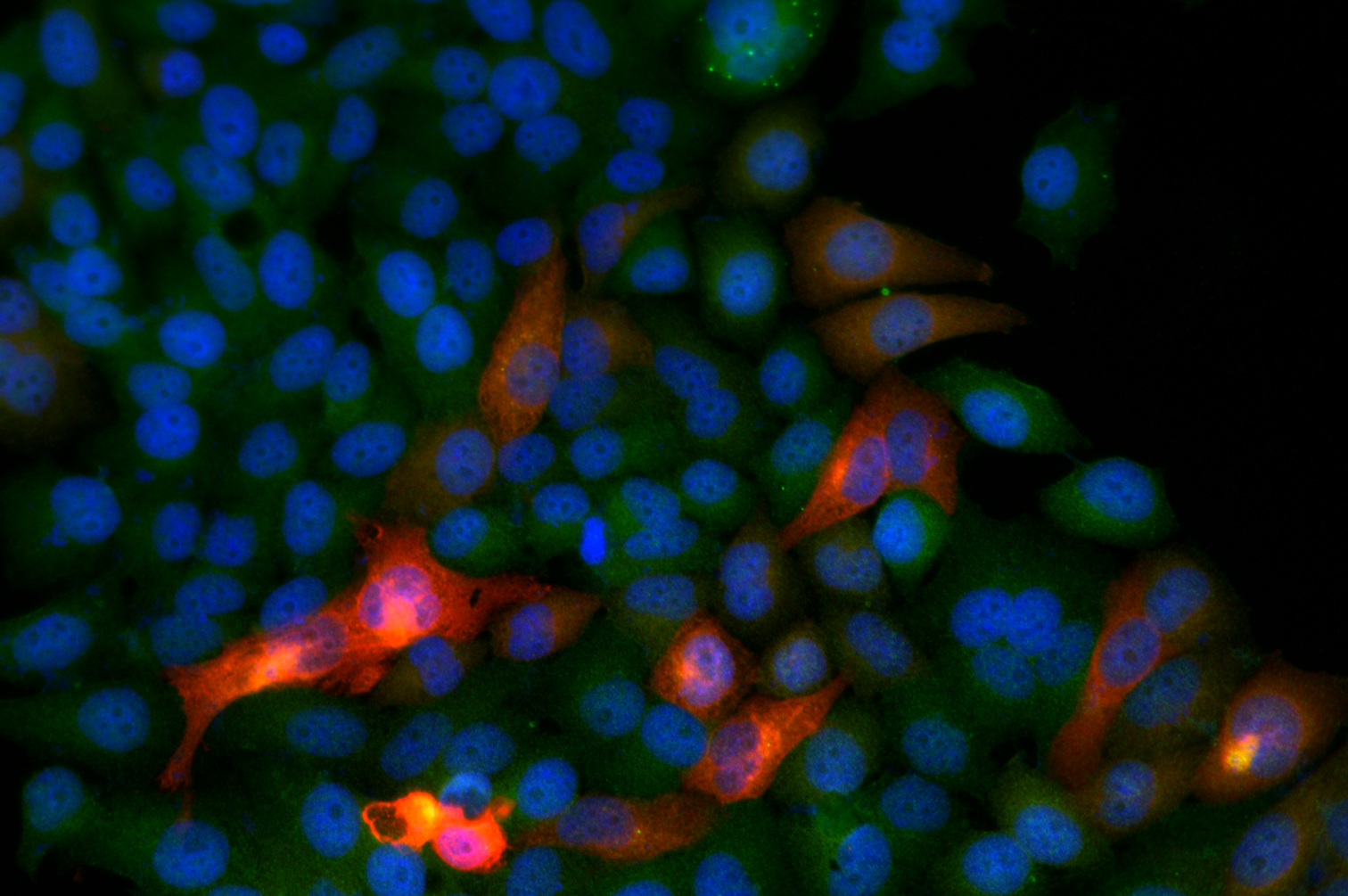

Supplement: Supplementary file 7 — Source data Fig. 5 [file 44319_2024_248_MOESM7_ESM.zip › Figure 5/Fig. 5B/DMSO.tif]

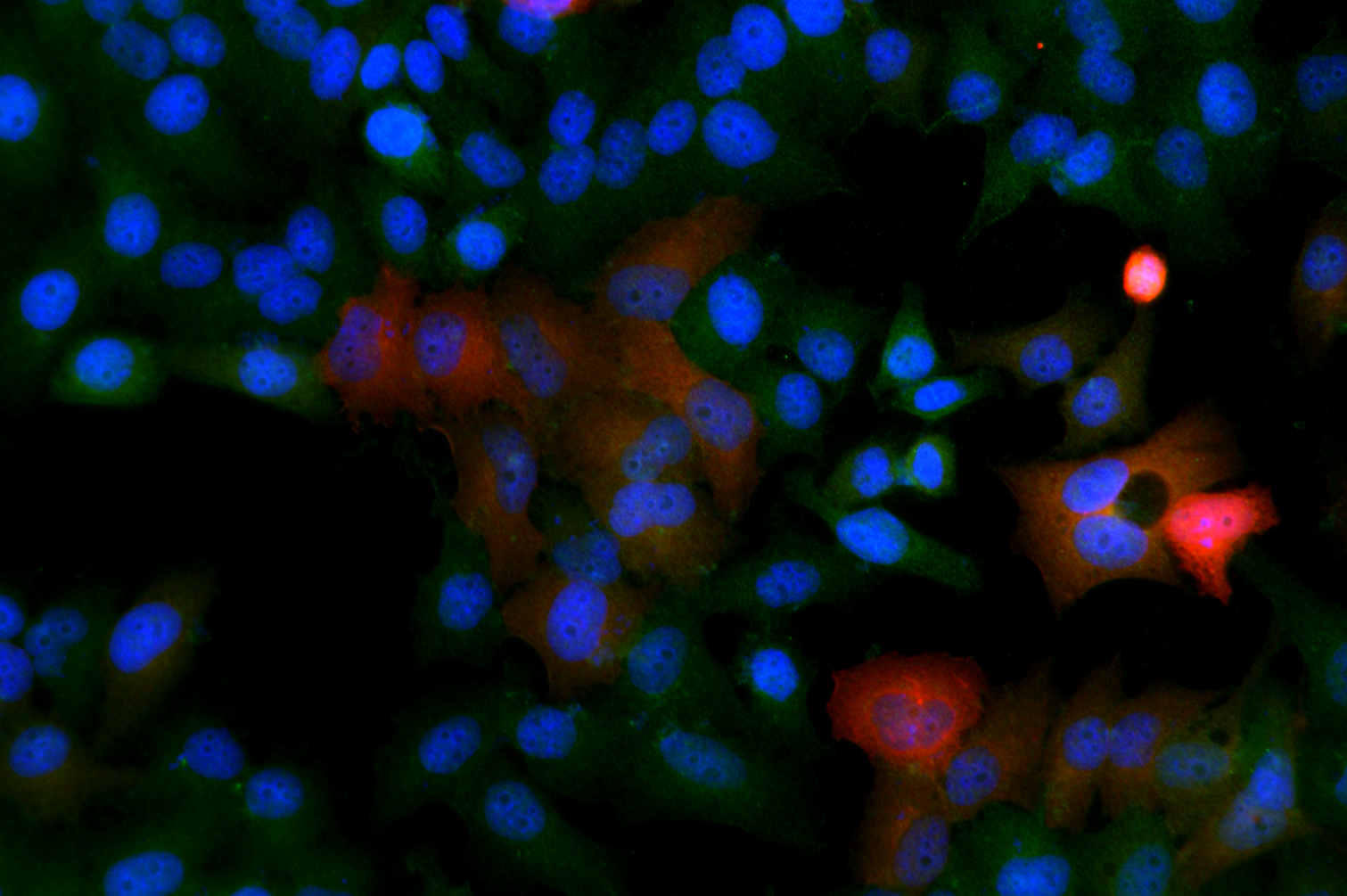

Supplement: Supplementary file 7 — Source data Fig. 5 [file 44319_2024_248_MOESM7_ESM.zip › Figure 5/Fig. 5B/KN-93.tif]

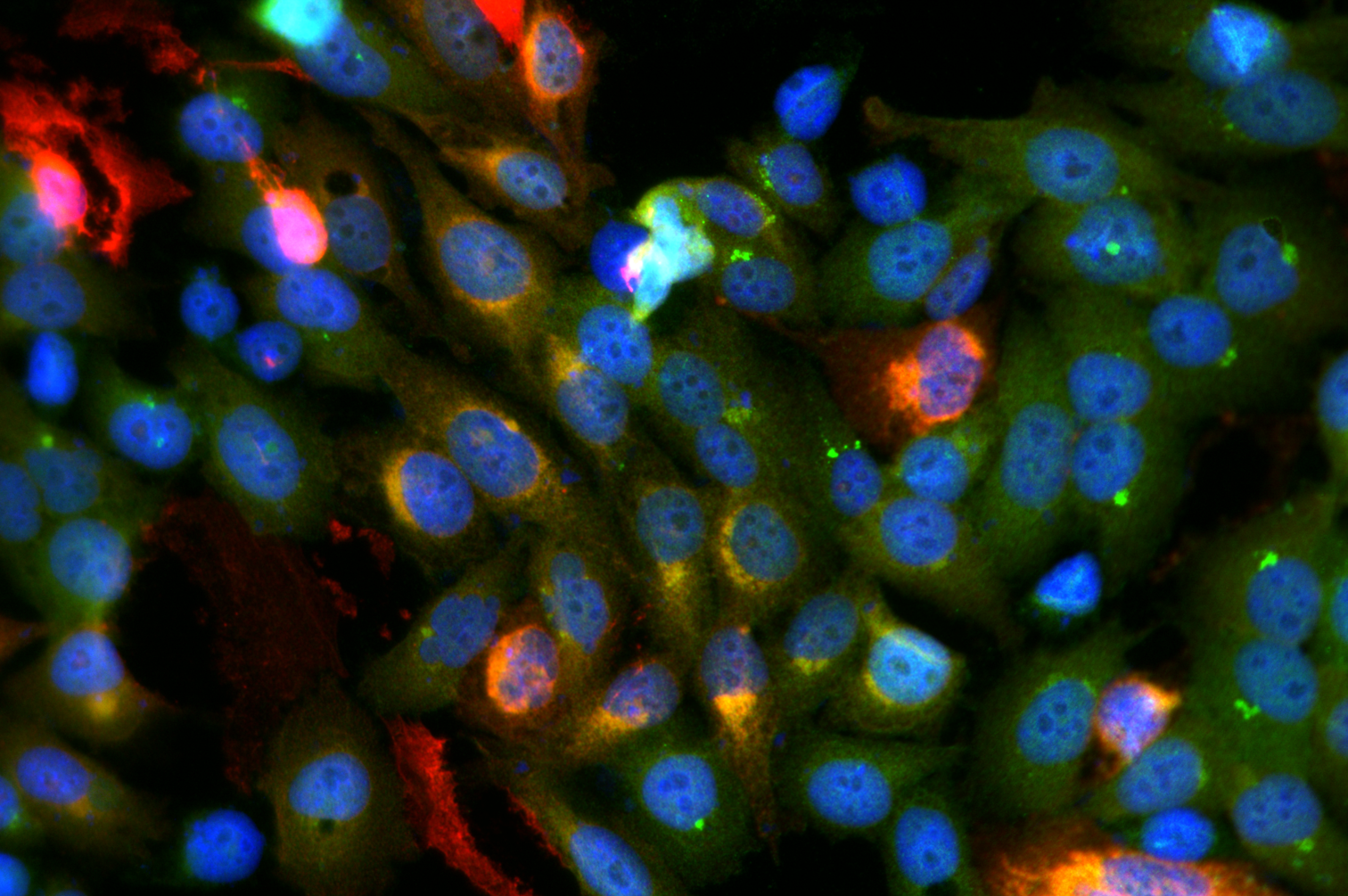

Supplement: Supplementary file 7 — Source data Fig. 5 [file 44319_2024_248_MOESM7_ESM.zip › Figure 5/Fig. 5B/MG132.tif]

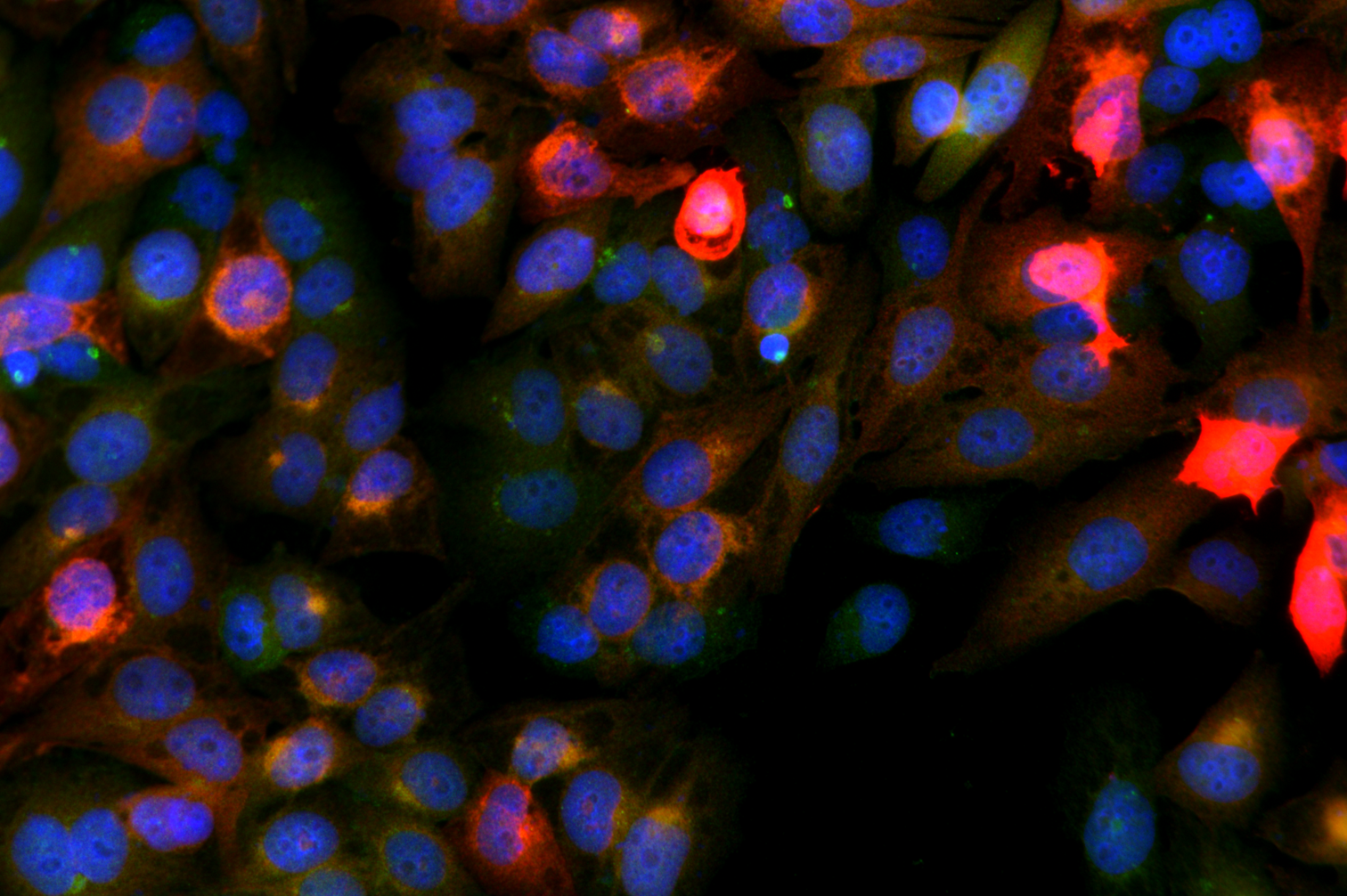

Supplement: Supplementary file 7 — Source data Fig. 5 [file 44319_2024_248_MOESM7_ESM.zip › Figure 5/Fig. 5B/MG132+KN-93.tif]

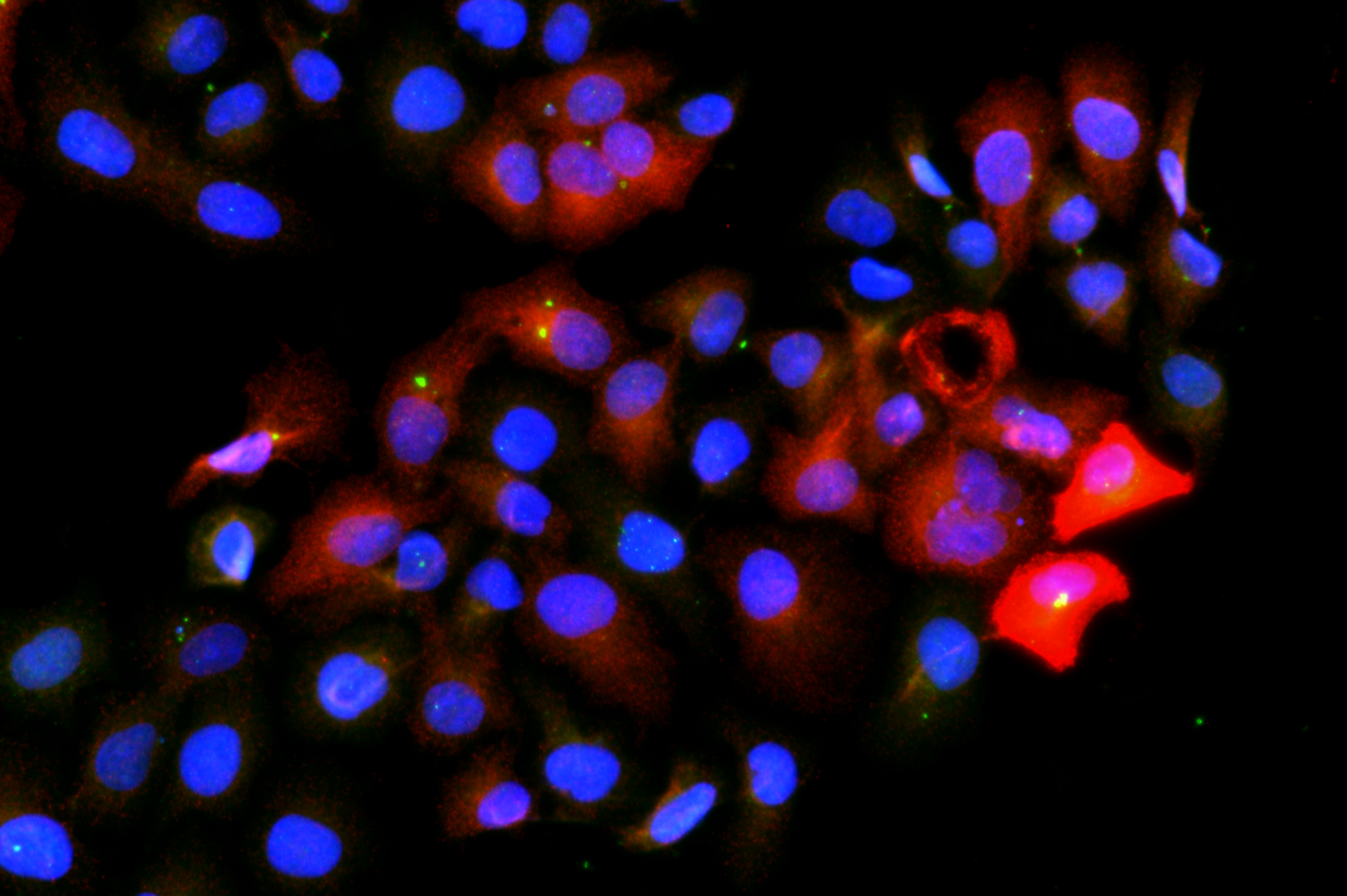

Supplement: Supplementary file 8 — Source data Fig. 6 [file 44319_2024_248_MOESM8_ESM.zip › Figure 6/Fig. 6B/BAG3 3A-DMSO.tif]

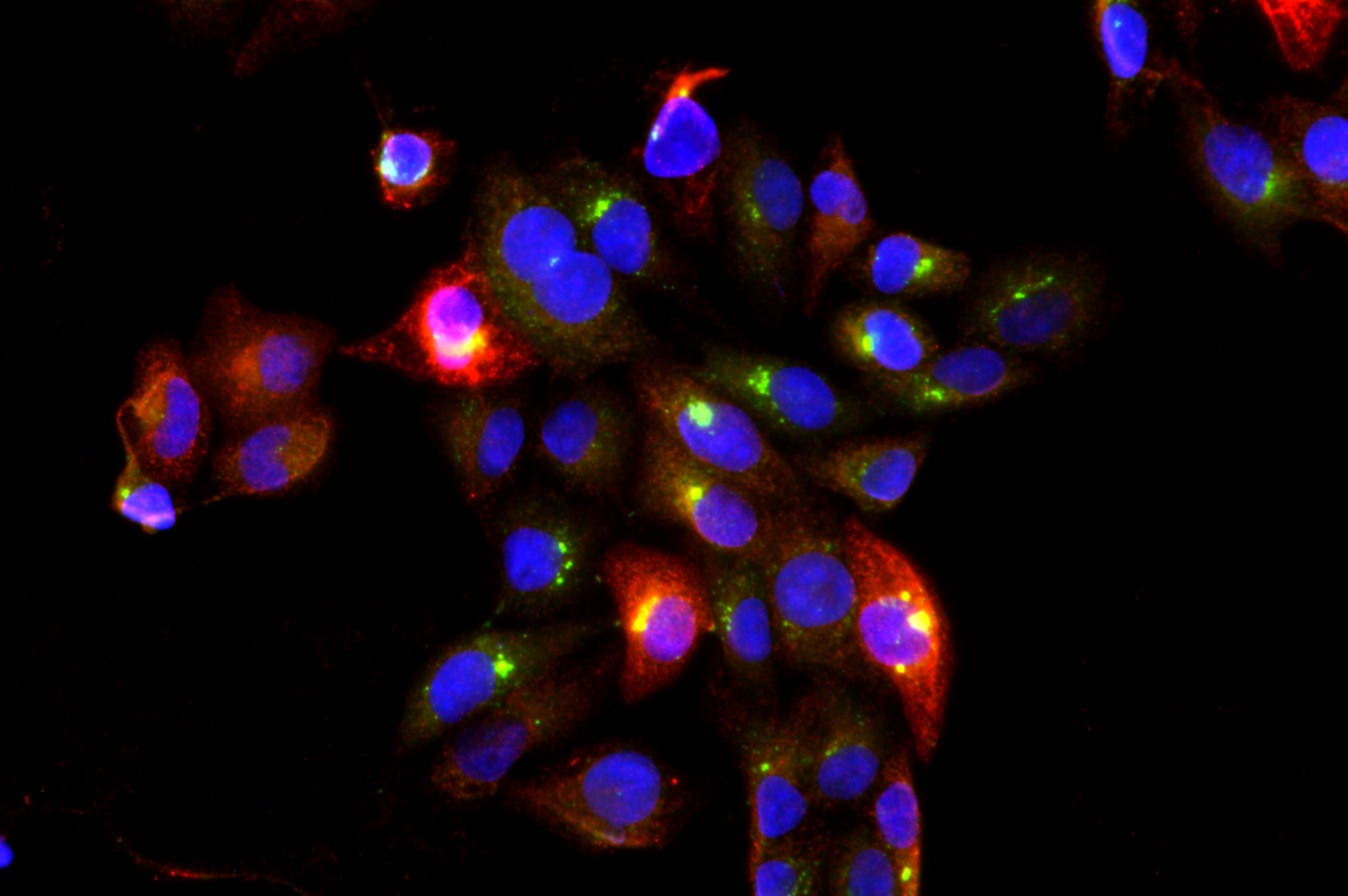

Supplement: Supplementary file 8 — Source data Fig. 6 [file 44319_2024_248_MOESM8_ESM.zip › Figure 6/Fig. 6B/BAG3 3A-MG132.tif]
