# Supplementary figures and images for: CaMKII suppresses proteotoxicity by phosphorylating BAG3 in response to proteasomal dysfunction (part 2 of 2)
Source: EMBO Rep. 2024 Sep 11;25(10):4488–514. doi: 10.1038/s44319-024-00248-w (PMC11466968; doi:10.1038/s44319-024-00248-w)

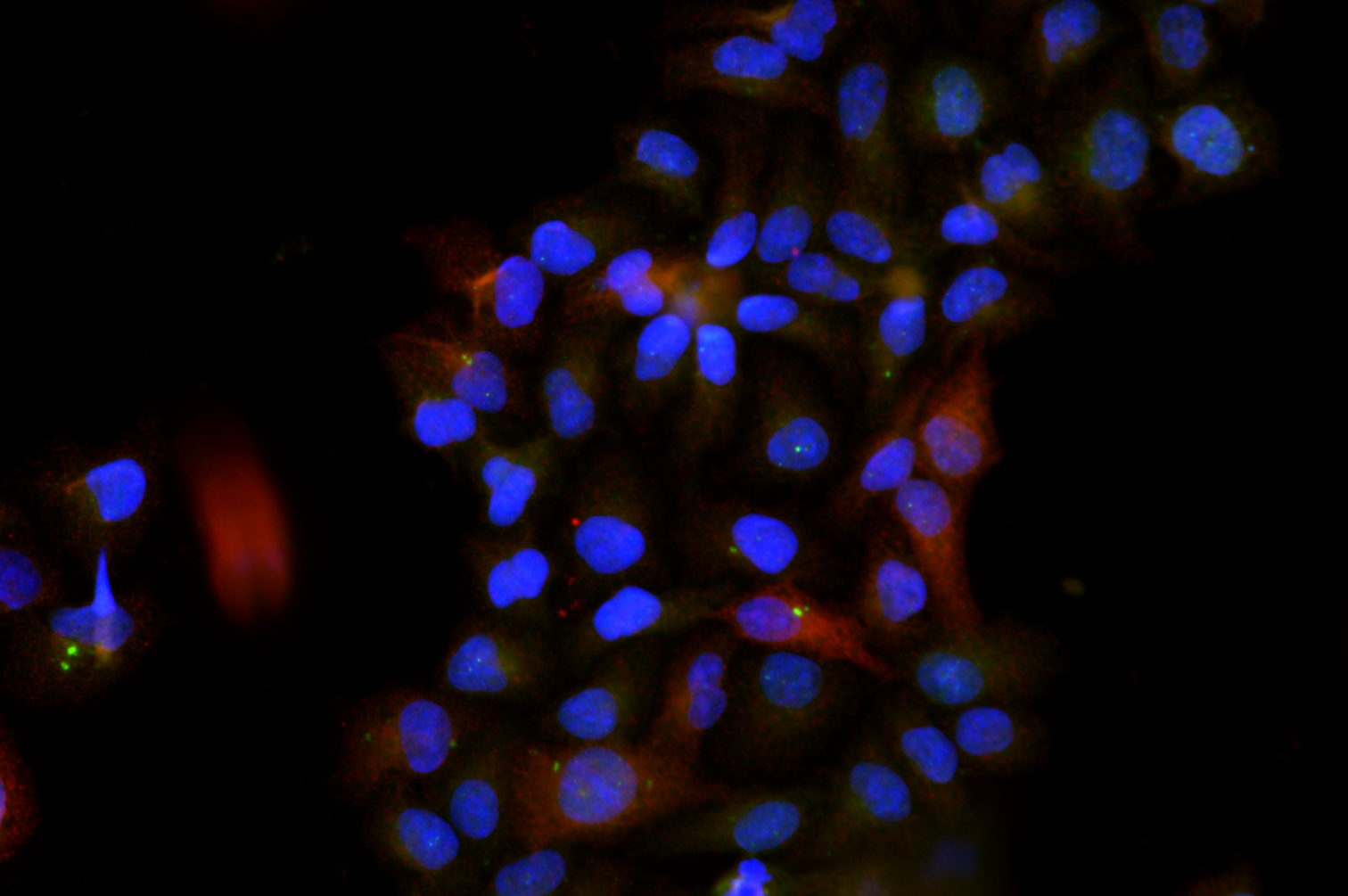

Supplement: Supplementary file 8 — Source data Fig. 6 [file 44319_2024_248_MOESM8_ESM.zip › Figure 6/Fig. 6B/BAG3 WT-DMSO.tif]

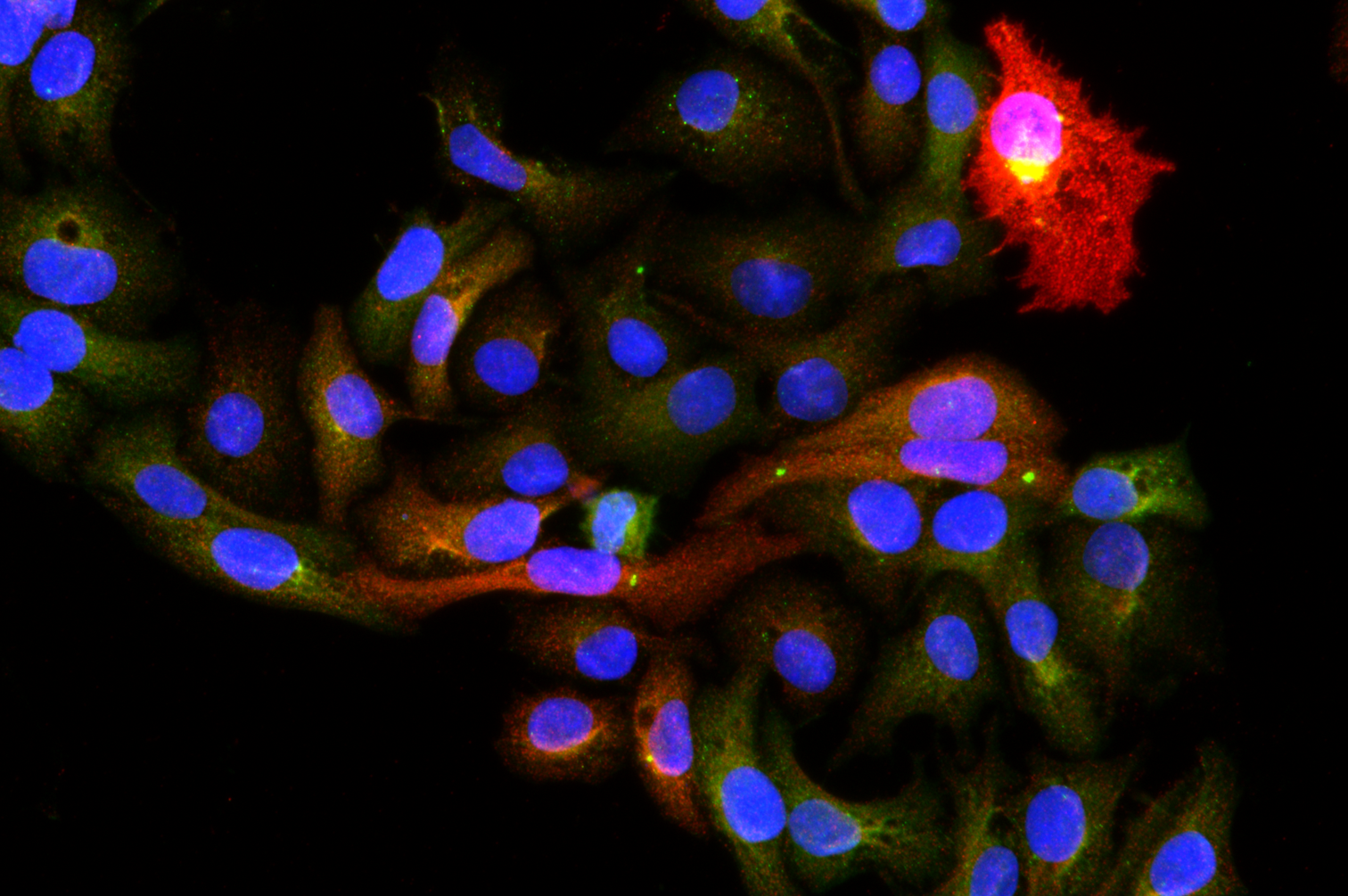

Supplement: Supplementary file 8 — Source data Fig. 6 [file 44319_2024_248_MOESM8_ESM.zip › Figure 6/Fig. 6B/BAG3 WT-MG132.tif]

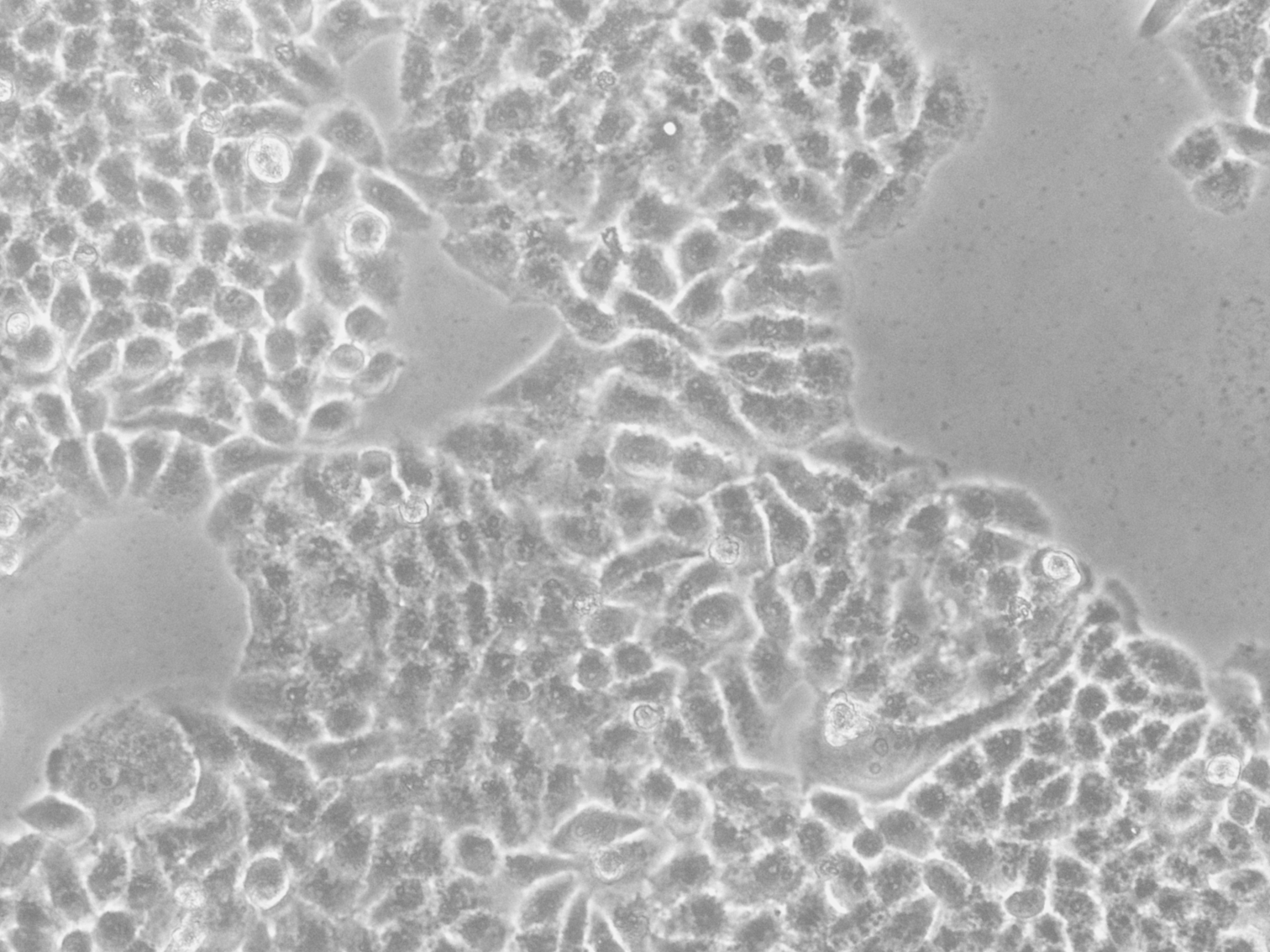

Supplement: Supplementary file 8 — Source data Fig. 6 [file 44319_2024_248_MOESM8_ESM.zip › Figure 6/Fig. 6D/BAG3 3A -DMSO.tif]

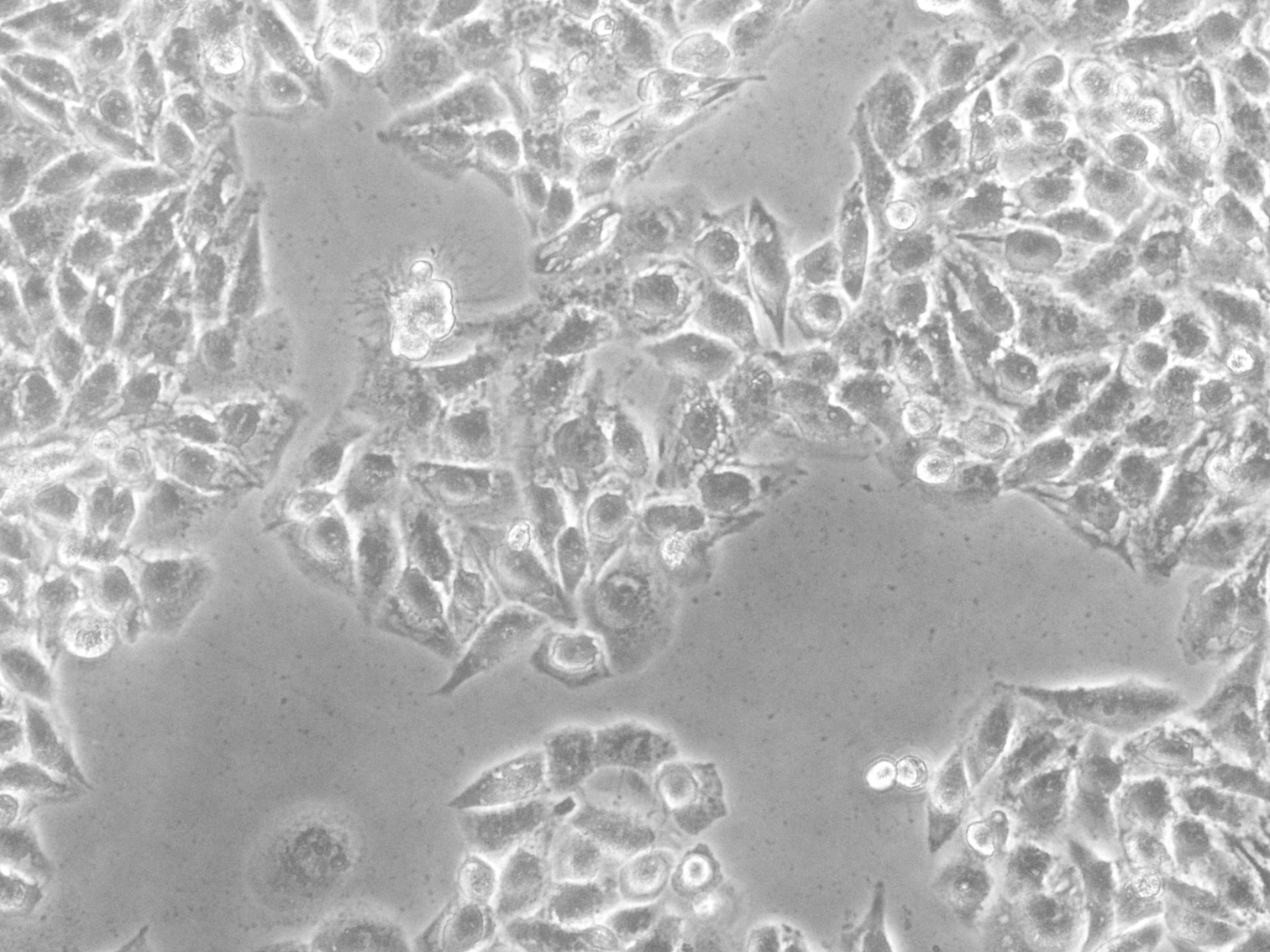

Supplement: Supplementary file 8 — Source data Fig. 6 [file 44319_2024_248_MOESM8_ESM.zip › Figure 6/Fig. 6D/BAG3 3A -MG132.tif]

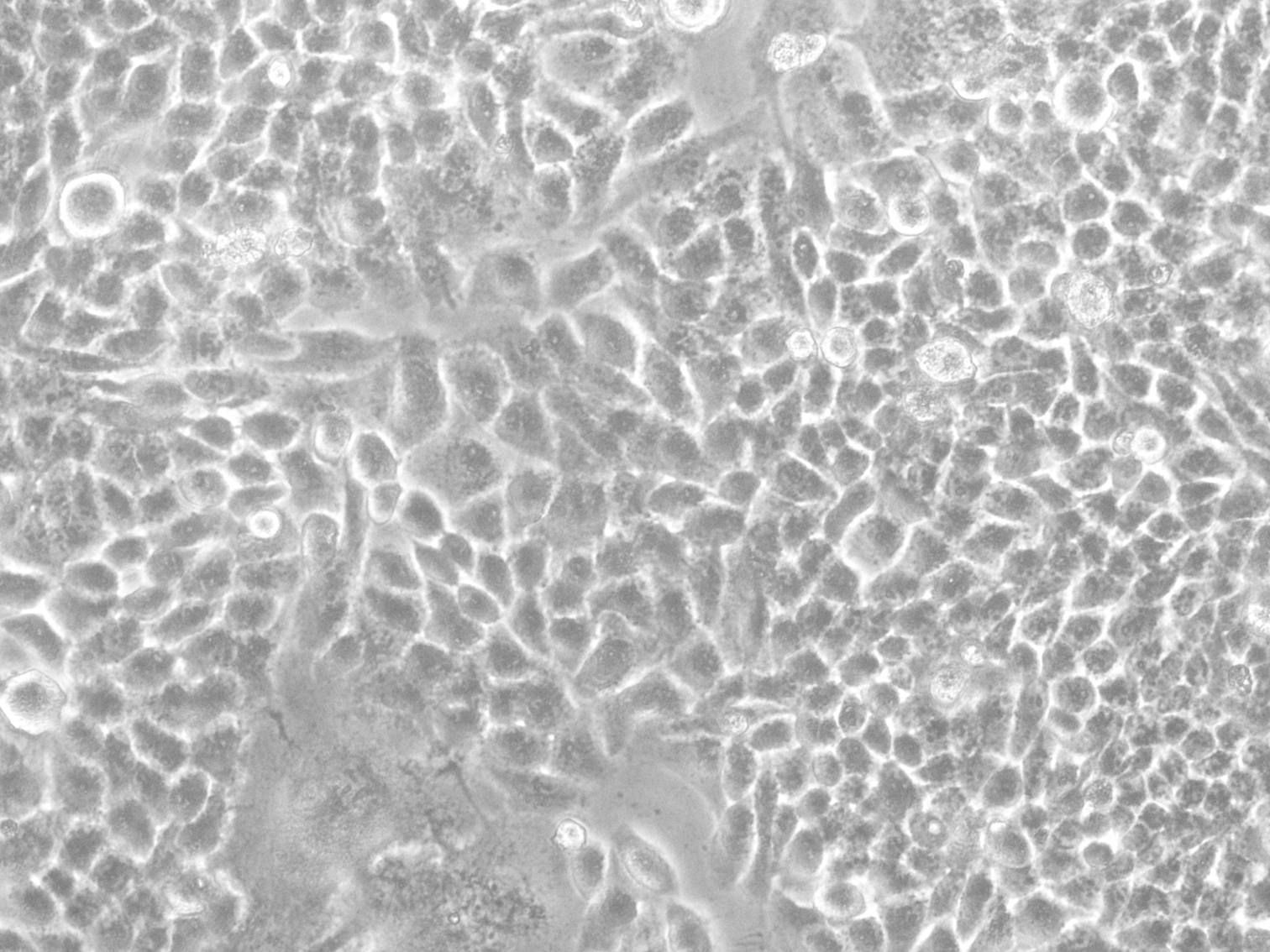

Supplement: Supplementary file 8 — Source data Fig. 6 [file 44319_2024_248_MOESM8_ESM.zip › Figure 6/Fig. 6D/BAG3 WT -DMSO.tif]

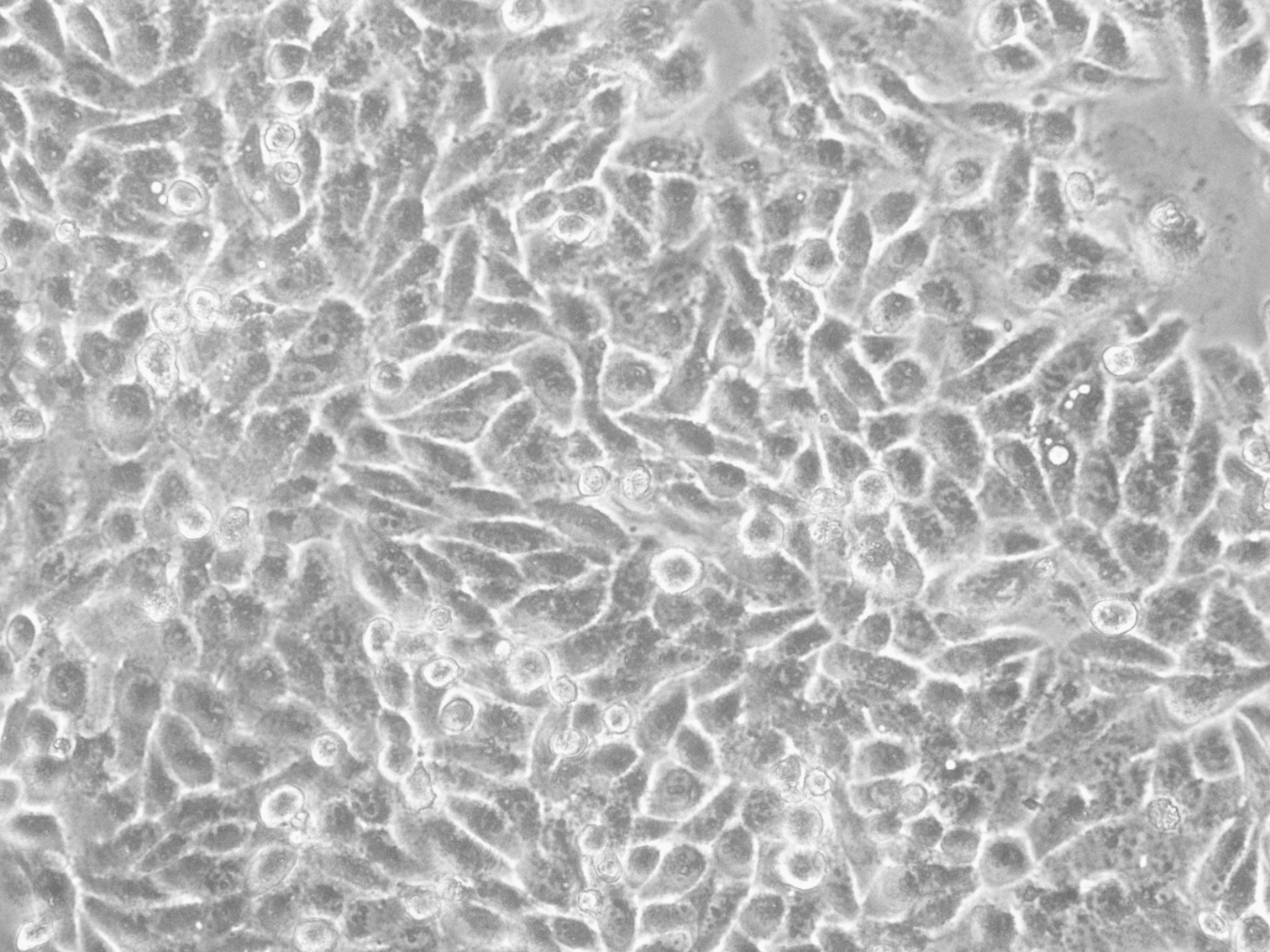

Supplement: Supplementary file 8 — Source data Fig. 6 [file 44319_2024_248_MOESM8_ESM.zip › Figure 6/Fig. 6D/BAG3 WT -MG132.tif]

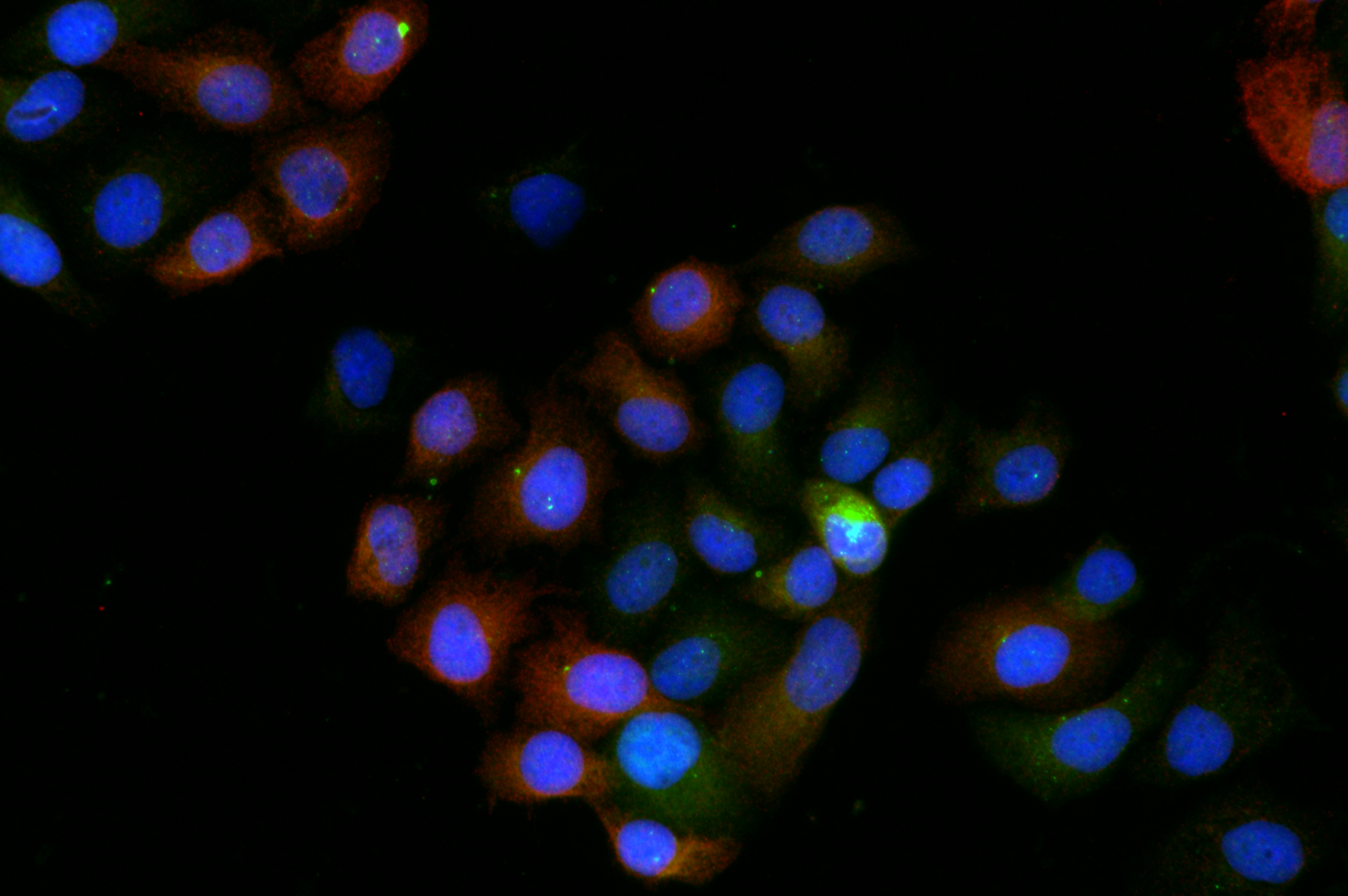

Supplement: Supplementary file 8 — Source data Fig. 6 [file 44319_2024_248_MOESM8_ESM.zip › Figure 6/Fig. 6H/BAG3 3D-DMSO.tif]

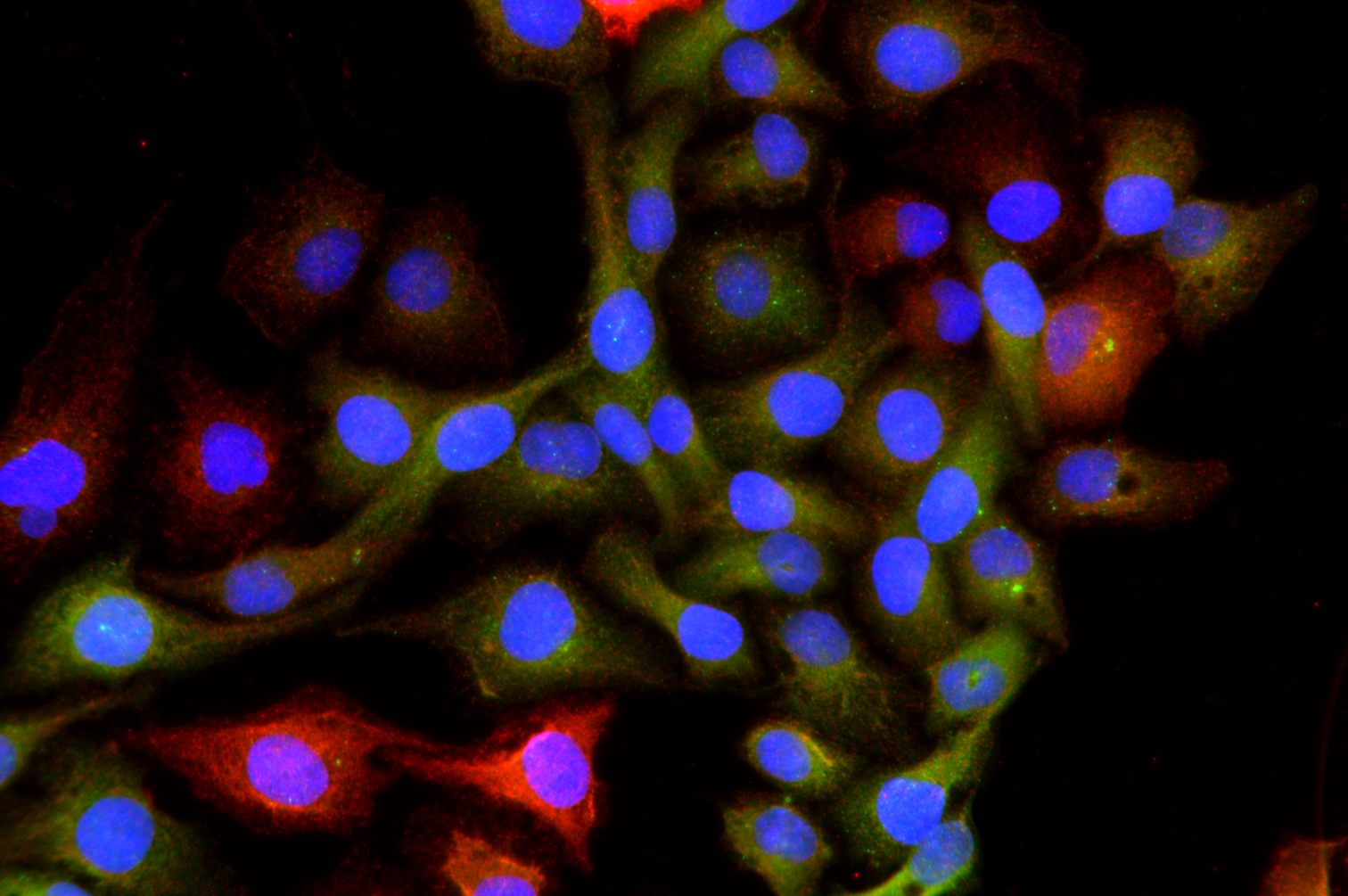

Supplement: Supplementary file 8 — Source data Fig. 6 [file 44319_2024_248_MOESM8_ESM.zip › Figure 6/Fig. 6H/BAG3 3D-MG132.tif]

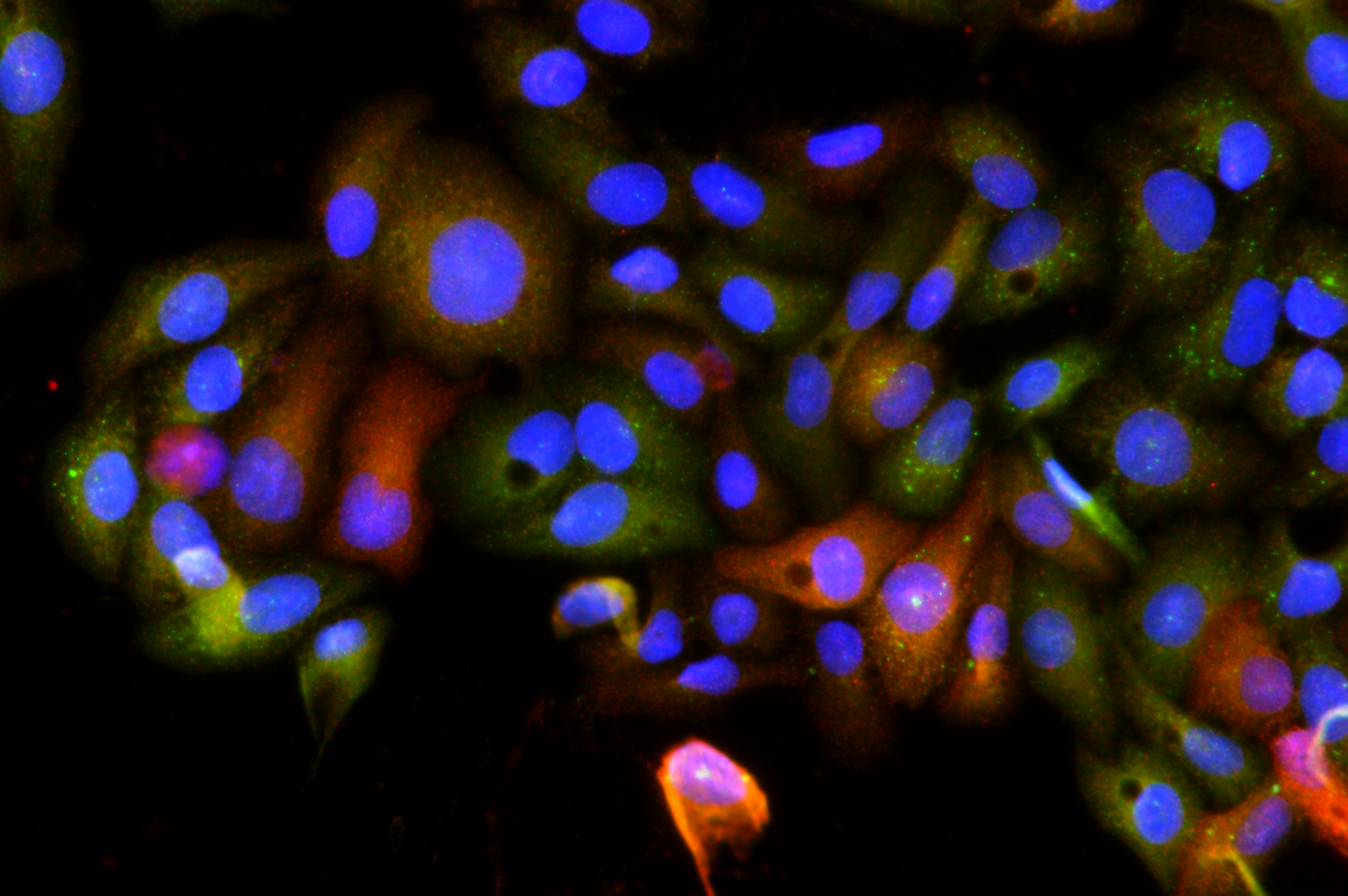

Supplement: Supplementary file 8 — Source data Fig. 6 [file 44319_2024_248_MOESM8_ESM.zip › Figure 6/Fig. 6H/BAG3 3D-MG132+KN-93.tif]

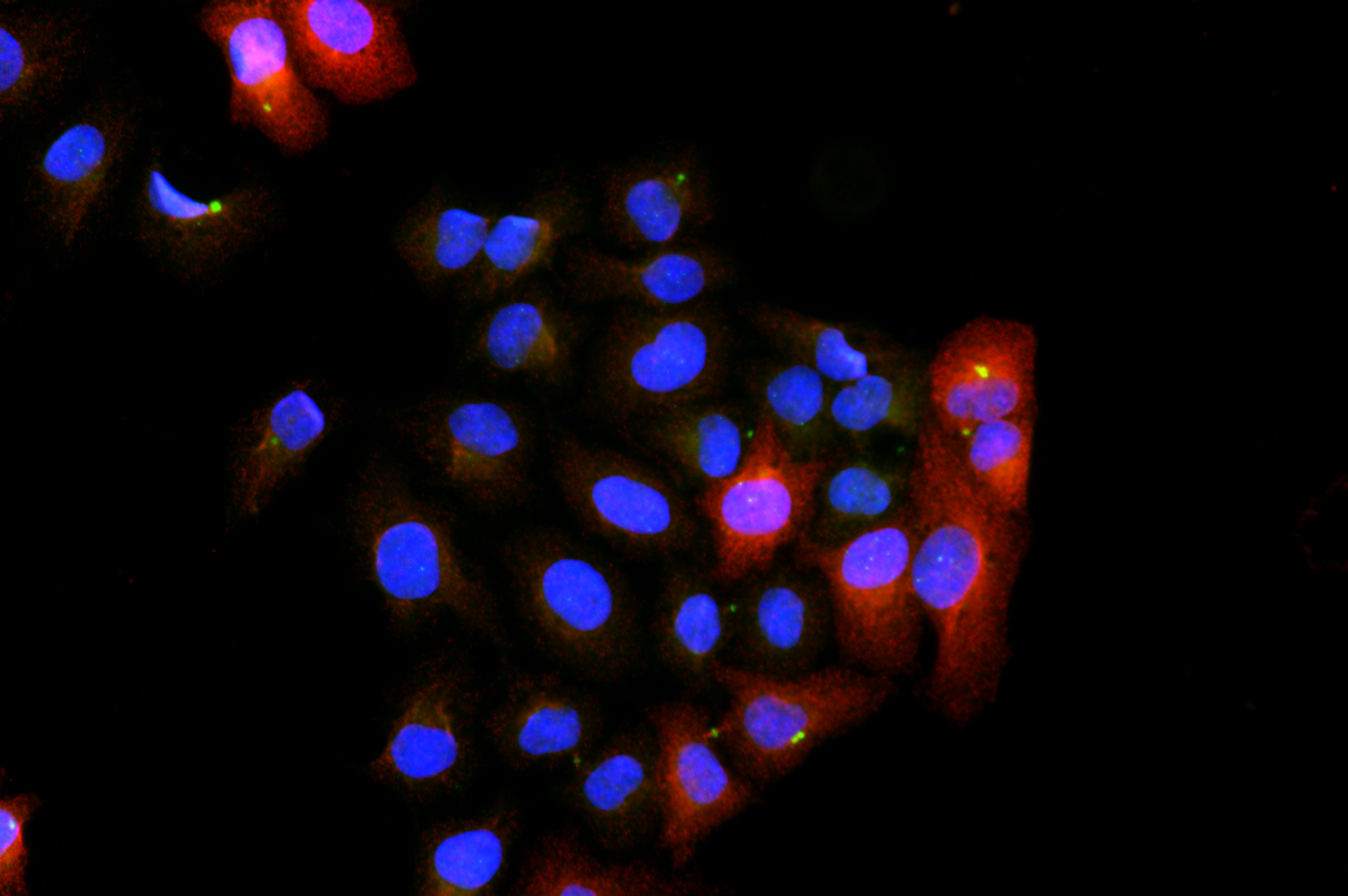

Supplement: Supplementary file 8 — Source data Fig. 6 [file 44319_2024_248_MOESM8_ESM.zip › Figure 6/Fig. 6H/BAG3 WT-DMSO.tif]

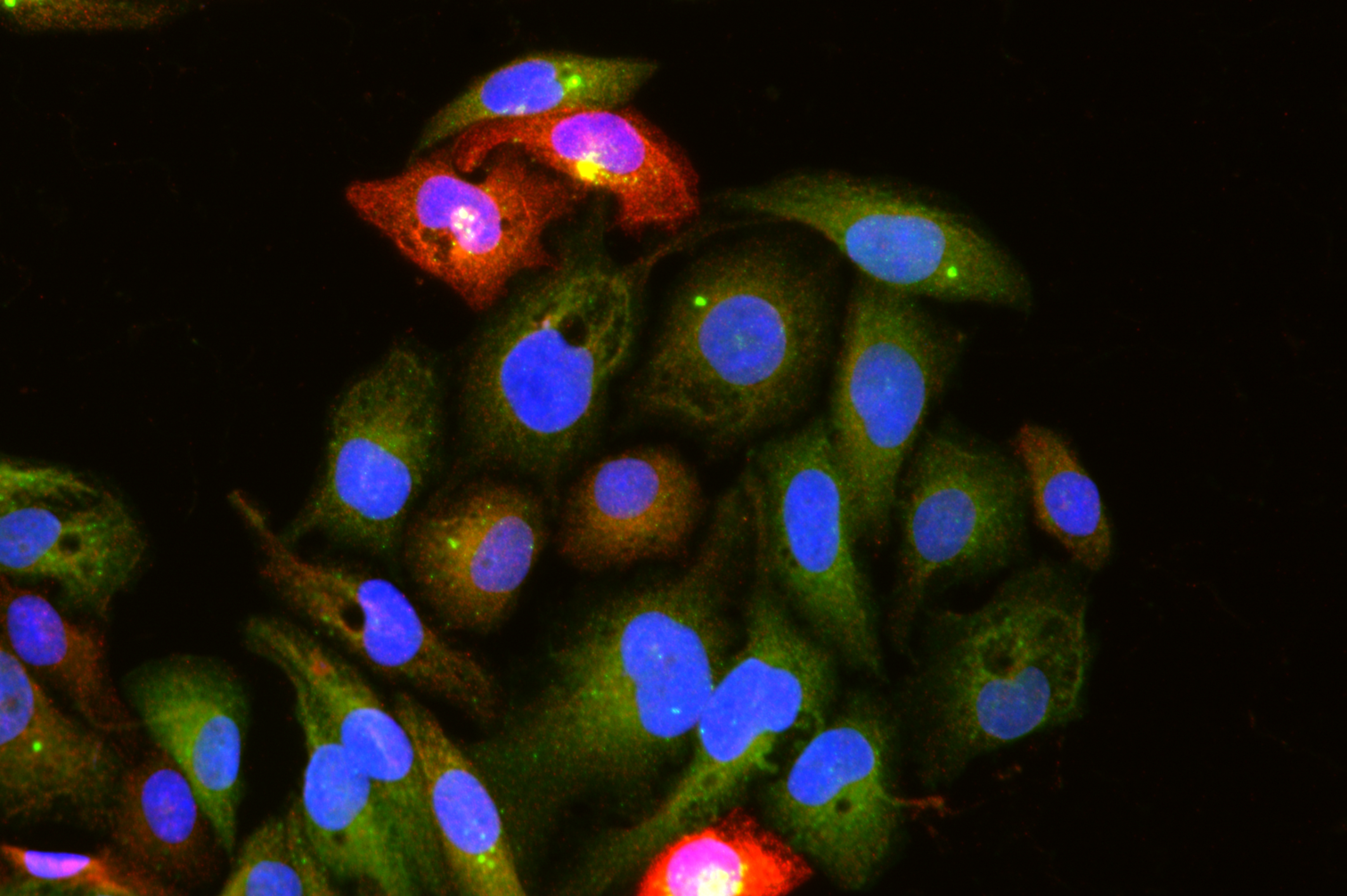

Supplement: Supplementary file 8 — Source data Fig. 6 [file 44319_2024_248_MOESM8_ESM.zip › Figure 6/Fig. 6H/BAG3 WT-MG132.tif]

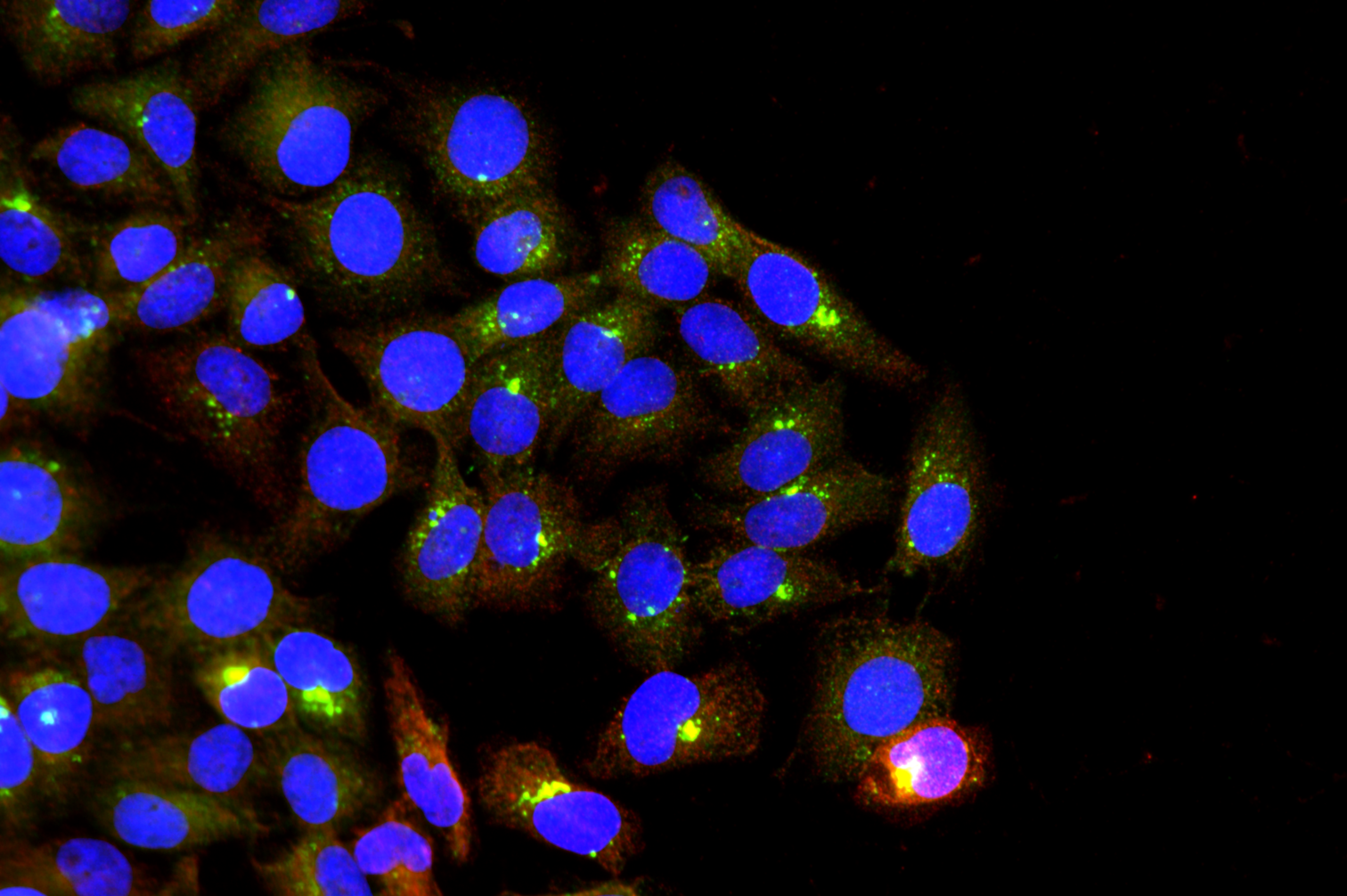

Supplement: Supplementary file 8 — Source data Fig. 6 [file 44319_2024_248_MOESM8_ESM.zip › Figure 6/Fig. 6H/BAG3 WT-MG132+KN-93.tif]

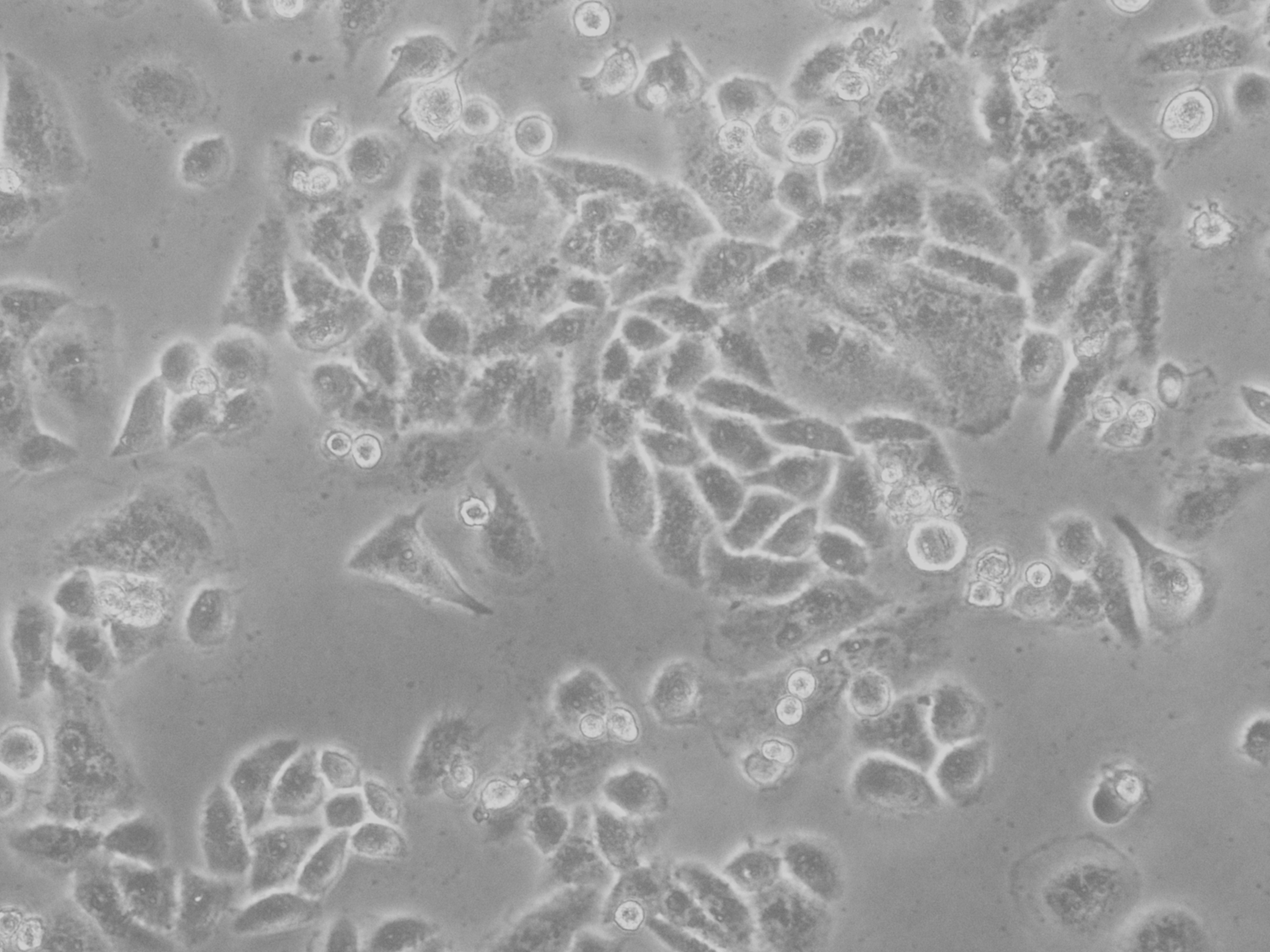

Supplement: Supplementary file 8 — Source data Fig. 6 [file 44319_2024_248_MOESM8_ESM.zip › Figure 6/Fig. 6J/BAG3 3D -DMSO.tif]

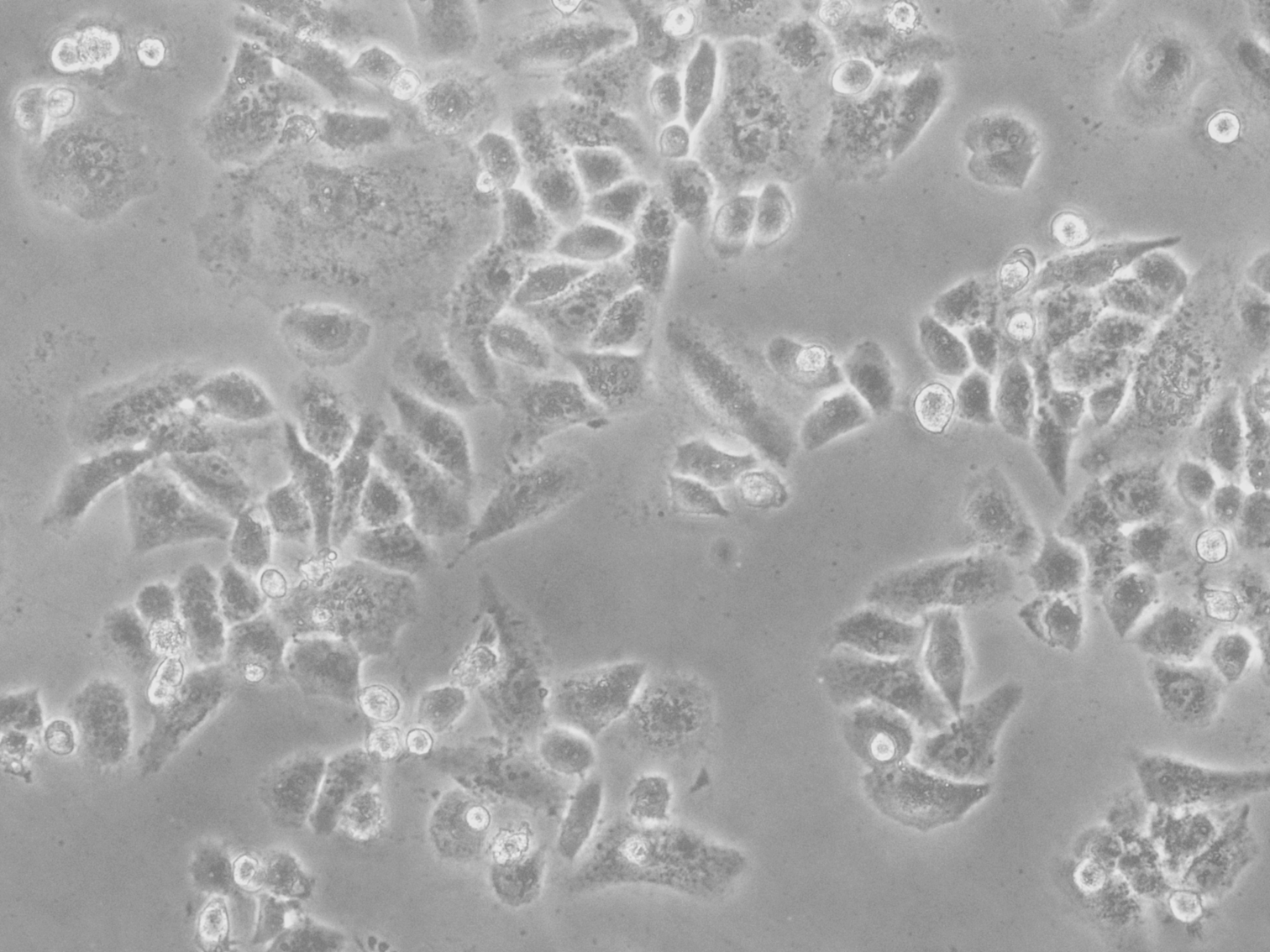

Supplement: Supplementary file 8 — Source data Fig. 6 [file 44319_2024_248_MOESM8_ESM.zip › Figure 6/Fig. 6J/BAG3 3D -MG132.tif]

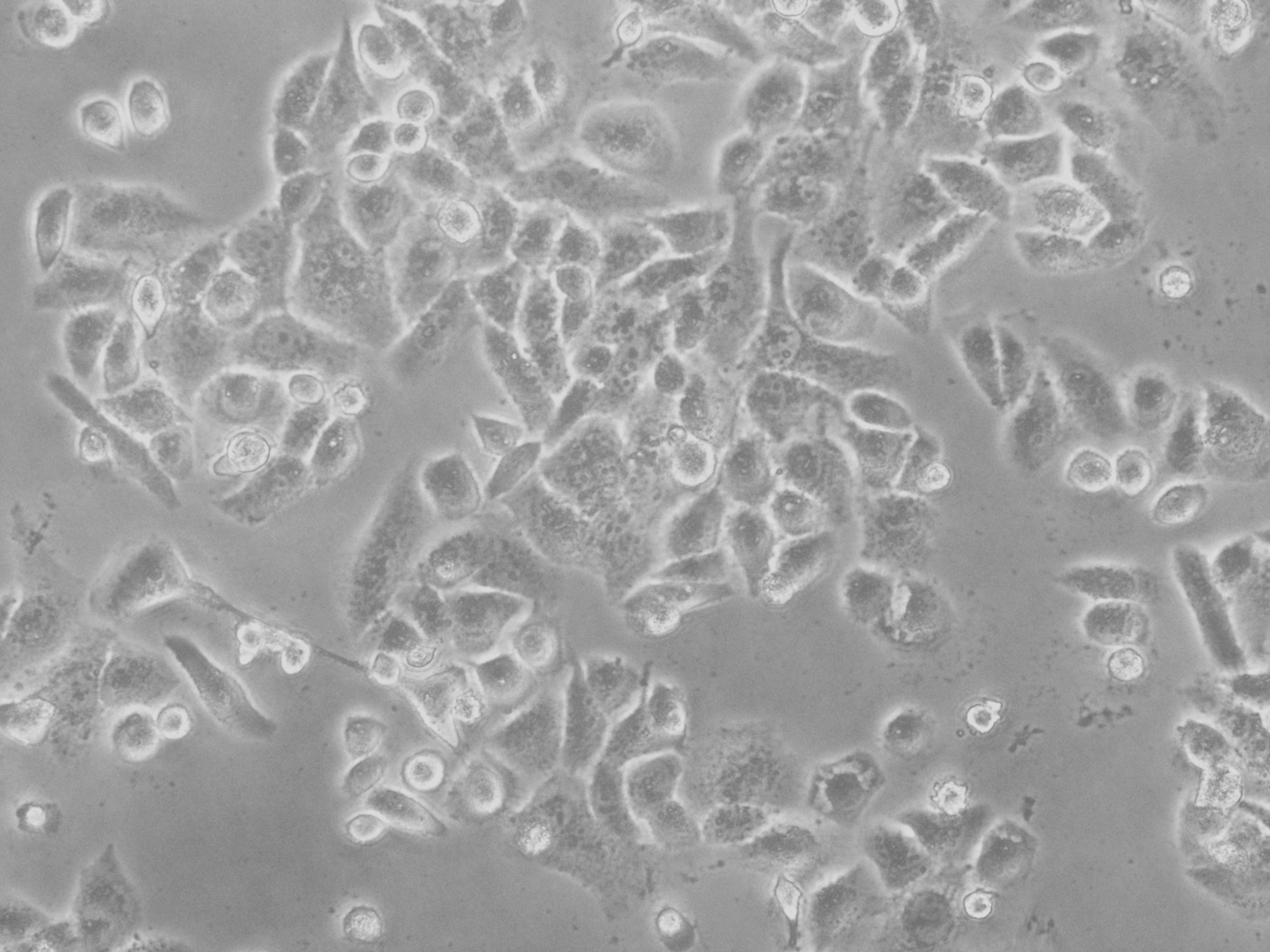

Supplement: Supplementary file 8 — Source data Fig. 6 [file 44319_2024_248_MOESM8_ESM.zip › Figure 6/Fig. 6J/BAG3 3D -MG132+KN-93.tif]

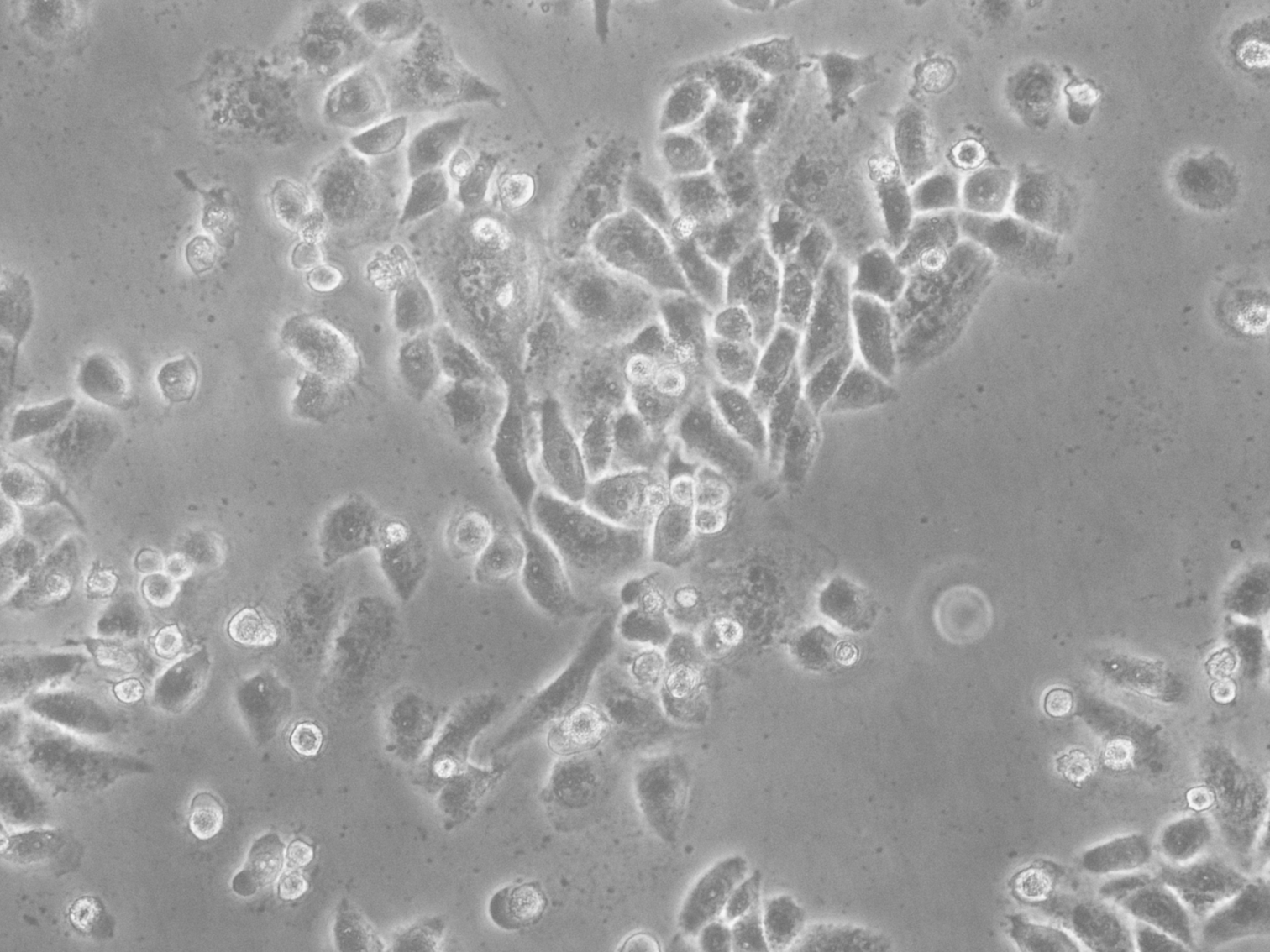

Supplement: Supplementary file 8 — Source data Fig. 6 [file 44319_2024_248_MOESM8_ESM.zip › Figure 6/Fig. 6J/BAG3 WT -DMSO.tif]

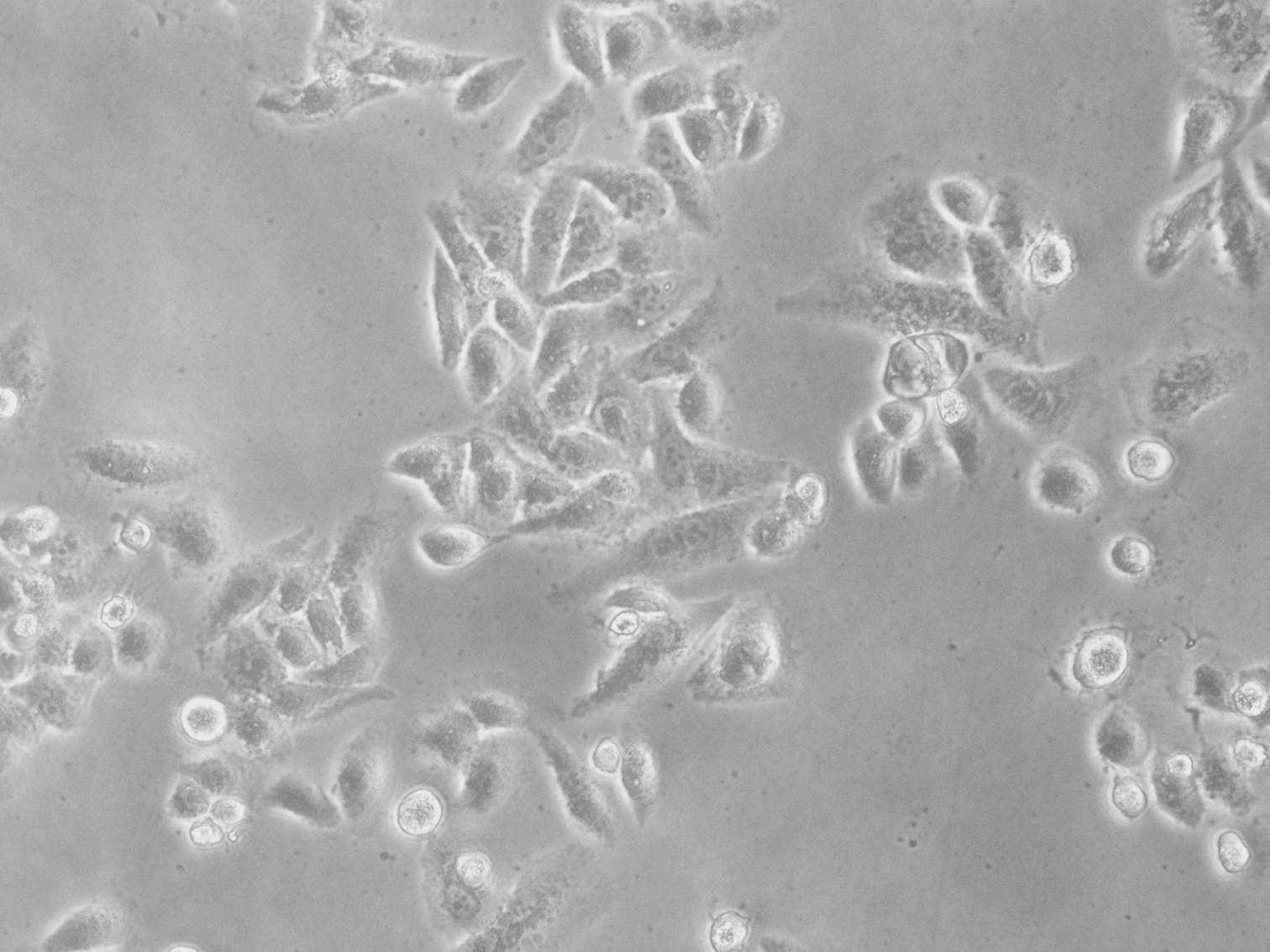

Supplement: Supplementary file 8 — Source data Fig. 6 [file 44319_2024_248_MOESM8_ESM.zip › Figure 6/Fig. 6J/BAG3 WT -MG132.tif]

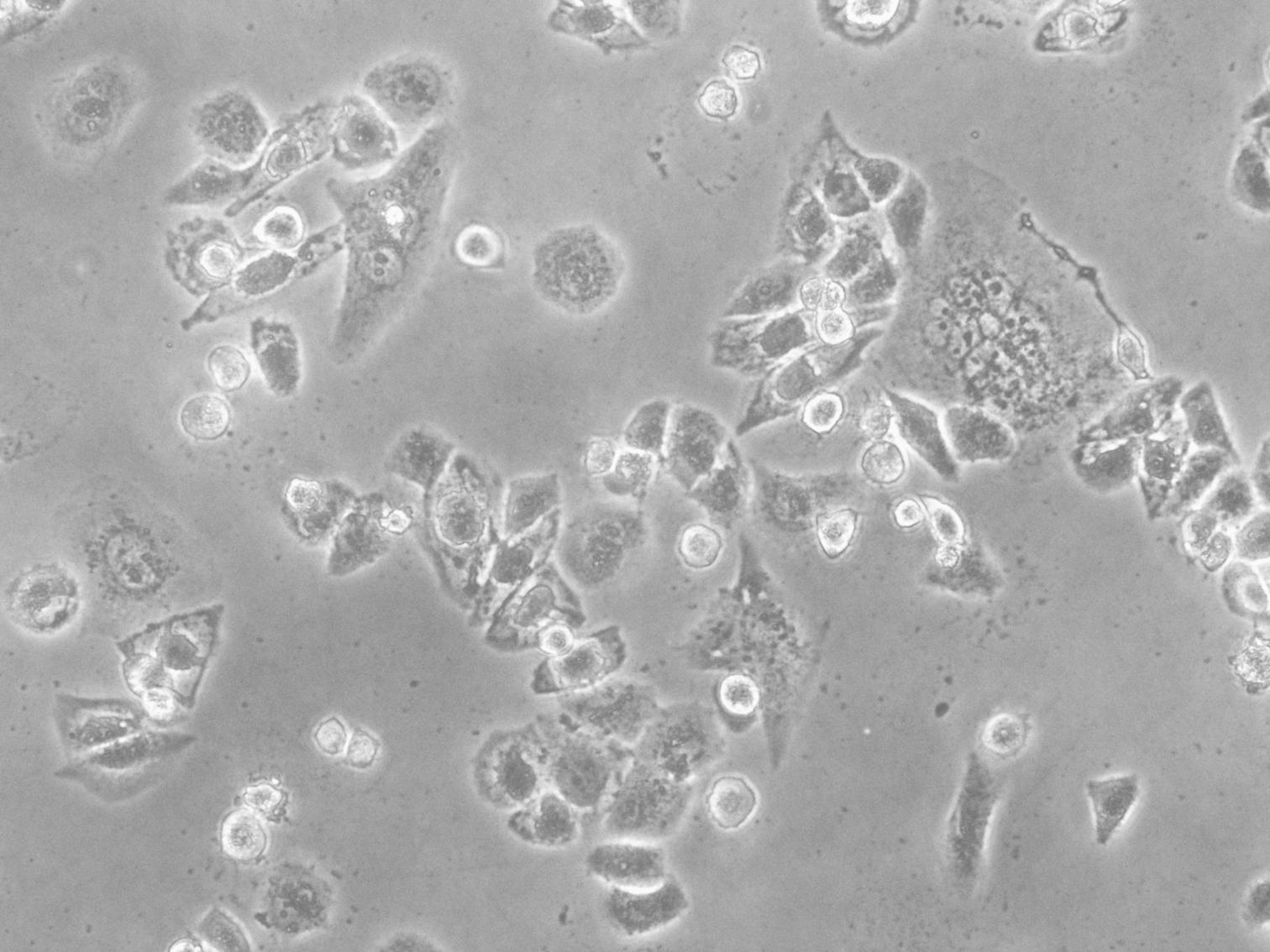

Supplement: Supplementary file 8 — Source data Fig. 6 [file 44319_2024_248_MOESM8_ESM.zip › Figure 6/Fig. 6J/BAG3 WT -MG132+KN-93.tif]

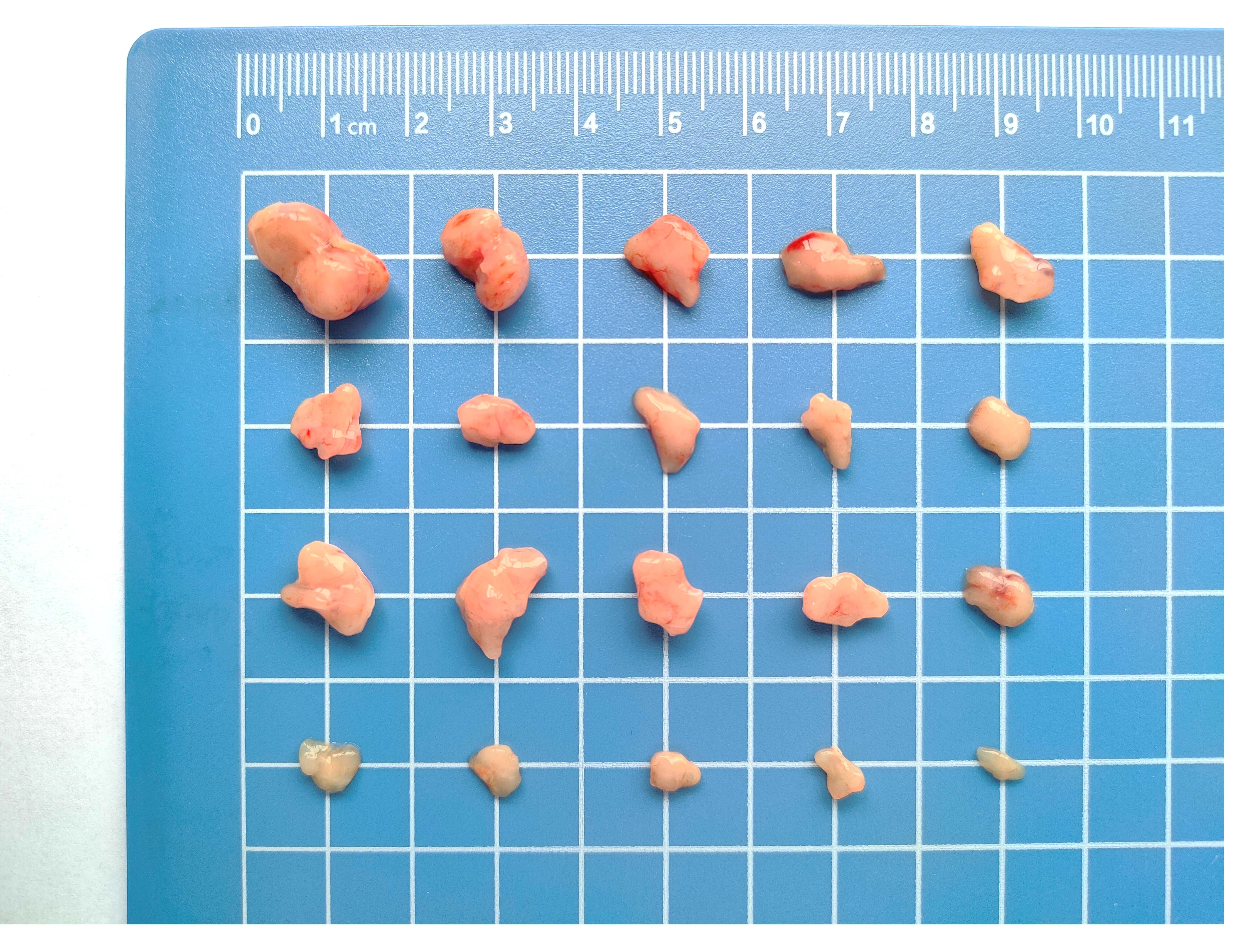

Supplement: Supplementary file 9 — Source data Fig. 7 [file 44319_2024_248_MOESM9_ESM.zip › Figure 7/Fig. 7N/image.tif]
