# Supplementary material for: CaMKII suppresses proteotoxicity by phosphorylating BAG3 in response to proteasomal dysfunction
Source: EMBO Rep. 2024 Sep 11;25(10):4488–514. doi: 10.1038/s44319-024-00248-w (PMC11466968; doi:10.1038/s44319-024-00248-w)
Supplement: Supplementary file 10 — Expanded View Figures [file 44319_2024_248_MOESM10_ESM.pdf]

Expanded View Figures

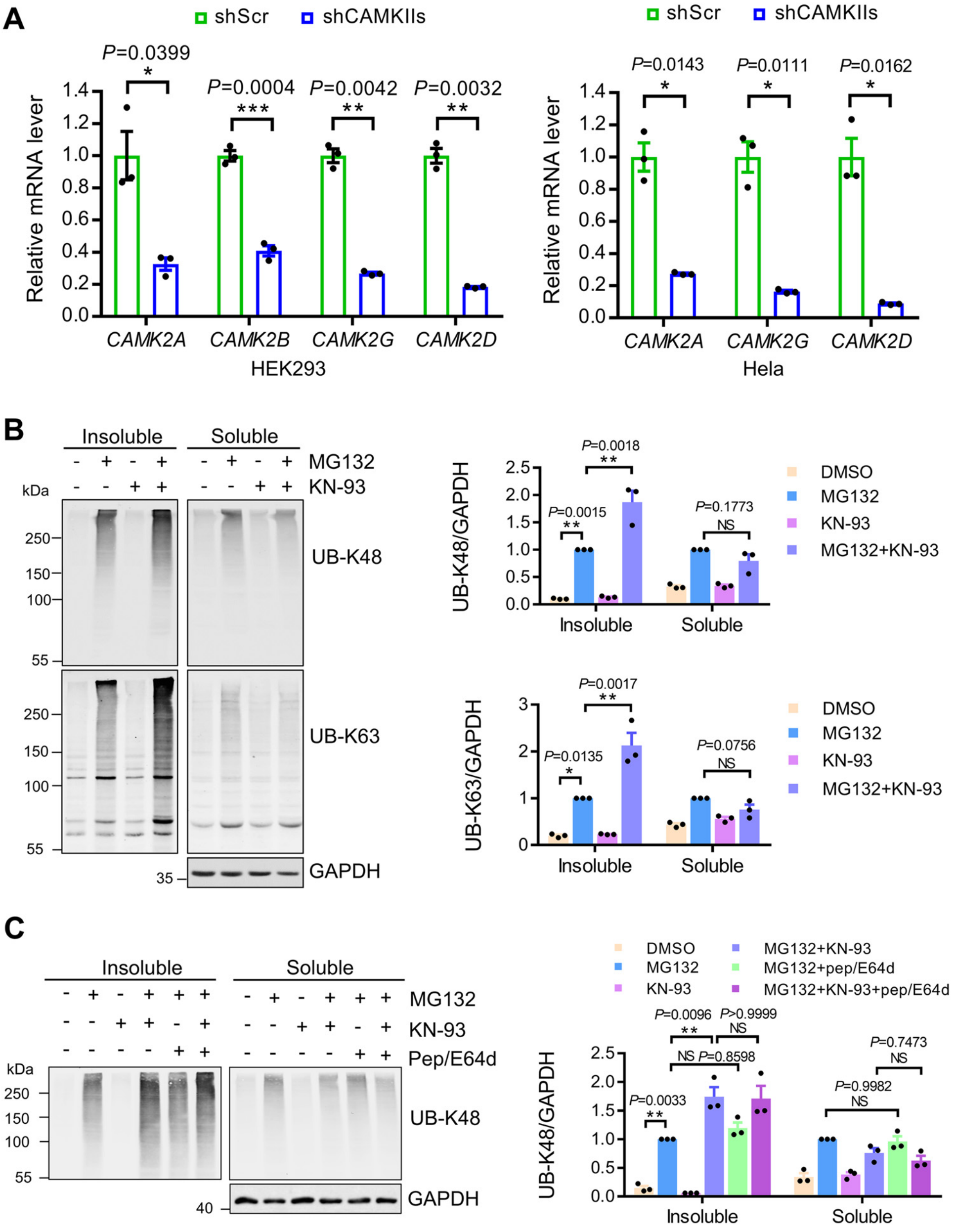

Figure EV1. CaMKII regulates the synthesis of aggregated ubiquitinated proteins in response to proteasome inhibition, related to Fig. 1.

(**A**) Confirmation of *CAMK2* knockdown in HEK293 and HeLa cells. After transfected with indicated plasmids for 48 h, the mRNA levels of *CAMK2* were analyzed by qRT-PCR. Data are mean ± SEM of biological replicates ($n = 3$). The *P* value was determined by Student's *t*-test (two-sided). **P* < 0.05; ***P* < 0.01; ****P* < 0.001.
(**B**) Immunoblotting and quantification of ubiquitin (UB-K48 and UB-K63) in NP-40-soluble and -insoluble fractions of HEK293 cells treated with indicated drugs (MG132 (1 μM), KN-93 (10 μM)) for 14 h. Data are mean ± SEM of biological replicates ($n = 3$). (**C**) Immunoblotting and quantification of UB-K48 in NP-40-soluble and -insoluble fractions of HEK293 cells treated with indicated drugs (MG132 (1 μM), KN-93 (10 μM), Pepstatin A (Pep, 25 μg/ml)/E64d (25 μg/ml)) for 14 h. Data are mean ± SEM of biological replicates ($n = 3$). For (**B**, **C**), the *P* value was determined by a one-way ANOVA analysis. NS not significant; **P* < 0.05; ***P* < 0.01.

 © The Author(s)

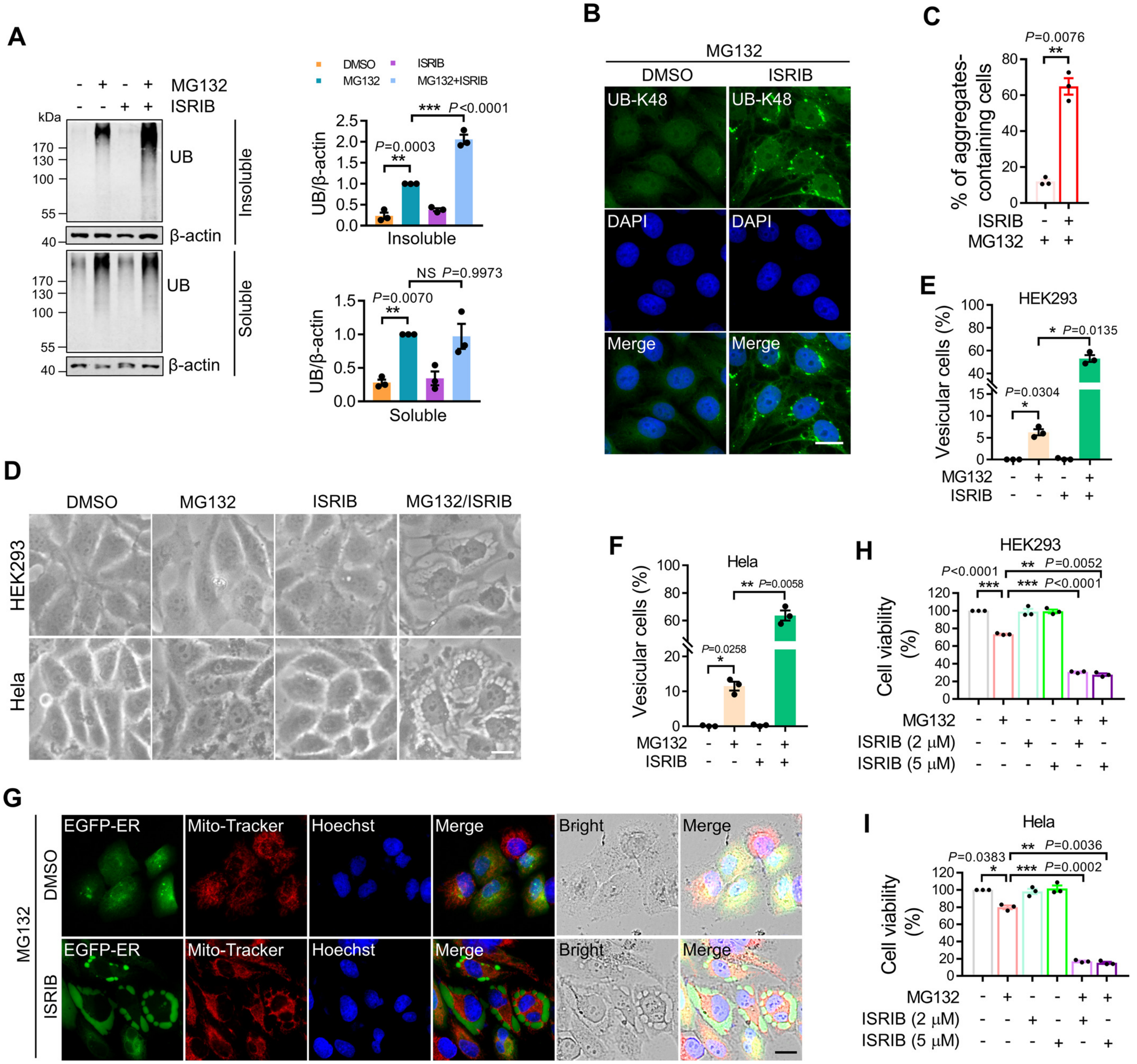

Figure EV2. Inhibiting eIF2α phosphorylation aggravates the proteasome inhibitor-induced proteotoxicity, related to Fig. 3.

(**A**) Immunoblotting and quantification of indicated proteins in NP-40-soluble and -insoluble fractions of HEK293 cells treated with indicated drugs (MG132 (1 μM), ISRIB (5 μM)) for 14 h. Data are mean ± SEM of biological replicates ($n = 3$). (**B**) Representative UB-K48 staining images of HeLa cells treated with indicated drugs (MG132 (0.5 μM), ISRIB (5 μM)) for 14 h. Scale bar: 20 μm. (**C**) Quantitative analysis of results in (**B**). Data are mean ± SEM of biological replicates ($n = 3$). At least 50 cells were randomly selected from each group to score. (**D**) Cell morphology imaging of HEK293 and HeLa cells treated with indicated drugs (MG132 (1 μM), ISRIB (5 μM)) for 24 h. Scale bar: 10 μm. (**E**, **F**) Quantitative analysis of results in (**D**). Data are mean ± SEM of biological replicates ($n = 3$). At least 50 cells were randomly selected from each group to score. (**G**) Representative images of HeLa cells transfected with ER-EGFP and treated with indicated drugs (MG132 (1 μM), ISRIB (5 μM)) for 24 h. Mitochondria and nuclei were stained with Mito-Tracker (red) and Hoechst (blue), respectively. Scale bar: 20 μm. (**H**, **I**) Cell viability of HEK293 and HeLa cells treated with indicated drugs (MG132 (1 μM), ISRIB (2 μM, 5 μM)) for 48 h. Data are mean ± SEM of biological replicates ($n = 3$). For (**C**), the *P* value was determined by Student's *t* test (two-sided). $^{**}P < 0.01$. For (**A**, **E**, **F**, **H**, **I**), the *P* value was determined by a one-way ANOVA analysis. NS not significant; $^{*}P < 0.05$; $^{**}P < 0.01$; $^{***}P < 0.001$.

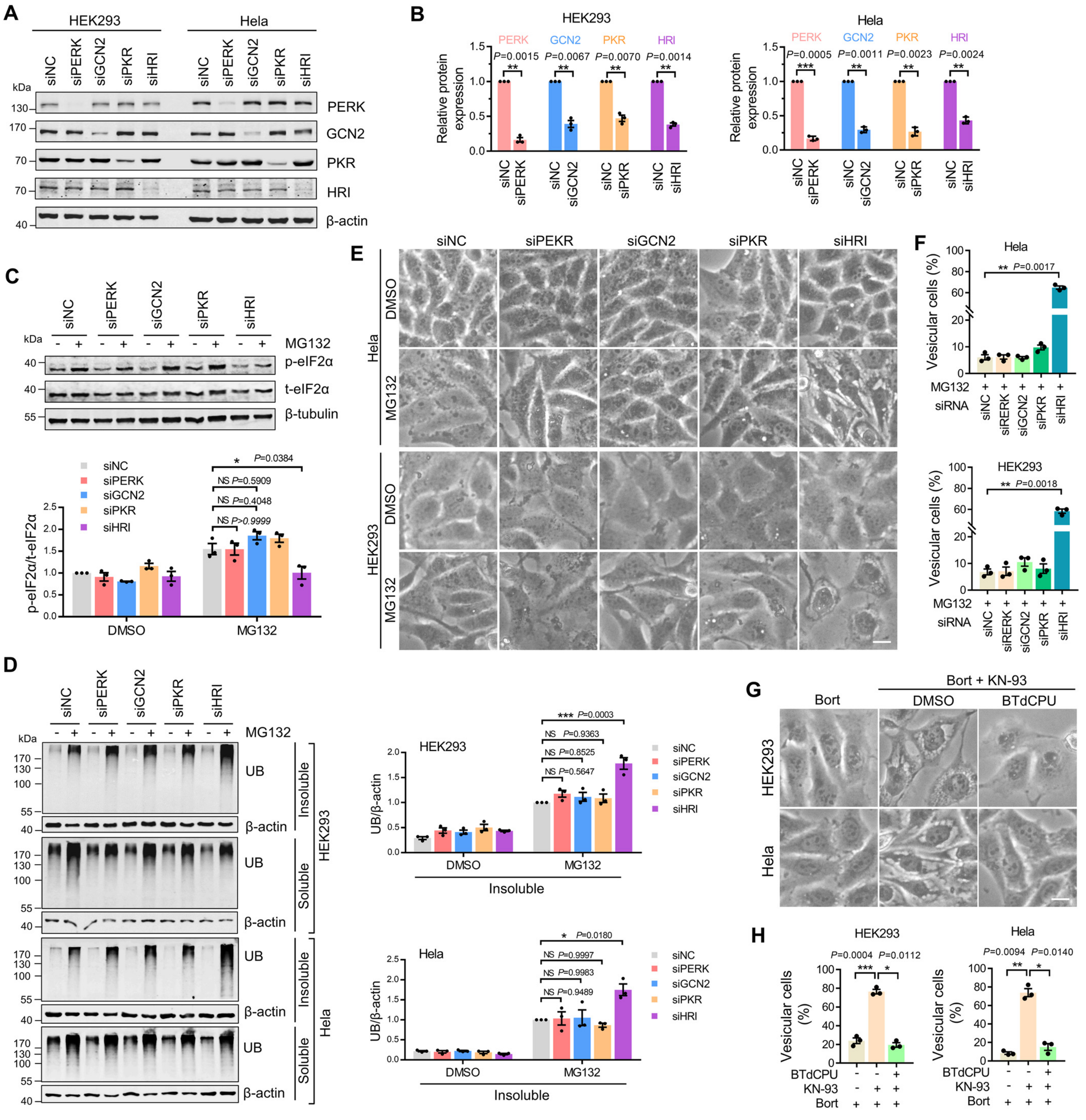

Figure EV3. HRI is critical for eIF2α activation and proteotoxicity reduction during proteasome dysfunction, related to Fig. 3.

(**A**) Confirmation of EIF2AKs knockdown in HEK293 and HeLa cells. After transfected with indicated siRNAs, the whole cell lysates were subjected to western blot analysis with indicated antibodies. (**B**) Quantitative analysis of results in (**A**). Data are mean ± SEM of biological replicates ($n = 3$). (**C**) Immunoblotting and quantification of p-eIF2α (S51) in HeLa cells transfected with indicated siRNAs and treated with MG132. Data are mean ± SEM of biological replicates ($n = 3$). (**D**) Immunoblotting and quantification of UB in NP-40-soluble and -insoluble fractions of HEK293 and HeLa cells transfected with indicated siRNAs and treated with MG132. Data are mean ± SEM of biological replicates ($n = 3$). (**E**) Cell morphology imaging of HEK293 and HeLa cells transfected with indicated siRNAs and treated with MG132. Scale bar: 10 μm. (**F**) Quantitative analysis of results in (**E**). Data are mean ± SEM of biological replicates ($n = 3$). At least 50 cells were randomly selected from each group to score. (**G**) Cell morphology imaging of HEK293 and HeLa cells treated with indicated drugs (Bortezomib (1 μM), KN-93 (10 μM), BTdCPU (0.5 μM)) for 24 h. Scale bar: 10 μm. (**H**) Quantitative analysis of results in (**G**). Data are mean ± SEM of biological replicates ($n = 3$). At least 50 cells were randomly selected from each group to score. For (**B**), the P value was determined by Student's t test (two-sided). $^{**}P < 0.01$; $^{***}P < 0.001$. For (**C**, **D**, **F**, **H**), the P value was determined by a one-way ANOVA analysis. NS not significant; $^{*}P < 0.05$; $^{**}P < 0.01$; $^{***}P < 0.001$.

© The Author(s)

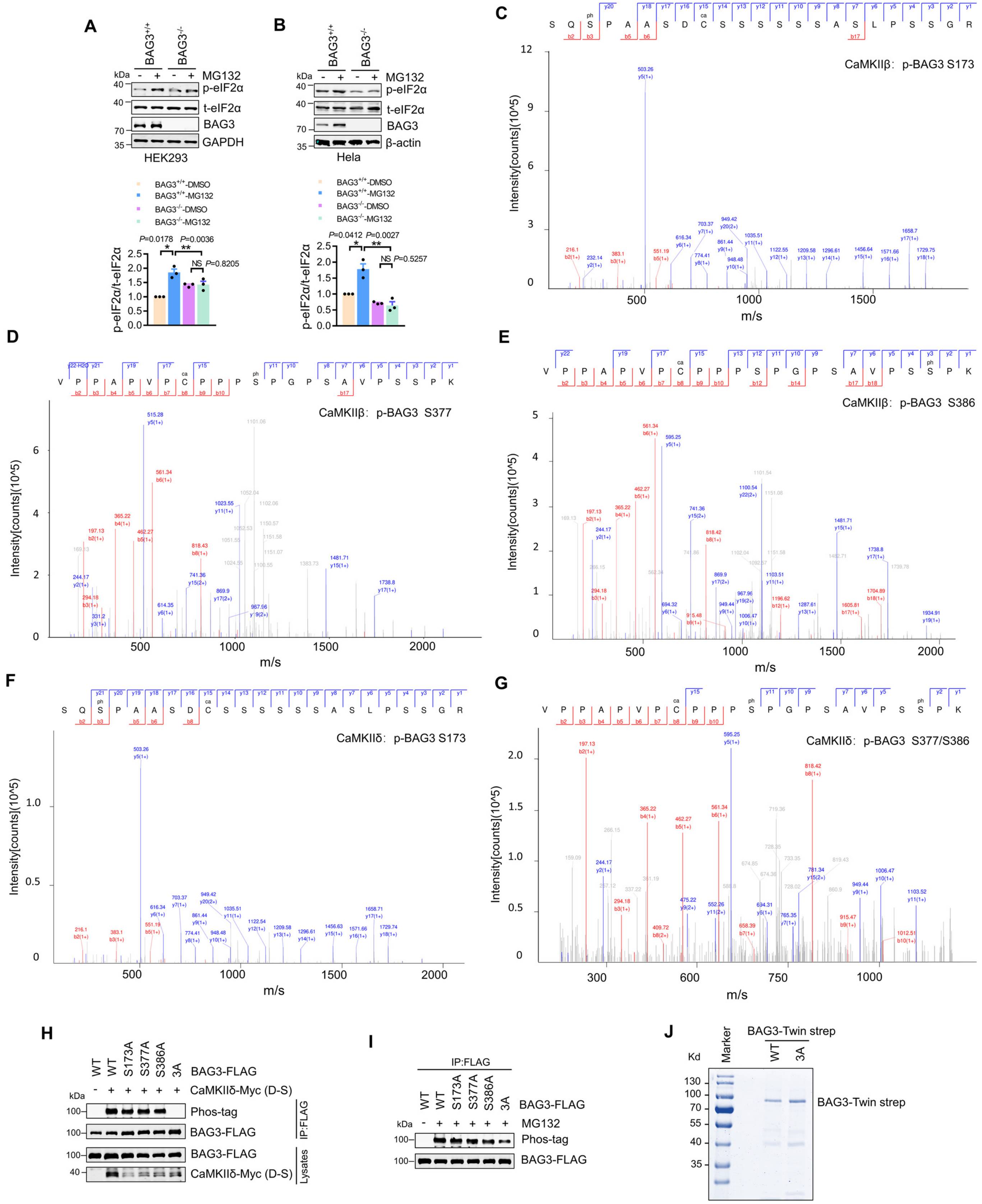
A
BAG3+/+
BAG3-/-
kDa
MG132
p-eIF2α
t-eIF2α
BAG3
GAPDH
HEK293
BAG3+/+-DMSO
BAG3+/+-MG132
BAG3-/--DMSO
BAG3-/--MG132
P=0.0178 P=0.0036
NS P=0.8205
p-eIF2α/t-eIF2α
B
BAG3+/+
BAG3-/-
MG132
p-eIF2α
t-eIF2α
BAG3
β-actin
Hela
P=0.0412 P=0.0027
NS P=0.5257
p-eIF2α/t-eIF2α
C
CaMKIIβ: p-BAG3 S173
Intensity[counts](10^5)
m/s
D
CaMKIIβ: p-BAG3 S377
Intensity[counts](10^5)
m/s
E
CaMKIIβ: p-BAG3 S386
Intensity[counts](10^5)
m/s
F
CaMKIIδ: p-BAG3 S173
Intensity[counts](10^5)
m/s
G
CaMKIIδ: p-BAG3 S377/S386
Intensity[counts](10^5)
m/s
H
WT WT S173A S377A S386A 3A BAG3-FLAG
CaMKIIδ-Myc (D-S)
kDa
Phos-tag
BAG3-FLAG
IP:FLAG
BAG3-FLAG
CaMKIIδ-Myc (D-S)
Lysates
I
IP:FLAG
WT WT S173A S377A S386A 3A BAG3-FLAG
kDa
MG132
Phos-tag
BAG3-FLAG
J
Marker
BAG3-Twin strep
WT 3A
Kd
BAG3-Twin strep

Figure EV4. CaMKII phosphorylates BAG3, related to Fig. 4.

(**A**, **B**) Immunoblotting and quantification of p-eIF2α (S51) in BAG3 knockout cells treated with 1 µM MG132 for 14 h. Data are mean ± SEM of biological replicates ($n = 3$). The P value was determined by Student's t test (two-sided). NS not significant; $^*P < 0.05$; $^{**}P < 0.01$. (**C–G**) Mass spectrum of the phosphopeptide containing the S173/S377/S386 phosphorylated residue of BAG3. (**H**, **I**) Phosphorylation level of re-expressed BAG3 (WT or mutants) in $BAG3^{-/-}$ HEK293 cells treated with MG132 (**I**) or not (**H**). (**J**) Confirmation of purified BAG3-Twin Strep (WT or 3A) by coomassie blue staining.

 © The Author(s)

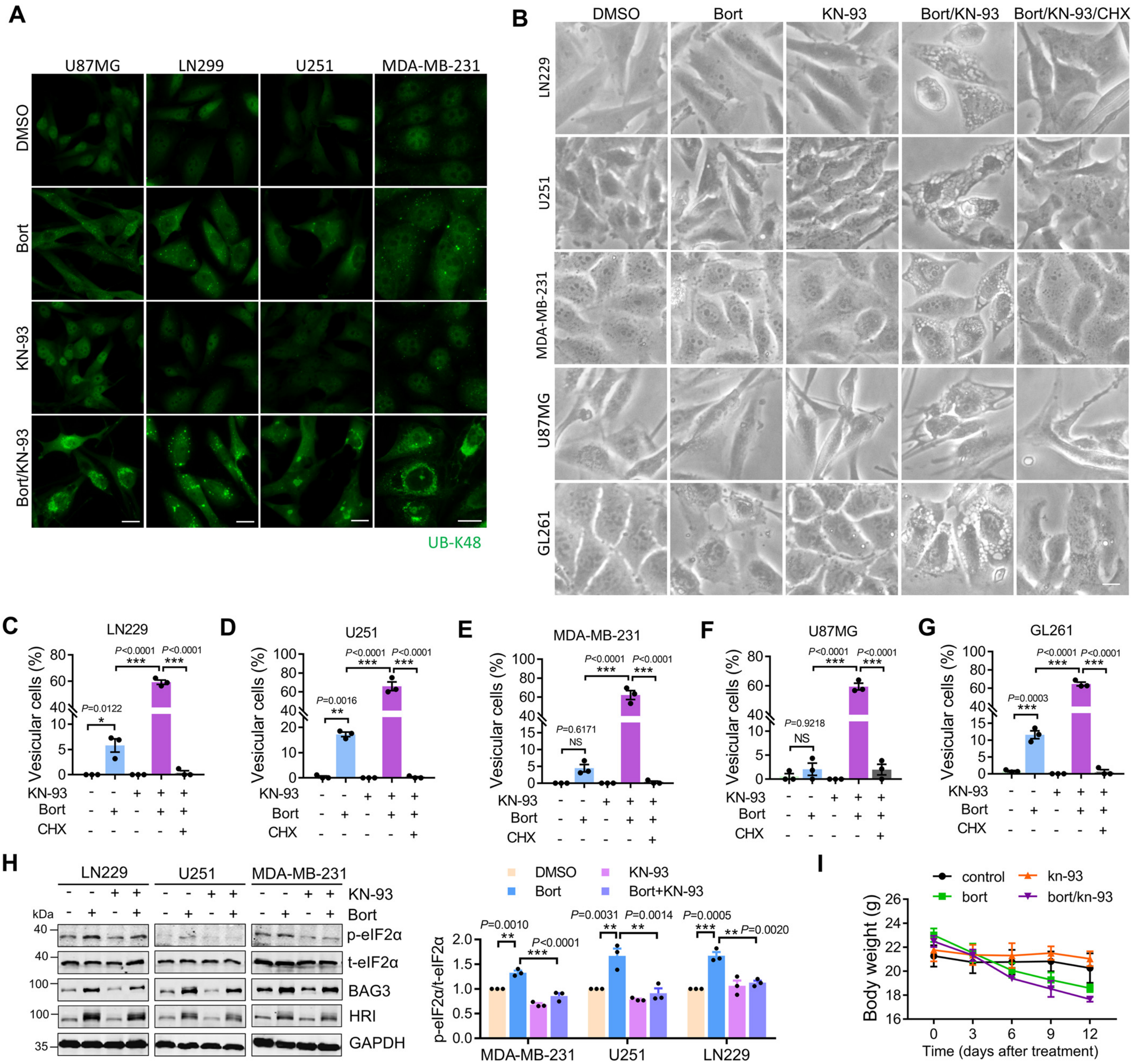

Figure EV5. Inhibition of CaMKII enhances the anti-tumor activity of proteasome inhibitor, related Fig. 7.

(**A**) Representative UB-K48 staining images of tumor cells treated with indicated drugs. Scale bar: 20 µm. (**B**) Cell morphology imaging of tumor cells treated with indicated drugs (Bortezomib (10 nM), KN-93 (10 µM), CHX (25 µg/ml)) for 24 h. Scale bar: 10 µm. (**C–G**) Quantitative analysis of results in (**B**). Data are mean ± SEM of biological replicates ($n = 3$). At least 50 cells were randomly selected from each group to score. (**H**) Immunoblotting and quantification of indicated proteins in tumor cells treated with indicated drugs. Data are mean ± SEM of biological replicates ($n = 3$). (**I**) The body weight of each group was calculated once time every three days. Data are mean ± SEM of 5 mice. For (**C–H**), the *P* value was determined by a one-way ANOVA analysis. NS not significant; *$P < 0.05$; **$P < 0.01$; ***$P < 0.001$.
